# Pd-catalyzed site-selective C(sp^2^)–H radical acylation of phenylalanine containing peptides with aldehydes†
†Electronic supplementary information (ESI) available: Experimental procedures, data for new compounds, and ^1^H and ^13^C NMR spectra. CCDC 1939205 and 1939206. For ESI and crystallographic data in CIF or other electronic format see DOI: 10.1039/c9sc03425k


**DOI:** 10.1039/c9sc03425k

**Published:** 2019-08-07

**Authors:** Marcos San Segundo, Arkaitz Correa

**Affiliations:** a University of the Basque Country (UPV/EHU) , Department of Organic Chemistry I , Joxe Mari Korta R&D Center, Avda. Tolosa 72 , 20018 Donostia-San Sebastián , Spain . Email: arkaitz.correa@ehu.eus

## Abstract

The site-selective functionalization of C–H bonds within a peptide framework remains a challenging task of prime synthetic importance. Herein, the first Pd-catalyzed δ-C(sp^2^)–H acylation of Phe containing peptides with aldehydes is described. This oxidative coupling is distinguished by its site-specificity, tolerance of sensitive functional groups, scalability, and enantiospecificity and exhibits entire chemoselectivity for Phe motifs over other amino acid units. The compatibility of this dehydrogenative acylation platform with a number of oligopeptides of high structural complexity illustrates its ample opportunities for the late-stage peptide modification and bioconjugation.

## Abstract 2

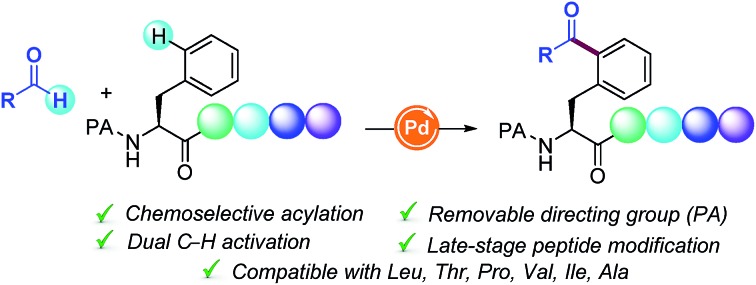
A novel Pd-catalyzed δ-C(sp^2^)–H functionalization reaction with readily available aldehydes towards the assembly of non-proteogenic acylated Phe-containing oligopeptides is presented.

## Introduction

**Scheme 1:**
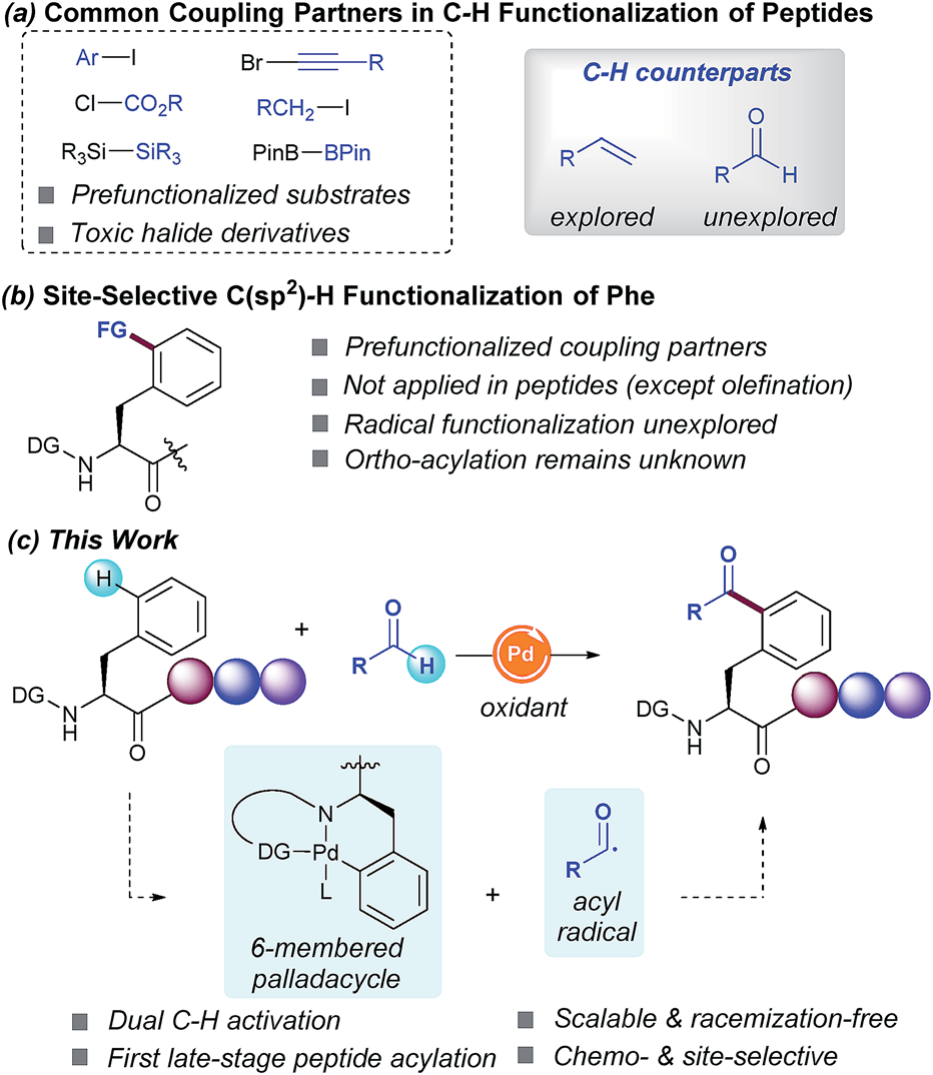
C(sp^2^)–H functionalization of peptides.

Driven by their enhanced biological activities and often improved pharmacokinetics compared with their native counterparts, non-natural amino acids and peptides derived thereof have lately emerged as powerful scaffolds in proteomics and drug discovery.^1^ As a result, recent years have witnessed tremendous interest in the site-specific chemical modification of peptides for the ultimate precise engineering of proteins.^2^ In this regard, transition-metal catalysis has played a critical role in bioconjugation^3^ and recently unlocked new paradigms for the site-selective C–H functionalization of peptides.^2^ The latter has altered the landscape of peptide modification strategies, thus clearly complementing classical techniques from an atom- and step-economic standpoint and allowing the sustainable manipulation of otherwise unreactive C–H bonds.^4^ However, despite the remarkable advances realized, the available functionalization portfolio in these endeavors primarily relies on toxic halide derivatives as coupling partners (Scheme 1, route a), hence reinforcing a change in the strategy to implement more versatile C–H counterparts.

The functionalization of C(sp^3^)–H bonds has been extensively studied and a number of functional groups (FG) can be selectively introduced into the α-amino acid backbone^5^ as well as in the β-, γ- and δ-positions within the hydrocarbon side-chains.^6^ In sharp contrast, relatively few methods are available for the parent C(sp^2^)–H functionalization of aromatic side chains of peptides. Although the modification of tryptophan-containing peptides has proven to be a rather explored avenue,^7^ the diversification of phenylalanine (Phe) residues remains comparatively unexplored. In fact, just a few isolated examples for the modification of simple Phe units are known to date, but they have not been applied within a challenging peptide framework. The most studied technique is the Pd-catalyzed δ-C(sp^2^)–H olefination introduced by Yu in simple systems,8*h* and recently elegantly extended to peptides and cyclopeptides by Cross9*a* and Wang,9*c* respectively (Scheme 1, route b). In this light, we envisioned that the introduction of novel, yet atom-economical C–H coupling partners could enrich our chemical toolbox for the rarely explored late-stage modification of Phe-containing peptides, thus streamlining the rapid assembly of biomolecules of paramount relevance and providing access to novel α-amino acids and peptides beyond those found in naturally occurring proteins.

Radical chemistry has recently flourished into a key technique for creating molecular complexity.^10^ However, the radical functionalization of peptides based on inner-sphere reaction mechanisms has thus far remained elusive.^11^ In this respect, inspired by the emerging trends in radical reactions,^10^ we sought to exploit the practical use of aldehydes as versatile and cost-efficient radical sources.^12^ Although the metal-catalyzed directed C(sp^2^)–H acylation has been previously studied, its application as a late-stage functionalization tool within a peptide framework remains unknown. Based on the commonly accepted Pd^II^/Pd^IV^ manifold,12e,f we anticipated that the judicious choice of the directing group (DG) would be crucial for achieving high positional selectivity upon the formation of a 6-membered palladacycle prone to undergo further addition of the corresponding acyl radical species. Likewise, avoidance of undesired decarbonylation12*a* of the transient acyl radical species poses a crucial challenge. If successful, such a conceptually simple strategy would result in the virtually unexplored carbon-centered radical acylation for the late-stage introduction of ketone motifs within peptides in a predictable and efficient manner (Scheme 1, route c). As part of our interest in sustainable catalysis, herein we report the first Pd-catalyzed site-selective C(sp^2^)–H acylation of Phe containing peptides with aldehydes. The salient features of our method include high chemoselectivity, broad group tolerance, scalability, retention of the native chirality, predictable site-selectivity and facile removal of the required DG.

## Results and discussion

As a proof-of-concept with a simple system, we began our investigations by selecting the acylation of picolinamide (PA)-protected L–Phe–OMe (**1a**) with *p*-tolyl aldehyde (**2a**) as the model reaction. This auxiliary was originally introduced by Daugulis^13^ and has demonstrated superior directing abilities to enable a number of transformations in the realm of C–H activation, including a variety of C(sp^3^)–H modifications of peptides.6a–c,8c–e After systematic evaluation of all reaction parameters,^14^ we found that the desired transformation was feasible and the corresponding acylated compound **3a** was obtained in 79% yield when a combination of Pd(OAc)_2_ as the catalyst, dicumyl peroxide (DCP) as the oxidant, Ag_2_CO_3_ as the additive, and DMF as the solvent was used (Table 1, entry 1). Although **3a** was obtained as a mixture of mono- and diacylated products (**3a** : **3a′**, 7 : 3 ratio), an optimal balance between yield and mono-selectivity was successfully achieved. Since oxidation of the aldehyde **2a** to the corresponding benzoic acid was often observed, an excess of **2a** was required in order to achieve full conversion. Importantly, neither undesired radical acylation on the α-C(sp^3^)–H bond of the Phe backbone11*d* nor the alkylation upon a decarbonylation reaction pathway12*a* was ever observed. As expected, control experiments verified the crucial role of both the Pd catalyst and oxidant as not even traces of **3a** were detected in their absence (entries 2 and 3, respectively).

**Table 1:** Pd-catalyzed δ-C(sp^2^)–H acylation of **1a** with *p*-tolylaldehyde^*a*^

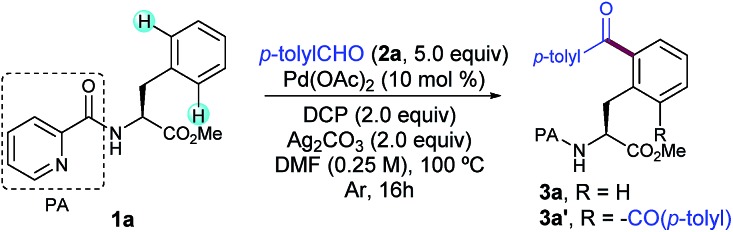		
Entry	Change from standard conditions	**3a** ^*b*^ (%)
1	None	79 (7 : 3)^*c*^ ^,^^*d*^
2	Without Pd(OAc)_2_	0
3	Without DCP	0
4	Without Ag_2_CO_3_	56 (8 : 2)^*c*^
5	Under air	66 (8 : 2)^*c*^
6	DMA instead of DMF	78 (6 : 4)^*c*^
7	6.0 equiv. of **2a**	88 (6 : 4)^*c*^
8	K_2_S_2_O_8_ instead of DCP	0
9	DTBP instead of DCP	64 (8 : 2)^*c*^
10	TBHP_aq_ instead of DCP	63 (7 : 3)^*c*^

^*a*^Reaction conditions: **1a** (0.25 mmol), **2a** (1.25 mmol), Pd(OAc)_2_ (10 mol%), DCP (2.0 equiv.), and Ag_2_CO_3_ (2.0 equiv.) in DMF (1 mL) at 100 °C for 16 h under Ar.

^*b*^Conversion determined by ^1^H NMR analysis.

^*c*^Ratio of mono- and diacylated products.

^*d*^Yield of the isolated product after column chromatography. DCP = dicumyl peroxide; DTBP = di-*tert*-butyl peroxide; TBHP = *tert*-butyl hydroperoxide.

**Scheme 2:**
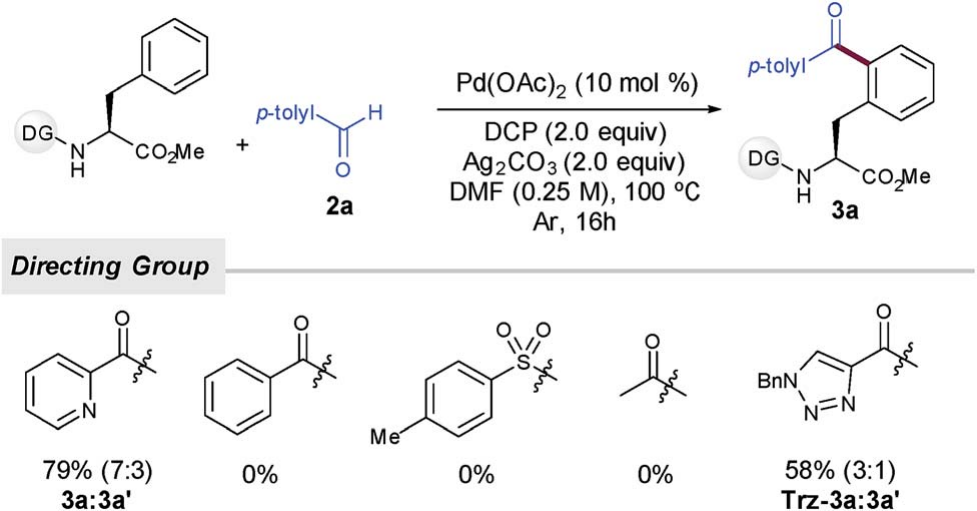
Influence of the DG.

Notably, as commonly observed in related Pd-catalyzed cross-coupling techniques,6a,b,h,i the addition of Ag_2_CO_3_ proved to be highly beneficial for the process to occur (entry 4) and the reaction outcome was rather sensitive to the amount of silver carbonate.^15^ In order to overcome the persistent problem of regioselectivity between the mono- and diacylation reactions, the evaluation of supporting ligands, equivalents of **2a** and other parameters was carefully performed.^14^ Unfortunately, higher selectivity toward the monoacylation product was only achieved at the expense of having much lower overall yields. Likewise, although inorganic persulfates entirely inhibited the reaction (entry 8), other peroxides such as DTBP or commonly used TBHP afforded lower yields of **3a** (entries 9 and 10).^14^ In general terms, reactivity was favored over selectivity and preferential monoacylation (8 : 2) was only achieved when lower yields were obtained (up to 66%, entries 4, 5 and 9), which may underpin the tendency of the monoacylated compound **3a** to undergo a subsequent acylation reaction toward the formation of **3a′**. As initially anticipated, subtle modifications on the DG had a determinant impact on the reaction outcome. Although benzoyl-, tosyl- or acetyl-protected substrates devoid of an additional nitrogen-chelating unit remained unreactive, a related carboxamide bearing a 1,2,3-triazole unit could be also employed as an efficient bidentate DG, albeit in comparatively lower yields (Scheme 2).^14^,^16^

With the optimized conditions in hand, we next investigated the scope of the δ-C(sp^2^)–H acylation protocol with respect to the aldehyde (Table 2). Gratifyingly, a wide variety of electronically diverse aldehydes smoothly underwent the target dehydrogenative coupling in moderate to excellent yields. In general, aromatic aldehydes bearing electron-donating groups such as OMe (**2b** and **2e**), Et_2_N (**2c**) and 2,3-dihydrofuryl (**2d**) provided the corresponding products **3b–e** as mixtures of mono- and diacylated compounds, which were easily separated by column chromatography. In this respect, the highly electron-rich 2,4,6-trimethoxybenzaldehyde (**2i**) afforded selectively the diacylated compound **3i′** in 67% yield; its absolute configuration was verified by X-ray analysis. Conversely, *p*-hydroxybenzaldehyde **2f** provided selectively the corresponding monoacylated product **3f**. Likewise, the lower tendency to oxidation of benzaldehydes **2g–h** bearing electron-withdrawing groups resulted in a high selectivity toward the monoacylation and furnished **3g–h** in good yields. Remarkably, pharmaceutically relevant heterocyclic motifs could be also accommodated and thus *N*-methylindolyl (**2j**) and 2-thienyl carboxaldehyde (**2k**) selectively afforded the corresponding monoacylated products (**3j** and **k**).

**Table 2:** Pd-catalyzed δ-C(sp^2^)–H acylation of Phe derivatives with aldehydes^*a*^ ^,^^*b*^

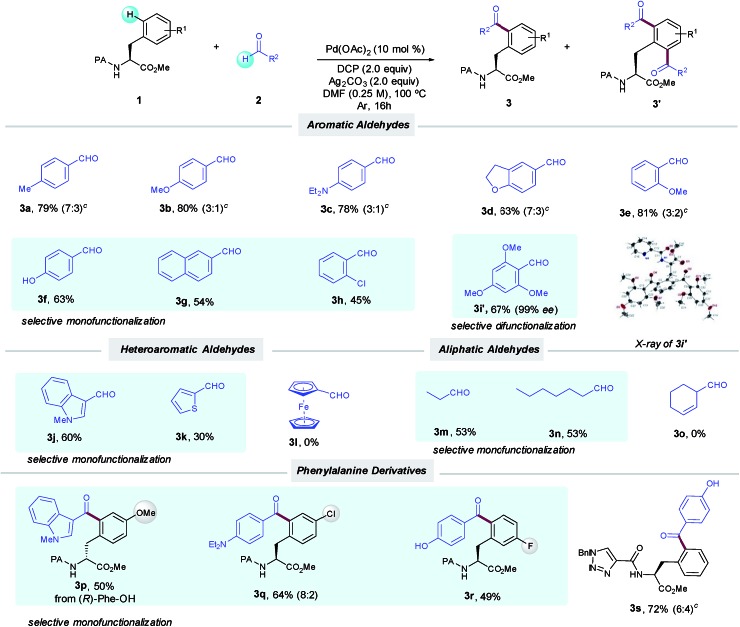

^*a*^As for Table 1, entry 1.

^*b*^Yield of the isolated product after column chromatography, average of at least two independent runs.

^*c*^Ratio of mono- and diacylated products (**3** : **3′**).

Additionally, aliphatic aldehydes could also be employed toward the selective monoacylation of **3m**,**n**, albeit in moderate yields. The latter selectivity could be related to their lower reactivity since full conversion was not achieved. Of remarkable importance are **3c** and **3d**, where high chemoselectivity was achieved toward the preferential activation of the aldehyde motif *versus* the C(sp^3^)–H bonds adjacent to nitrogen and oxygen atoms.5*a* Moreover, Phe substituted derivatives smoothly furnished monoacylated products **3p–r** in moderate to good yields. As verified by HPLC analysis,^14^ no racemization occurred along our oxidative process. It is important to note that the method was found incompatible with the use of aldehydes incorporating alkenes or a ferrocene motif, which could be tentatively attributed to competitive radical functionalization reactions.

Encouraged by these results, we next evaluated our oxidative acylation in the more complex setting of dipeptides, which are known to undergo oxidative fragmentations upon the formation of α-carbon radicals^17^ and hence their selective acylation could be a challenging task to tackle. Notably, dipeptides containing Phe (**5a**,**b**), Leu (**5c–e**) and Pro (**5g**) units selectively underwent the PA-directed acylation with a variety of benzaldehydes on the terminal Phe unit (Table 3). Of particular importance is the tolerance to the oxidizable side-chain hydroxyl group in Thr of dipeptide **5f**, which remained intact along the oxidative process. Likewise, the C–H acylation could be also efficiently directed by a triazole-containing group (**5h**). The preservation of the native chirality of the substrates was underpinned by NMR analysis. The robustness of our method was further demonstrated by the site-selective functionalization of tri- (**5i**,**j**), tetra- (**5k**) and even pentapeptides (**5l′–n**) in moderate to good yields. It is known that the additional amide bonds within oligopeptides can reasonably deactivate the metal catalyst by the formation of *N*,*N*-chelated complexes.6g,^18^ Indeed, by comparison of pentapeptide **5l′** with **5m**,**n** bearing Pro residues, where such an undesired catalyst deactivation is avoided, the site-selective acylation was achieved in excellent yields. The latter underscores the high potential for Pro residues as key elements at the late-stage functionalization in peptide settings. Noteworthy, the acylation exclusively occurred at the N-terminal Phe unit and other residues bearing oxidizable aliphatic chains with reactive secondary C–H bonds such as Leu, Ile, Ala, and Val remained intact.^19^ Collectively, the small library of oligopeptides rapidly assembled illustrates the vast potential of our catalytic manifold to introduce ketone motifs in a late-stage fashion to furnish densely decorated peptides. The reaction represents an innovative, yet challenging dehydrogenative radical technique, which offers previously unrecognized opportunities in the field of peptide chemistry. In this respect, although good to excellent yields could be obtained with certain peptides (up to 78% yield), it is important to note that the sometimes obtained low to moderate yields were due to incomplete conversion of the starting material; the reactions were very clean and the only side-product was derived from the oxidation of the aldehyde to the corresponding carboxylic acid, which was easily removed upon the reaction work-up.

**Table 3:** Late-stage Pd-catalyzed δ-C(sp^2^)–H acylation of Phe-containing peptides with aldehydes^*a*^ ^,^^*b*^

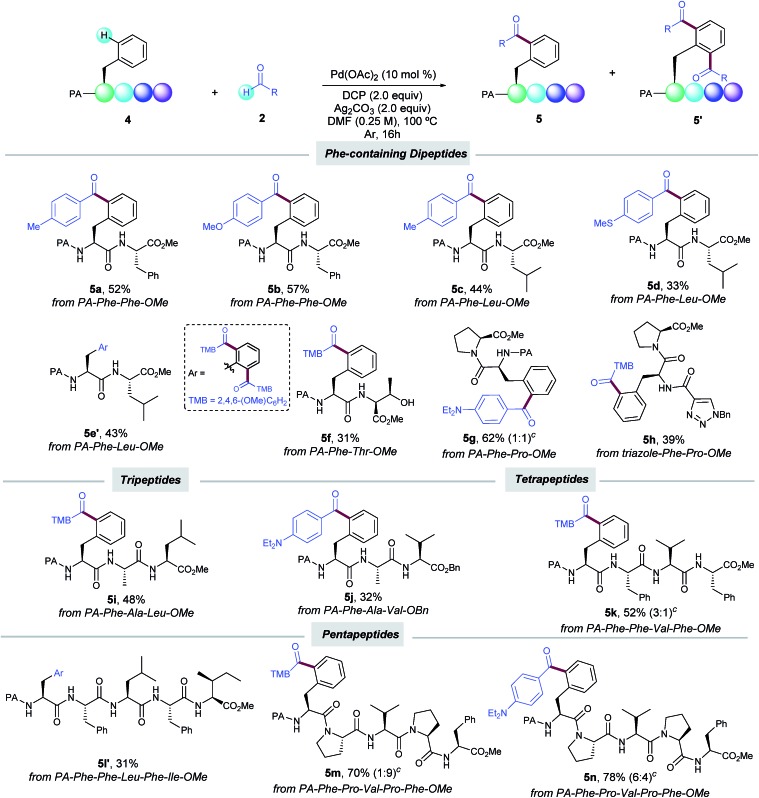

^*a*^As for Table 1, entry 1.

^*b*^Yield of the isolated product after column chromatography, average of at least two independent runs.

^*c*^Ratio of mono- and diacylated products (**5** : **5′**).

**Scheme 3:**
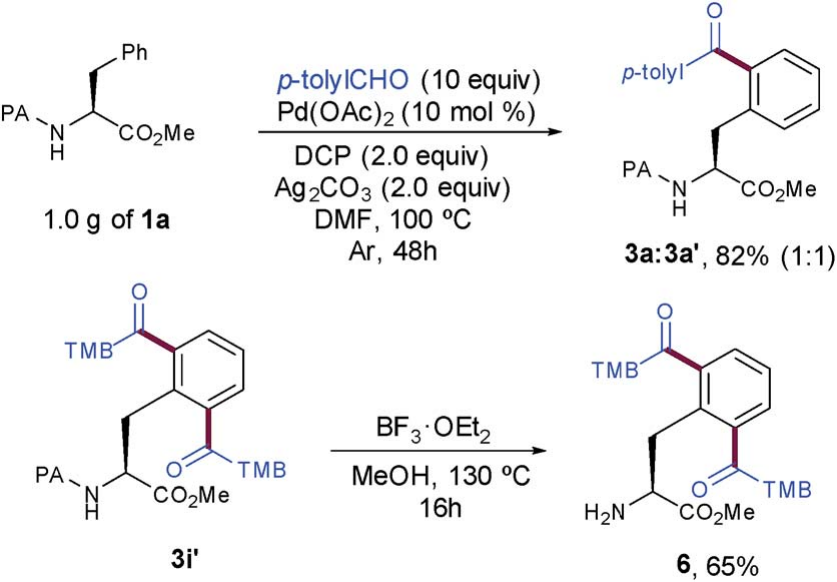
Cleavage of the DG and gram scale synthesis.

The synthetic utility and robustness of our site-selective functionalization manifold were highlighted by scaling up the acylation reaction to the gram level and **3a** was obtained in a remarkable 82% yield. However, the extended reaction time to reach completion resulted in a lower selectivity than that of the experiment at 0.25 mmol (1 : 1 *vs.* 7 : 3). The facile removal of the PA group6a,11d showcased its practicality to ultimately deliver highly functionalized peptide molecules bearing a synthetically versatile free-amino group (Scheme 3).

In order to expand the potential of Phe as a fully diversifiable unit through the formation of a 6-membered palladacycle, we next studied the PA-directed Pd-catalyzed C–halogen bond-forming processes upon a related Pd(ii)/Pd(iv) regime. Yu and co-workers have developed iodination8*h* reactions with a combination of PhI(OAc)_2_ and I_2_ using triflamide as the DG. Owing to the more practical features of non-halogenated and easily removable PA, we successfully accomplished a variety of dihalogenation reactions of Phe derivatives;^14^,^20^ the corresponding dibromination with *N*-bromosuccinimide was efficiently applied to the assembly of a small family of substituted Phe derivatives **7a–e** in excellent yields (Table 4). The structure of **7c** was unambiguously assigned by X-ray analysis verifying that the bromination proceeded with enantiospecificity. Importantly, the use of related halosuccinimides afforded iodinated (**7f**) and chlorinated (**7g**) products in good to excellent yields. The latter illustrated that PA can be an efficient auxiliary for performing not only C–H acylations but also relevant C–H halogenation reactions in Phe derivatives.

**Table 4:** C(sp^2^)–H halogenation of Phe derivatives^*a*^ ^,^^*b*^

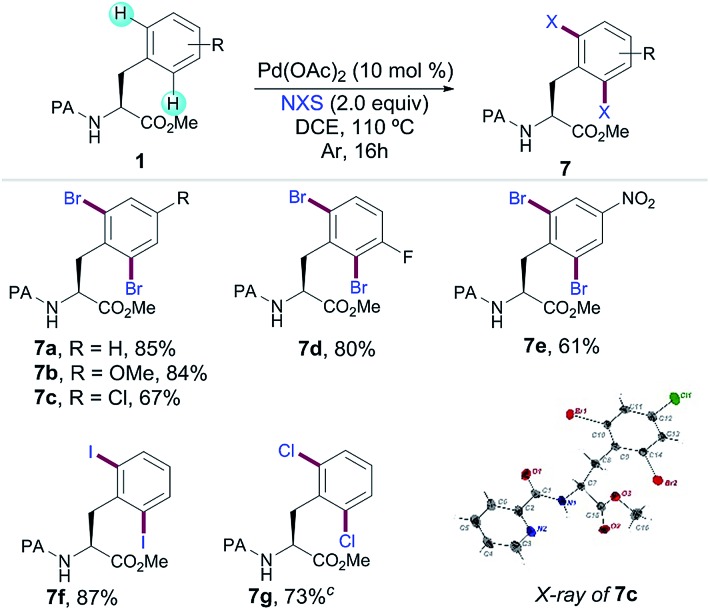

^*a*^Reaction conditions: **1** (0.25 mmol), Pd(OAc)_2_ (10 mol%), NXS (2.0 equiv.), DCE (2 mL), Ar, 110 °C, 16 h.

^*b*^Yield of the isolated product after column chromatography, average of at least two independent runs.

^*c*^AgF (2.0 equiv.) was added.

**Scheme 4:**
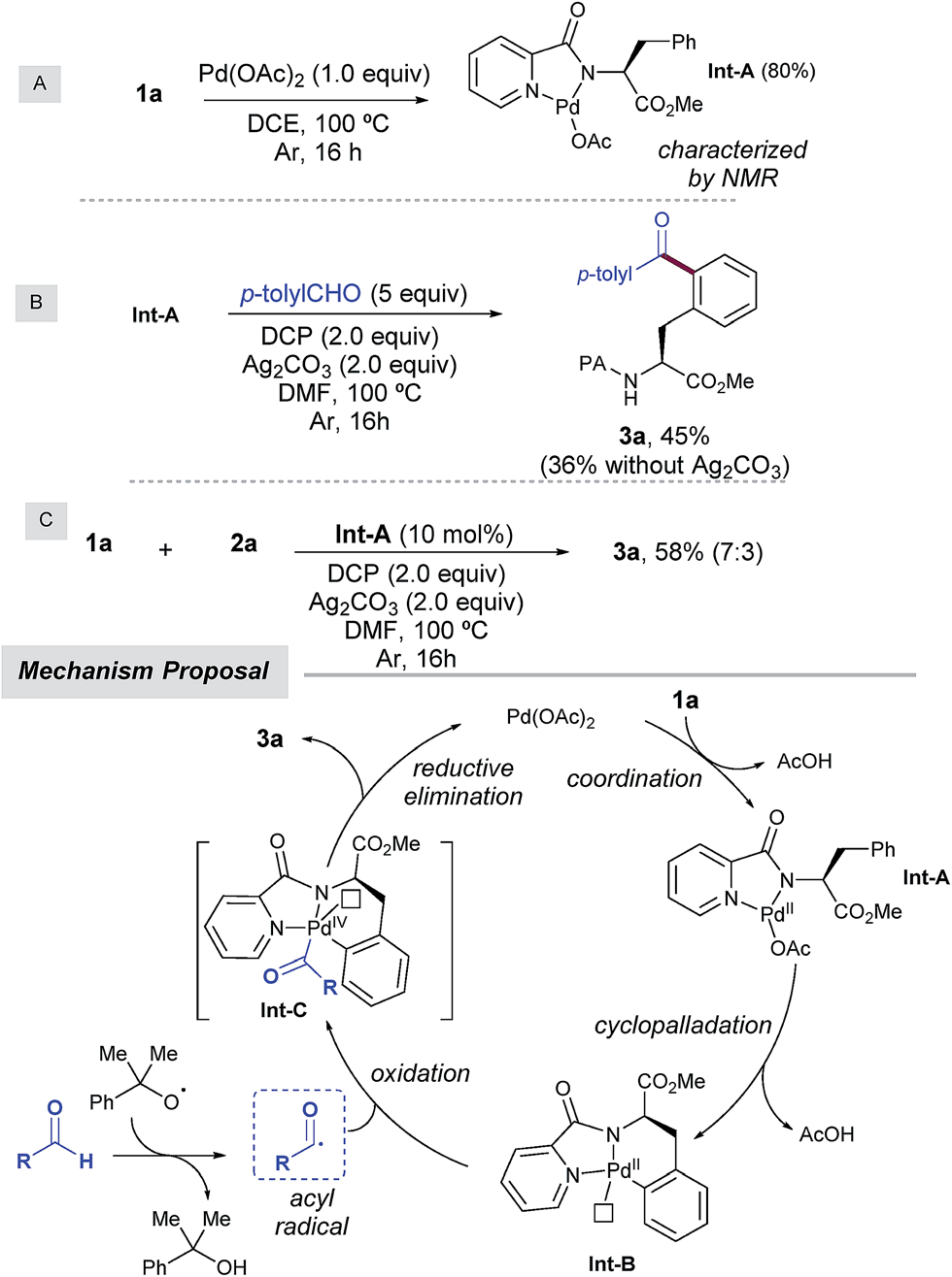
Control experiments and the proposed mechanism.

To shed light on the reaction mechanism, we carried out several control experiments with **1a** as a simple model system (Scheme 4). On the one hand, we found that the acylation of **1a** with aldehyde **2a** was entirely inhibited in the presence of radical traps such as TEMPO, BHT, and diphenylethylene,^14^ which indicated that a radical scenario may be operative. On the other hand, in order to support the intermediacy of a palladacycle intermediate the following experiments were performed. First, a stoichiometric reaction of **1a** with Pd(OAc)_2_ provided **IntA** in 80% yield, which was characterized by NMR spectroscopy. Second, when **IntA** was subjected to the optimized acylation conditions, **3a** was obtained in 45% yield as the monoacetylated compound, and in lower yield in the absence of silver carbonate.6a,^21^ Likewise, **IntA** efficiently catalyzed the formation of **3a** from **1a** in 58% isolated yield, which underpinned its key role as a viable precatalyst.

On the basis of the above results and previous literature reports,^12^ a plausible monomeric reaction mechanism is proposed in Scheme 4. Complexation of **1a** with Pd(OAc)_2_ would initially afford Pd(ii) complex **IntA**,6*c* which would next undergo a directed *ortho*-selective cyclometallation to provide the six-membered palladacycle **IntB**.9a,b The latter would next react with the acyl radical,^12^ which was *in situ* generated upon hydrogen atom abstraction by cumyl peroxyl radical species to provide transient Pd(iii) species12e,f which would be subsequently oxidized to deliver **IntC**.^22^ This species has been proposed to exist as either Pd(iv)^23^ or dimeric Pd(iii)^24^ intermediates and would furnish the acylated product **3a** through C–C bond forming reductive elimination, thereby regenerating the active Pd(ii) catalyst. Importantly, a competitive intramolecular C–N bond forming reductive elimination8*f* was never observed. At this stage, the involvement of polynuclear Pd complexes6*a* or heterodimeric Pd–Ag^21^ intermediates cannot be ruled out within our catalytic cycle.

## Conclusions

In summary, we have developed a practical protocol for the assembly of non-proteogenic acylated Phe-containing oligopeptides *via* a novel Pd-catalyzed δ-C(sp^2^)–H functionalization reaction with abundant and readily available aldehydes. From a fundamental point of view, this transformation represents a robust, yet innovative means for the radical functionalization of a wide range of Phe-containing compounds, thus expanding the landscape of peptide synthesis to provide heavily substituted peptide analogues containing aryl, heteroaryl and even aliphatic ketone residues. The important features of our strategy are the widespread availability of aldehydes, the broad functional group tolerance, the retention of the chiral integrity of the existing stereocenters in peptide settings, the site-selectivity toward the functionalization of the Phe unit assisted by the N-terminal PA group, and the facile removal of the required PA group. Moreover, the process can be extended to the use of medicinally relevant 1,2,3-triazoles as alternative bidentante DGs. Therefore, we anticipate that our Pd-catalyzed oxidative acylation method could become a powerful platform technology of tremendous importance in drug discovery and protein engineering.

## Conflicts of interest

There are no conflicts to declare.

## Supplementary Material

Supplementary information

Crystal structure data
