# Supplementary material for: Pd-catalyzed site-selective C(sp2)–H radical acylation of phenylalanine containing peptides with aldehydes
Source: Chem Sci. 2019 Aug 7;10(38):8872–9. doi: 10.1039/c9sc03425k (PMC6853082; doi:10.1039/c9sc03425k)
Supplement: Supplementary file 1 [file SC-010-C9SC03425K-s001.pdf]

Electronic Supplementary Material (ESI) for Chemical Science.
This journal is © The Royal Society of Chemistry 2019

Supporting Information

Pd-Catalyzed Site-Selective C(sp^2)–H Radical Acylation of Phenylalanine Containing Peptides with Aldehydes

*Marcos San Segundo and Arkaitz Correa**

University of the Basque Country (UPV/EHU), Department of Organic Chemistry I

Joxe Mari Korta R&D Center, Avda. Tolosa 72, 20018

Donostia-San Sebastián (Spain) .e-mail: arkaitz.correa@ehu.eus

1.- General Considerations **S2**

2.- Optimization Details **S3**

3.- Preparation of Starting Materials **S8**

4.- Pd-Catalyzed δ-C(sp^2)-H Acylation of Phe Derivatives **S15**

5.- Deprotection of 3i' **S35**

6.- Pd-catalyzed C(sp^2)–H Dihalogenation of Phe Derivatives **S36**

7.- X-Ray Crystallography of 3i' **S40**

8.- X-Ray Crystallography of 7c **S42**

9.- Mechanistic Experiments **S44**

10.- Determination of *ee* by HPLC Analysis **S46**

11.- ^{1}H NMR and ^{13}C NMR Spectra **S47**

1.-General Considerations

Reagents. Palladium acetate was purchased from Fluorochem. *N,N*-Dimethylformamide (DMF) (anhydrous, 99.8%) and dicumyl peroxide (DCP) (98%) were purchased from Sigma-Aldrich. Ag_2CO_3 (99.5%, metal basis) was purchased from Alfa Aesar. All the aldehydes were commercially available and were used without further purification.

Analytical Methods. ^{1}H NMR and ^{13}C NMR spectra as well as IR, HRMS and melting points (where applicable) are included for all new compounds. ^{1}H and ^{13}C NMR spectra were recorded on a Bruker 400 at 20 ºC. All ^{1}H NMR spectra are reported in parts per million (ppm) downfield of TMS and were measured relative to the signals for $CHCl_3$ (7.26 ppm), unless otherwise indicated. All ^{13}C NMR spectra were reported in ppm relative to residual $CHCl_3$ (77 ppm), unless otherwise indicated, and were obtained with ^{1}H decoupling. Coupling constants, *J*, are reported in hertz. Melting points were measured using open glass capillaries in a Büchi SMP-20 apparatus. High resolution mass spectra (HRMS) were performed by Central Service of Analysis de Álava-SGIker of UPV/EHU and were acquired on a LC/Q-TOF mass spectrometer equipped with an electrospray source ESI Agilent Jet Stream. Infrared spectra were recorded on a Bruker Alpha P. Flash chromatography was performed with EM Science silica gel 60 (230-400 mesh). HPLC chiral analyses were performed in a Waters Delta Prep 4000 equipment with a Waters 2487 dual detector. X-Ray analysis to determine the absolute structure using likelihood methods (Hooft, Straver & Spek, 2008) was performed using PLATON (Spek, 2010). The results indicated that the absolute structure had been correctly assigned. The method calculated that the probability that the structure is inverted is smaller than 10^{-99}. The absolute structure parameter y (Hooft, Straver & Spek, 2008) was calculated using PLATON (Spek, 2010). The resulting value was y=-0.005(17), which together with Flack parameter value, indicate that the absolute structure has probably been determined correctly. The yields reported in the manuscript correspond to isolated yields and represent an average of at least two independent runs.

2.-Optimization Details

General Procedure: Coupling of 1a with *p*-tolualdehyde (2a)

A reaction tube containing a stirring bar was charged with **1a** (0.25 mmol, 71 mg), oxidant (0.50 mmol) (if solid), and metal source (10 mol %). The reaction tube was then evacuated and back-filled with dry argon (this sequence was repeated up to three times). Then, oxidant (if liquid), **2a** (1.25 mL) and solvent (1.00 mL) were added by syringe under argon atmosphere. The reaction tube was next warmed up to the corresponding temperature and stirred for 16 hours. The mixture was allowed to warm to room temperature, concentrated under reduced pressure and the corresponding product was purified by flash chromatography (hexanes/EtOAc 7/3). The purity of the corresponding product **3a** was verified by ^{1}H NMR.

Table S1. Screening of oxidants[a]

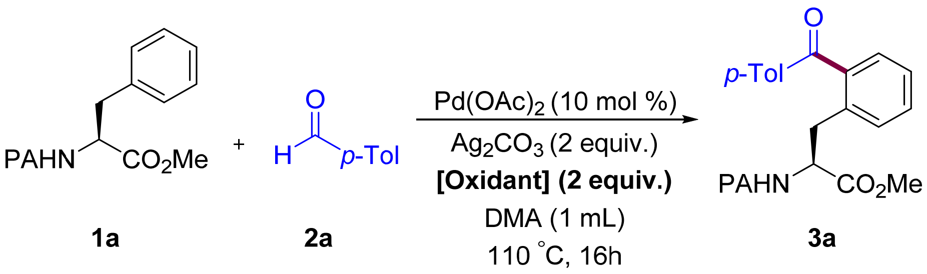

1a **2a** **3a**

Entry	Oxidant	Yield (%)[b,c]
1	$(NH_4)_2S_2O_8$	0
2	$K_2S_2O_8$	0
3	Oxone	0
4	$TBHP_{aq}$	63 (72:28)
5	$TBHP_{dec}$	72 (69:31)
6	DTBP	64 (8:2)
7	DCP	78 (6:4)[d]
8	H_2O_2	0
9	O_2 (1 atm)	0

[a]Reaction conditions: **1a** (0.25 mmol), **2a** (1.5 mmol), $Pd(OAc)_2$ (10 mol %), Ag_2CO_3 (2 equiv.), dry DMA (1 mL), Ar, 16h at 110 °C. [b] Conversion determined by ^{1}H NMR analysis. [c]Ratio of mono- and diacylated product **3a**:**3a'**. [d]Yield of isolated product after column chromatography.

Table S2. Screening of Metals[a]

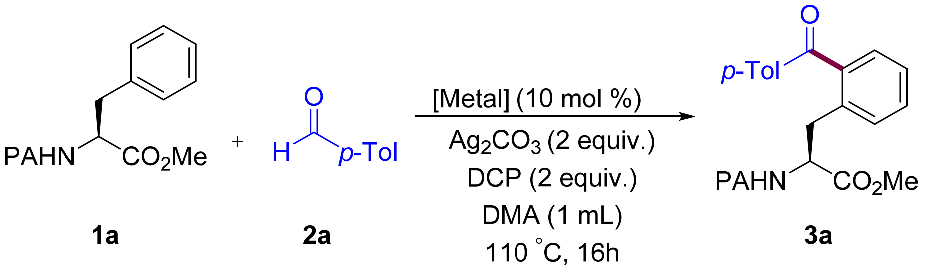

Entry	Metal	Yield (%)[b]
1	$Pd(OAc)_2$	79 (7:3)
2	$Mn(OAc)_2$	0
3	$Ni(acac)_2$	0
4	$Co(acac)_2$	0
5	$Ru_3(CO)_9$	0
6	$RuCl_3$	0
7	[RuCl(*p*-cymene)]$_2$	0

[a]Reaction conditions: **1a** (0.25 mmol), **2a** (1.5 mmol), [M] (10 mol%), Ag_2CO_3 (2 equiv.), dry DMA (1 mL), Ar, 16h, 110 °C. Yield of isolated product after column chromatography. [b]Ratio of mono- and diacylated products.

Table S3. Screening of Ligands[a]

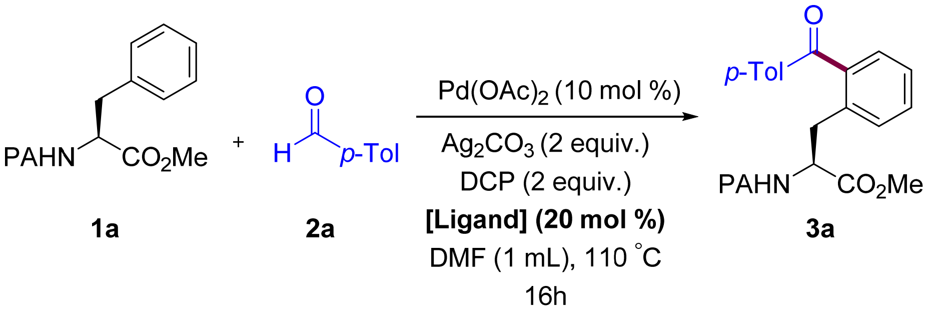

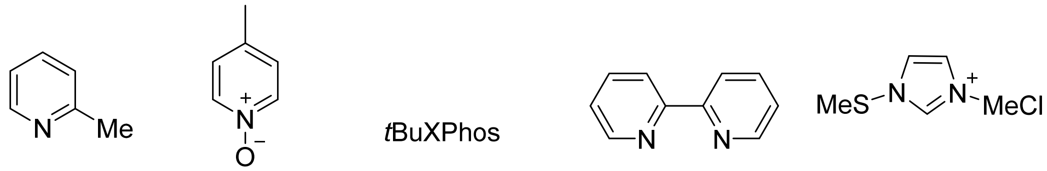

L1	L2	L3	L4	L5
64% (78:22)[b]	48% (9:1)[b]	73% (64:36)[b]	60% (82:18)[b]	59% (77:23)[b]

[a]Reaction conditions: **1a** (0.25 mmol), **2a** (1.0 mmol), $Pd(OAc)_2$ (10 mol%), Ag_2CO_3 (2 equiv.), dry DMF (1 mL), Ar, 16h, 110 °C. Conversion determined by ^1H NMR analysis. [b]Ratio of mono- and diacylated product.

Table S4. Screening of temperature[a]

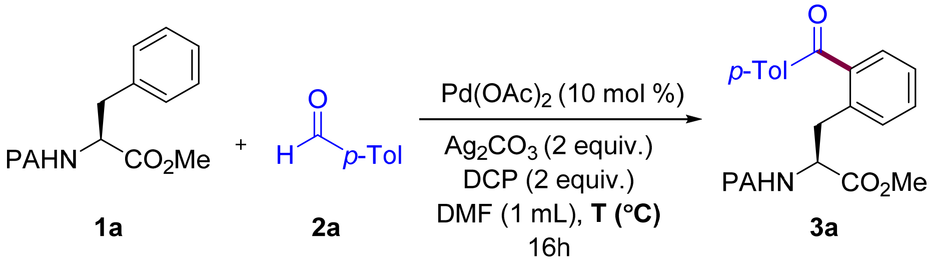

Entry	T (°C)	Yield (%)[b,c]
1	90	54 (85:15)
2	100	79 (7:3)[d]
3	110	66 (83:17)
4	120	63 (78:22)
5	130	61 (75:25)

[a]Reaction conditions: **1a** (0.25 mmol), **2a** (1.25 mmol), $Pd(OAc)_2$ (10 mo l%), Ag_2CO_3 (2 equiv.), dry DMF (1 mL), N_2, 16h, 110 °C. [b]Conversion determined by ^{1}H NMR analysis. [c]Ratio of mono- and diacylated product. [d]Yield of isolated product after column chromatography.

Table S5. Screening of aldehyde equivalents[a]

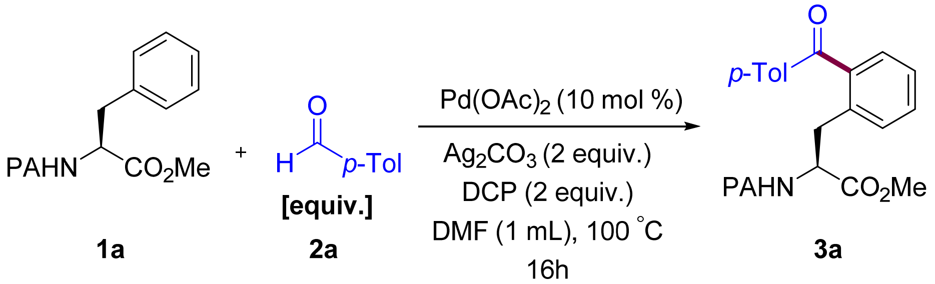

Entry	Equiv. of 2a	Yield (%)[b,c]
1	4	73 (75:25)
**2**	**5**	**79 (7:3)[d]**
3	6	88 (6:4)

[a]Reaction conditions: **1a** (0.25 mmol), **2a** (1.0-1.5 mmol), $Pd(OAc)_2$ (10 mol %), Ag_2CO_3 (2 equiv.), dry DMF (1 mL), Ar, 16h, 100 °C. [b]Conversion determined by ^{1}H NMR analysis. [c]Ratio of mono- and diacylated product. [d]Yield of isolated product after column chromatography.

Table S6. Solvent screening[a]

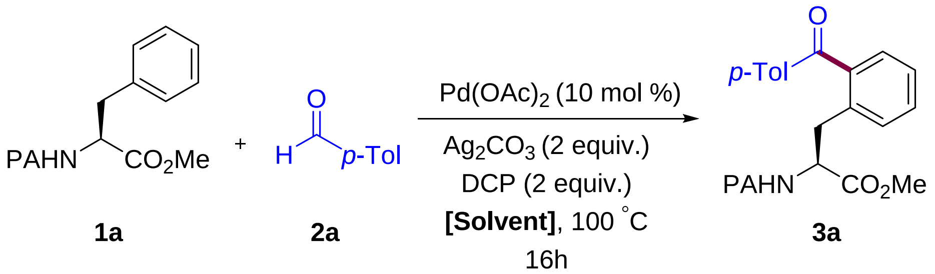

Entry	Solvent	Yield (%)[b,c]
1	DMF	79 (7:3)[d]
2	DCE	84 (38:62)
3	Toluene	79 (54:46)
4	PhCl	75 (48:52)
5	Dioxane	75 (55:45)

[a]Reaction conditions: **1a** (0.25 mmol), **2a** (1.5 mmol), $Pd(OAc)_2$ (10 mol %), Ag_2CO_3 (2 equiv.), dry Solvent (1 mL), Ar, 16h, 110 °C. [b]Conversion determined by ^1H NMR analysis. [c]Ratio of mono- and diacylated product. [d]Yield of isolated product after column chromatography.

Table S7. Screening of directing groups[a]

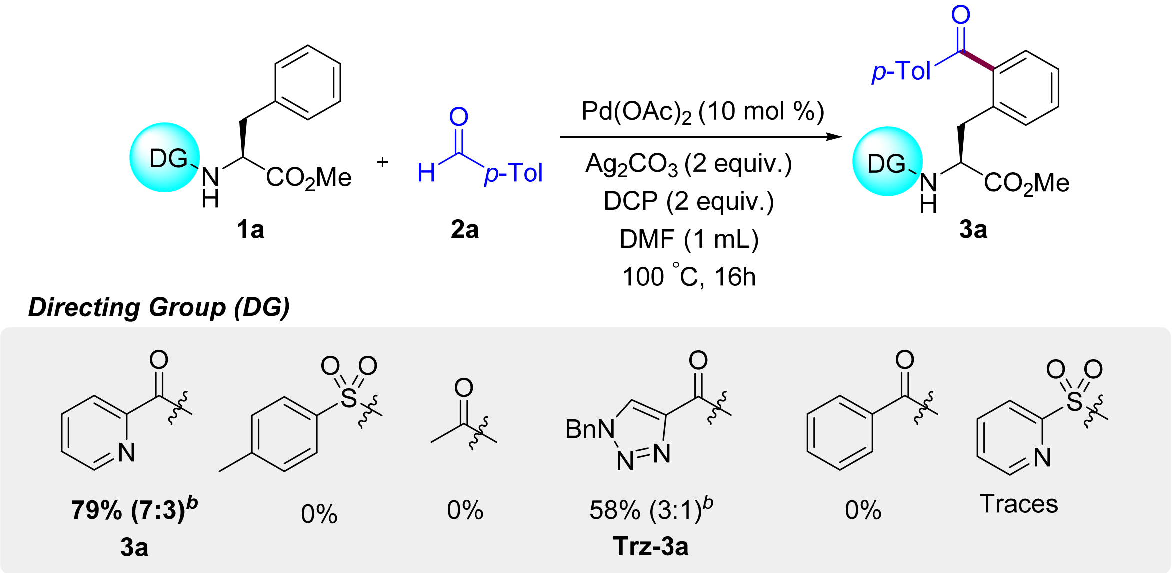

[a]Reaction conditions: **1a** (0.25 mmol), **2a** (1.25 mmol), $Pd(OAc)_2$ (10 mol %), Ag_2CO_3 (2 equiv.), dry DMF (1 mL), Ar 16h, 100 °C. Yield of isolated product after column chromatography. [b]Ratio of mono- and diacylated products.

Table S8. Control experiments[a]

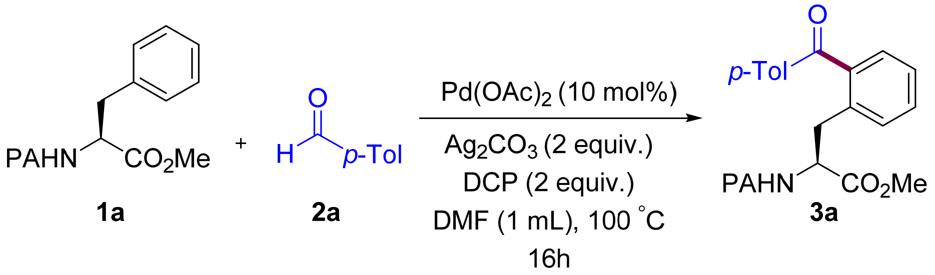

entry	$Pd(OAc)_2$	DCP	Ag_2CO_3	Yield of 3a (%)[b,c]
1	✓	✓	✓ (2.0 equiv)	79 (7:3)[d]
2	✗	✓	✗	0
3	✓	✗	✓	0
4	✓	✓	✗	56 (82:18)
5	✓	✓	✓ (0.5 equiv)	65 (7:3)
6	✓	✓	✓ (1.0 equiv)	71 (7:3)

[a]Reaction conditions: **1a** (0.25 mmol), **2a** (1.0 mmol), $Pd(OAc)_2$ (10 mol %), Ag_2CO_3 (2 equiv.), dry DMF (1 mL), Ar, 16h, 110 °C. [b]Conversion determined by ^1H NMR analysis. [c]Ratio of mono- and diacylated product. [d]Yield of isolated product after column chromatography.

3.-Preparation of the Starting Materials

***α*-Amino carbonyl compound 1**

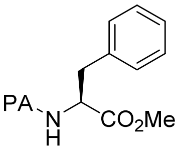

1a[a]

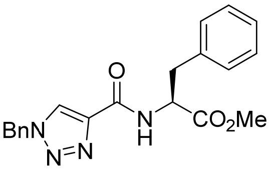

1b[a]

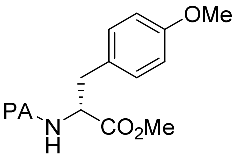

1c[a]

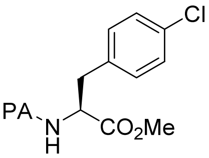

1d[a]

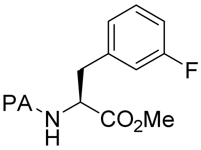

1e[b]

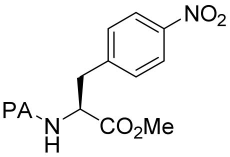

1f[b]

Peptide derivatives 4

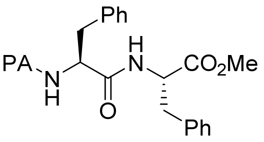

PA-Phe-Phe-OMe
4a[b]

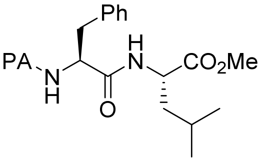

PA-Phe-Leu-OMe
4b[a]

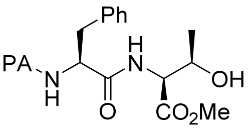

PA-Phe-Thr-OMe
4c[b]

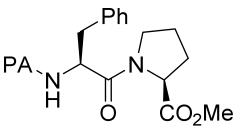

PA-Phe-Pro-OMe
4d[b]

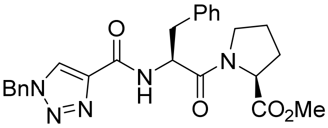

Trz-Phe-Pro-OMe
4e[b]

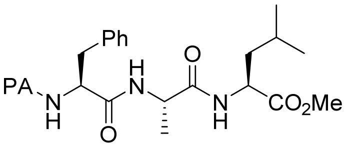

PA-Phe-Ala-Leu-OMe
4f[b]

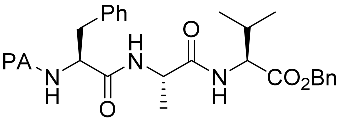

PA-Phe-Ala-Val-OBn
4g[b]

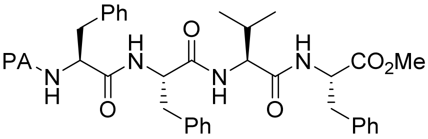

PA-Phe-Phe-Val-Phe-OMe
4h[b]

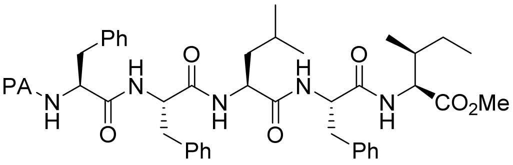

PA-Phe-Phe-Leu-Phe-ILe-OMe
4i[b]

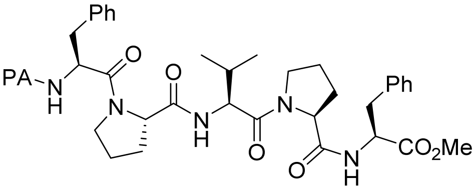

PA-Phe-Pro-Val-Pro-Phe-OMe
4j[b]

[a] Prepared following literature procedures. [b] Synthesis reported herein.

3.1.-Preparation of Picolinamide-protected Phe-containing Derivatives 1

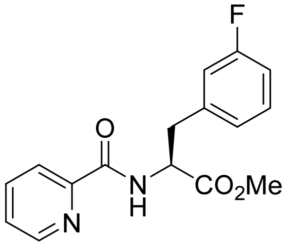

(**1e**)

Methyl (*S*)-3-(3-fluorophenyl)-2-(picolinamido)propanoate (1e). A reaction flask containing a stirring bar was charged with commercially available methyl (2*S*)-2-amino-3-(3-fluorophenyl)propanoate hydrochloride (4.80 mmol, 1.13 g), EDC·HCl (5.76 mmol, 768 mg), *N*-hydroxybenzotriazole (5.76 mmol, 1.09 g), triethylamine (5.76 mmol, 0.77 mL) in DCM (40 mL) at 0 °C. The resulting solution was stirred at room temperature overnight. The mixture was then washed with water, dried over $MgSO_4$ and concentrated under reduced pressure. Finally, the resulting residue was purified by flash chromatography to obtain 1.30 g (90% yield) of **1e** as a colorless oil. ^{1}H NMR (400 MHz, $CDCl_3$) δ 8.58 (d, *J* = 4.8 Hz, 1H), 8.53 (d, *J* = 8.3 Hz, 1H), 8.18 (d, *J* = 7.8 Hz, 1H), 7.86 (td, *J* = 7.7, 1.7 Hz, 1H), 7.46 (ddd, *J* = 7.6, 4.8, 1.2 Hz, 1H), 7.31 – 7.22 (m, 1H), 6.99 (d, *J* = 7.7 Hz, 1H), 6.96 – 6.89 (m, 2H), 5.19 – 5.00 (m, 1H), 3.77 (s, 3H), 3.35 – 3.19 (m, 2H). ^{13}C NMR (101 MHz, $CDCl_3$) δ 171.6, 164.1, 161.7, 149.3, 148.4, 138.7 (d, *J* = 8.1 Hz), 137.4, 130.1 (d, *J* = 8.1 Hz), 126.6, 125.1, 125.1, 122.4, 116.3 (d, *J* = 21.2 Hz), 114.2 (d, J = 21.2), 53.4, 52.6, 38.1. IR (cm^{-1}): 3029, 1743, 1654, 1531, 1178. HRMS *calcd.* for ($C_{16}H_{15}FN_2O_3$): 302.1067, *found* 302.1070

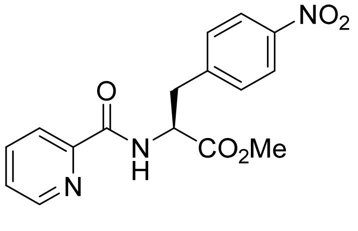

(**1f**)

Methyl (*S*)-3-(4-nitrophenyl)-2-(picolinamido)propanoate (1f). Following the procedure above, commercially available methyl (2*S*)-2-amino-3-(4-nitrophenyl)propanoate hydrochloride (5.75 mmol, 1.5 g) provided 1.30 g (90% yield) of **1f** as a white solid. Mp 69-70 ºC. ^{1}H NMR (400 MHz, $CDCl_3$) δ 8.60 – 8.46 (m, 2H), 8.22 – 8.03 (m, 3H), 7.84 (td, *J* = 7.7, 1.7 Hz, 1H), 7.51 – 7.31 (m, 3H), 5.24 – 4.97 (m, 1H), 3.75 (s, 3H), 3.55 – 3.17 (m, 2H). ^{13}C NMR (101 MHz, $CDCl_3$) δ 171.1, 164.0, 148.9, 148.4, 147.1, 144.0, 137.4, 130.2, 126.6, 123.7, 122.3, 53.0, 52.7, 38.1. IR (cm^{-1}): 3129, 1755, 1643. HRMS *calcd.* for ($C_{16}H_{15}N_3O_5$): 329.1012, *found* 329.1015.

3.2.-Preparation of picolinamide-protected peptide derivatives 4

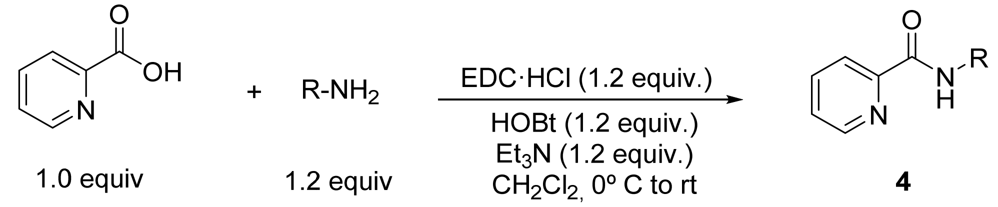

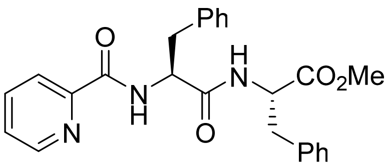

(**4a**)

PA-Phe-Phe-OMe

Methyl picolinoyl-*L*-phenylalanyl-*L*-phenylalaninate (4a). Following the procedure above, H-Phe-Phe-OMe (3.07 mmol, 1.0 g) provided 1.10 g (83% yield) of **4a** as a white solid. Mp 124-125°C. ^{1}H NMR (400 MHz, $CDCl_3$) δ 8.59 (d, *J* = 4.7 Hz, 1H), 8.51 (d, *J* = 8.4 Hz, 1H), 8.16 (d, *J* = 7.8 Hz, 1H), 7.88 (td, *J* = 7.7, 1.6 Hz, 1H), 7.48 (dd, *J* = 6.6, 4.7 Hz, 1H), 7.30-7.29 (m, 4H), 7.27 – 7.20 (m, 1H), 7.13 – 7.04 (m, 3H), 6.95 (dd, *J* = 7.5, 2.0 Hz, 2H), 6.49 (d, *J* = 8.4 Hz, 1H), 4.96 – 4.77 (m, 2H), 3.70 (s, 3H), 3.22 (d, *J* = 8.0 Hz, 2H), 3.14 – 2.97 (m, 2H). ^{13}C NMR (101 MHz, $CDCl_3$) δ 171.5, 170.3, 164.5, 149.2, 148.4, 137.4, 136.7, 135.7, 129.5, 129.3, 128.7, 128.5, 127.1, 126.6, 122.5, 54.5, 53.4, 52.4, 38.0, 37.8. IR (cm^{-1}): 3029, 1743, 1656, 1517, 1434, 1213, 1178. HRMS *calcd.* for ($C_{25}H_{25}N_3O_4$): 431.1845, *found* 431.1856.

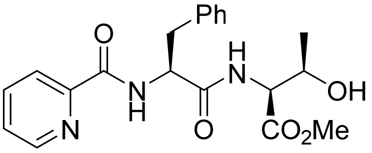

(**4c**)

PA-Phe-Thr-OMe

Methyl picolinoyl-*L*-phenylalanyl-*L*-threoninate (4c). Following the procedure above, H-Phe-Thr-OMe (3.57 mmol, 1.0 g) provided 1.00 g (73% yield) of **4c** as a white solid. Mp 141-142 °C. ^{1}H NMR (400 MHz, $CDCl_3$) δ 8.64 (d, *J* = 8.2 Hz, 1H), 8.57 (d, *J* = 4.2 Hz, 1H), 8.15 (d, *J* = 7.8 Hz, 1H), 7.85 (td, *J* = 7.7, 1.7 Hz, 1H), 7.50 – 7.42 (m, 1H), 7.36 – 7.18 (m, 6H), 6.96 (d, *J* = 8.8 Hz, 1H), 4.95 (q, *J* = 7.1 Hz, 1H), 4.61 (dd, *J* = 8.9, 2.9 Hz, 1H), 4.37 – 4.25 (m, 1H), 3.75 (s, 3H), 3.35 – 3.19 (m, 2H), 2.79 (brs, 1H), 1.16 (d, *J* = 6.4 Hz, 3H). ^{13}C NMR (101 MHz, $CDCl_3$) δ 171.3, 171.1, 164.7, 149.1, 148.5, 137.5, 136.5, 129.5, 128.7, 127.1, 126.7, 122.5, 68.3, 57.7, 55.1, 52.7, 38.2, 20.0. IR(cm^{-1}): 1743, 1656, 1520, 1434, 1213. HRMS *calcd.* for ($C_{20}H_{23}N_3O_5$): 385,1638, *found* 385.1653.

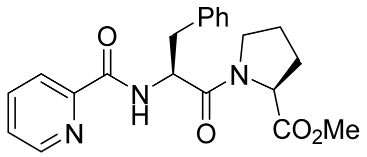

(**4d**)

PA-Phe-Pro-OMe

Methyl [(*S*)-2-phenyl-1-(picolinamido)ethyl]-*L*-prolinate (4d). Following the procedure above, H-Phe-Pro-OMe (5.43 mmol, 1.50 g) provided 1.00 g (48% yield) of **4d** as a colorless oil. ^{1}H NMR (400 MHz, $CDCl_3$) δ 8.74 (d, *J* = 8.7 Hz, 1H), 8.57 (d, *J* = 4.0 Hz, 1H), 8.12 (d, *J* = 7.8 Hz, 1H), 7.83 (td, *J* = 7.7, 1.7 Hz, 1H), 7.47 – 7.40 (m, 1H), 7.38 – 7.23 (m, 5H), 5.17 (q, *J* = 15.0, 7.5 Hz, 1H), 4.57 – 4.47 (m, 1H), 3.78 (s, 3H), 3.33 – 3.07 (m, 4H), 2.23 – 2.13 (m, 1H), 2.01 – 1.88 (m, 3H). ^{13}C NMR (101 MHz, $CDCl_3$) δ 172.4, 170.2, 163.9, 149.5, 148.5, 137.3, 136.5, 129.8, 128.5, 127.0, 126.4, 122.2, 59.2, 52.4, 52.3, 47.1, 39.3, 29.2, 25.1. IR (cm^{-1}): 2952, 1743, 1673, 1646, 1512, 1430, 1196, 1174. HRMS *calcd.* for ($C_{21}H_{23}N_3O_4$): 381.1689, *found* 381.1702.

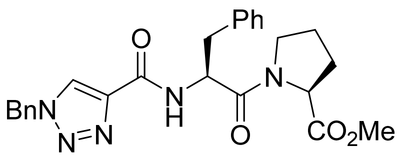

(**4e**)

Trz-Phe-Pro-OMe

Methyl (1-benzyl-1*H*-1,2,3-triazole-4-carbonyl)-*L*-phenylalanyl-*L*-prolinate (4e). Following the procedure above, H-Phe-Pro-OMe (3.62 mmol, 1.00 g) provided 1.10 g (66% yield) of **4e** as a colorless oil. ^{1}H NMR (500 MHz, DMSO-d_6 at 80°C) δ 8.51 (s, 1H), 8.03 (d, *J* = 8.1 Hz, 1H), 7.45 – 7.06 (m, 10H), 5.64 (s, 2H), 5.00 (q, *J* = 7.2 Hz, 1H), 4.40 (dd, *J* = 8.6, 4.8 Hz, 1H), 3.81 – 3.70 (m, 1H), 3.66 (s, 3H), 3.51 – 3.38 (m, 1H), 3.18 – 2.99 (m, 2H), 2.21 – 2.03 (m, 1H), 1.97 – 1.70 (m, 3H). ^{13}C NMR (126 MHz, DMSO-d_6 at 80°C) δ 171.5, 168.9, 158.6, 141.9, 136.6, 135.0, 128.9, 128.8, 128.3, 127.8, 127.6, 127.6, 127.5, 125.9, 58.4, 52.9, 51.2, 51.1, 46.1, 36.7, 28.1, 24.1. IR (cm^{-1}): 3393, 2952, 1741, 1639, 1567, 1496, 1433, 1195, 1173, 1044, 718, 699. HRMS *calcd.* for ($C_{25}H_{27}N_5O_4$): 461.2063, *found* 461.2076.

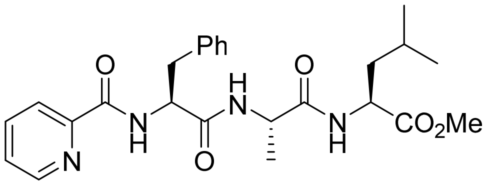

(**4f**)

PA-Phe-Ala-Leu-OMe

Methyl picolinoyl-L-phenylalanyl-L-alanyl-L-leucinate (4f). Following the procedure above, H-Phe-Ala-Leu-OMe (1.38 mmol, 500 mg) provided 400 mg (62% yield) of **4f** as a colorless oil. ^{1}H NMR (400 MHz, $CDCl_3$) δ 8.64 – 8.47 (m, 2H), 8.15 (dt, *J* = 7.8, 1.1 Hz, 1H), 7.86 (td, *J* = 7.7, 1.7 Hz, 1H), 7.47 (ddd, J = 7.6, 4.8, 1.3 Hz,

1H), 7.36 – 7.20 (m, 6H), 6.63 (dd, *J* = 13.6, 7.8 Hz, 2H), 4.86 (q, *J* = 7.0 Hz, 1H), 4.64 – 4.42 (m, 2H), 3.75 (s, 3H), 3.25 (d, *J* = 6.7 Hz, 2H), 1.73 – 1.53 (m, 4H), 1.34 (d, *J* = 7.0 Hz, 3H), 0.95 (dd, *J* = 6.0, 3.5 Hz, 6H). ^{13}C NMR (101 MHz, $CDCl_3$) δ 173.2, 171.6, 170.7, 164.8, 149.1, 148.5, 137.5, 136.5, 129.4, 129.4, 128.8, 127.2, 126.7, 122.4, 122.4, 54.9, 52.4, 51.0, 49.1, 41.3, 38.1, 24.9, 22.9, 22.0, 17.9. IR (cm^{-1}): 3386, 1745, 1644, 1578, 1054, 744. HRMS *calcd.* for ($C_{25}H_{32}N_4O_5$): 468.2373, *found* 468.2378

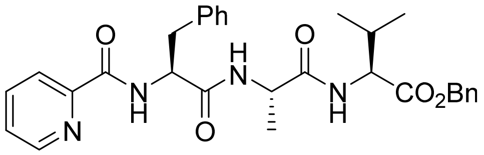

(4g)

PA-Phe-Ala-Val-OBn

Benzyl picolinoyl-*L*-phenylalanyl-*L*-alanyl-*L*-valinate (4g). Following the procedure above, H-Phe-Ala-Val-OBn (1.65 mmol, 700 mg) provided 510 mg (58% yield) of **4g** as a white solid. Mp 143-144 °C. ^{1}H NMR (400 MHz, $CDCl_3$) δ 8.62 – 8.51 (m, 2H), 8.14 (d, *J* = 7.8 Hz, 1H), 7.85 (td, *J* = 7.7, 1.7 Hz, 1H), 7.46 (ddd, *J* = 7.6, 4.7, 1.2 Hz, 1H), 7.38 – 7.36 (m, 3H), 7.32 – 7.18 (m, 5H), 6.75 (dd, *J* = 10.7, 7.9 Hz, 2H), 5.18 (dd, *J* = 36, 16 Hz, 2H), 4.89 (q, *J* = 6.8 Hz, 1H), 4.58 – 4.45 (m, 2H), 3.24 (d, *J* = 7.4 Hz, 2H), 2.29 – 2.12 (m, 1H), 1.32 (d, *J* = 7.0 Hz, 3H), 0.90 (dd, *J* = 16.1, 6.9 Hz, 6H). ^{13}C NMR (101 MHz, $CDCl_3$) δ 171.8, 171.6, 170.8, 164.7, 149.1, 148.4, 137.5, 136.5, 135.4, 129.4, 128.8, 128.7, 128.6, 128.5, 127.2, 126.7, 122.5, 67.2, 57.4, 54.7, 49.2, 38.1, 31.2, 19.1, 18.0, 17.8.. IR (cm^{-1}): 3304, 2969, 1738, 1647, 1520, 1373, 1203. HRMS *calcd.* for ($C_{30}H_{34}N_4O_5$): 530.2529, *found* 530.2543.

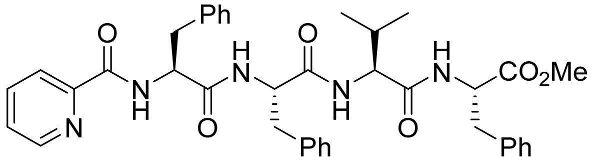

(4h)

PA-Phe-Phe-Val-Phe-OMe

Methyl picolinoyl-*L*-phenylalanyl-*L*-phenylalanyl-*L*-valyl-*L*-phenylalaninate (4h). Following the procedure above, H-Phe-Phe-Val-Phe-OMe (0.87 mmol, 500 mg) provided 490 mg (83% yield) of **4h** as a white solid. Mp 193-194°C ^{1}H NMR (400 MHz, $CDCl_3$) δ 8.57 (dd, *J* = 16.7, 6.0 Hz, 2H), 8.03 (d, *J* = 7.7 Hz, 1H), 7.84 (td, *J* = 7.7, 1.7 Hz, 1H), 7.48 (ddd, *J* = 7.6, 4.8, 1.2 Hz, 1H), 7.36 – 7.21 (m, 7H), 7.20 – 7.14 (m, 3H), 7.08 (dd, *J* = 13.4, 7.8 Hz, 2H), 6.99 – 6.95 (m, 5H), 6.62 (d, *J* = 8.7 Hz, 1H), 4.97 – 4.84 (m, 2H), 4.69 (q, *J* = 6.7 Hz, 1H), 4.42 (dd, *J* = 8.7, 6.5 Hz, 1H), 3.73 (s, 3H), 3.30 – 3.05 (m, 5H), 2.88 (dd, *J* = 13.9, 6.6 Hz, 1H), 2.12 (q, *J* = 6.7 Hz, 1H), 0.84 (dd, *J* = 33.2, 6.8 Hz, 6H). ^{13}C NMR (101 MHz, $CDCl_3$) δ 172.0, 170.7, 170.6, 170.6, 164.7, 148.9, 148.4, 137.4, 136.4, 136.3, 136.0, 129.4, 129.4, 129.2, 128.8, 128.7,

128.6, 127.2, 127.1, 126.9, 126.7, 122.4, 58.7, 54.8, 54.5, 53.5, 52.4, 38.0, 37.9, 30.7, 19.2, 18.1. IR (cm^{-1}): 3290, 2928, 1744, 1639, 1518, 1216. HRMS *calcd.* for ($C_{39}H_{43}N_5O_6$): 677.3213, *found* 677.3227.

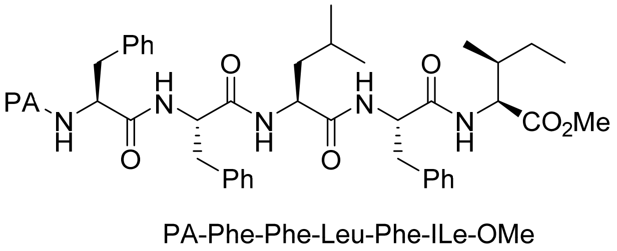

(**4i**)

Methyl picolinoyl-*L*-phenylalanyl-*L*-phenylalanyl-*L*-leucyl-*L*-phenylalanyl-*L*-isoleucinate (4i). Following the procedure above, H-Phe-Phe-Leu-Ile-OMe (0.71 mmol, 500 mg) provided 350 mg (61% yield) of **4i** as a white solid. Mp 181-182°C. ^{1}H NMR (400 MHz, $CDCl_3$) δ 8.65 (d, *J* = 4.0 Hz, 1H), 8.52 (d, *J* = 4.1 Hz, 1H), 7.86 (td, *J* = 7.6, 1.7 Hz, 1H), 7.75 (d, *J* = 7.8 Hz, 1H), 7.60 – 7.53 (m, 1H), 7.42 (d, *J* = 7.3 Hz, 2H), 7.37 (d, *J* = 7.2 Hz, 1H), 7.31 (d, *J* = 7.0 Hz, 4H), 7.22 (q, *J* = 8.2, 7.5 Hz, 3H), 7.14 (d, *J* = 7.2 Hz, 1H), 7.04 (d, *J* = 8.3 Hz, 1H), 6.90 (d, *J* = 7.1 Hz, 1H), 6.76 (s, 4H), 6.72 – 6.67 (m, 1H), 6.27 (d, *J* = 6.0 Hz, 1H), 4.85 – 4.73 (m, 1H), 4.57 (dd, *J* = 8.5, 5.9 Hz, 1H), 4.48 – 4.39 (m, 2H), 4.37 – 4.25 (m, 1H), 3.75 (s, 3H), 3.50 (dd, *J* = 14.2, 4.5 Hz, 1H), 3.34 – 3.15 (m, 4H), 3.06 (dd, *J* = 14.2, 10.6 Hz, 1H), 2.70 (dd, *J* = 14.0, 5.9 Hz, 1H), 1.63 – 1.51 (m, 1H), 1.45 – 1.27 (m, 4H), 1.00 (d, *J* = 7.0 Hz, 6H), 0.89 (dd, *J* = 15.2, 6.5 Hz, 6H). ^{13}C NMR (101 MHz, $CDCl_3$) δ 172.5, 171.9, 171.6, 171.3, 171.0, 165.8, 148.6, 148.1, 138.1, 137.5, 135.6, 135.0, 129.5, 129.2, 129.2, 128.8, 128.5, 128.4, 127.8, 127.2, 127.1, 126.4, 122.3, 57.1, 56.6, 54.6, 54.2, 53.0, 52.1, 39.4, 37.6, 37.3, 37.0, 36.0, 25.3, 24.3, 23.5, 20.8, 15.5, 11.8. IR (cm^{-1}): 3279, 3063, 2929, 1741, 1636, 1518, 1203, 733, 697. HRMS *calcd.* for ($C_{46}H_{56}N_6O_7$): 804.4210, *found* 804.4202.

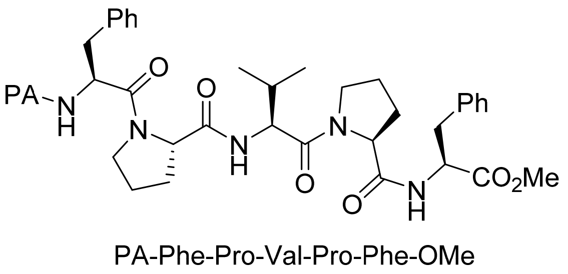

(**4j**)

Methyl picolinoyl-*L*-phenylalanyl-*L*-prolyl-*L*-valyl-*L*-prolyl-*L*-phenylalaninate (4j). Following the procedure above, H-Phe-Pro-Val-Pro-Phe-OMe (0.81 mmol, 500 mg) provided 440 mg (75% yield) of **4j** as a white solid. Mp 92-93°C. ^{1}H NMR (400 MHz, $CDCl_3$) δ 8.74 (d, *J* = 8.6 Hz, 1H), 8.59 (d, *J* = 4.9 Hz, 1H), 8.12 (d, *J* = 7.8 Hz, 1H), 7.83 (t, *J* = 7.7 Hz, 1H), 7.44 (dd, *J* = 7.6, 4.8 Hz, 1H), 7.35 – 7.16 (m, 11H), 7.06

(dd, *J* = 7.1, 4.8 Hz, 1H), 5.17 (q, *J* = 7.2 Hz, 1H), 4.84 – 4.74 (m, 1H), 4.65 – 4.52 (m, 3H), 3.86 – 3.75 (m, 1H), 3.71 (s, 3H), 3.64 – 3.51 (m, 1H), 3.51 – 3.31 (m, 1H), 3.28 – 3.04 (m, 6H), 2.34 – 2.18 (m, 2H), 2.08 – 1.89 (m, 4H), 1.77 – 1.73 (m, 2H), 0.96 (t, *J* = 6.3 Hz, 6H). ^{13}C NMR (101 MHz, $CDCl_3$) δ 171.8, 171.8, 171.4, 171.0, 170.9, 164.0, 149.5, 148.5, 137.3, 136.6, 136.1, 129.5, 129.4, 128.7, 128.7, 127.2, 127.1, 126.4, 122.3, 60.4, 60.1, 56.0, 53.8, 52.5, 52.4, 47.8, 47.7, 39.5, 38.1, 31.5, 27.9, 27.6, 25.3, 25.2, 19.7, 18.0. IR (cm^{-1}): 2928, 2857, 1741, 1673, 1510, 1433, 1211, 750. HRMS *calcd.* for ($C_{40}H_{48}N_6O_7$): 724.3584, *found* 724.3586.

4.-Pd-Catalyzed δ-C(sp^2)-H Acylation of Phe Derivatives

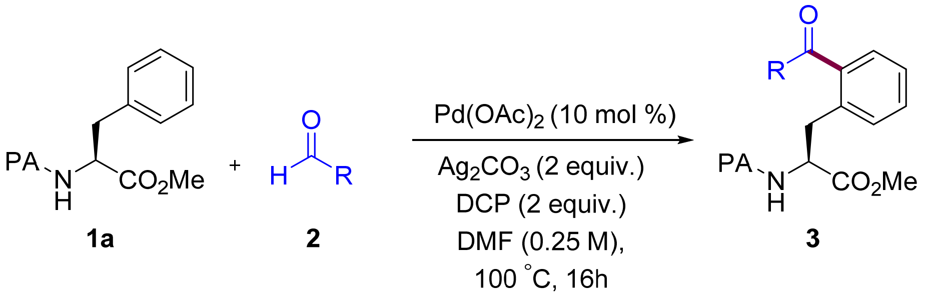

General Procedure: A reaction tube containing a stirring bar was charged with the corresponding Phe derivative **1** (0.25 mmol, 1.00 equiv.), $Pd(OAc)_2$ (10 mol %), Ag_2CO_3 (0.50 mmol, 2.00 equiv.), DCP (0.50 mmol, 2.00 equiv.) and the corresponding aldehyde **2a** (if solid) (1.25 mmol, 5.00 equiv.). The reaction tube was then evacuated and back-filled with dry Ar (this sequence was repeated up to three times). The corresponding aldehyde **2** (if liquid) (1.25 mmol, 5.00 equiv.) and DMF (1 mL) were added under argon atmosphere. The reaction tube was next warmed up to 100 °C and stirred for 16 h. The mixture was then allowed to warm to room temperature, diluted with EtOAc and washed with aq. $NaHCO_3$ (20 mL). The aqueous layer was extracted with EtOAc (3 x 20 mL) and evaporated. The resulting crude was then purified by column chromatography to afford the corresponding product.

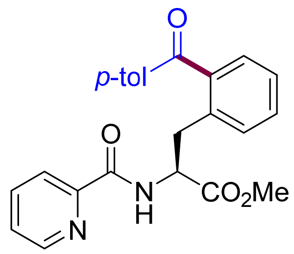

(3a)

Methyl (*S*)-3-(2-(4-methylbenzoyl)phenyl)-2-(picolinamido)propanoate (3a). Following the general procedure, using commercially available 4-methylbenzaldehyde (1.25 mmol, 150 µL) and **1a**[1] (0.25 mmol, 71 mg) provided 79 mg (79% yield) (7:3 ratio) of **3a:3a'** as a colorless oil. ^{1}H NMR (400 MHz, $CDCl_3$) δ 8.82 (d, J = 8.0 Hz, 1H), 8.47 (d, J = 4.4 Hz, 1H), 8.05 (d, J = 7.8 Hz, 1H), 7.78 (td, J = 7.7, 1.7 Hz, 1H), 7.68 (d, J = 8.2 Hz, 2H), 7.49 – 7.41 (m, 2H), 7.41 – 7.35 (m, 1H), 7.33 – 7.27 (m, 2H), 7.16 (d, J = 8.0 Hz, 2H), 5.05 – 4.91 (m, 1H), 3.67 (s, 3H), 3.47 – 3.31 (m, 2H), 2.39 (s, 3H).^{13}C NMR (101 MHz, $CDCl_3$) δ 197.7, 172.0, 164.3, 149.3, 148.1, 144.2, 138.9, 137.4, 136.4, 135.1, 131.3, 130.9, 130.8, 129.8, 129.1, 126.3, 122.5, 54.4, 52.6, 34.7, 21.9. IR (cm^{-1}): 3373, 2970, 1742, 1657, 1603, 1512, 1260. HRMS *caldc* for ($C_{24}H_{22}N_2O_4$): 402.1580, *found* 402.1579. *This reaction was also performed in a higher*

---

[1] He, G.; Zhao, Y.; Zhang, S.; Lu, C.; Chen, G. *J. Am. Chem. Soc.* **2012**, *134*, 3.

*scale: the use of **1a** (3.52 mmol, 1.00 g) and **2a** (2.08 mL) in DMF (12 mL) provided 1.33 g (82% yield) of **3a** (1:1 ratio) as a yellowish oil.*

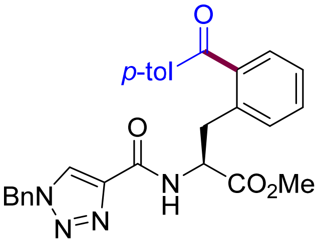

(Trz-3a)

Methyl (*S*)-2-(1-benzyl-1*H*-1,2,3-triazole-4-carboxamido)-3-(2-(4-methylbenzoyl)phenyl)propanoate (Trz-3a). Following the general procedure, using commercially available 4-methylbenzaldehyde (1.25 mmol, 150 µL) and **1b**[2] (0.25 mmol, 91 mg) provided 70 mg (58% yield) (3:1 ratio) of **Trz-3a** as a colorless oil. *The following data correspond to a mixture of mono- and difunctionalized compounds:* ^{1}H NMR (400 MHz, $CDCl_3$) δ 8.83 (d, *J* = 7.6 Hz, 0.5H), 8.52 (d, *J* = 7.3 Hz, 0.5H), 7.89 (d, *J* = 9.3 Hz, 1H), 7.76 (t, *J* = 8.4 Hz, 3H), 7.50 – 7.42 (m, 2H), 7.41 – 7.36 (m, 3H), 7.28 (dp, *J* = 10.5, 3.9 Hz, 6H), 5.61 – 5.42 (m, 2H), 4.98 – 4.89 (m, 0.5H), 4.79 – 4.66 (m, 0.5H), 3.78 – 3.72 (m, 0.5H), 3.69 (s, 1.5H), 3.63 (s, 1.5H), 3.38 (dd, *J* = 13.8, 5.1 Hz, 0.5H), 3.30 – 3.15 (m, 1H), 2.44 (s, 4.5H). ^{13}C NMR (101 MHz, $CDCl_3$) δ 197.8, 197.4, 171.8, 171.4, 160.3, 160.3, 144.9, 144.4, 143.5, 143.2, 140.6, 138.7, 136.3, 135.2, 135.1, 134.7, 134.0, 133.9, 131.1, 131.0, 131.0, 130.9, 129.9, 129.4, 129.3, 129.2, 129.1, 129.1, 128.4, 128.4, 126.3, 125.5, 54.5, 54.4, 54.3, 53.6, 52.5, 52.5, 34.4, 30.5, 21.9, 21.9. IR (cm^{-1}): 3300, 2951, 1742, 1659, 1603, 1588, 1265, 1211. HRMS *caldc* for **Trz-3a** ($C_{28}H_{26}N_4O_4$): 482.1954 *found,* 482.1952.

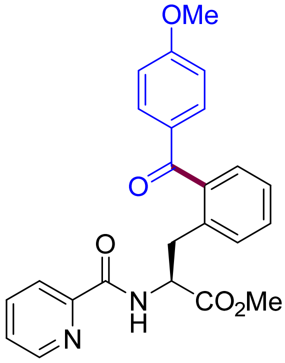

(3b)

Methyl (*S*)-3-[2-(4-methoxybenzoyl)phenyl]-2-(picolinamido)propanoate (3b). Following the general procedure, using commercially available 4-methoxybenzaldehyde (1.25 mmol, 153 mg) and **1a** (0.25 mmol, 71 mg) provided 91 mg (80% yield) (3:1 ratio) of **3b** as a colorless oil. *Both isomers were separated and independently characterized. Monofunctionalized **3b**:* ^{1}H NMR (400 MHz, $CDCl_3$) δ

[2] Ye, X.; He, Z.; Ahmed, T.; Weise, K.; Akhmedov, N. G.; Petersen, J. L.; Shi, X. *Chem. Sci.* **2013**, *4*, 3712.

8.79 (d, J = 8.0 Hz, 1H), 8.47 (d, J = 4.3 Hz, 1H), 8.03 (d, J = 7.8 Hz, 1H), 7.77 (d, J = 8.7 Hz, 3H), 7.46 – 7.40 (m, 2H), 7.39 – 7.33 (m, 1H), 7.32 – 7.23 (m, 2H), 6.83 (d, J = 8.8 Hz, 2H), 5.05 – 4.90 (m, 1H), 3.84 (s, 3H), 3.67 (s, 3H), 3.45 – 3.29 (m, 2H). ^{13}C NMR (101 MHz, $CDCl_3$) δ 196.6, 172.0, 164.6, 163.8, 149.5, 148.4, 139.2, 137.1, 136.2, 133.1, 131.2, 130.6, 130.5, 129.4, 126.2, 126.2, 122.3, 113.6, 55.6, 54.3, 52.5, 34.7. IR (cm^{-1}): 2953, 1741, 1675, 1652, 1596, 1510, 1256. HRMS *caldc* for ($C_{24}H_{22}N_2O_5$): 418.1529, *found* 418.1535. *Difunctionalized **3b'**:* ^{1}H NMR (400 MHz, $CDCl_3$) δ 8.74 (d, J = 8.7 Hz, 1H), 8.35 (d, J = 4.1 Hz, 1H), 7.96 (d, J = 7.8 Hz, 1H), 7.82 (d, J = 8.9 Hz, 4H), 7.70 (td, J = 7.7, 1.7 Hz, 1H), 7.43 – 7.37 (m, 2H), 7.34 – 7.27 (m, 2H), 6.83 (d, J = 8.8 Hz, 4H), 4.89 – 4.81 (m, 1H), 3.85 (s, 6H), 3.63 – 3.58 (m, 1H), 3.30 (s, 3H), 3.49 – 3.41 (m, 1H). ^{13}C NMR (101 MHz, $CDCl_3$) δ 196.3, 171.7, 164.6, 164.0, 149.6, 148.2, 141.0, 136.7, 135.2, 133.1, 130.5, 130.2, 125.8, 125.4, 122.2, 113.8, 55.6, 53.9, 52.6, 30.9. IR (cm^{-1}): 3100, 2930, 2846, 1742, 1653, 1595, 1509, 1255, 1172, 1153, 1025, 848, 605. HRMS *caldc* for ($C_{32}H_{28}N_2O_7$): 552.1897, *found* 552.1910.

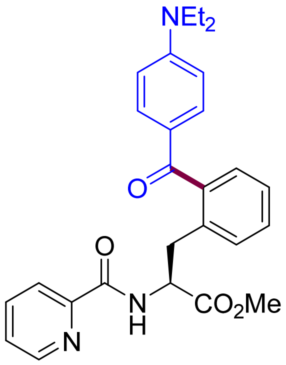

(3c)

Methyl (*S*)-3-[2-(4-(diethylamino)benzoyl)phenyl]-2-(picolinamido)propanoate (3c). Following the general procedure, using commercially available 4-diethylaminobenzaldehyde (1.25 mmol, 222 mg) and **1a** (0.25 mmol, 71 mg) provided 98 mg (78% yield) (3:1 ratio) of **3c** as a colorless oil. *Both isomers were separated and the following data correspond to the monofunctionalized **3c**:* ^{1}H NMR (400 MHz, $CDCl_3$) δ 8.99 (d, J = 7.7 Hz, 1H), 8.53 (d, J = 6.0 Hz, 1H), 8.07 (d, J = 7.8 Hz, 1H), 7.77 (t, J = 7.7 Hz, 1H), 7.72 (d, J = 8.9 Hz, 2H), 7.46 – 7.30 (m, 4H), 7.30 – 7.22 (m, 1H), 6.56 (d, J = 8.7 Hz, 2H), 5.02 – 4.92 (m, 1H), 3.68 (s, 3H), 3.42 (q, J = 7.2 Hz, 4H), 3.35 (t, J = 7.2 Hz, 2H), 1.21 (t, J = 7.0 Hz, 6H).^{13}C NMR (101 MHz, $CDCl_3$) δ 195.7, 172.1, 164.7, 151.5, 149.6, 148.4, 140.1, 137.0, 135.6, 133.4, 130.6, 129.8, 129.0, 126.0, 124.5, 122.2, 110.0, 54.5, 52.4, 44.6, 34.5, 12.6. IR (cm^{-1}): 3380, 2969, 1742, 1671, 1584, 1516, 1268, 1194, 1145. HRMS *caldc* for ($C_{27}H_{29}N_3O_4$): 459.2158, *found* 459.2165.

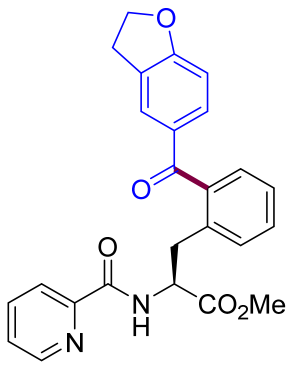

(3d)

Methyl (*S*)-3-[2-(2,3-dihydrobenzofuran-5-carbonyl)phenyl]-2-(picolinamido)propanoate (3d). Following the general procedure, using commercially available 2,3-dihydrobenzofuran-5-carboxyaldehyde (1.25 mmol, 157 μL) and **1a** (0.25 mmol, 71 mg) provided 73 mg (63% yield) (7:3 ratio) of **3d** as a colorless oil. *Both isomers were separated and independently characterized. Monofunctionalized **3d**:* ^{1}H NMR (400 MHz, $CDCl_3$) δ 8.81 (d, *J* = 8.0 Hz, 1H), 8.49 (d, *J* = 4.7 Hz, 1H), 8.05 (d, *J* = 7.8 Hz, 1H), 7.78 (td, *J* = 7.7, 1.7 Hz, 1H), 7.72 (s, 1H), 7.60 (dd, *J* = 8.4, 1.9 Hz, 1H), 7.48 – 7.40 (m, 2H), 7.40 – 7.34 (m, 1H), 7.33 – 7.23 (m, 2H), 6.69 (d, *J* = 8.4 Hz, 1H), 5.08 – 4.90 (m, 1H), 4.65 (t, *J* = 9.4 Hz, 2H), 3.69 (s, 3H), 3.47 – 3.30 (m, 2H), 3.26 – 3.11 (m, 2H). ^{13}C NMR (101 MHz, $CDCl_3$) δ 196.5, 172.0, 164.7, 164.5, 149.4, 148.3, 139.4, 137.0, 136.0, 133.2, 131.0, 130.8, 130.4, 129.2, 127.8, 127.6, 126.2, 126.1, 122.2, 108.9, 72.4, 54.3, 52.5, 34.6, 29.0. IR (cm^{-1}): 3379, 2952, 1742, 1675, 1649, 1602, 1513, 1435, 1294, 1270, 1214, 1086, 939. HRMS *caldc* for ($C_{25}H_{22}N_2O_5$): 430.1529, *found* 430.1522. *Difunctionalized **3d'***: ^{1}H NMR (400 MHz, $CDCl_3$) δ 8.84 (d, *J* = 8.6 Hz, 1H), 8.41 (d, *J* = 4.1 Hz, 1H), 7.99 (d, *J* = 7.8 Hz, 1H), 7.81 – 7.72 (m, 3H), 7.68 (dd, *J* = 8.4, 1.9 Hz, 2H), 7.42 – 7.40 (m, 2H), 7.38 – 7.27 (m, 2H), 6.73 (d, *J* = 8.4 Hz, 2H), 4.93 – 4.80 (m, 1H), 4.78 – 4.55 (m, 4H), 3.64 (s, 3H), 3.61 – 3.57 (m, 1H), 3.45 (dd, *J* = 14.1, 11.0 Hz, 1H), 3.33 – 3.12 (m, 4H). ^{13}C NMR (101 MHz, $CDCl_3$) δ 196.2, 171.7, 165.0, 164.6, 149.6, 148.2, 141.3, 136.7, 134.8, 133.2, 130.5, 130.2, 128.0, 127.7, 125.8, 125.4, 122.1, 109.1, 72.4, 53.8, 52.5, 30.8, 29.0. IR (cm^{-1}): 2953, 1742, 1673, 1651, 1584, 1263, 1245. HRMS *caldc* for ($C_{34}H_{28}N_2O_7$): 576.1897, *found* 576.1889.

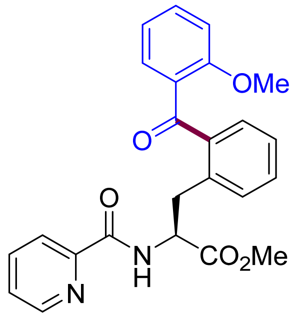

(3e)

Methyl (*S*)-3-[2-(2-methoxybenzoyl)phenyl]-2-(picolinamido)propanoate (3e). Following the general procedure, using commercially available 2-methoxybenzaldehyde (1.25 mmol, 151 μL) and **1a** (0.25 mmol, 71 mg) provided 97 mg (81% yield) (3:2 ratio) of **3e** as a colorless oil. *Both isomers were separated and independently characterized. Monofunctionalized **3e**:* ^{1}H NMR (400 MHz, $CDCl_3$) δ 8.88 (d, *J* = 8.0 Hz, 1H), 8.49 (d, *J* = 4.0 Hz, 1H), 8.09 (d, *J* = 7.8 Hz, 1H), 7.78 (td, *J* = 7.7, 1.7 Hz, 1H), 7.57 – 7.44 (m, 2H), 7.44 – 7.35 (m, 3H), 7.35 – 7.27 (m, 1H), 7.26 – 7.12 (m, 1H), 7.00 (t, *J* = 7.2 Hz, 1H), 6.93 (d, *J* = 8.3 Hz, 1H), 5.15 – 5.04 (m, 1H), 3.75 (s, 3H), 3.69 – 3.64 (m, 1H), 3.65 (s, 3H), 3.54 (dd, *J* = 13.6, 9.2 Hz, 1H). ^{13}C NMR (101 MHz, $CDCl_3$) δ 198.2, 172.2, 164.5, 158.4, 149.6, 148.3, 139.7, 137.1, 136.8, 133.2, 131.5, 131.2, 131.1, 130.8, 129.2, 126.5, 126.2, 122.3, 120.5, 111.7, 55.6, 54.4, 52.5, 35.0. IR (cm^{-1}): 3110, 1739, 1672, 1514, 1365, 1228, 1216. HRMS *caldc* for ($C_{24}H_{22}N_2O_5$): 418.1529, *found* 418.1533. *Difunctionalized **3e'**:* ^{1}H NMR (400 MHz, $CDCl_3$) δ 9.28 (d, *J* = 8.0 Hz, 1H), 8.46 (d, *J* = 0.8 Hz, 1H), 8.06 (d, *J* = 7.8 Hz, 1H), 7.74 (td, *J* = 7.7, 1.7 Hz, 1H), 7.66 (dd, *J* = 7.6, 1.8 Hz, 2H), 7.55 – 7.45 (m, 2H), 7.37 (d, *J* = 7.7 Hz, 2H), 7.35 – 7.26 (m, 1H), 7.17 (t, *J* = 7.7 Hz, 1H), 7.03 (t, *J* = 8.0 Hz, 2H), 6.90 (d, *J* = 8.4 Hz, 2H), 5.13 – 5.01 (m, 1H), 4.03 (dd, *J* = 13.8, 4.3 Hz, 1H), 3.72 (s, 3H), 3.69 – 3.64 (m, 1H), 3.60 (s, 6H). ^{13}C NMR (101 MHz, $CDCl_3$) δ 197.9, 172.2, 164.9, 158.8, 150.1, 148.3, 142.1, 136.8, 135.4, 133.9, 131.7, 131.6, 128.7, 125.8, 125.6, 122.1, 120.6, 111.9, 55.6, 54.2, 52.5, 29.6. IR (cm^{-1}): 3301, 2949, 1745, 1667, 1596, 1514, 1485, 1462, 1434, 1294, 1249, 1020, 756. HRMS *calc* for ($C_{32}H_{28}N_2O_7$): 552.1897, *found* 552.1895.

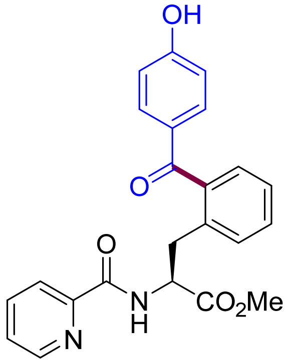

(3f)

Methyl (*S*)-3-[2-(4-hydroxybenzoyl)phenyl]-2-(picolinamido)propanoate (3f). Following the general procedure, using commercially available 4-hydroxybenzaldehyde (1.25 mmol, 153 mg) and **1a** (0.25 mmol, 71 mg) provided 64 mg (63% yield) of **3f** as a colorless oil. ^{1}H NMR (400 MHz, $CDCl_3$) δ 8.86 (d, *J* = 8.0 Hz, 1H), 8.50 (d, *J* = 4.3 Hz, 1H), 8.03 (d, *J* = 8.1 Hz, 1H), 7.78 (td, *J* = 7.7, 1.6 Hz, 1H), 7.70 (d, *J* = 8.6 Hz, 2H), 7.43 – 7.36 (m, 3H), 7.33 – 7.26 (m, 2H), 6.77 (d, *J* = 8.7

Hz, 2H), 5.06 – 4.92 (m, 1H), 3.67 (s, 3H), 3.43 (dd, *J* = 13.9, 5.5 Hz, 1H), 3.33 (dd, *J* = 13.9, 8.9 Hz, 1H). ^{13}C NMR (101 MHz, $CDCl_3$) δ 196.9, 172.0, 165.1, 161.8, 149.0, 148.5, 139.2, 137.4, 135.8, 133.4, 131.1, 130.5, 129.7, 129.6, 126.6, 126.4, 122.4, 115.5, 54.5, 52.7, 34.9. IR (cm^{-1}): 3288, 2969, 1741, 1652, 1585, 1512, 1434, 1269, 1236, 1149. HRMS *calcd.* for ($C_{23}H_{20}N_2O_5$): 404.1372, *found* 404.1373.

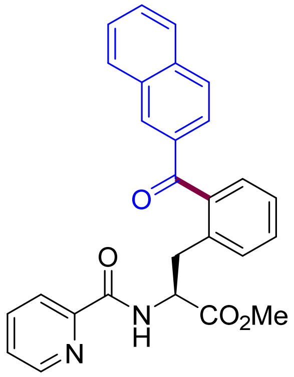

(3g)

Methyl (*S*)-3-[2-(2-naphthoyl)phenyl]-2-(picolinamido)propanoate (3g). Following the general procedure, using commercially available 2-naphthaldehyde (1.25 mmol, 195 mg) and **1a** (0.25 mmol, 71 mg) provided 59 mg (54% yield) of **3g** as a colorless oil. ^{1}H NMR (400 MHz, $CDCl_3$) δ 8.72 (d, *J* = 8.2 Hz, 1H), 8.24 (d, *J* = 4.0 Hz, 1H), 8.11 (s, 1H), 8.03 – 7.92 (m, 2H), 7.83 (t, *J* = 7.7 Hz, 2H), 7.77 (d, *J* = 8.1 Hz, 1H), 7.64 – 7.55 (m, 2H), 7.53 – 7.45 (m, 3H), 7.39 (d, *J* = 7.4 Hz, 1H), 7.34 – 7.27 (m, 1H), 7.14 (ddd, *J* = 7.5, 4.8, 1.0 Hz, 1H), 5.08 – 4.96 (m, 1H), 3.68 (s, 3H), 3.56 – 3.39 (m, 2H). ^{13}C NMR (101 MHz, $CDCl_3$) δ 197.8, 172.0, 164.5, 149.2, 148.1, 138.8, 136.9, 136.8, 135.7, 135.0, 133.4, 132.2, 131.5, 131.0, 130.0, 129.9, 128.7, 128.3, 127.8, 126.8, 126.3, 125.9, 125.6, 122.1, 54.4, 52.6, 34.7. IR (cm^{-1}): 3372, 3057, 2924, 2852, 1714, 1672, 1511, 1292, 1275, 1233, 908, 727. HRMS *caldc* for ($C_{27}H_{22}N_2O_4$): 438.1580, *found* 438.1587.

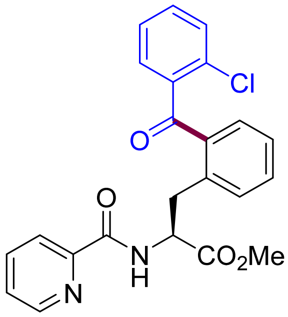

(3h)

Methyl (*S*)-3-[2-(2-chlorobenzoyl)phenyl]-2-(picolinamido)propanoate (3h). Following the general procedure, using commercially available 2-chlorobenzaldehyde (1.25 mmol, 141 μL) and **1a** (0.25 mmol, 71 mg) provided 56 mg (53% yield) of **3h** as a colorless oil. ^{1}H NMR (400 MHz, $CDCl_3$) δ 8.90 (d, *J* = 8.2 Hz, 1H), 8.56 (d, *J* = 4.8 Hz, 1H), 8.10 (d, *J* = 7.8 Hz, 1H), 7.80 (td, *J* = 7.7, 1.7 Hz, 1H), 7.52 – 7.39 (m, 6H),

7.39 – 7.28 (m, 2H), 7.28 – 7.17 (m, 1H), 5.23 – 5.12 (m, 1H), 3.78 (s, 3H), 3.73 – 3.56 (m, 2H). ^{13}C NMR (101 MHz, $CDCl_3$) δ 197.3, 172.2, 164.5, 149.5, 148.3, 139.2, 138.4, 137.2, 137.1, 132.5, 132.3, 132.2, 131.8, 130.5, 130.4, 126.9, 126.2, 126.3, 122.3, 54.1, 52.6, 35.6. IR (cm^{-1}): 3220, 1742, 1672, 1515, 1434, 1297, 1250, 931, 747. HRMS *caldc* for ($C_{23}H_{19}ClN_2O_4$): 422.1033, *found* 422.1031.

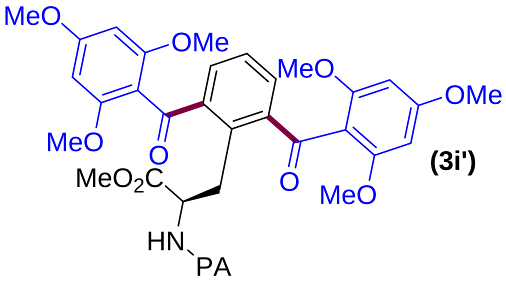

(3i')

Methyl (*S*)-3-[2,6-bis(2,4,6-trimethoxybenzoyl)phenyl]-2-(picolinamido) propanoate (3i'). Following the general procedure, using commercially available 2,4,6-trimethoxybenzaldehyde (1.25 mmol, 245 mg) and **1a** (0.25 mmol, 71 mg) provided 112 mg (67% yield) of **3i'** as a white solid. Mp 91-92 °C. ^{1}H NMR (400 MHz, $CDCl_3$) δ 9.77 (d, J = 7.2 Hz, 1H), 8.58 (d, J = 4.5 Hz, 1H), 8.07 (d, J = 7.8 Hz, 1H), 7.73 (td, J = 7.7, 1.7 Hz, 1H), 7.46 (d, J = 7.7 Hz, 2H), 7.32 (dd, J = 6.6, 4.8 Hz, 1H), 7.10 (t, J = 7.7 Hz, 1H), 6.10 (s, 4H), 5.15 – 5.06 (m, 1H), 4.36 (dd, J = 13.5, 4.2 Hz, 1H), 3.84 (s, 6H), 3.74 (s, 3H), 3.66 (s, 12H), 3.63 – 3.57 (m, 1H). ^{13}C NMR (101 MHz, $CDCl_3$) δ 197.0, 172.7, 165.2, 163.1, 159.5, 150.4, 148.4, 141.9, 136.6, 136.6, 132.6, 125.8, 125.6, 122.0, 112.6, 90.7, 55.9, 55.5, 54.2, 52.2, 29.0. IR (cm^{-1}): 3301, 2944, 1744, 1669, 1602, 1586, 1456, 1226, 1206, 1150, 1127. HRMS *caldc* for ($C_{36}H_{36}N_2O_{11}$): 672.2319, *found* 672.2307.

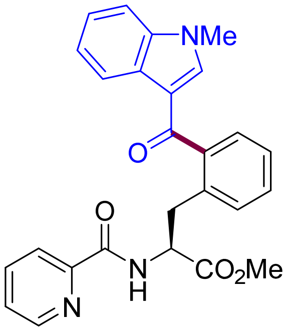

(3j)

Methyl (*S*)-3-[2-(1-methyl-1*H*-indole-3-carbonyl)phenyl]-2-(picolinamido) propanoate (3j). Following the general procedure, using commercially available 1-methylindole-3-carboxyaldehyde (1.25 mmol, 199 mg) and **1a** (0.25 mmol, 71 mg) provided 66 mg (60% yield) of **3j** as a yellowish oil. ^{1}H NMR (400 MHz, $CDCl_3$) δ 8.87 (d, J = 8.0 Hz, 1H), 8.47 – 8.34 (m, 1H), 8.27 (d, J = 4.0 Hz, 1H), 7.93 (d, J = 7.8 Hz, 1H), 7.67 (td, J = 7.7, 1.7 Hz, 1H), 7.48 – 7.36 (m, 3H), 7.35 – 7.29 (m, 2H), 7.28 – 7.22 (m, 4H), 5.03 – 4.86 (m, 1H), 3.67 (s, 3H), 3.64 (s, 3H), 3.52 – 3.38 (m, 2H). ^{13}C

NMR (101 MHz, $CDCl_3$) δ 192.0, 172.1, 164.6, 149.4, 148.1, 141.2, 139.2, 137.6, 136.8, 135.6, 131.0, 130.0, 128.9, 127.0, 126.3, 125.9, 123.7, 123.0, 122.9, 122.0, 117.1, 109.7, 54.5, 52.5, 34.4, 33.6. IR (cm^{-1}): 3374, 2950, 1742, 1673, 1613, 1522, 1464, 1369, 1232, 748. HRMS *caldc* for ($C_{26}H_{23}N_3O_5$): 441.1689, *found* 441.1764.

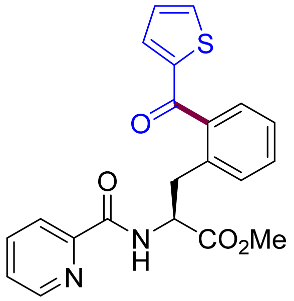

(3k)

Methyl (*S*)-2-(picolinamido)-3-(2-(thiophene-2-carbonyl)phenyl)propanoate (3k). Following the general procedure, using 2-thiophenecarboxaldehyde (1.25 mmol, 114 μL) provided 30 mg (30% yield) of **3k** as a yellowish oil. ^1H NMR (400 MHz, $CDCl_3$) δ 8.80 (d, *J* = 8.1 Hz, 1H), 8.48 (ddd, *J* = 4.9, 1.6, 0.8 Hz, 1H), 8.04 (dt, *J* = 7.9, 1.0 Hz, 1H), 7.76 (td, *J* = 7.7, 1.7 Hz, 1H), 7.66 (dd, *J* = 4.9, 1.2 Hz, 1H), 7.57 – 7.27 (m, 6H), 6.99 (dd, *J* = 4.9, 3.8 Hz, 1H), 5.02 – 4.97 (m, 1H), 3.67 (s, 3H), 3.47 – 3.35 (m, 2H). ^{13}C NMR (101 MHz, $CDCl_3$) δ 189.8, 171.9, 164.5, 149.5, 148.4, 145.0, 138.6, 137.1, 136.1, 135.2, 131.3, 131.1, 129.3, 128.1, 126.4, 126.2, 122.3, 54.3, 52.5, 34.6. IR (neat, cm^{-1}): 3320, 1737, 1670, 1629, 1508. HRMS *calcd.* for ($C_{21}H_{18}N_2O_4S$): 394.0987, *found* 394.0988.

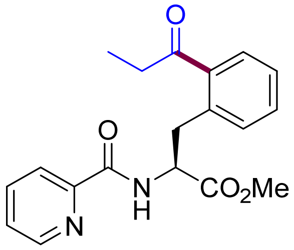

(3m)

Methyl (*S*)-2-(picolinamido)-3-(2-propionylphenyl)propanoate (3m). Following the general procedure, using commercially available propionaldehyde (1.25 mmol, 91 μL) and **1a** (0.25 mmol, 71 mg) provided 45 mg (53% yield) of **3m** as a colorless oil. ^1H NMR (400 MHz, $CDCl_3$) δ 8.83 (d, *J* = 8.2 Hz, 1H), 8.55 (d, *J* = 4.0 Hz, 1H), 8.06 (d, *J* = 7.8 Hz, 1H), 7.78 (td, *J* = 7.7, 1.7 Hz, 1H), 7.68 (d, *J* = 6.4 Hz, 1H), 7.45 – 7.33 (m, 3H), 7.29 (td, *J* = 7.4, 1.8 Hz, 1H), 5.07 – 4.93 (m, 1H), 3.73 (s, 3H), 3.55 – 3.39 (m, 2H), 3.01 – 2.89 (m, 2H), 1.20 (t, *J* = 7.2 Hz, 3H). ^{13}C NMR (101 MHz, $CDCl_3$) δ 205.1, 172.3, 164.5, 149.6, 148.4, 138.3, 137.2, 136.7, 132.2, 131.6, 129.0, 127.1, 126.3, 122.4, 54.3, 52.5, 35.5, 34.7, 8.5. IR (cm^{-1}): 3377, 2938, 1741, 1677, 1514, 1216, 752. HRMS *calcd.* for ($C_{19}H_{20}N_2O_4$): 340.1423, *found* 340.1420

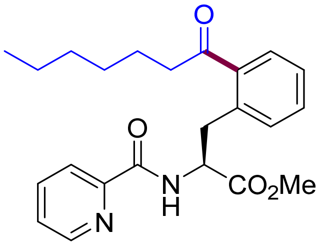

(3n)

Methyl (*S*)-3-(2-heptanoylphenyl)-2-(picolinamido)propanoate (3n). Following the general procedure, using commercially available heptaldehyde (1.25 mmol, 175 µL) and **1a** (0.25 mmol, 71 mg) provided 53.0 (53% yield) of **3n** as a colorless oil. ^{1}H NMR (400 MHz, $CDCl_3$) δ 8.92 (d, *J* = 8.1 Hz, 1H), 8.56 (d, *J* = 4.8 Hz, 1H), 8.08 (d, *J* = 7.9 Hz, 1H), 7.80 (t, *J* = 6.9 Hz, 1H), 7.69 (d, *J* = 7.8 Hz, 1H), 7.48 – 7.35 (m, 3H), 7.35 – 7.25 (m, 1H), 5.09 – 4.95 (m, 1H), 3.75 (s, 3H), 3.56 – 3.38 (m, 2H), 3.00 – 2.87 (m, 2H), 1.79 – 1.67 (m, 2H), 1.43 – 1.22 (m, 6H), 0.98 – 0.81 (m, 3H). ^{13}C NMR (101 MHz, $CDCl_3$) δ 204.9, 172.3, 164.5, 149.6, 148.4, 138.5, 137.2, 136.8, 132.1, 131.6, 129.0, 127.1, 126.3, 122.4, 54.4, 52.5, 41.7, 35.4, 31.8, 29.1, 24.5, 22.7, 14.2. IR (cm^{-1}): 2928, 2857, 1741, 1673, 1510, 1433, 1211. HRMS *calcd.* for ($C_{23}H_{28}N_2O_4$): 396.2049, *found* 396.2050.

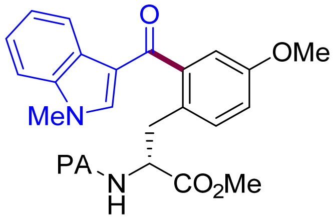

(3p)

Methyl (*D*)-3-[4-methoxy-2-(1-methyl-1*H*-indole-3-carbonyl)phenyl]-2-(picolinamido)propanoate (3p). Following the general procedure, using commercially available 1-methylindole-3-carboxyaldehyde (1.25 mmol, 199 mg) and **1c**[3] (0.25 mmol, 79.0 mg) provided 59 mg (50% yield) of **3p** as a colorless oil. ^{1}H NMR (400 MHz, $CDCl_3$) δ 8.88 (d, *J* = 7.9 Hz, 1H), 8.49 – 8.40 (m, 1H), 8.33 (d, *J* = 4.5 Hz, 1H), 7.98 (d, *J* = 7.8 Hz, 1H), 7.71 (td, *J* = 7.7, 1.7 Hz, 1H), 7.42 – 7.26 (m, 6H), 7.00 (d, *J* = 2.7 Hz, 1H), 6.95 (dd, *J* = 8.5, 2.8 Hz, 1H), 5.02 – 4.86 (m, 1H), 3.78 (s, 3H), 3.71 (s, 3H), 3.67 (s, 3H), 3.48 – 3.32 (m, 2H). ^{13}C NMR (101 MHz, $CDCl_3$) δ 191.6, 172.1, 164.6, 157.6, 149.4, 148.1, 142.2, 139.2, 137.6, 136.8, 132.1, 127.3, 126.9, 125.9, 123.7, 122.9, 122.0, 116.9, 115.2, 114.6, 109.7, 55.5, 54.6, 52.4, 33.7, 33.6. IR (cm^{-1}): 3424, 2950, 1741, 1672, 1604, 1519, 1464, 1367, 1217, 731. HRMS *caldc* for ($C_{27}H_{25}N_3O_5$): 471.1794, *found* 471. 1806.

[3] Kluge, A.; Lagu, B.; Maiti, P.; Panigrahi, S. K. PCT Int. Appl. (**2018**), WO 2018213150 A1.

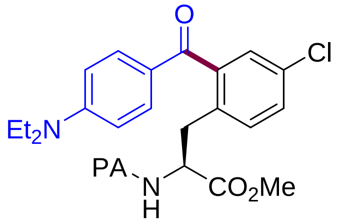

(3q)

Methyl (*S*)-3-[4-chloro-2-(4-(diethylamino)benzoyl)phenyl]-2-(picolinamido) propanoate (3q). Following the general procedure, using commercially available 4-diethylaminobenzaldehyde (1.25 mmol, 222 mg) and **1d**[4] (0.25 mmol, 79.5 mg) provided 85.0 mg (64% yield) (8:2 ratio) of **3q** as a colorless oil. *Both isomers were separated and the following data correspond to the monofunctionalized compound* ***3q:*** ^{1}H NMR (400 MHz, $CDCl_3$) δ 8.87 (d, *J* = 7.9 Hz, 1H), 8.53 (d, *J* = 4.4 Hz, 1H), 8.08 (d, *J* = 7.8 Hz, 1H), 7.78 (td, *J* = 7.7, 1.7 Hz, 1H), 7.69 (d, *J* = 9.1 Hz, 2H), 7.40 – 7.37 (m, 1H), 7.35 (d, *J* = 2.5 Hz, 2H), 7.30 (d, *J* = 1.9 Hz, 1H), 6.56 (d, *J* = 9.1 Hz, 2H), 5.04 – 4.88 (m, 1H), 3.69 (s, 3H), 3.43 (q, *J* = 7.1 Hz, 4H), 3.39 – 3.22 (m, 2H), 1.22 (t, *J* = 7.1 Hz, 6H). ^{13}C NMR (101 MHz, $CDCl_3$) δ 194.0, 171.8, 164.6, 151.7, 149.5, 148.4, 141.8, 137.4, 134.1, 133.4, 132.0, 129.7, 128.6, 126.2, 123.8, 122.3, 110.2, 54.1, 52.5, 44.7, 34.1, 12.6. IR (cm^{-1}): 3371, 2971, 1741, 1673, 1583, 1512, 1407, 1270, 1259, 1190, 1149. HRMS *caldc* for ($C_{27}H_{28}ClN_3O_4$): 493.1768, *found* 493.1773.

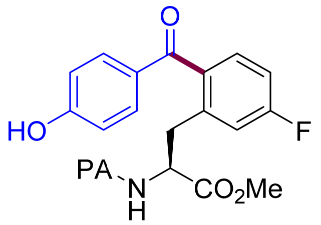

(3r)

Methyl (*S*)-3-[5-fluoro-2-(4-hydroxybenzoyl)phenyl]-2-(picolinamido)propanoate (3r). Following the general procedure, using commercially available 4-hydroxybenzaldehyde (1.25 mmol, 153 μL) and **1e** (0.25 mmol, 76 mg) provided 52 mg (49% yield) of **3r** as a colorless oil. ^{1}H NMR (400 MHz, $CDCl_3$) δ 9.05 (d, *J* = 8.0 Hz, 1H), 8.52 (d, *J* = 4.5 Hz, 2H), 8.03 (d, *J* = 7.7 Hz, 1H), 7.79 (td, *J* = 7.7, 1.7 Hz, 1H), 7.66 (d, *J* = 8.7 Hz, 2H), 7.41 (ddd, *J* = 7.6, 4.7, 1.2 Hz, 1H), 7.33 (dd, *J* = 8.5, 5.8 Hz, 1H), 7.12 (dd, *J* = 9.6, 2.5 Hz, 1H), 6.97 (td, *J* = 8.3, 2.6 Hz, 1H), 6.82 (d, *J* = 8.7 Hz, 2H), 5.09 – 4.96 (m, 1H), 3.71 (s, 3H), 3.47 (dd, *J* = 13.9, 5.3 Hz, 1H), 3.35 (dd, *J* = 13.9, 9.0 Hz, 1H). ^{13}C NMR (101 MHz, $CDCl_3$) δ 195.9, 171.7, 165.2, 163.6 (d, $J_{C\text{-}F}$ = 252.5 Hz), 162.0, 148.8, 148.6, 139.3 (d, $J_{C\text{-}F}$ = 8.0 Hz) , 137.4, 135.3 (d, $J_{C\text{-}F}$ = 3.0 Hz), 133.3, 132.0 (d, $J_{C\text{-}F}$ = 9.0 Hz), 129.5, 126.7, 122.4, 118.1 (d, *J* = 22.2 Hz), 115.5, 113.5 (d, $J_{C\text{-}F}$ = 22.2 Hz), 54.3, 52.8, 34.8. IR (cm^{-1}): 2955, 1742, 1655, 1601, 1585,

[4] Wang, X.; Niu, S.; Xu, L.; Zhang, C.; Meng, L.; Zhang, X.; Ma, D. *Org. Lett.* **2017**, *19*, 246.

1514, 1435, 1311, 1283, 1233, 1160. HRMS *caldc* for ($C_{23}H_{19}FN_2O_5$): 422.1278, *found* 422.1293.

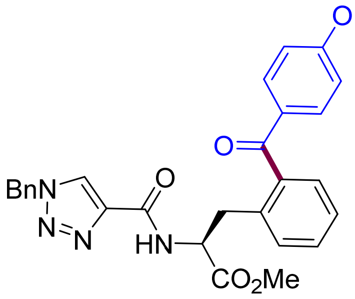

(3s)

Methyl (*S*)-2-(1-benzyl-1*H*-1,2,3-triazole-4-carboxamido)-3-[2-(4-hydroxybenzoyl)phenyl] propanoate (3s). Following the general procedure, using commercially available 4-hydroxybenzaldehyde (1.25 mmol, 153.0 mg) and **1b** (0.25 mmol, 91.0 mg) provided 96.0 mg (72% yield) (6:4 ratio) of **3s** as a colorless oil. *Both isomers were separated and the following data correspond to the monofunctionalized compound **3s**:* ^{1}H NMR (400 MHz, $CDCl_3$) δ 8.76 (d, *J* = 7.2 Hz, 1H), 8.09 (s, 1H), 7.93 (s, 1H), 7.71 (d, *J* = 8.7 Hz, 2H), 7.49 – 7.20 (m, 8H), 6.85 (d, *J* = 8.7 Hz, 2H), 5.63 – 5.46 (m, 2H), 5.00 – 4.87 (m, 1H), 3.72 (s, 3H), 3.44 – 3.20 (m, 2H). ^{13}C NMR (101 MHz, $CDCl_3$) δ 196.9, 171.9, 161.8, 160.6, 142.9, 139.1, 135.9, 133.8, 133.6, 131.0, 130.7, 129.7, 129.7, 129.4, 129.2, 128.4, 126.4, 125.8, 115.5, 54.6, 54.5, 52.7, 34.5. IR (cm^{-1}): 3144, 1745, 1649, 1572, 1272, 1215, 1151. HRMS *caldc* for ($C_{27}H_{24}N_4O_5$): 484.1747, *found* 484.1759.

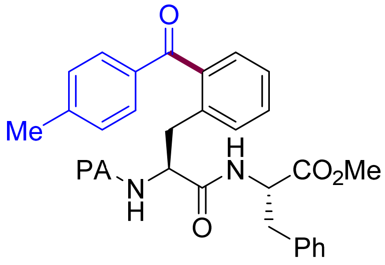

(**5a**)

Methyl [(*S*)-3-(2-(4-methylbenzoyl)phenyl)-2-(picolinamido)propanoyl]-*L*-phenylalaninate (5a). Following the general procedure, using commercially available 4-methylbenzaldehyde (1.25 mmol, 150µL) and PA-Phe-Phe-OMe (0.25 mmol, 108 mg) provided 72 mg (52% yield) of **5a** as a colorless oil. ^{1}H NMR (400 MHz, $CDCl_3$) δ 9.07 (d, *J* = 8.1 Hz, 1H), 8.54 (d, *J* = 4.5 Hz, 1H), 8.03 (d, *J* = 7.8 Hz, 1H), 7.79 (td, *J* = 7.7, 1.7 Hz, 1H), 7.70 (d, *J* = 8.2 Hz, 2H), 7.43 – 7.36 (m, 3H), 7.36 – 7.31 (m, 1H), 7.25 – 7.19 (m, 3H), 7.09 – 7.06 (m, 6H), 4.99 – 4.89 (m, 1H), 4.85 – 4.76 (m, 1H), 3.65 (s, 3H), 3.34 (dd, *J* = 14.0, 4.7 Hz, 1H), 3.21 – 2.99 (m, 3H), 2.43 (s, 3H). ^{13}C NMR (101 MHz, $CDCl_3$) δ 198.2, 171.7, 170.9, 164.6, 149.2, 148.1, 144.3, 138.2, 137.6, 137.5, 136.0, 135.4, 131.3, 131.0, 130.9, 130.1, 129.4, 129.2, 128.5, 127.0,

126.4, 126.1, 122.7, 55.4, 53.6, 52.3, 38.1, 35.4, 21.9. IR (cm^{-1}): 3302, 3029, 2951, 2925, 1742, 1656, 1512, 1433, 1269, 1211, 930, 731. HRMS *calcd.* for ($C_{33}H_{31}N_3O_5$): 549.2264, *found* 549.2268.

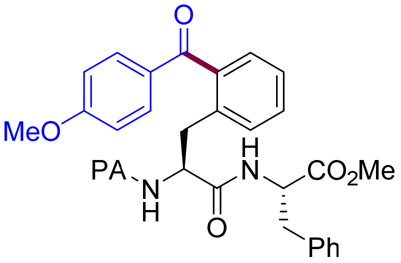

(5b)

Methyl [(*S*)-3-(2-(4-methoxybenzoyl)phenyl)-2-(picolinamido)propanoyl]-*L*-phenylalaninate (5b). Following the general procedure, using commercially available 4-methoxylbenzaldehyde (1.25 mmol, 153µL) and PA-Phe-Phe-OMe (0.25 mmol, 108 mg) along 48h provided 81 mg (57%) of **5b** as a colorless oil. ^{1}H NMR (400 MHz, $CDCl_3$) δ 9.07 (d, *J* = 8.0 Hz, 1H), 8.54 (d, *J* = 4.4 Hz, 1H), 8.02 (d, *J* = 7.8 Hz, 1H), 7.85 – 7.75 (m, 3H), 7.42 – 7.35 (m, 3H), 7.35 – 7.29 (m, 1H), 7.25 – 7.20 (m, 1H), 7.08 – 7.03 (m, 6H), 6.90 (d, *J* = 8.9 Hz, 2H), 4.97 – 4.88 (m, 1H), 4.85 – 4.75 (m, 1H), 3.88 (s, 3H), 3.64 (s, 3H), 3.31 (dd, *J* = 14.0, 4.8 Hz, 1H), 3.20 – 2.99 (m, 3H). ^{13}C NMR (101 MHz, $CDCl_3$) δ 197.1, 171.6, 170.9, 165.0, 163.8, 149.6, 148.4, 138.5, 137.3, 137.1, 136.0, 133.2, 131.1, 130.8, 130.7, 129.7, 129.4, 128.5, 127.0, 126.2, 126.0, 122.4, 113.7, 55.7, 55.2, 53.6, 52.3, 38.1, 35.2. IR (cm^{-1}): 3301, 2929, 1742, 1656, 1596, 1509, 1258. HRMS *calcd.* for ($C_{33}H_{31}N_3O_6$): 565.2213, *found* 565.2212.

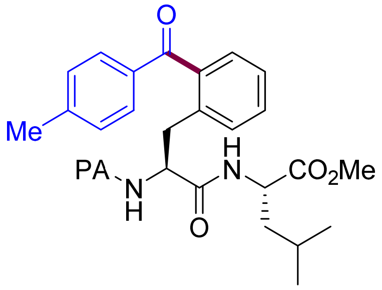

(5c)

Methyl [(*S*)-3-(2-(4-methylbenzoyl)phenyl)-2-(picolinamido)propanoyl]-*L*-leucinate (5c). Following the general procedure, using commercially available 4-methylbenzaldehyde (1.25 mmol, 150 µL) and PA-Phe-Leu-OMe (0.25 mmol, 99.0 mg) provided 56 mg (44% yield) of **5c** as a colorless oil. ^{1}H NMR (400 MHz, $CDCl_3$) δ 9.13 (d, *J* = 8.1 Hz, 1H), 8.57 (d, *J* = 4.6 Hz, 1H), 8.06 (d, *J* = 7.7 Hz, 1H), 7.85 – 7.68 (m, 3H), 7.53 – 7.35 (m, 4H), 7.27 (t, *J* = 7.4 Hz, 3H), 7.06 (d, *J* = 8.1 Hz, 1H), 5.08 – 4.97 (m, 1H), 4.68 – 4.50 (m, 1H), 3.69 (s, 3H), 3.44 (dd, *J* = 13.9, 4.3 Hz, 1H), 3.20 (dd, *J* = 13.9, 10.1 Hz, 1H), 2.45 (s, 3H), 1.68 – 1.59 (m, 3H), 0.90 (d, *J* = 5.0 Hz, 6H). ^{13}C NMR (101 MHz, $CDCl_3$) δ 198.20, 172.91, 171.00, 164.84, 149.43, 148.27, 144.09, 137.84, 137.67, 136.95, 135.27, 131.20, 130.90, 130.77, 130.15, 129.00,

126.07, 125.92, 122.17, 55.16, 52.13, 50.86, 41.33, 35.86, 24.72, 22.72, 21.87, 21.68. IR (cm^{-1}): 3303, 2955, 1743, 1658, 1516, 1269. HRMS *caldc* for ($C_{30}H_{33}N_3O_5$): 515.2420, *found* 515.2418.

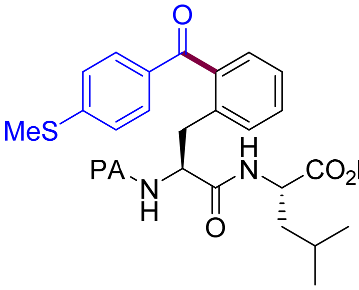

(5d)

Methyl [(*S*)-3-(2-(4-(methylthio)benzoyl)phenyl]-2-(picolinamido)propanoyl)-*L*-leucinate (5d). Following the general procedure, using commercially available 4-(methylthio)benzaldehyde (1.25 mmol, 166 μL) and PA-Phe-Leu-OMe (0.25 mmol, 99.0 mg) provided 45 mg (33% yield) of **5d** as a colorless oil. ^{1}H NMR (400 MHz, $CDCl_3$) δ 9.08 (d, J = 8.2 Hz, 1H), 8.55 (d, J = 4.5 Hz, 1H), 8.05 (d, J = 7.8 Hz, 1H), 7.83 – 7.73 (m, 3H), 7.48 – 7.33 (m, 4H), 7.32 – 7.21 (m, 3H), 7.03 (d, J = 8.1 Hz, 1H), 5.07 – 4.95 (m, 1H), 4.65 – 4.50 (m, 1H), 3.69 (s, 3H), 3.44 (dd, J = 14.0, 4.3 Hz, 1H), 3.21 (dd, J = 14.0, 10.1 Hz, 1H), 2.54 (s, 3H), 1.68 – 1.56 (m, 3H), 0.90 (d, J = 5.8 Hz, 6H). ^{13}C NMR (101 MHz, $CDCl_3$) δ 197.6, 173.1, 171.1, 165.0, 149.5, 148.4, 146.5, 137.9, 137.8, 137.1, 134.1, 131.4, 131.2, 131.1, 130.1, 126.2, 126.1, 124.8, 122.3, 55.3, 52.3, 51.0, 41.5, 35.9, 24.9, 22.9, 22.0, 14.8. IR (cm^{-1}): 2955, 1742, 1655, 1586, 1514, 1434, 1268, 1089. HRMS *caldc* for ($C_{30}H_{33}N_3O_5S$): 547.2141, *found* 547.2143.

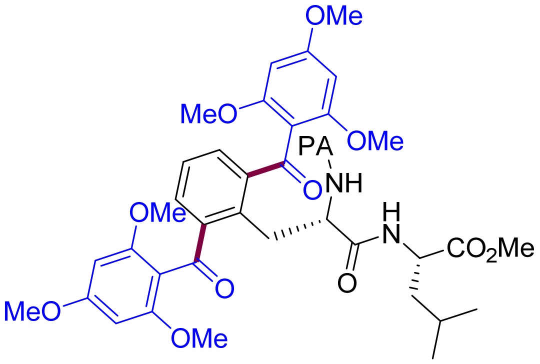

(5e')

Methyl [(*S*)-3-(2,6-bis(2,4,6-trimethoxybenzoyl)phenyl)-2-(picolinamido) propanoyl]-*L*-leucinate (5e'). Following the general procedure, using commercially available 2,4,6-trimethoxybenzaldehyde (1.25 mmol, 245 mg) and PA-Phe-Leu-OMe (0.25 mmol, 99.0 mg) provided 85 mg (43% yield) of **5e'** as a colorless oil. ^{1}H NMR (400 MHz, $CDCl_3$) δ 9.73 (d, J = 7.5 Hz, 1H), 8.55 (d, J = 4.7 Hz, 1H), 8.03 (d, J = 7.8 Hz, 1H), 7.71 (td, J = 7.7, 1.7 Hz, 1H), 7.48 (d, J = 7.7 Hz, 2H), 7.30 (ddd, J = 7.6, 4.7, 1.2 Hz, 1H), 7.21 (d, J = 8.2 Hz, 1H), 7.10 (t, J = 7.7 Hz, 1H), 6.09 (s, 4H), 5.11 – 5.00 (m, 1H), 4.70 – 4.57 (m, 1H), 4.09 (dd, J = 14.1, 2.7 Hz, 1H), 3.83 (s, 6H), 3.79 – 3.71

(m, 1H), 3.68 (s, 3H), 3.65 (s, 12H), 1.72 – 1.55 (m, 3H), 0.86 (dd, J = 5.9, 4.2 Hz, 6H). ^{13}C NMR (101 MHz, $CDCl_3$) δ 197.6, 173.4, 171.9, 165.6, 163.1, 159.4, 150.5, 148.5, 137.9, 136.6, 133.4, 125.8, 125.6, 122.0, 113.1, 90.9, 56.0, 55.6, 55.2, 52.1, 51.1, 41.5, 29.8, 24.9, 22.9, 22.2. IR (cm^{-1}): 2953, 1742, 1672, 1604, 1588, 1518, 1434, 1228, 1206, 1150, 1127. HRMS *caldc* for ($C_{42}H_{47}N_3O_{12}$): 785.3160, *found,* 785.3186.

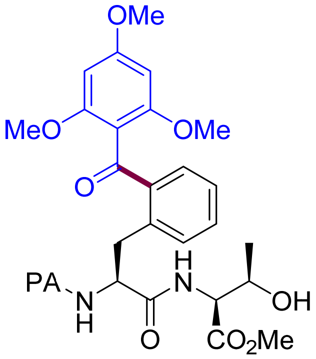

(5f)

Methyl [(*S*)-2-(picolinamido)-3-(2-(2,4,6-trimethoxybenzoyl)phenyl)propanoyl]-*L*-threoninate (5f). Following the general procedure, using commercially available 2,4,6-trimethoxybenzaldehyde (1.25 mmol, 245.0 mg) and PA-Phe-Thr-OMe (0.25 mmol, 96.3 mg) provided 46 mg (31% yield) of **5f** as a colorless oil. ^{1}H NMR (400 MHz, $CDCl_3$) δ 8.98 (d, J = 7.6 Hz, 1H), 8.56 (d, J = 4.8 Hz, 1H), 8.06 (d, J = 7.7 Hz, 1H), 7.77 (td, J = 7.7, 1.7 Hz, 1H), 7.46 (d, J = 7.2 Hz, 1H), 7.38 (ddd, J = 7.7, 4.8, 1.2 Hz, 1H), 7.35 – 7.29 (m, 3H), 7.21 – 7.13 (m, 1H), 6.14 (s, 2H), 5.07 – 4.96 (m, 1H), 4.59 (dd, J = 8.8, 3.2 Hz, 1H), 4.35 – 4.21 (m, 1H), 3.85 (s, 3H), 3.81 – 3.72 (m, 1H), 3.70 (s, 3H), 3.68 (s, 6H), 3.43 (dd, J = 13.4, 9.7 Hz, 1H), 2.63 (brs, 1H), 1.20 (d, J = 6.4 Hz, 3H). ^{13}C NMR (101 MHz, $CDCl_3$) δ 197.9, 172.2, 171.1, 165.0, 162.8, 159.1, 149.6, 148.4, 138.8, 138.1, 137.2, 132.0, 131.9, 131.9, 126.9, 126.3, 122.3, 112.7, 90.9, 68.6, 57.9, 56.1, 55.6, 55.54, 52.6, 36.6, 20.1. IR (cm^{-1}): 3349, 2945, 1742, 1663,1604, 1589,1516, 1227, 1205, 1127. HRMS *caldc* for ($C_{30}H_{33}N_3O_9$): 579.2217, *found* 579.2213.

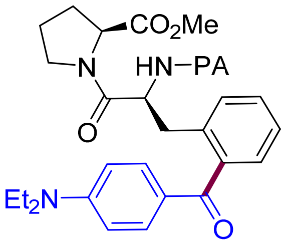

(5g)

Methyl [(*S*)-3-(2-(4-(diethylamino)benzoyl)phenyl)-2-(picolinamido)propanoyl]-*L*-prolinate (5g). Following the general procedure, using commercially available 4-diethylaminobenzaldehyde (1.25 mmol, 222 mg) and PA-Phe-Pro-OMe (0.25 mmol, 95.0 mg) provided 100.0 mg (62% yield) (1:1 ratio mono:di) of **5g** as a colorless oil. *Both isomers were separated and independently characterized. Monofunctionalized* ***5g:***

^{1}H NMR (400 MHz, $CDCl_3$) δ 8.75 (d, *J* = 8.7 Hz, 1H), 8.54 (d, *J* = 4.5 Hz, 1H), 8.00 (d, *J* = 7.8 Hz, 1H), 7.85 – 7.70 (m, 3H), 7.44 – 7.20 (m, 5H), 6.60 (d, *J* = 9.1 Hz, 2H), 5.27 – 5.08 (m, 1H), 4.48 (dd, *J* = 8.2, 4.3 Hz, 1H), 3.73 (s, 3H), 3.68 – 3.61 (m, 1H), 3.54 – 3.37 (m, 6H), 3.01 (dd, *J* = 13.8, 9.8 Hz, 1H), 2.23 – 2.11 (m, 1H), 2.05 – 1.88 (m, 3H), 1.22 (t, *J* = 7.0 Hz, 6H). ^{13}C NMR (101 MHz, $CDCl_3$) δ 196.1, 172.5, 170.5, 163.9, 151.6, 149.7, 148.3, 140.5, 137.0, 135.1, 133.4, 131.7, 129.3, 128.3, 126.1, 126.0, 124.7, 122.1, 110.1, 59.2, 52.4, 52.3, 46.8, 44.7, 35.9, 29.2, 25.1, 12.7. IR (cm^{-1}): 2973, 1744, 1643, 1585, 1515, 1430, 1269, 1194, 1146. HRMS *caldc* for ($C_{32}H_{36}N_4O_5$): 556.2686, *found* 556.2701. *Difunctionalized* ***5g'***: ^{1}H NMR (400 MHz, $CDCl_3$) δ 9.25 (d, *J* = 8.1 Hz, 1H), 8.44 (d, *J* = 4.4 Hz, 1H), 7.92 (d, *J* = 7.7 Hz, 1H), 7.76 (d, *J* = 8.9 Hz, 4H), 7.67 (td, *J* = 7.7, 1.7 Hz, 1H), 7.40 – 7.30 (m, 3H), 7.27 – 7.21 (m, 1H), 6.54 (d, *J* = 8.9 Hz, 4H), 4.93 – 4.82 (m, 1H), 4.42 (dd, *J* = 8.2, 4.8 Hz, 1H), 3.64 (s, 3H), 3.52 – 3.33 (m, 12H), 2.24 – 2.04 (m, 1H), 2.04 – 1.80 (m, 3H), 1.23 (t, *J* = 7.1 Hz, 12H). ^{13}C NMR (101 MHz, $CDCl_3$) δ 195.6, 172.6, 170.5, 165.0, 151.7, 150.2, 148.5, 142.0, 136.3, 134.5, 133.6, 129.4, 125.4, 124.9, 124.4, 121.9, 110.1, 59.1, 53.1, 52.2, 46.2, 44.7, 30.2, 29.1, 25.2, 12.7. IR (cm^{-1}): 2973, 1746, 1649, 1586, 1523, 1431, 1409, 1267, 1186, 1149. HRMS *caldc* for ($C_{43}H_{49}N_5O_6$): 731.3683, *found* 731.3697.

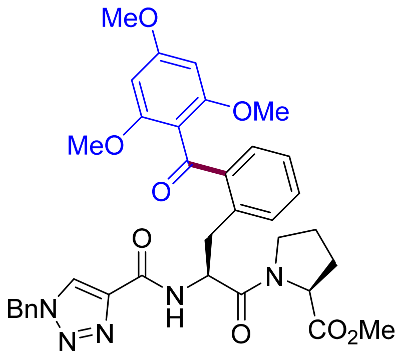

(5h)

Methyl [(*S*)-2-(1-benzyl-1*H*-1,2,3-triazole-4-carboxamido)-3-(2-(2,4,6-trimethoxybenzoyl)phenyl)propanoyl]-*L*-prolinate (5h). Following the general procedure, using commercially available 2,4,6-trimethoxybenzaldehyde (1.25 mmol, 245.0 mg) and **4e** (0.25 mmol, 115.3 mg) provided 64.0 mg (39% yield) of **5h** as a colorless oil. ^{1}H NMR (400 MHz, $CDCl_3$) δ 7.82 – 7.74 (m, 2H), 7.46 (d, *J* = 7.9 Hz, 1H), 7.43 – 7.34 (m, 4H), 7.33 – 7.14 (m, 4H), 6.15 (s, 2H), 5.58 – 5.45 (m, 2H), 5.36 – 5.25 (m, 1H), 4.59 – 4.51 (m, 1H), 4.02 – 3.92 (m, 2H), 3.86 (s, 3H), 3.75 (s, 3H), 3.70 – 3.69 (m, 1H), 3.69 (s, 6H), 3.12 (dd, *J* = 13.3, 10.3 Hz, 1H), 2.29 – 2.15 (m, 1H), 2.06 – 1.92 (m, 3H). ^{13}C NMR (101 MHz, $CDCl_3$) δ 197.5, 172.6, 170.8, 162.6, 159.7, 158.8, 143.3, 138.7, 137.3, 134.0, 132.9, 131.9, 131.8, 129.3, 129.1, 128.3, 126.9, 125.2, 90.8, 59.2, 55.9, 55.6, 54.5, 52.3, 51.8, 47.1, 36.4, 29.2, 25.3. IR (cm^{-1}): 2950,

1742, 1645, 1603, 1587, 1570, 1452, 1436, 1226, 1204, 1126. HRMS *caldc* for ($C_{35}H_{37}N_5O_8$): 655.2642, *found* 655.2654.

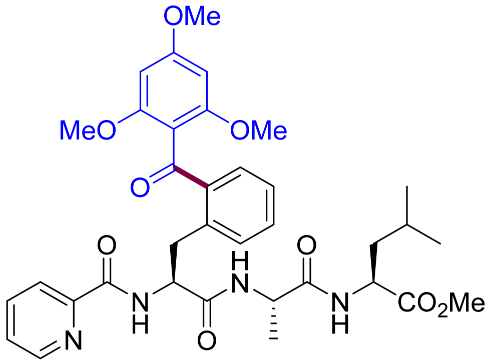

(5i)

Methyl [(*S*)-2-(picolinamido)-3-(2-(2,4,6-trimethoxybenzoyl)phenyl)propanoyl]-*L*-alanyl-*L*-leucinate (5i). Following the general procedure, using commercially available 2,4,6-trimethoxybenzaldehyde (0.75 mmol, 147.0 mg) and PA-Phe-Ala-Leu-OMe (0.15 mmol, 70.0 mg) provided 48.0 mg (48% yield) of **5i** as a yellowish oil. ^{1}H NMR (400 MHz, $CDCl_3$) δ 9.23 (d, *J* = 7.1 Hz, 1H), 8.58 (d, *J* = 4.3 Hz, 1H), 8.04 (d, *J* = 7.8 Hz, 1H), 7.78 (td, *J* = 7.7, 1.7 Hz, 1H), 7.46 (d, *J* = 7.7 Hz, 1H), 7.40 (ddd, *J* = 7.6, 4.8, 1.2 Hz, 1H), 7.37 – 7.32 (m, 2H), 7.22 – 7.15 (m, 1H), 7.00 (d, *J* = 8.2 Hz, 1H), 6.91 (d, *J* = 7.8 Hz, 1H), 6.14 (s, 2H), 4.96 – 4.85 (m, 1H), 4.61 – 4.47 (m, 2H), 3.86 (s, 3H), 3.77 – 3.73 (m, 1H), 3.70 (s, 3H), 3.68 (s, 6H), 3.28 (dd, *J* = 13.5, 10.2 Hz, 1H), 1.66 – 1.53 (m, 3H), 1.39 (d, *J* = 7.1 Hz, 3H), 0.86 (dd, *J* = 8.3, 5.6 Hz, 6H). ^{13}C NMR (101 MHz, $CDCl_3$) δ 198.2, 173.2, 172.0, 171.9, 165.4, 162.9, 159.1, 149.6, 148.5, 138.8, 138.3, 137.2, 132.0, 131.8, 127.0, 126.4, 122.4, 112.5, 90.9, 56.1, 55.6, 52.3, 51.0, 49.2 41.1, 36.7, 24.9, 22.9, 21.9, 17.3. IR (cm^{-1}): 3301, 2955, 1740, 1655, 1603, 1588, 1514, 1226, 1205, 1127. HRMS *caldc* for ($C_{35}H_{42}N_4O_9$): 662.2952, *found* 662.2957.

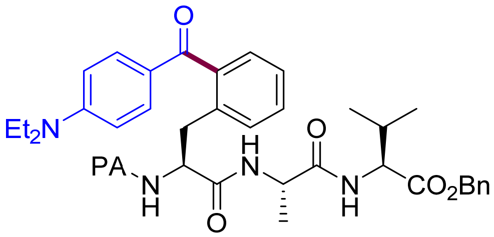

(5j)

Benzyl [(*S*)-3-(2-(4-(diethylamino)benzoyl)phenyl)-2-(picolinamido)propanoyl]-*L*-alanyl-*L*-valinate (5j). Following the general procedure, using commercially available 4-diethylaminobenzaldehyde (0.75 mmol, 133 mg) and PA-Phe-Ala-Val-OBn (0.15 mmol, 80.0 mg) provided 34.0 mg (32% yield) of **5j** as a colorless oil. ^{1}H NMR (400 MHz, $CDCl_3$) δ 9.60 (d, *J* = 6.6 Hz, 1H), 8.62 (d, *J* = 4.6 Hz, 1H), 8.05 (d, *J* = 7.8 Hz, 1H), 7.84 – 7.72 (m, 3H), 7.51 – 7.31 (m, 9H), 7.26 (td, *J* = 7.3, 6.9, 1.3 Hz, 1H), 6.97 (dd, *J* = 16.3, 8.1 Hz, 2H), 6.63 (d, *J* = 9.1 Hz, 2H), 5.18 (q, *J* = 12.0 Hz, 2H),

4.92 – 4.83 (m, 1H), 4.57 – 4.47 (m, 2H), 3.46 (q, *J* = 7.1 Hz, 4H), 3.33 (dd, *J* = 13.9, 4.2 Hz, 1H), 3.17 (dd, *J* = 13.9, 10.3 Hz, 1H), 2.26 – 2.14 (m, 1H), 1.36 (d, *J* = 7.1 Hz, 3H), 1.24 (t, *J* = 7.0 Hz, 6H), 0.88 (dd, *J* = 11.3, 6.8 Hz, 6H). ^{13}C NMR (101 MHz, $CDCl_3$) δ 196.3, 172.0, 171.5, 165.9, 151.8, 149.7, 148.6, 139.5, 137.1, 136.7, 135.7, 133.7, 130.8, 130.2, 129.4, 128.7, 128.5, 128.5, 128.4, 126.3, 126.0, 124.4, 122.5, 110.2, 66.9, 57.6, 56.5, 49.2, 44.8, 35.2, 31.1, 19.2, 18.0, 17.4, 12.7. IR (cm^{-1}): 3310, 2970, 1739, 1652, 1587, 1522, 1375, 1269, 1216, 1197, 1147. HRMS *caldc* for ($C_{41}H_{47}N_5O_6$): 705.3526, *found* 705.3541.

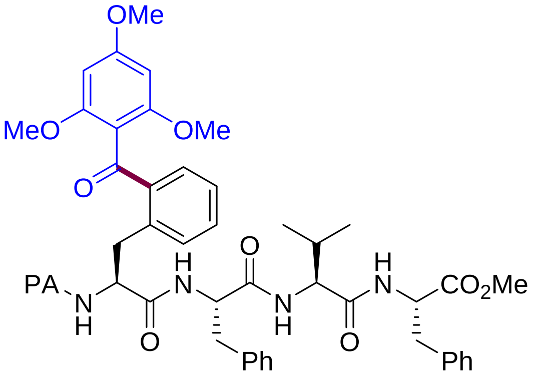

(5k)

Methyl [(*S*)-2-(picolinamido)-3-(2-(2,4,6-trimethoxybenzoyl)phenyl)propanoyl]-*L*-phenylalanyl-*L*-valyl-*L*-phenylalaninate (5k). Following the general procedure, using commercially available 2,4,6-trimethoxybenzaldehyde (0.75 mmol, 147.0 mg) and PA-Phe-Phe-Val-Phe-OMe (0.15 mmol, 102.0 mg) provided 73.0 mg (52% yield) (3:1 ratio) of **5k** as a colorless oil. *Both isomers were separated and the following data correspond to the monofunctionalized* ***5k**:* ^{1}H NMR (400 MHz, $CDCl_3$) δ 9.68 (d, *J* = 5.8 Hz, 1H), 8.64 (d, *J* = 4.4 Hz, 1H), 7.89 (d, *J* = 7.7 Hz, 1H), 7.80 (td, *J* = 7.6, 1.7 Hz, 1H), 7.51 – 7.42 (m, 2H), 7.41 – 7.34 (m, 2H), 7.33 – 7.18 (m, 6H), 7.08 (d, *J* = 6.9 Hz, 2H), 7.01 – 6.84 (m, 6H), 6.18 (s, 2H), 4.91 – 4.82 (m, 1H), 4.78 – 4.63 (m, 2H), 4.26 (dd, *J* = 8.4, 6.1 Hz, 1H), 3.90 (s, 3H), 3.74 (s, 3H), 3.71 (s, 6H), 3.60 (dd, *J* = 13.6, 3.6 Hz, 1H), 3.40 – 3.10 (m, 4H), 3.01 (dd, *J* = 14.0, 5.6 Hz, 1H), 2.28 – 2.10 (m, 1H), 0.74 (dd, *J* = 44.8, 6.9 Hz, 6H). ^{13}C NMR (101 MHz, $CDCl_3$) δ 198.3, 172.1, 172.0, 170.9, 170.9, 165.9, 163.0, 159.2, 149.2, 148.5, 139.5, 137.9, 137.1, 136.7, 136.0, 131.9, 131.6, 131.4, 129.4, 129.3, 128.6, 128.6, 126.9, 126.9, 126.8, 126.4, 122.4, 112.4, 90.9, 59.4, 57.3, 56.0, 55.6, 54.6, 53.5, 52.4, 37.9, 36.7, 35.0, 29.6, 19.4, 17.6. IR (cm^{-1}): 3302, 2995, 1646, 1604, 1517, 1226, 1205, 1128. HRMS *caldc* for ($C_{49}H_{53}N_5O_{10}$): 871.3792, *found* 871.3802.

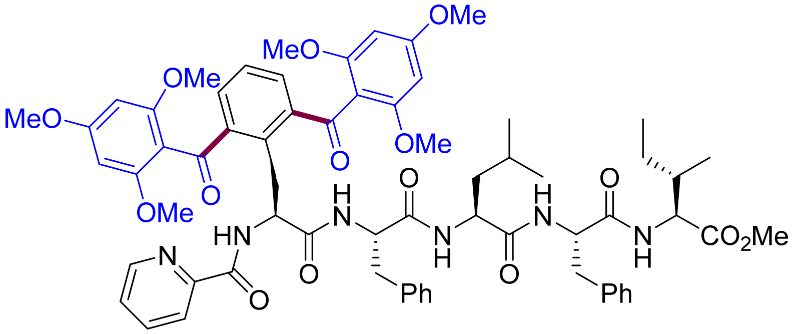

(5l')

Methyl [(*S*)-3-(2,6-bis(2,4,6-trimethoxybenzoyl)phenyl)-2-(picolinamido) propanoyl]-*L*-phenylalanyl-*L*-leucyl-*L*-phenylalanyl-*L*-isoleucinate (5l'). Following the general procedure, using commercially available 2,4,6-trimethoxybenzaldehyde (0.75 mmol, 147.0 mg) and PA-Phe-Phe-Leu-Phe-Ile-OMe (0.15 mmol, 121.0 mg) provided 50.0 mg (31% yield) of **5l'** as a colorless oil. ^{1}H NMR (400 MHz, $CDCl_3$) δ 10.20 (d, *J* = 4.7 Hz, 1H), 8.66 (d, *J* = 4.5 Hz, 1H), 7.81 – 7.71 (m, 2H), 7.47 – 7.44 (m, 1H), 7.43 (d, *J* = 4.0 Hz, 2H), 7.35 (d, *J* = 9.2 Hz, 1H), 7.21 – 7.14 (m, 4H), 7.14 – 7.04 (m, 3H), 7.03 – 6.93 (m, 4H), 6.83 – 6.71 (m, 3H), 6.01 (s, 4H), 4.95 – 4.85 (m, 1H), 4.70 – 4.61 (m, 1H), 4.54 – 4.37 (m, 2H), 4.30 – 4.19 (m, 1H), 3.79 (s, 6H), 3.71 (s, 3H), 3.56 (s, 12H), 3.47 (dd, *J* = 13.8, 11.8 Hz, 2H), 3.32 (ddd, *J* = 17.1, 12.5, 4.9 Hz, 2H), 3.03 – 2.93 (m, 1H), 2.90 – 2.79 (m, 1H), 2.03 – 1.90 (m, 1H), 1.62 – 1.50 (m, 1H), 1.43 – 1.14 (m, 4H), 0.97 (d, *J* = 6.8 Hz, 3H), 0.92 (t, *J* = 7.4 Hz, 3H), 0.79 (t, *J* = 6.6 Hz, 6H). ^{13}C NMR (101 MHz, $CDCl_3$) δ 197.1, 173.4, 172.5, 172.1, 171.4, 171.3, 166.5, 163.3, 159.6, 149.4, 148.8, 142.2, 138.4, 136.9, 136.2, 135.6, 132.3, 129.6, 129.2, 128.5, 128.0, 126.8, 126.4, 126.1, 125.7, 122.2, 112.4, 91.0, 57.2, 57.0, 56.0, 55.6, 55.6, 55.2, 53.1, 52.0, 39.4, 37.8, 37.6, 36.0, 28.9, 25.3, 24.3, 23.4, 21.0, 15.5, 11.8. IR (cm^{-1}): 3289, 2956, 1739, 1661, 1603, 1520, 1455, 1365, 1227, 1217, 1205, 1127. HRMS *caldc* for ($C_{66}H_{76}N_6O_{15}$): 1192.5369, *found* 1192.5391.

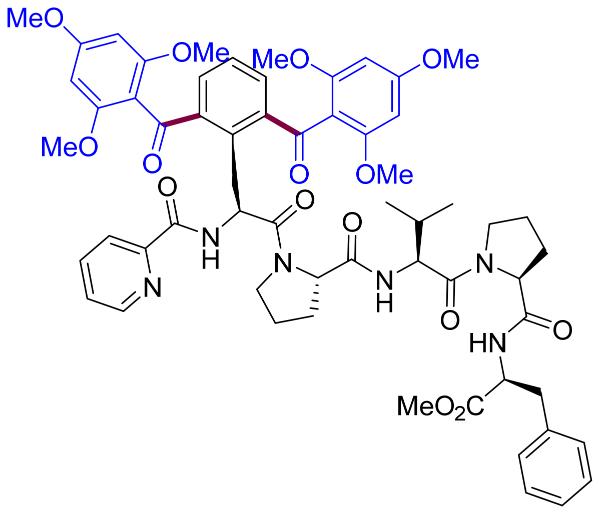

(5m')

Methyl [(*S*)-3-(2,6-bis(2,4,6-trimethoxybenzoyl)phenyl)-2-(picolinamido) propanoyl]-*L*-prolyl-*L*-valyl-*L*-prolyl-*L*-phenylalaninate (5m'). Following the general procedure, using commercially available 2,4,6-trimethoxybenzaldehyde (0.75 mmol, 147.0 mg) and PA-Phe-Pro-Val-Pro-Phe-OMe (0.15 mmol, 109.0 mg) provided 123.0 mg (70% yield) (13:87 ratio) of **5m'** as a colorless oil. *Both isomers were separated and the following data correspond to the difunctionalized* ***5m'****:* ^{1}H NMR (400 MHz, $CDCl_3$) δ 9.88 (d, J = 7.2 Hz, 1H), 8.64 (d, J = 4.8 Hz, 1H), 8.02 (d, J = 7.8 Hz, 1H), 7.84 – 7.68 (m, 2H), 7.49 (d, J = 7.8 Hz, 2H), 7.41 – 7.08 (m, 8H), 6.14 (s, 4H), 5.18 (t, J = 8.2 Hz, 1H), 4.74 (d, J = 6.7 Hz, 2H), 4.61 – 4.53 (m, 1H), 4.37 (t, J = 8.1 Hz, 1H), 4.21 (d, J = 13.8 Hz, 1H), 4.13 – 3.94 (m, 3H), 3.86 (s, 6H), 3.68 (s, 15H), 3.56 – 3.33 (m, 2H), 3.17 – 3.01 (m, 2H), 2.40 – 2.20 (m, 2H), 2.20 – 2.06 (m, 1H), 2.06 – 1.86 (m, 6H), 0.85 (dd, J = 11.6, 6.7 Hz, 6H). ^{13}C NMR (101 MHz, $CDCl_3$) δ 197.6, 173.3, 172.0, 171.8, 171.5, 171.3, 165.6, 163.2, 159.4, 150.5, 148.7, 136.9, 136.7, 136.1, 133.0, 129.4, 128.6, 128.4, 127.1, 126.0, 125.7, 122.0, 112.9, 90.9, 60.0, 56.4, 56.1, 55.6, 53.8, 53.4, 52.3, 47.7, 47.2, 38.0, 30.9, 29.4, 27.3, 27.1, 25.6, 25.1, 19.5, 18.3. IR (cm^{-1}): 3304, 2964, 1745, 1665, 1603, 1518, 1454, 1432, 1226, 1205, 1151, 1127. HRMS *caldc* for ($C_{60}H_{68}N_6O_{15}$): 1112.4743, *found* 1112.4765.

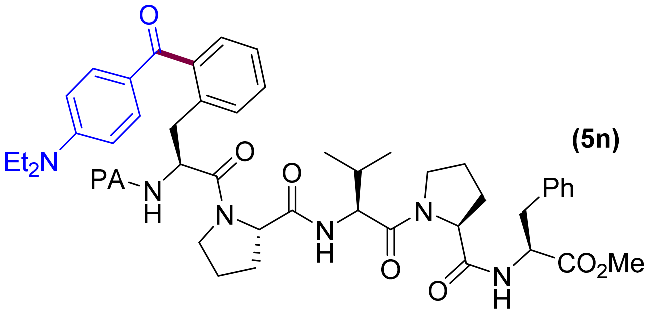

(5n)

Methyl [(*S*)-3-(2-(4-(diethylamino)benzoyl)phenyl)-2-(picolinamido)propanoyl]-*L*-prolyl-*L*-valyl-*L*-prolyl-*L*-phenylalaninate (5n). Following the general procedure, using commercially available 4-diethylaminobenzaldehyde (0.75 mmol, 133 mg) and

PA-Phe-Pro-Val-Pro-Phe-OMe (0.15 mmol, 109.0 mg) provided 113.0 mg (78% yield) (6:4 ratio) of **5n** as a colorless oil. *Both isomers were separated and independently characterized. Monofunctionalized **5n**:* ^{1}H NMR (400 MHz, $CDCl_3$) δ 8.78 (d, *J* = 8.6 Hz, 1H), 8.63 – 8.54 (m, 1H), 8.00 (d, *J* = 7.8 Hz, 1H), 7.82 – 7.71 (m, 3H), 7.41 – 7.24 (m, 10H), 7.16 (d, *J* = 8.2 Hz, 2H), 6.60 (d, *J* = 9.1 Hz, 2H), 5.26 – 5.07 (m, 1H), 4.77 (q, *J* = 6.4 Hz, 1H), 4.59 (dd, *J* = 8.1, 2.7 Hz, 2H), 4.51 – 4.40 (m, 1H), 3.83 – 3.73 (m, 1H), 3.69 (s, 3H), 3.63 – 3.49 (m, 2H), 3.48 – 3.39 (m, 5H), 3.30 – 2.96 (m, 4H), 2.36 – 2.23 (m, 2H), 2.12 – 1.79 (m, 7H), 1.22 (t, *J* = 7.0 Hz, 6H), 0.88 (d, *J* = 6.7 Hz, 6H).^{13}C NMR (101 MHz, $CDCl_3$) δ 195.9, 171.8, 171.7, 171.1, 171.0, 170.8, 164.0, 151.5, 149.5, 148.3, 148.3, 140.2, 136.9, 136.0, 135.0, 133.3, 131.3, 129.2, 128.5, 128.4, 127.0, 126.1, 126.0, 124.4, 122.0, 110.0, 60.0, 59.9, 55.9, 53.7, 52.4, 52.2, 47.5, 47.0, 44.5, 37.9, 35.9, 31.1, 27.3, 27.1, 25.1, 25.0, 19.4, 17.9, 12.5. IR (cm^{-1}): 2970, 1739, 1637, 1588, 1523, 1433, 1385, 1216, 1204. HRMS *caldc* for ($C_{51}H_{61}N_7O_8$): 899.4582, *found* 899.4538. *Difunctionalized **5n'***: ^{1}H NMR (400 MHz, $CDCl_3$) δ 9.21 (d, *J* = 8.0 Hz, 1H), 8.45 (d, *J* = 4.8 Hz, 1H), 7.93 (d, *J* = 7.7 Hz, 1H), 7.74 (d, *J* = 9.1 Hz, 4H), 7.69 (td, *J* = 7.7, 1.8 Hz, 1H), 7.53 (d, *J* = 8.0 Hz, 1H), 7.42 – 7.36 (m, 2H), 7.34 – 7.24 (m, 6H), 7.15 (d, *J* = 6.8 Hz, 2H), 6.52 (d, *J* = 9.0 Hz, 4H), 4.88 (q, *J* = 7.6 Hz, 1H), 4.72 (q, *J* = 6.5 Hz, 1H), 4.59 – 4.50 (m, 2H), 4.28 (t, *J* = 7.7 Hz, 1H), 3.76 – 3.70 (m, 2H), 3.67 (s, 3H), 3.63 – 3.56 (m, 1H), 3.41 (q, *J* = 7.2 Hz, 13H), 3.05 (t, *J* = 6.4 Hz, 1H), 2.30 – 2.23 (m, 2H), 2.05 – 1.81 (m, 7H), 1.22 (t, *J* = 7.1 Hz, 12H), 0.72 (dd, *J* = 6.8, 3.6 Hz, 6H).^{13}C NMR (101 MHz, $CDCl_3$) δ 195.6, 172.2, 171.9, 171.8, 171.3, 171.1, 165.0, 151.7, 150.0, 148.5, 141.8, 136.4, 136.2, 134.3, 133.5, 129.6, 129.4, 128.6, 127.1, 125.5, 125.1, 124.3, 121.9, 110.1, 59.9, 59.9, 56.3, 54.0, 53.0, 52.3, 47.60, 46.6, 44.7, 38.0, 30.9, 30.7, 27.3, 26.7, 25.4, 25.2, 19.4, 18.0, 12.7.IR (cm^{-1}): 3302, 2970, 1740, 1637, 1585, 1523, 1432, 1355, 1266, 1185, 1148. HRMS *caldc* for ($C_{62}H_{74}N_8O_9$): 1074.5579, *found* 1074.5565.

5.-Deprotection of 3i'

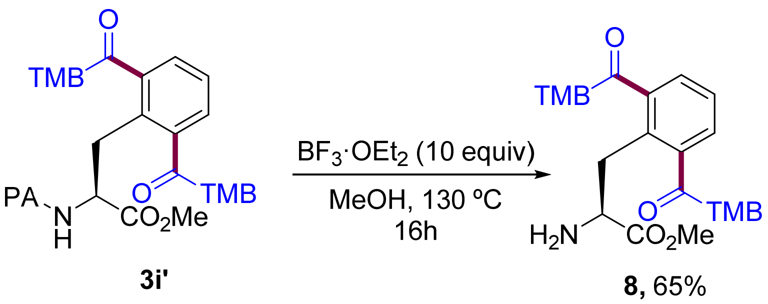

Methyl (*S*)-2-amino-3-[2,6-bis(2,4,6-trimethoxybenzoyl)phenyl]propanoate (8). Picolinamide **3i'** (0.193 mmol, 130 mg) was dissolved in dry MeOH (3.5 mL) under argon. Then $BF_3 \cdot OEt_2$ (4.3 mmol, 0.54 mL) was added and the system was sealed under argon. The reaction was stirred at 130 ºC for 16h. The resulting solution was evaporated and purified by column chromatography (hexanes:EtOAc 7:3) to provide 71.5 mg (65% yield) of **8** as a white solid. Mp 88-90 ºC ^{1}H NMR (400 MHz, $CDCl_3$) δ 7.46 (dd, *J* = 7.1, 2.1 Hz, 1H), 7.16 – 7.01 (m, 2H), 6.20 – 6.08 (m, 4H), 4.40 (dd, *J* = 13.6, 5.4 Hz, 1H), 3.84 (s, 3H), 3.83 (s, 3H), 3.78 (s, 3H), 3.73 (s, 3H), 3.69 – 3.61 (m, 12H), 3.24 (dd, *J* = 17.1, 13.6 Hz, 1H). ^{13}C NMR (101 MHz, $CDCl_3$) δ 196.3, 173.4, 164.2, 162.9, 161.8, 159.2, 159.1, 138.1, 136.3, 132.7, 130.8, 130.1, 126.2, 112.5, 91.0, 90.9, 60.6, 56.0, 55.6, 55.5, 52.4, 25.9. IR (neat, cm^{-1}): 3205, 1738, 1667, 1120. HRMS *calcd.* for ($C_{30}H_{33}NO_{10}$): 567.2104, *found* 567.21.21.

6.-Pd-catalyzed C(sp^2)–H Dihalogenation of Phe Derivatives

Table S9. Bromination of 1a

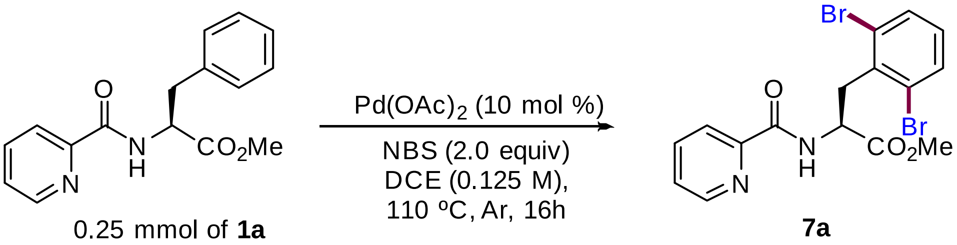

Entry	Variation from standard conditions	Yield (%)[a]
**1**	none	85
**2**	without $Pd(OAc)_2$	0
**3**	reaction at 80 ºC	75
**4**	under air	78
**5**	$RuCl_2(PPh_3)_2$ instead of $Pd(OAc)_2$	0
**6**	$Ru_3(CO)_{12}$ instead of $Pd(OAc)_2$	0
**7**[b]	$[RuCl(p\text{-cymene})]_2$ instead of $Pd(OAc)_2$	0

[a] Yield obtained after purification by column chromatography (Hexanes: EtOAc 7:3).

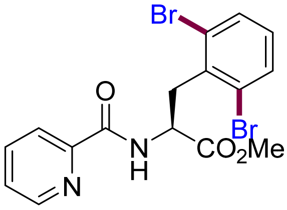

(7a)

Methyl (*S*)-3-(2,6-dibromophenyl)-2-(picolinamido)propanoate (7a). A reaction tube containing a stirring bar was charged with **1a** (0.25 mmol, 71 mg), NBS (0.50 mmol, 89 mg) and $Pd(OAc)_2$ (5 mol %, 5.5 mg). The reaction tube was then evacuated and back-filled with dry argon (this sequence was repeated up to three times). Then, DCE (2 mL) was added under argon atmosphere and the reaction tube was next warmed up to 110 ºC and stirred for 16 hours. The mixture was then allowed to warm to room temperature, concentrated under reduced pressure and the corresponding product was purified by flash chromatography (Hex:EtOAc 7:3) to provide 93 mg (85% yield) of **7a** as an orange oil. ^{1}H NMR (400 MHz, $CDCl_3$) δ 8.72 (d, J = 9.0 Hz, 1H), 8.58 (dt, J = 4.7, 1.3 Hz, 1H), 8.06 (dt, J = 7.8, 1.1 Hz, 1H), 7.78 (td, J = 7.7, 1.7 Hz, 1H), 7.47 (d, J = 8.0 Hz, 2H), 7.40 (ddd, J = 7.6, 4.8, 1.2 Hz, 1H), 6.91 (t, J = 8.0 Hz, 1H), 5.29 (m, 1H), 3.75 (s, 3H), 3.67 – 3.51 (m, 2H). ^{13}C NMR (101 MHz, $CDCl_3$) δ 171.6, 164.1, 149.2, 148.3, 137.3, 135.7, 132.4, 129.7, 126.4, 126.2, 122.3, 52.8, 51.2, 39.1. IR (neat, cm^{-1}): 3320, 1739, 1674, 1509, 1429. HRMS *calcd.* for ($C_{16}H_{14}Br_2N_2O_3$): 439.9371, *found* 439.9378.

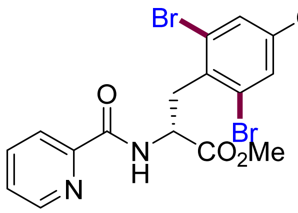

(7b)

Methyl (*R*)-3-(2,6-dibromo-4-methoxyphenyl)-2-(picolinamido)propanoate (7b). Following the general procedure, using picolinamide **1c** (0.25 mmol, 79 mg) provided 99 mg (84% yield) of **7b** as a white solid. Mp 100-101 ºC. ^{1}H NMR (400 MHz, $CDCl_3$) δ 8.69 (d, *J* = 8.9 Hz, 1H), 8.64 – 8.54 (m, 1H), 8.07 (dt, *J* = 7.9, 1.1 Hz, 1H), 7.79 (td, *J* = 7.7, 1.7 Hz, 1H), 7.41 (ddd, *J* = 7.7, 4.8, 1.3 Hz, 1H), 7.05 (s, 2H), 5.23 (m, 1H), 3.75 (s, 3H), 3.73 (s, 3H), 3.62 – 3.42 (m, 2H). ^{13}C NMR (101 MHz, $CDCl_3$) δ 171.6, 164.0, 158.8, 149.1, 148.1, 137.0, 127.3, 126.2, 125.7, 122.1, 118.1, 55.5, 52.6, 51.3, 38.0. IR (neat, cm^{-1}): 3374, 1739, 1672, 1591, 1432, 1252. HRMS *calcd.* for ($C_{17}H_{16}Br_2N_2O_4$): 469.9477, *found* 469.9482.

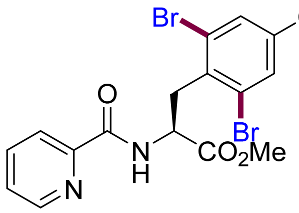

(7c)

Methyl (*S*)-3-(2,6-dibromo-4-chlorophenyl)-2-(picolinamido)propanoate (7c). Following the general procedure, using picolinamide **1d** (0.25 mmol, 79 mg) provided 80 mg (67% yield) of **7c** as a white solid. Mp 153-154 ºC. ^{1}H NMR (400 MHz, $CDCl_3$) δ 8.70 (d, *J* = 9.1 Hz, 1H), 8.58 (ddd, *J* = 4.9, 1.7, 0.9 Hz, 1H), 8.07 (dt, *J* = 7.9, 1.0 Hz, 1H), 7.80 (td, *J* = 7.7, 1.7 Hz, 1H), 7.50 (s, 1H), 7.42 (ddd, *J* = 7.6, 4.8, 1.2 Hz, 1H), 5.27 (m, 1H), 3.76 (s, 3H), 3.64 – 3.46 (m, 2H). ^{13}C NMR (101 MHz, $CDCl_3$) δ 171.4, 164.1, 149.1, 148.3, 137.4, 134.5, 134.1, 132.1, 126.5, 126.0, 122.4, 52.9, 51.0, 38.7. IR (neat, cm^{-1}): 3369, 1735, 1679, 1505, 1362. HRMS *calcd.* for ($C_{16}H_{13}Br_2ClN_2O_3$): 473.8981, *found* 473.8978.

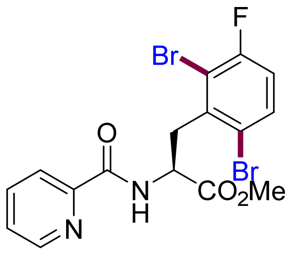

(7d)

Methyl (*S*)-3-(2,6-dibromo-3-fluorophenyl)-2-(picolinamido)propanoate (7d). Following the general procedure, using picolinamide **1e** (0.25 mmol, 76 mg) provided 92.5 mg (80% yield) of **7d** as a colorless oil. ^{1}H NMR (400 MHz, $CDCl_3$) δ 8.74 (d, *J* = 9.0 Hz, 1H), 8.61 (ddd, *J* = 4.9, 1.7, 0.9 Hz, 1H), 8.09 (dt, *J* = 7.8, 1.1 Hz, 1H), 7.82

(td, J = 7.7, 1.7 Hz, 1H), 7.55 – 7.38 (m, 2H), 6.92 (dd, J = 8.8, 7.6 Hz, 1H), 5.35 – 5.28 (m, 1H), 3.79 (s, 3H), 3.67 (d, J = 8.0 Hz, 3H). ^{13}C NMR (101 MHz, $CDCl_3$) δ 171.5, 164.2, 158.6 (d, $J_{C\text{-}F}$ = 248.5 Hz), 149.2, 148.4, 137.9, 137.3, 132.7 (d, $J_{C\text{-}F}$ = 7.1 Hz), 126.5, 122.3, 120.1 (d, $J_{C\text{-}F}$ = 3.0 Hz), 116.2 (d, $J_{C\text{-}F}$ = 24.2 Hz), 113.7 (d, $J_{C\text{-}F}$ = 22.2 Hz), 52.9, 51.1, 39.1. IR (neat, cm^{-1}): 3330, 1740, 1674, 1509, 1444. MS (ESI$^+$) m/z (%) 458 (M+H). HRMS *calcd.* for ($C_{16}H_{14}Br_2FN_2O_3$): 458.9277, *found* 459.9358.

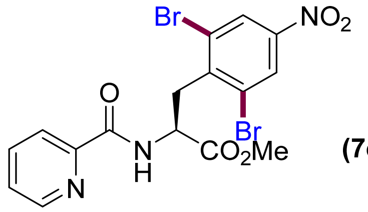

(7e)

Methyl (*S*)-3-(2,6-dibromo-4-nitrophenyl)-2-(picolinamido)propanoate (7e). Following the general procedure, using picolinamide **1f** (0.25 mmol, 82 mg) provided 74 mg (61% yield) of **7e** as a white solid. Mp 181-182 ºC. ^{1}H NMR (400 MHz, $CDCl_3$) δ 8.73 (d, J = 9.1 Hz, 1H), 8.59 (ddd, J = 4.8, 1.6, 0.8 Hz, 1H), 8.34 (s, 1H), 8.03 (dt, J = 7.8, 1.0 Hz, 1H), 7.81 (td, J = 7.7, 1.7 Hz, 1H), 7.44 (ddd, J = 7.6, 4.8, 1.2 Hz, 1H), 5.36 (m, 1H), 3.79 (s, 3H), 3.70 (d, J = 7.9 Hz, 2H). ^{13}C NMR (101 MHz, $CDCl_3$) δ 171.0, 164.1, 148.9, 148.4, 146.9, 143.4, 137.4, 127.1, 126.6, 126.3, 122.4, 53.1, 50.6, 39.6. IR (neat, cm^{-1}): 3230, 1746, 1670, 1511, 1151. HRMS *calcd.* for ($C_{16}H_{13}Br_2N_3O_5$): 484.9222, *found* 484.9227.

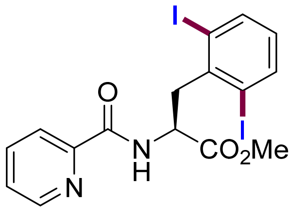

(7f)

Methyl (*S*)-3-(2,6-diiodophenyl)-2-(picolinamido)propanoate (7f). Following the general procedure, using picolinamide **1a** (0.25 mmol, 71 mg) and NIS (0.50 mmol, 112 mg) provided 117 mg (87% yield) of **7f** as a colorless oil. ^{1}H NMR (400 MHz, $CDCl_3$) δ 8.73 (d, J = 9.2 Hz, 1H), 8.60 (dt, J = 4.5, 1.8 Hz, 1H), 8.05 (d, J = 7.7 Hz, 1H), 7.79 (dddd, J = 7.2, 5.2, 3.9, 1.8 Hz, 3H), 7.49 – 7.33 (m, 1H), 6.50 (td, J = 7.7, 1.9 Hz, 1H), 5.37 (m, 1H), 3.78 (s, 3H), 3.76 – 3.50 (m, 2H). ^{13}C NMR (101 MHz, $CDCl_3$) δ 171.5, 164.1, 149.3, 148.3, 140.9, 140.4, 137.3, 130.4, 126.4, 122.4, 100.5, 52.9, 51.5, 48.2. IR (neat, cm^{-1}): 3300, 1738, 1672, 1507, 1432. HRMS *calcd.* for ($C_{16}H_{14}I_2N_2O_3$): 535.9094, *found* 535.9098.

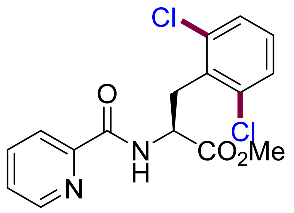

(7g)

Methyl (*S*)-3-(2,6-dichlorophenyl)-2-(picolinamido)propanoate (7g). Following the general procedure, using picolinamide **1a** (0.25 mmol, 71 mg), NCS (1.0 mmol, 134 mg) and AgF (0.50 mmol, 64 mg) provided 64 mg (73% yield) of **7g** as a colorless oil. ^{1}H NMR (400 MHz, $CDCl_3$) δ 8.69 (d, *J* = 8.9 Hz, 1H), 8.59 (ddd, *J* = 4.9, 1.8, 1.0 Hz, 1H), 8.08 (dt, *J* = 7.8, 1.1 Hz, 1H), 7.80 (td, *J* = 7.7, 1.7 Hz, 1H), 7.42 (ddd, *J* = 7.6, 4.8, 1.2 Hz, 1H), 7.27 (d, *J* = 7.9 Hz, 2H), 7.10 (dd, *J* = 8.5, 7.5 Hz, 1H), 5.22 (m, 1H), 3.76 (s, 3H), 3.61 – 3.51 (m, 2H). ^{13}C NMR (101 MHz, $CDCl_3$) δ 171.8, 164.3, 149.3, 148.4, 137.3, 136.3, 133.0, 128.9, 128.4, 126.5, 122.4, 52.8, 51.3, 33.9. IR (neat, cm^{-1}): 3330, 1741, 1675, 1509, 1433. HRMS *calcd.* for ($C_{16}H_{14}Cl_2N_2O_3$): 352.0381, *found* 352.0391.

7.- X-Ray Crystallography of 3i'

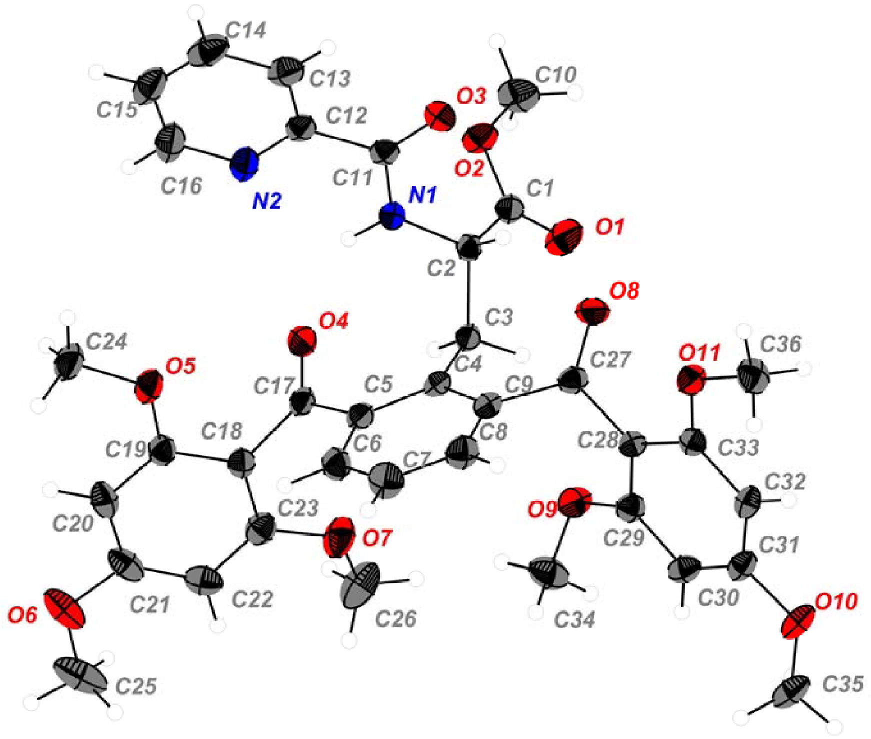

See CIF file attached.

Intensity data were collected on an Agilent Technologies Super-Nova diffractometer, which was equipped with monochromated Cu kα radiation (λ= 1.54184 Å) and Atlas CCD detector. Measurement was carried out at 150.01(10) K with the help of an Oxford Cryostream 700 PLUS temperature device. Data frames were processed (united cell determination, analytical absorption correction with face indexing, intensity data integration and correction for Lorentz and polarization effects) using the Crysalis software package. The structure was solved using SHELXT and refined by full-matrix least-squares with SHELXL-97. Final geometrical calculations were carried out with Mercury and PLATON as integrated in WinGX. Analysis of the absolute structure using likelihood methods (Hooft, Straver & Spek, 2008) was performed using PLATON (Spek, 2010). The results indicated that the absolute structure had been correctly assigned. The method calculated that the probability that the structure is inverted is smaller than 10^{-99}. The absolute structure parameter y (Hooft, Straver & Spek, 2008) was calculated using PLATON (Spek, 2010). The resulting value was y=-0.005(17), which together with Flack parameter value, indicate that the absolute structure has probably been determined correctly".

Empirical formula	$C_{36}H_{36}N_2O_{11}$
Formula weight	672.67
Temperature/K	150.00(10)
Crystal system	orthorhombic
Space group	$P2_12_12_1$
a/Å	10.02984(8)
b/Å	10.88201(8)
c/Å	30.5021(2)
α/°	90.0
β/°	90.0
γ/°	90.0
Volume/Å3	3329.14(4)
Z	4
ρcalcg/cm^3	1.342
μ/mm^{-1}	0.836
F(000)	1416.0
Crystal size/mm^3	0.375 × 0.296 × 0.195
Radiation	CuKα (λ = 1.54184)
2Θ range for data collection/°	8.628 to 139.96
Index ranges	$-12 \le h \le 11, -12 \le k \le 13, -36 \le l \le 37$
Reflections collected	37271
Independent reflections	6308 [R_{int} = 0.0329, R_{sigma} = 0.0202]
Data/restraints/parameters	6308/19/452
Goodness-of-fit on F2	1.054
R(F) (I>2σI, all)	0.0301, 0.0315
Rw(F2)[a] (I>2σI ,all)	0.0738, 0.0749
Largest diff. peak/hole / e Å-3	0.13/-0.17
Friedel coverage	100%
Flack x	0.03(5)
Hooft y	0.01(4)
P2(wrong)	$<10^{-99}$

[a]= $1/[\sigma^2(Fo^2)+(0.0406P)^2]$ where $P = [Max(Fo^2,0)+2Fc^2]/3$.

8.- X-Ray Crystallography of 7c

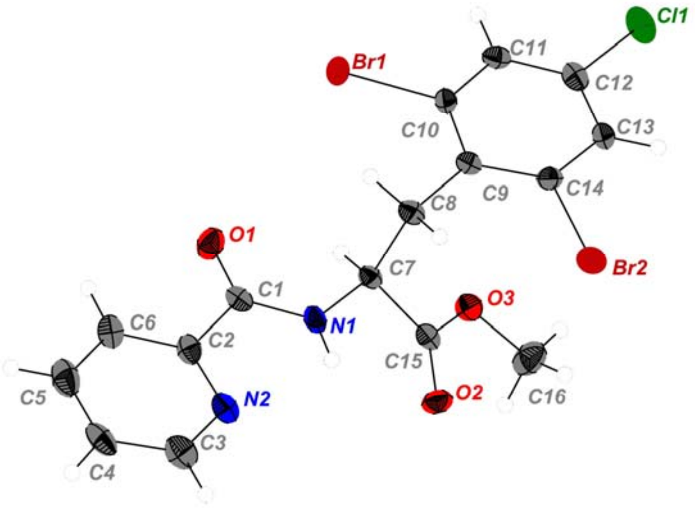

See CIF file attached.

Intensity data were collected on an Agilent Technologies Super-Nova diffractometer, which was equipped with monochromated Cu kα radiation (λ= 1.54184 Å) and Atlas CCD detector. Measurement was carried out at 150.01(10) K with the help of an Oxford Cryostream 700 PLUS temperature device. Data frames were processed (united cell determination, analytical absorption correction with face indexing, intensity data integration and correction for Lorentz and polarization effects) using the Crysalis software package. The structure was solved using SHELXT and refined by full-matrix least-squares with SHELXL-97. Final geometrical calculations were carried out with Mercury and PLATON as integrated in WinGX. Analysis of the absolute structure using likelihood methods (Hooft, Straver & Spek, 2008) was performed using PLATON (Spek, 2010). The results indicated that the absolute structure had been correctly assigned. The method calculated that the probability that the structure is inverted is smaller than 10^{-99}. The absolute structure parameter y (Hooft, Straver & Spek, 2008) was calculated using PLATON (Spek, 2010). The resulting value was y=-0.005(17), which together with Flack parameter value, indicate that the absolute structure has probably been determined correctly".

Empirical formula	$C_{16}H_{13}Br_2ClN_2O_3$
Formula weight	476.55
Temperature/K	150.00(10)
Crystal system	monoclinic
Space group	$P2_1$
a/Å	8.22428(16)

b/Å	13.92307(19)
c/Å	8.40898(16)
α/°	90.0
β/°	115.927(2)
γ/°	90.0
Volume/Å3	865.97(3)
Z	2
ρcalcg/cm^3	1.828
μ/mm^{-1}	7.522
F(000)	468.0
Crystal size/mm^3	0.427 × 0.164 × 0.094
Radiation	CuKα (λ = 1.54184)
2Θ range for data collection/°	11.702 to 137.916
Index ranges	-9 ≤ h ≤ 8, -16 ≤ k ≤ 16, -9 ≤ l ≤ 10
Reflections collected	9363
Independent reflections	3199 [R_{int} = 0.0382, R_{sigma} = 0.0373]
Data/restraints/parameters	3199/1/218
Goodness-of-fit on F2	1.047
R(F) (I>2σI, all)	R1 = 0.0295, wR2 = 0.0720
Rw(F2)[a] (I>2σI ,all)	R1 = 0.0301, wR2 = 0.0727
Largest diff. peak/hole / e Å-3	0.31/-0.36
Friedel coverage	100%
Flack x	0.00(2)
Hooft y	0.005(17)
P2(wrong)	$<10^{-99}$

[a]= $1/[\sigma^2(Fo^2)+(0.0441P)^2]$ where $P = [Max(Fo^2,0)+2Fc^2]/3$.

9.- Mechanistic Experiments

9.1-Synthesis and reactivity of IntA

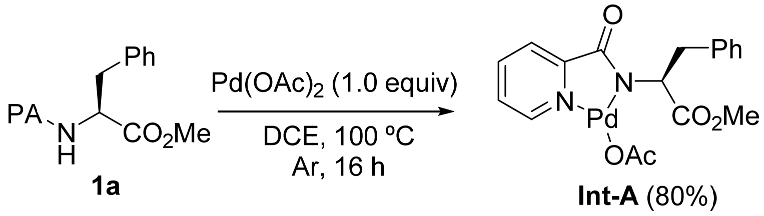

A reaction Schlenk containing a stirring bar was charged with **1a** (1.50 mmol, 426 mg) and $Pd(OAc)_2$ (1.50 mmol, 336 mg). The reaction tube was then evacuated and back-filled with dry argon (this sequence was repeated up to three times). Then, 1,2-dichoroethane (12 mL) was added under argon atmosphere. The reaction mixture was next warmed up to 100 ºC and stirred overnight. The resulting mixture was diluted with dichloromethane, filtered through a pad of celite and concentrated under reduced pressure. The obtained brown solid was filtrated and washed with hexanes to afford **IntA** as a yellowish solid (540 mg, 80 % yield). ^{1}H NMR (400 MHz, $CDCl_3$) δ 7.91 – 7.83 (m, 1H), 7.62 (ddd, *J* = 12.6, 7.5, 1.7 Hz, 2H), 7.20 (ddd, *J* = 8.1, 5.2, 2.7 Hz, 2H), 7.12 – 6.94 (m, 4H), 4.23 (dd, *J* = 7.8, 4.9 Hz, 1H), 3.61 (s, 3H), 3.24 (dd, *J* = 13.4, 5.1 Hz, 1H), 2.67 (dd, *J* = 13.4, 7.7 Hz, 1H), 2.13 (s, 3H). ^{13}C NMR (101 MHz, $CDCl_3$) δ 185.4, 172.3, 170.3, 155.0, 146.2, 140.1, 138.9, 129.4, 129.3, 128.1, 126.3, 126.1, 125.9, 58.7, 52.3, 38.3, 24.3. MS (ESI$^+$) m/z (%) 389 (M-OAc). HRMS *calcd.* for ($C_{16}H_{15}N_2O_3Pd$): 398.0118, *found* 398.0121.

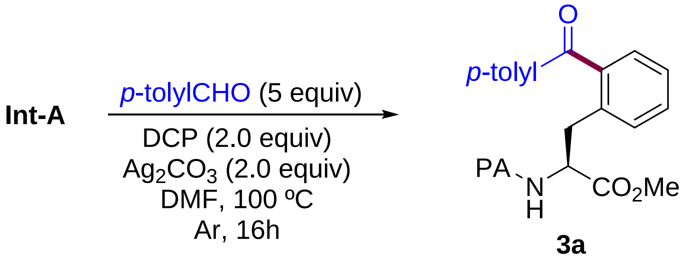

entry	DCP	Ag_2CO_3	Yield of 3a (%)^a
1	✓	✓	45
2	✓	✗	26
3	✗	✓	0
4	✗	✗	0

^{a} Yield of isolated product after column chromatography

1a + **2a** $\xrightarrow[\text{DCP (2.0 equiv), } Ag_2CO_3 \text{ (2.0 equiv), DMF, 100 ºC, Ar, 16h}]{p\text{-tolylCHO (5 equiv), } \textbf{Int-A} \text{ (10 mol\%)}}$ **3a**, 58% (7:3)

9.2- Experiments with radical traps

1a + **2a** $\xrightarrow[\text{DCP (2.0 equiv), } Ag_2CO_3 \text{ (2.0 equiv), Radical trap (1.0 equiv), DMF, 100 ºC, Ar, 16h}]{Pd(OAc)_2 \text{ (10 mol\%)}}$ **3a**

radical trap	3a (%)
TEMPO	0
BHT	0
diphenylethylene	0

10.-Determination of *ee* by HPLC Analysis

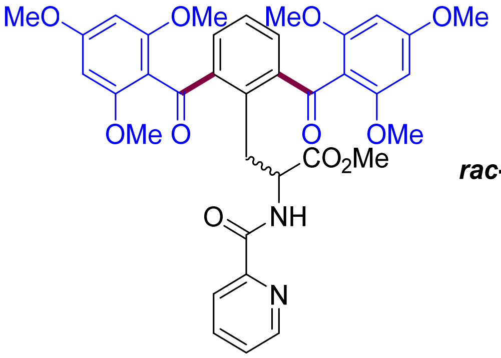

***rac*-(3i')**

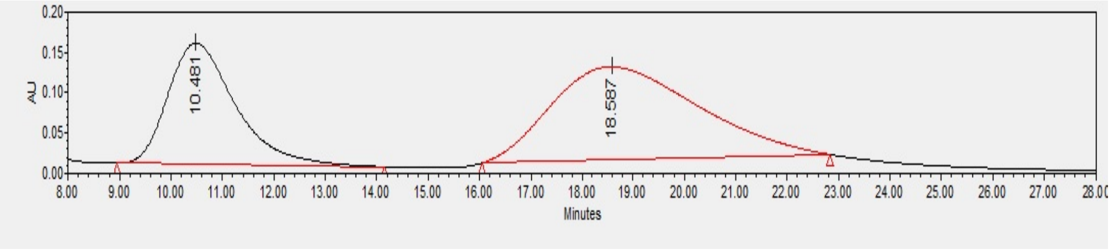

Chiralpak IA; 20:80 Hexane: isopropanol; 1 mL/min, λ = 245 nm

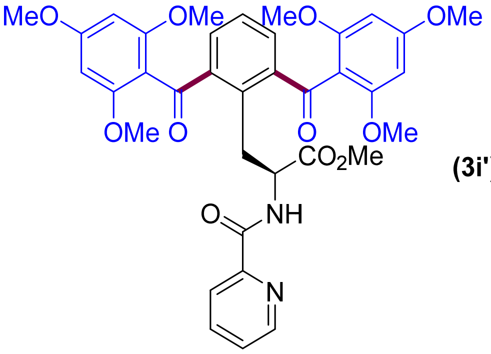

(3i') 99% *ee*

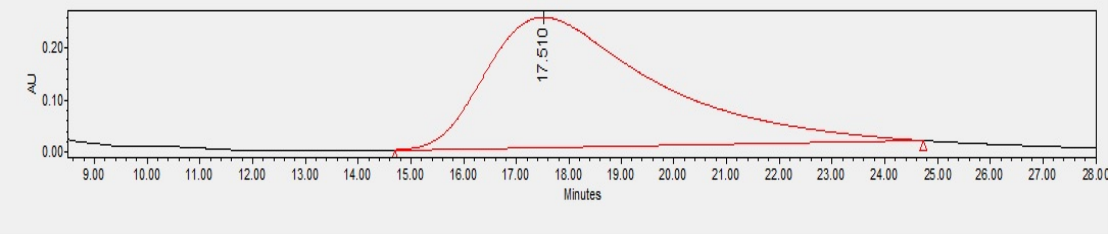

11.-^{1}H NMR and ^{13}C NMR Spectra

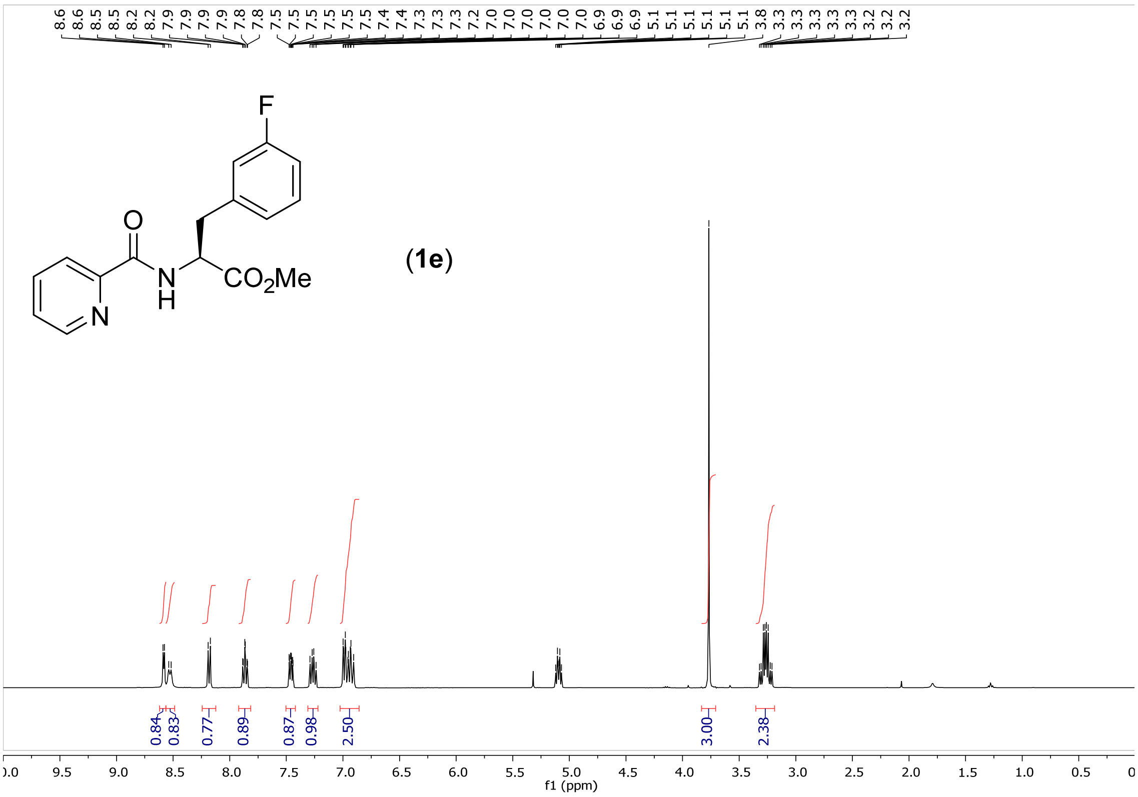

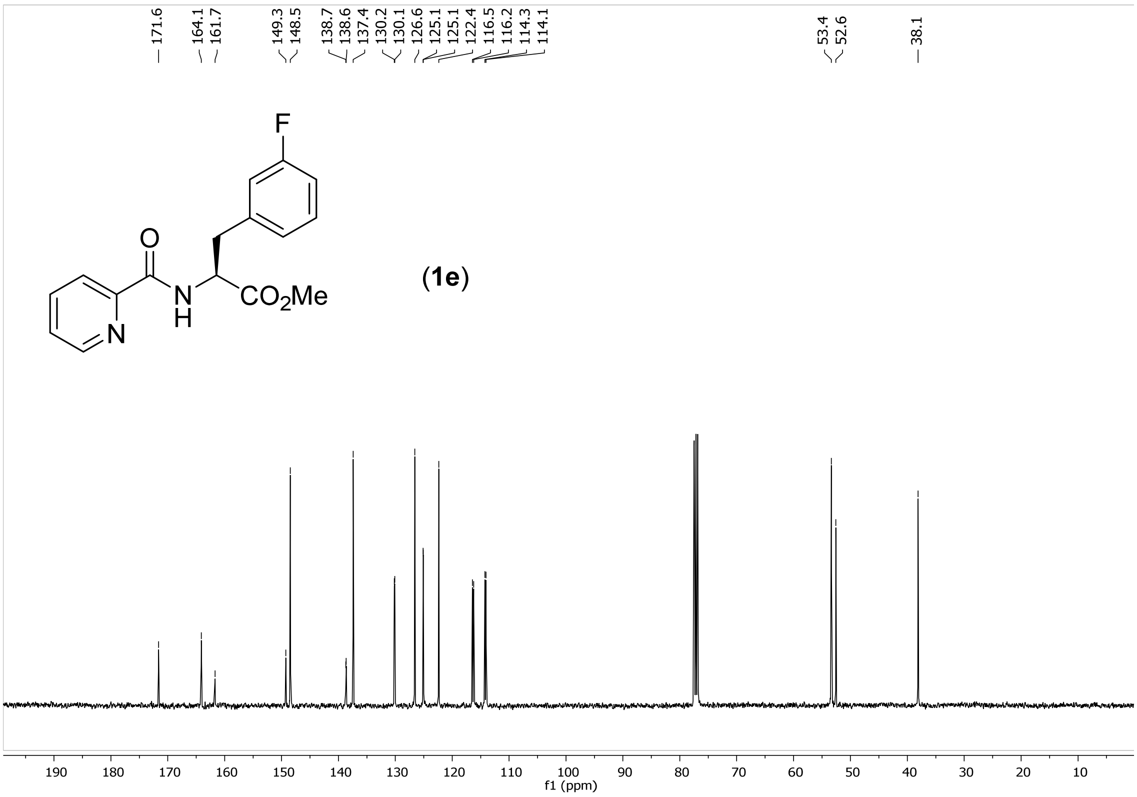

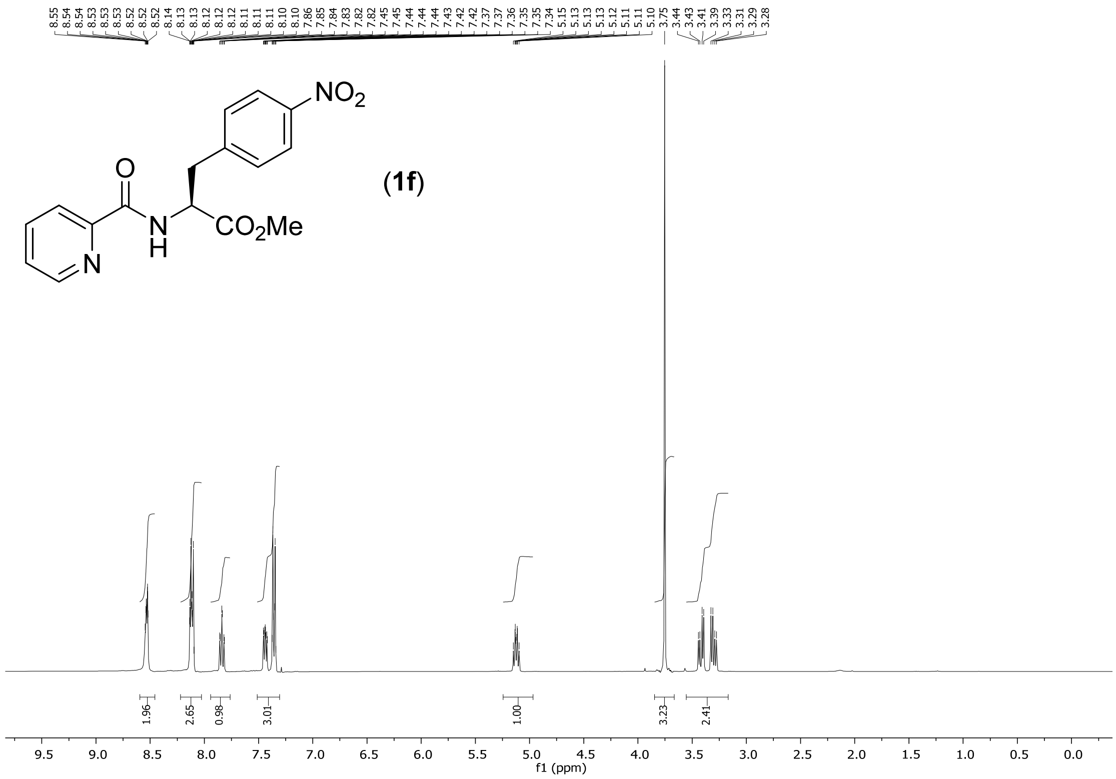
8.55 8.54 8.54 8.53 8.53 8.53 8.52 8.52 8.52 8.14 8.13 8.13 8.12 8.12 8.12 8.11 8.11 8.11 8.10 8.10 7.86 7.85 7.84 7.83 7.82 7.82 7.45 7.45 7.44 7.44 7.44 7.43 7.42 7.42 7.37 7.37 7.36 7.35 7.35 7.34 5.15 5.15 5.13 5.13 5.13 5.12 5.11 5.11 5.10 3.75 3.44 3.43 3.41 3.39 3.33 3.31 3.29 3.28
NO2
O
N
H
N
CO2Me
(1f)
1.96
2.65
0.98
3.01
1.00
3.23
2.41
f1 (ppm)

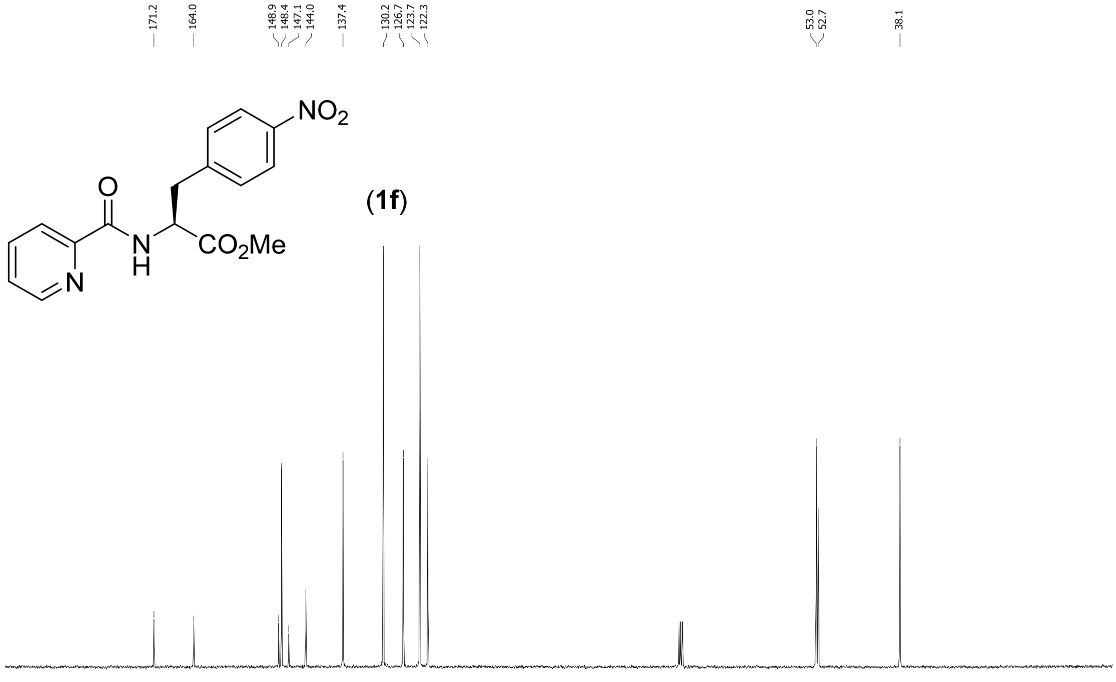
171.2
164.0
148.9 148.4 147.1 144.0
137.4
130.2 126.7 123.7 122.3
53.0 52.7
38.1
NO2
O
N
H
N
CO2Me
(1f)
f1 (ppm)

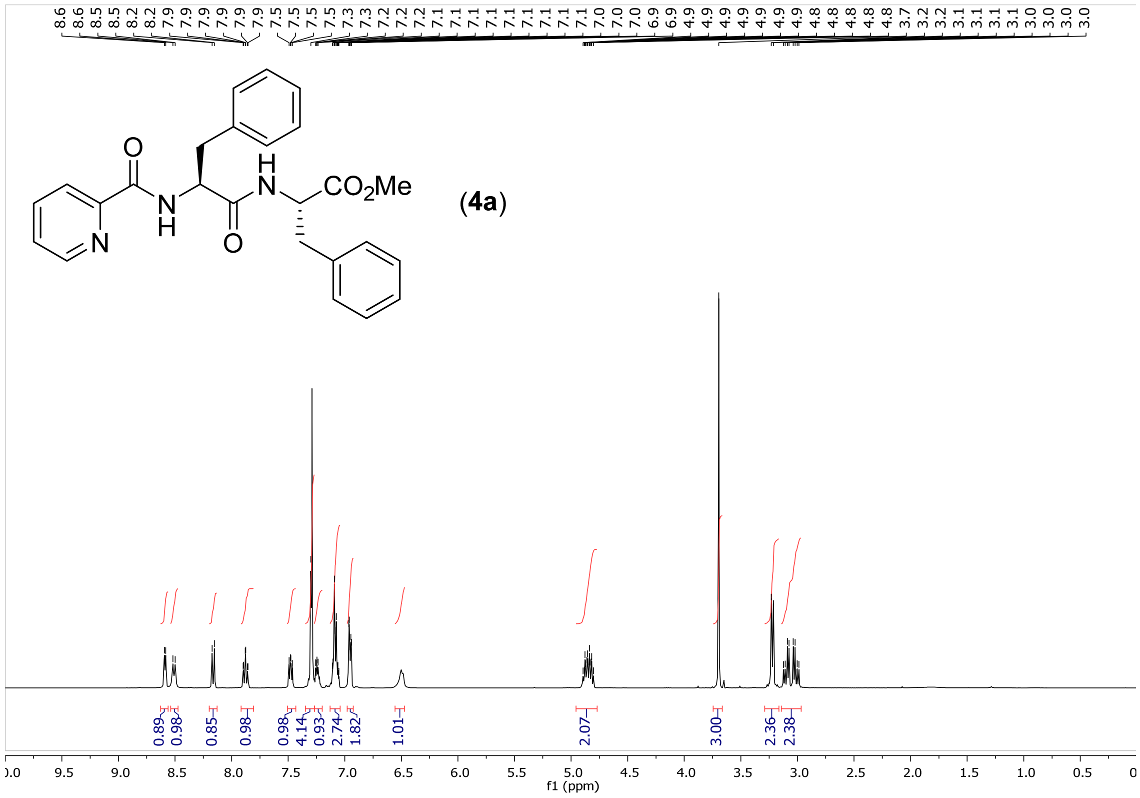
(4a)
f1 (ppm)

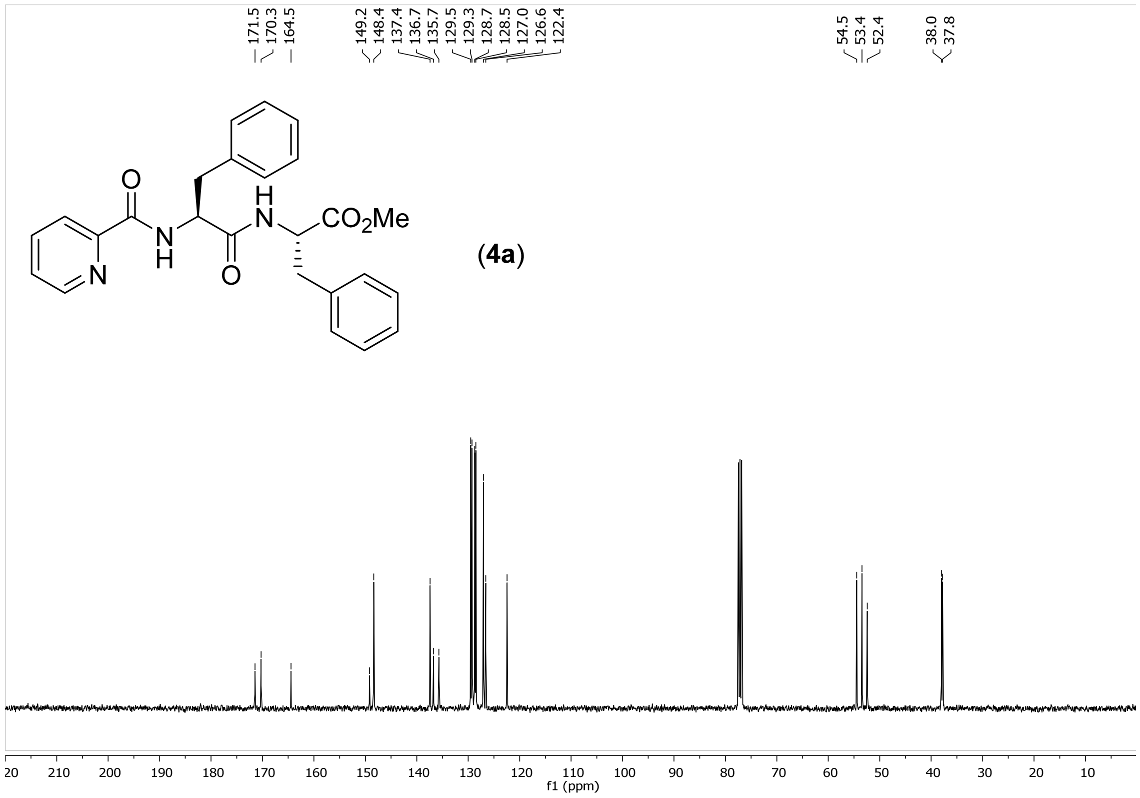
(4a)
f1 (ppm)

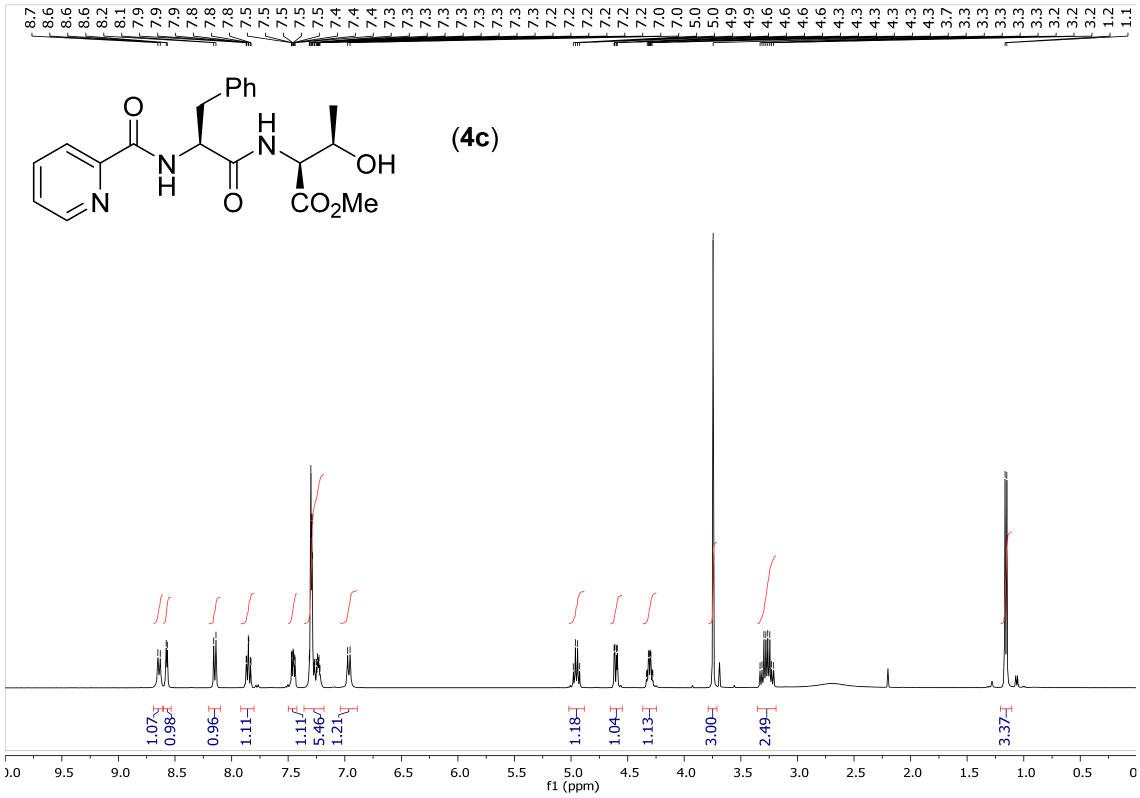

(**4c**)

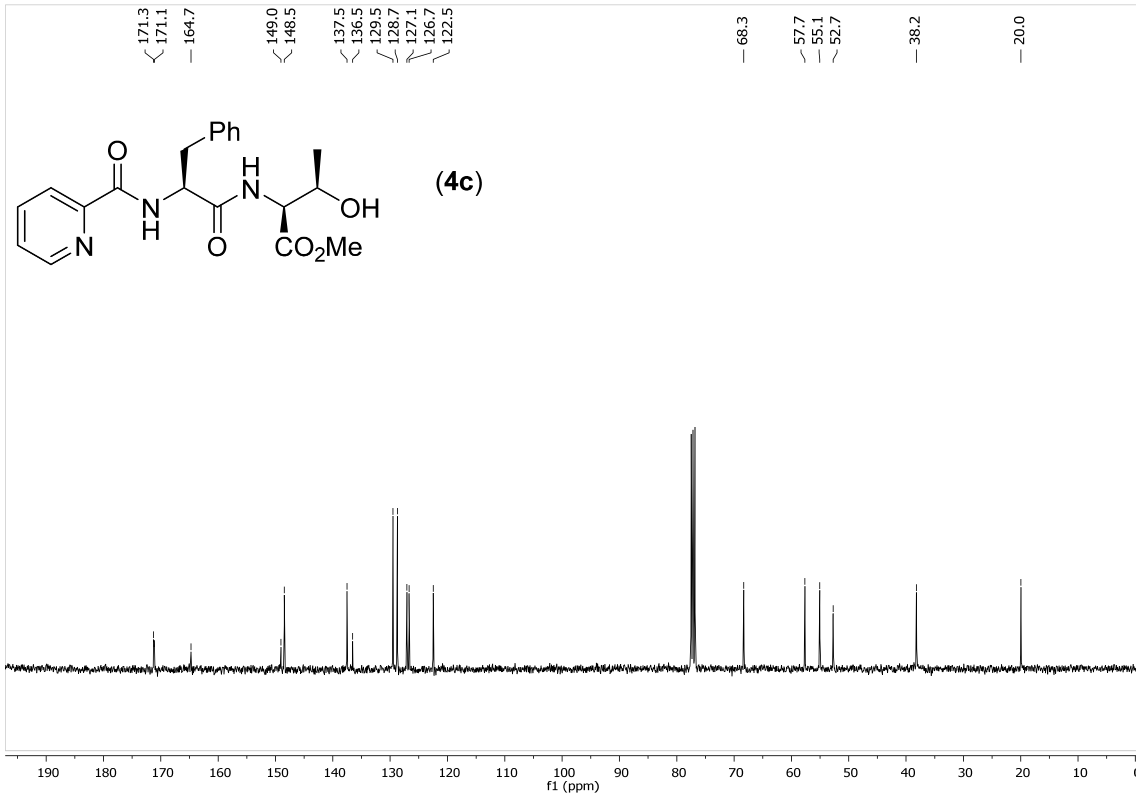

(**4c**)

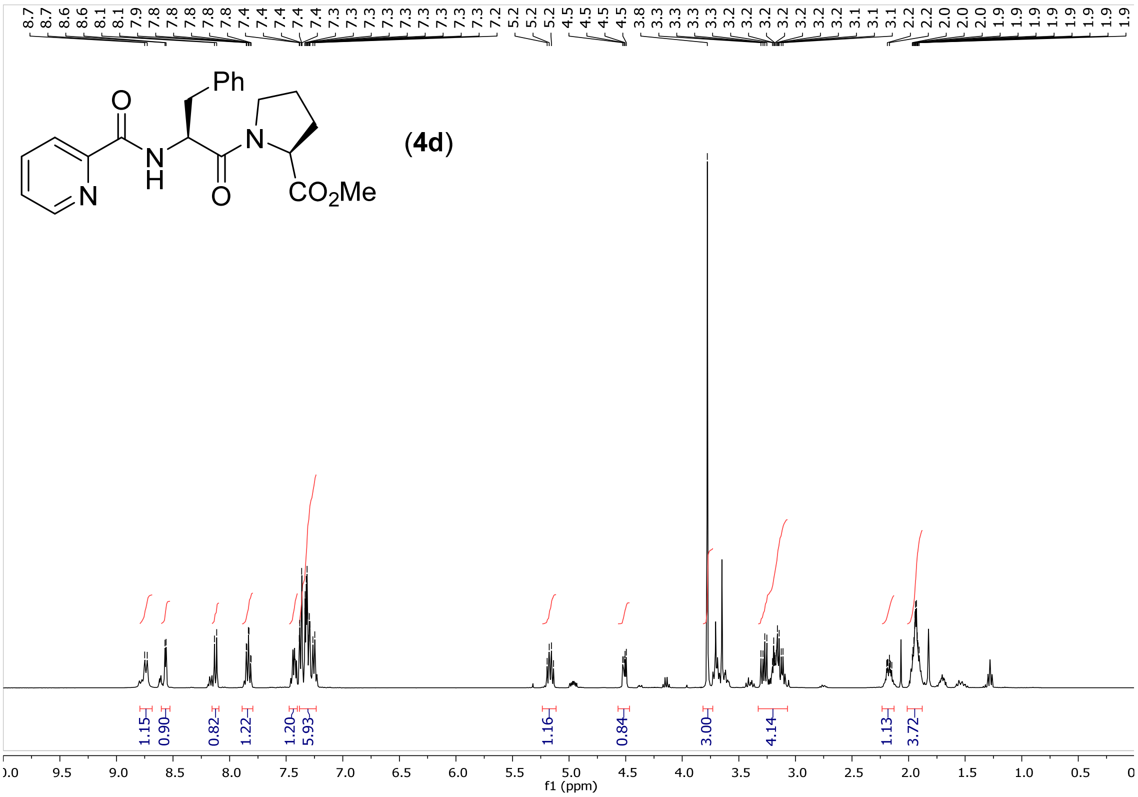

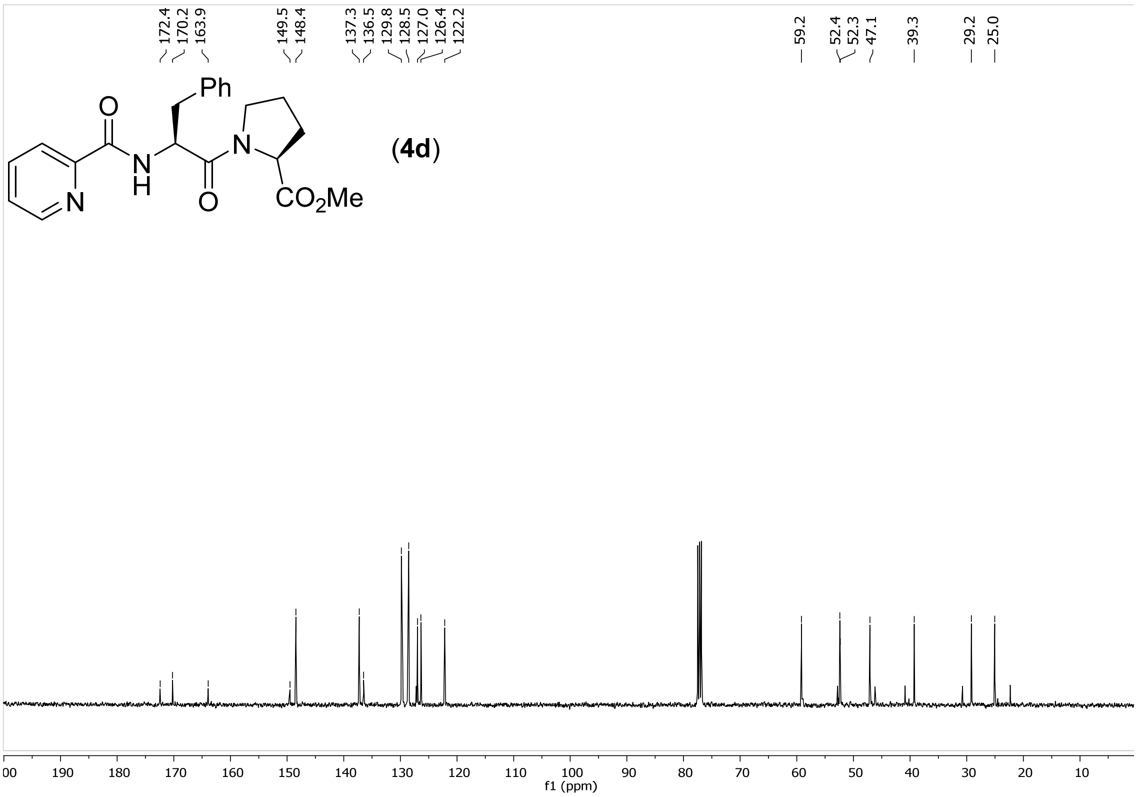

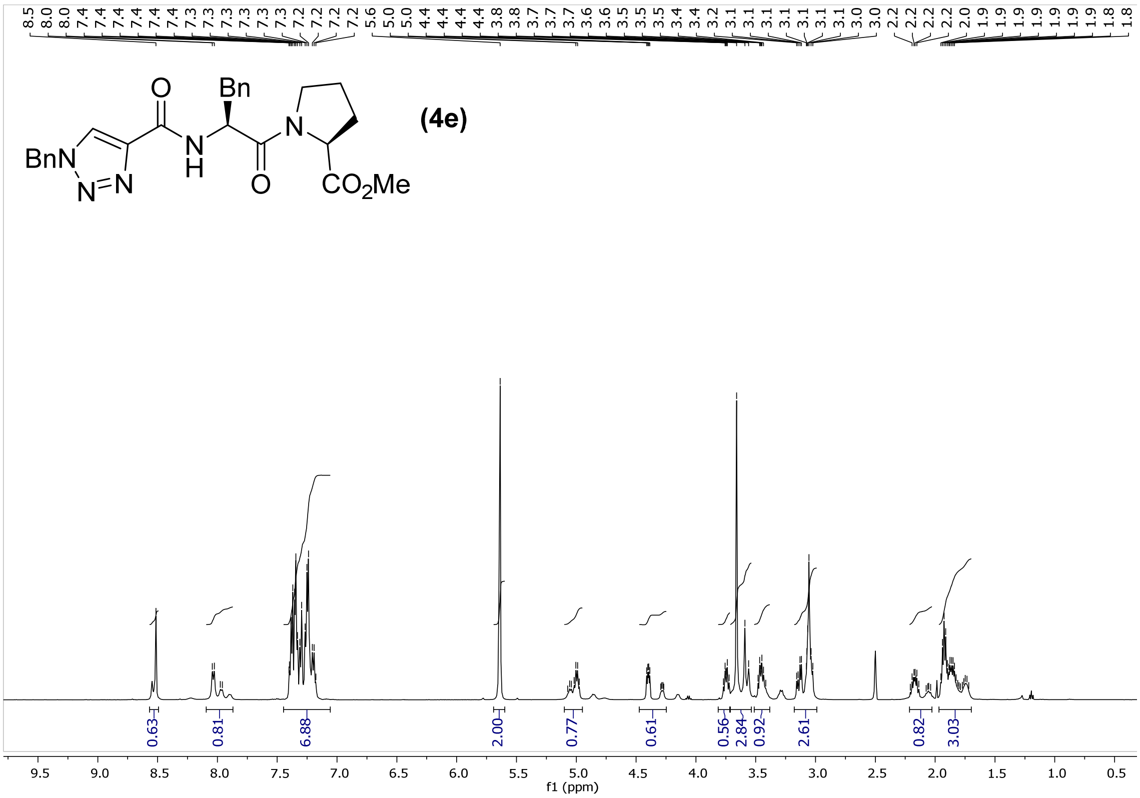
(4e)
Bn
BnN
N=N
O
N
H
O
N
CO2Me
f1 (ppm)

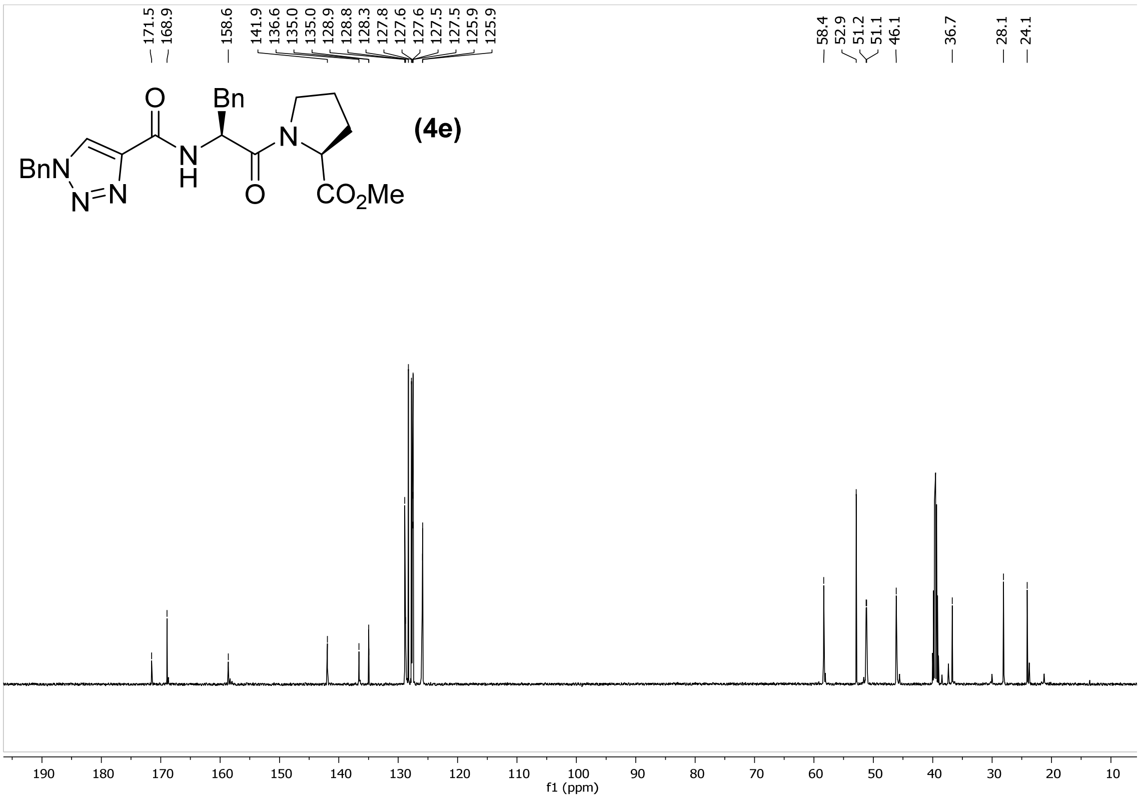
(4e)
Bn
BnN
N=N
O
N
H
O
N
CO2Me
f1 (ppm)

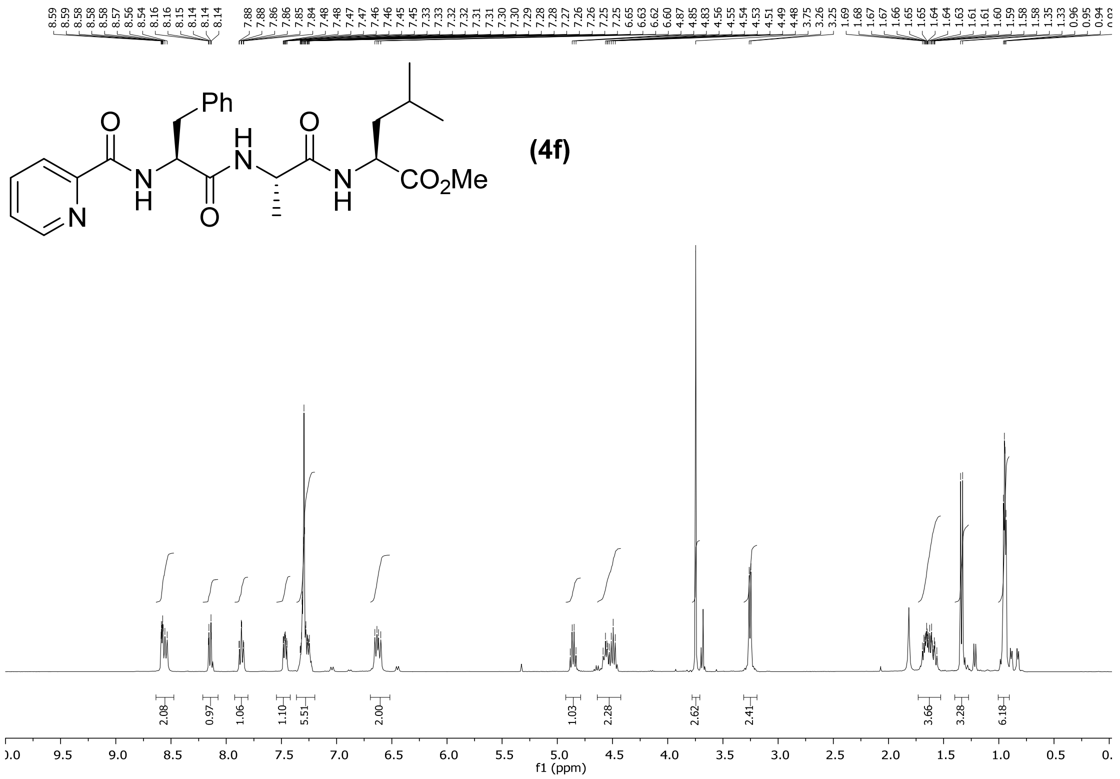

(4f)

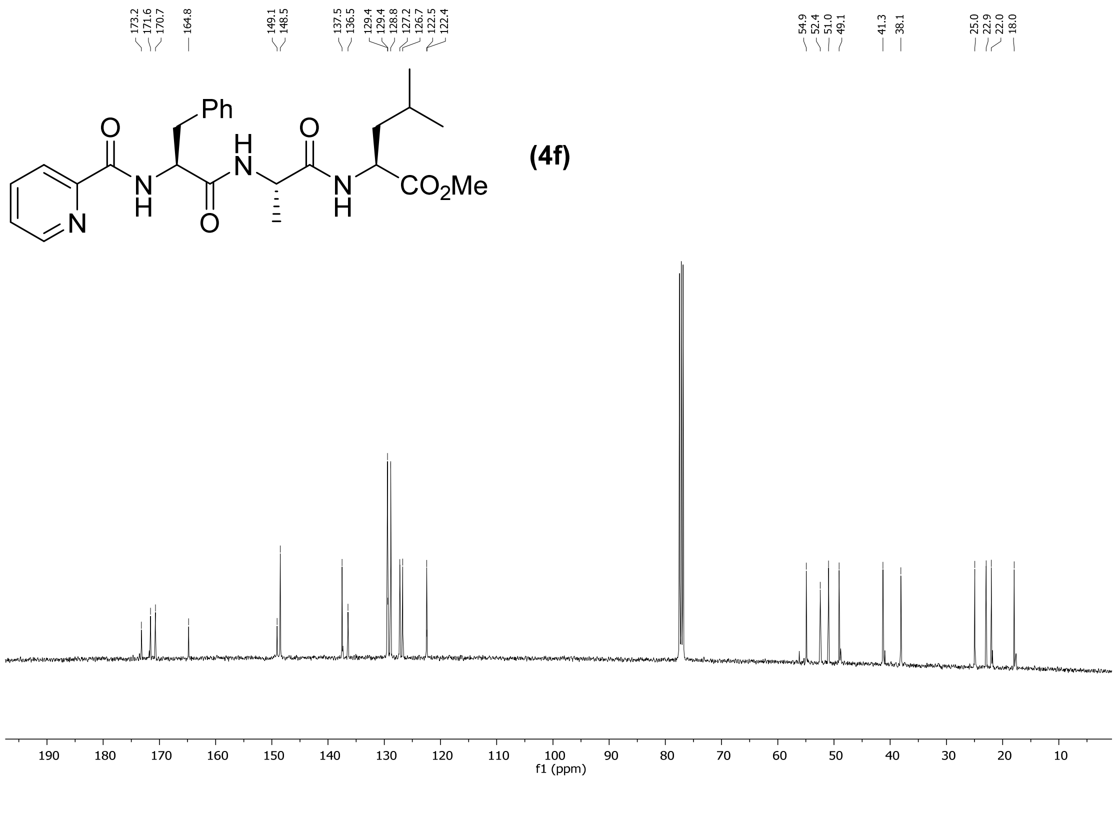

(4f)

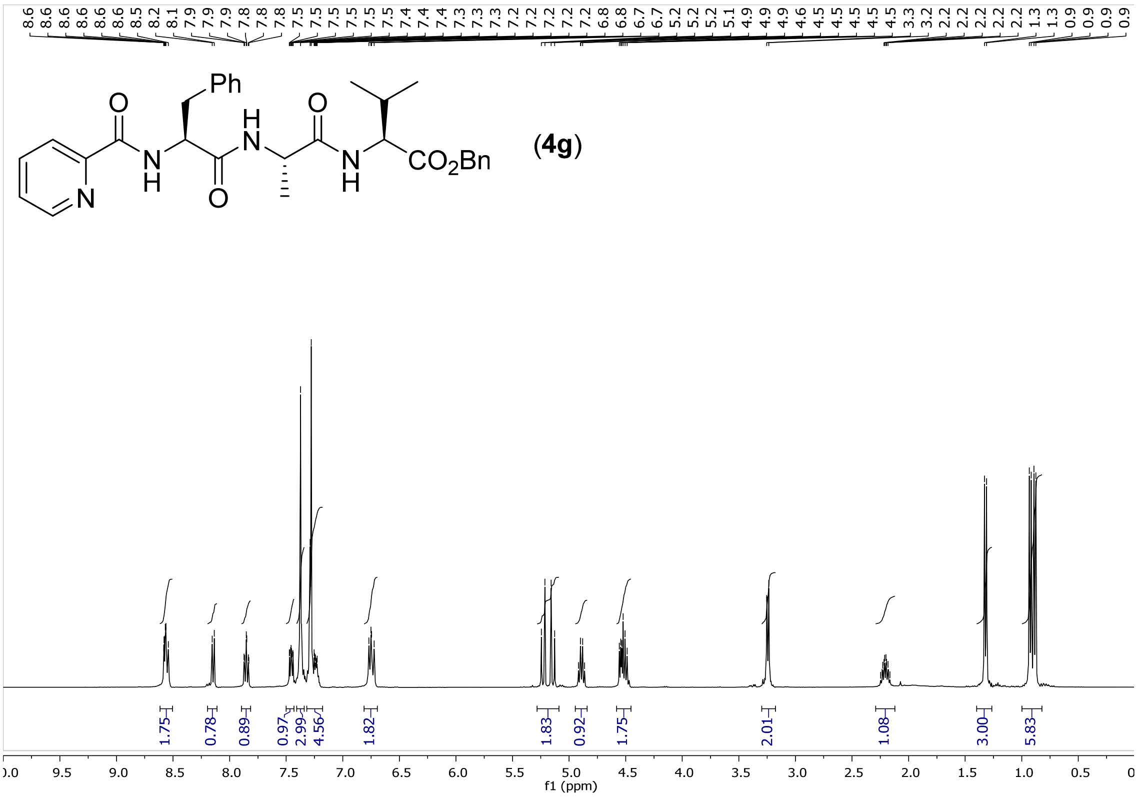

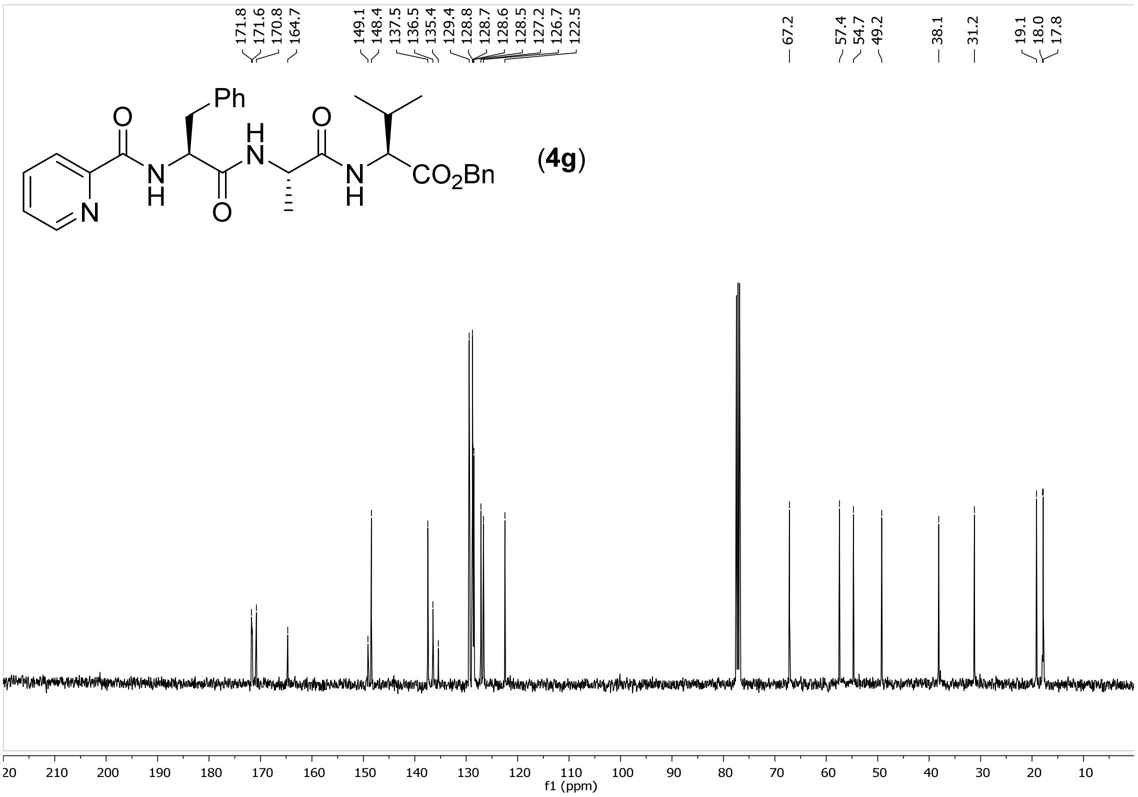

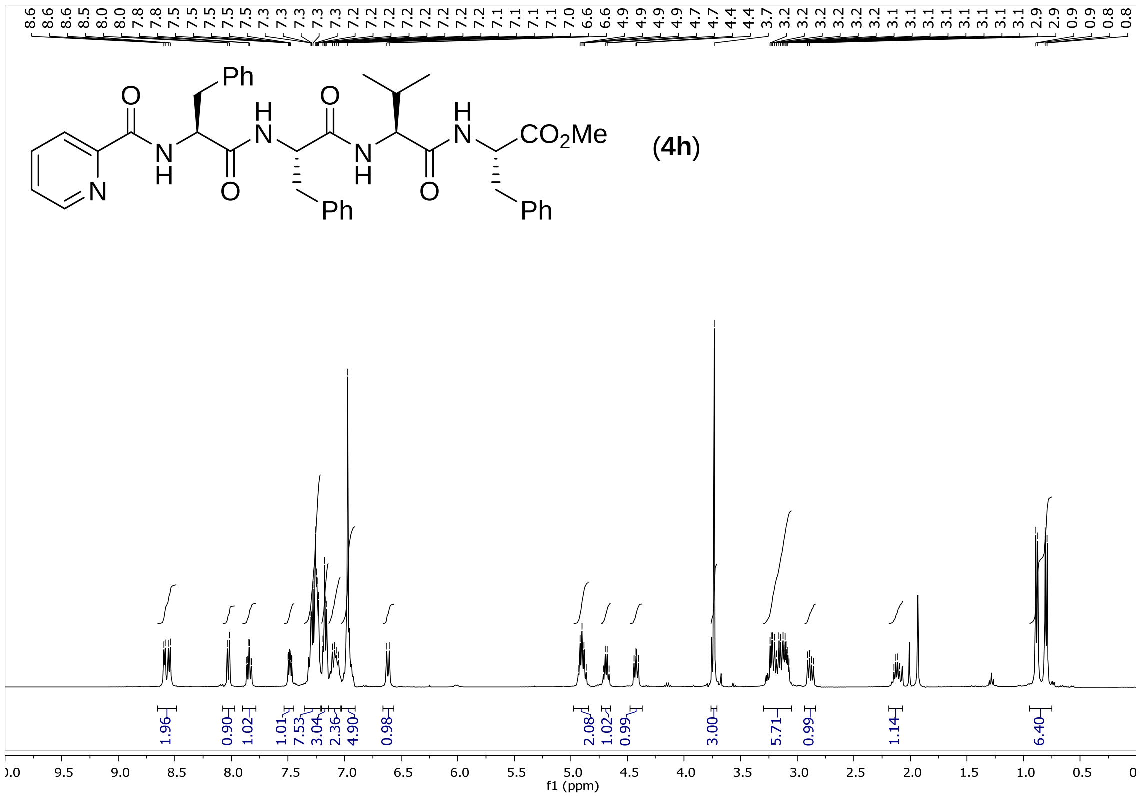

(4h)
f1 (ppm)

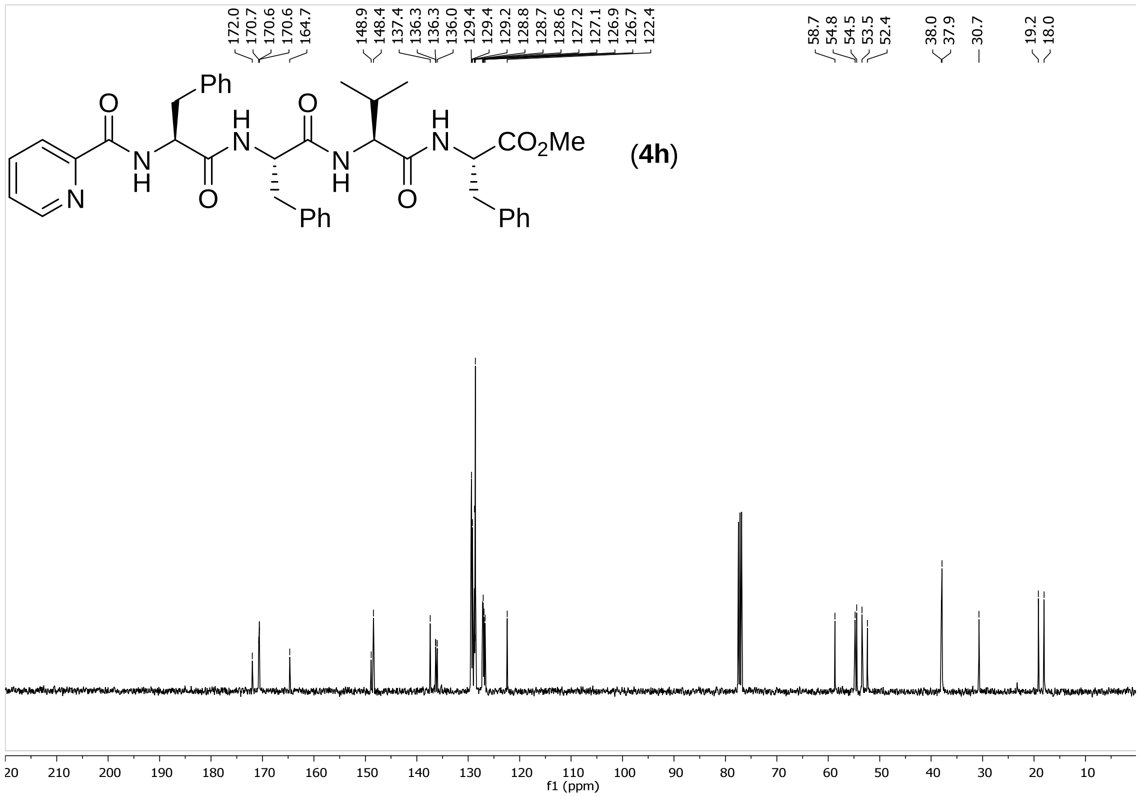

172.0
170.7
170.6
170.6
164.7
148.9
148.4
137.4
136.3
136.3
136.0
129.4
129.4
129.2
128.8
128.7
128.6
127.2
127.1
126.9
126.7
122.4
58.7
54.8
54.5
53.5
52.4
38.0
37.9
30.7
19.2
18.0
(4h)
f1 (ppm)

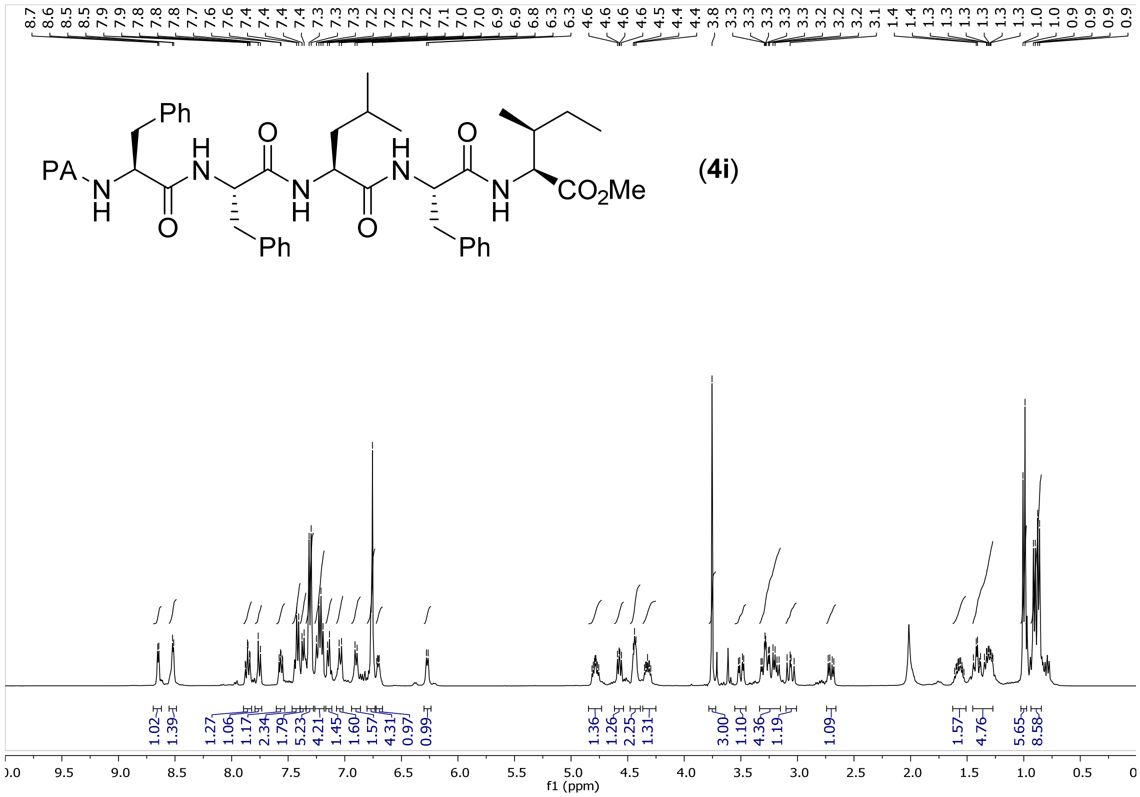

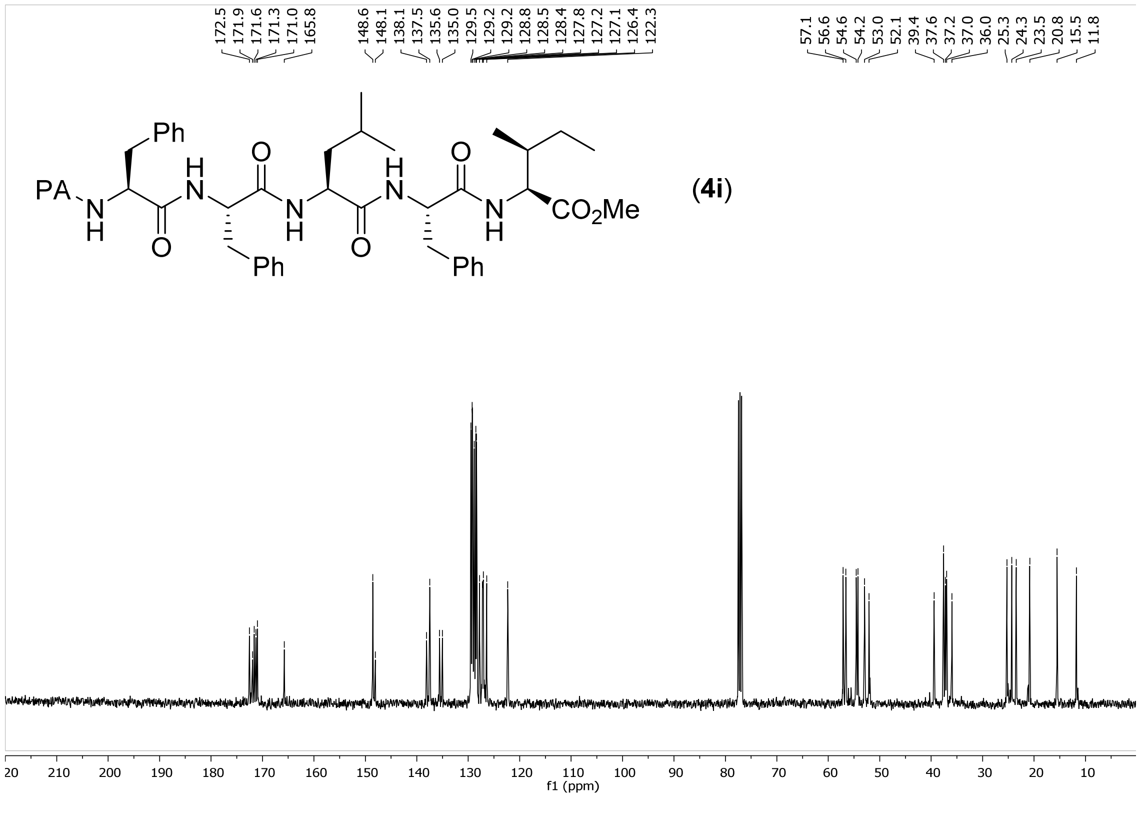

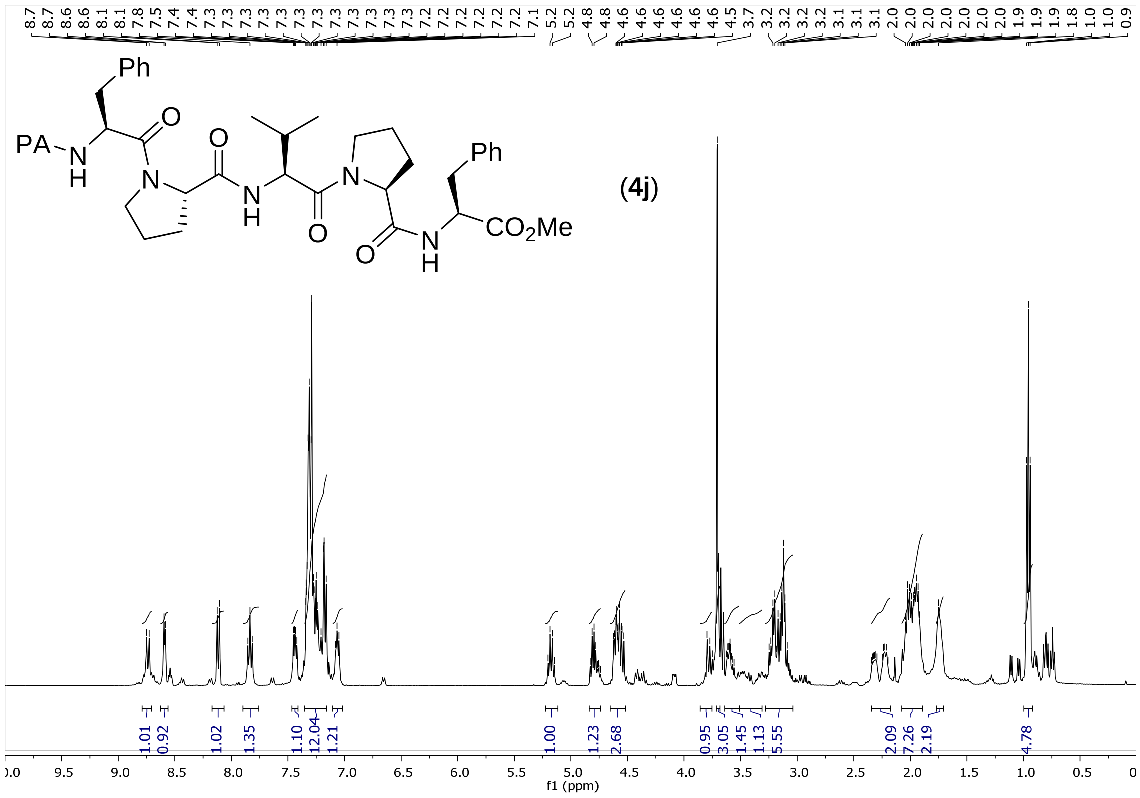

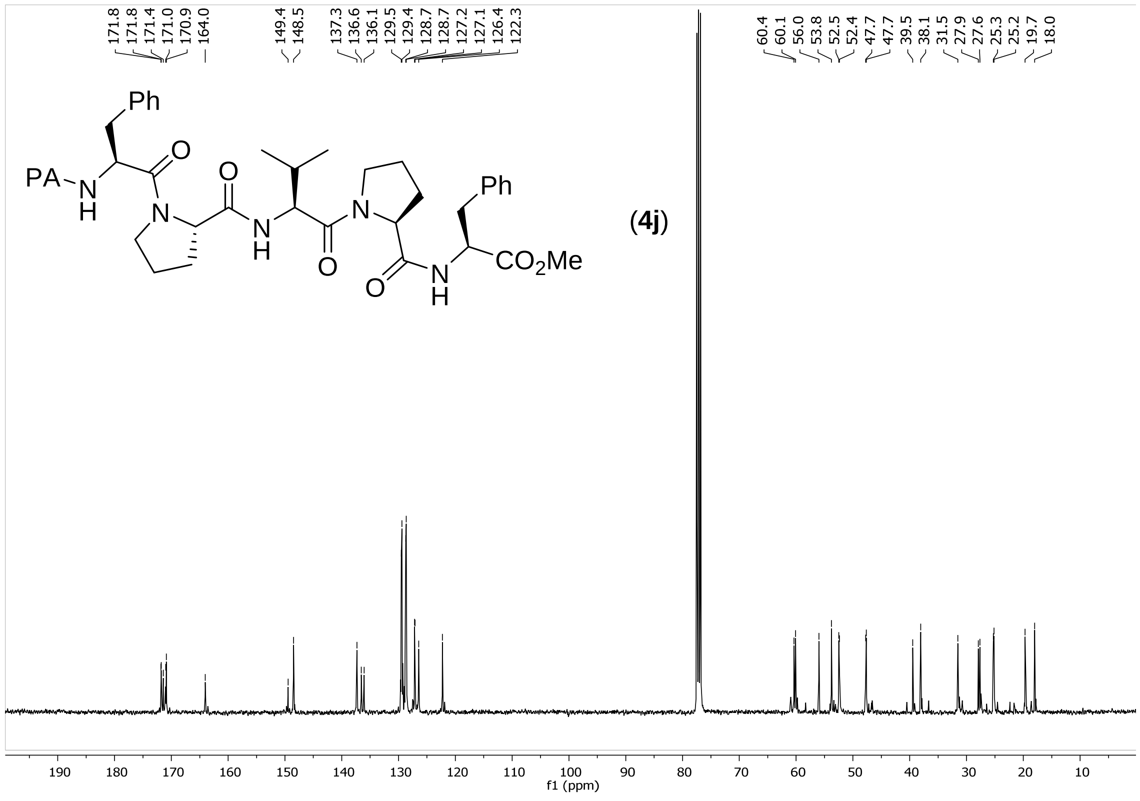

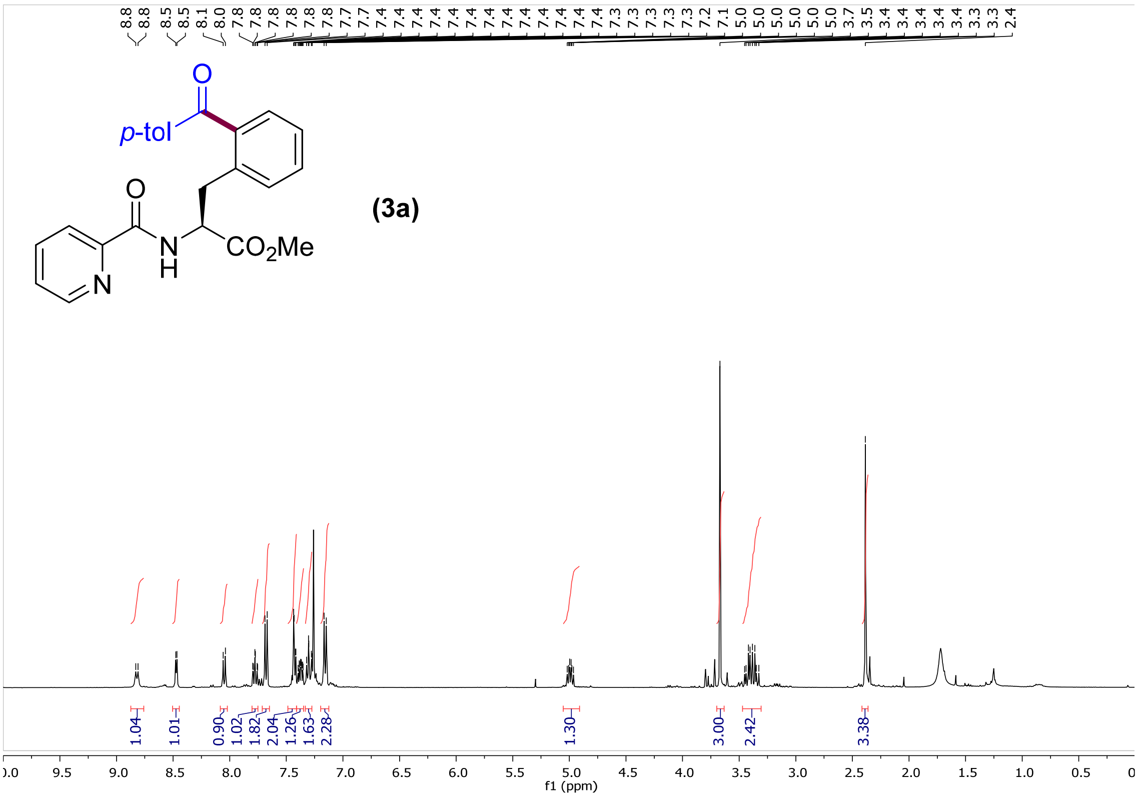
p-tol
(3a)
CO2Me
f1 (ppm)

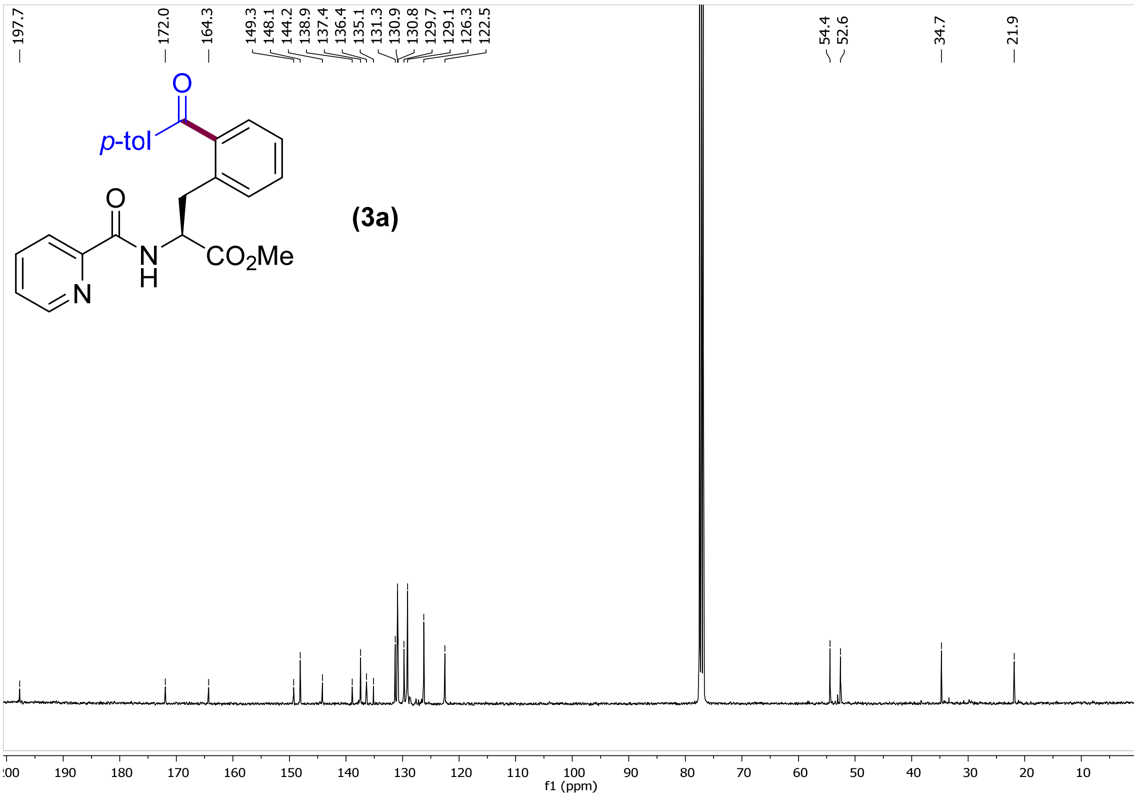
p-tol
(3a)
CO2Me
f1 (ppm)

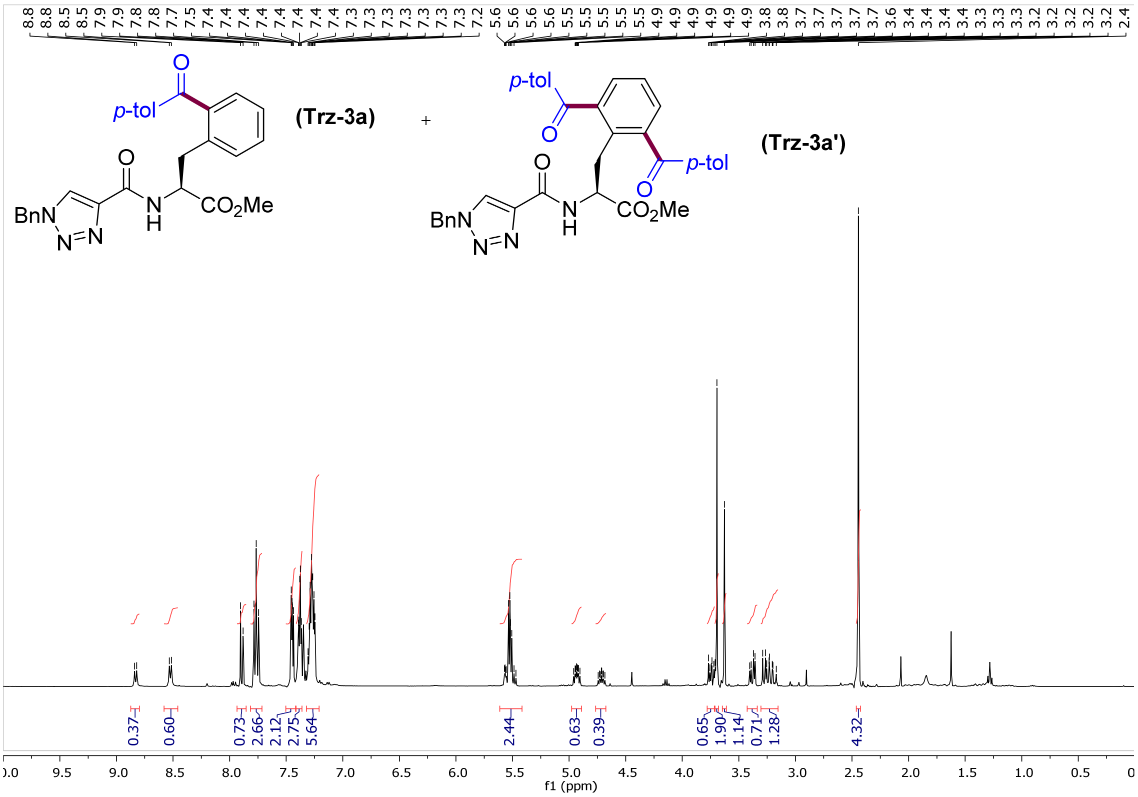
(Trz-3a)
(Trz-3a')
p-tol
BnN
N=N
N
H
CO_2Me
f1 (ppm)

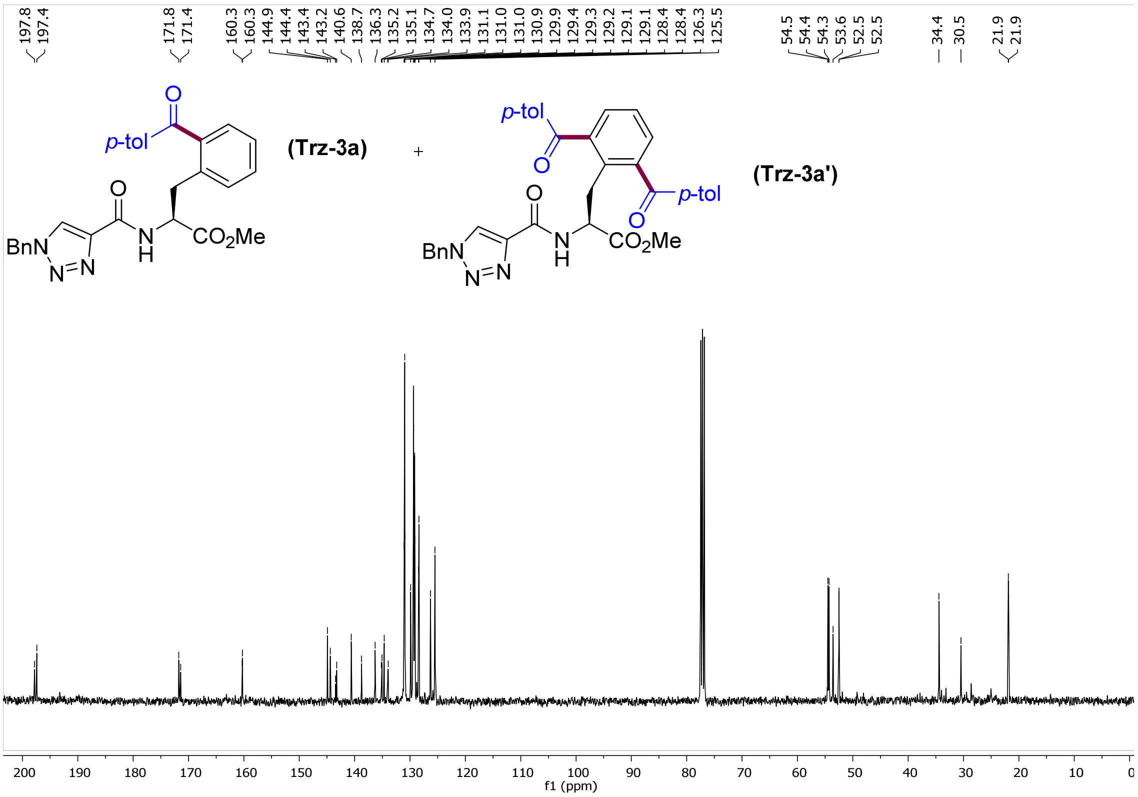
(Trz-3a)
(Trz-3a')
p-tol
BnN
N=N
N
H
CO_2Me
f1 (ppm)

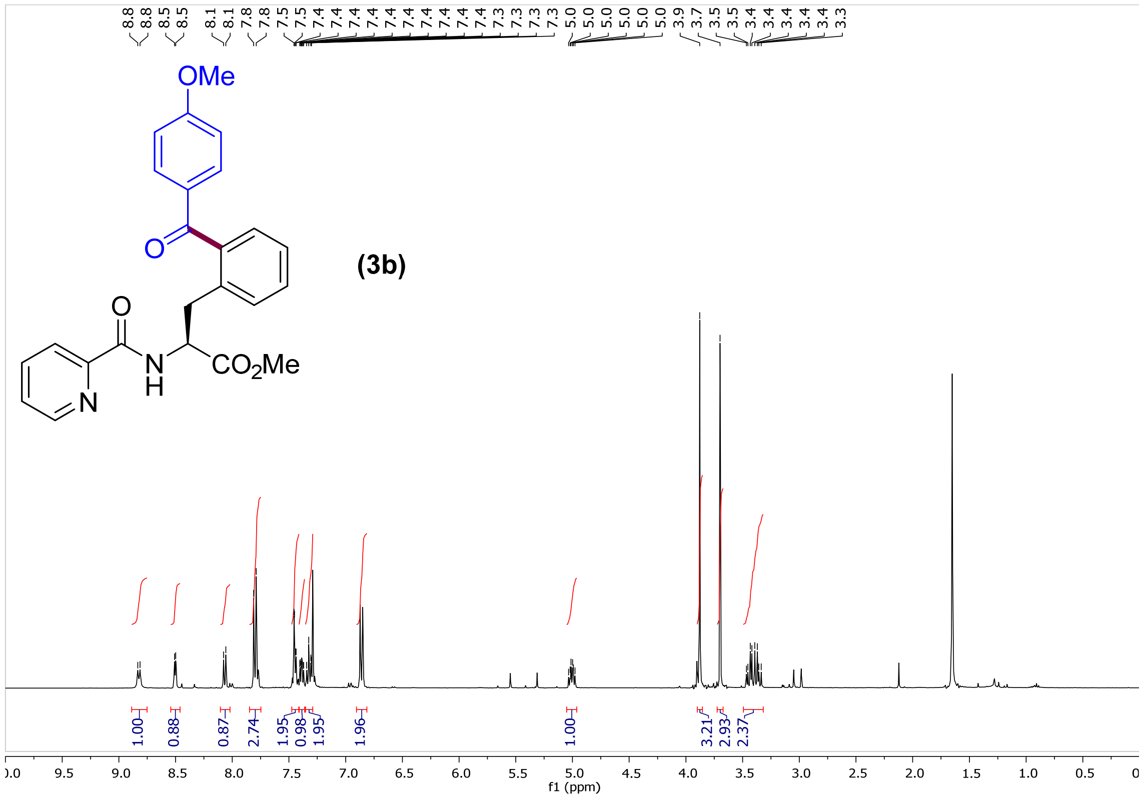

(3b)

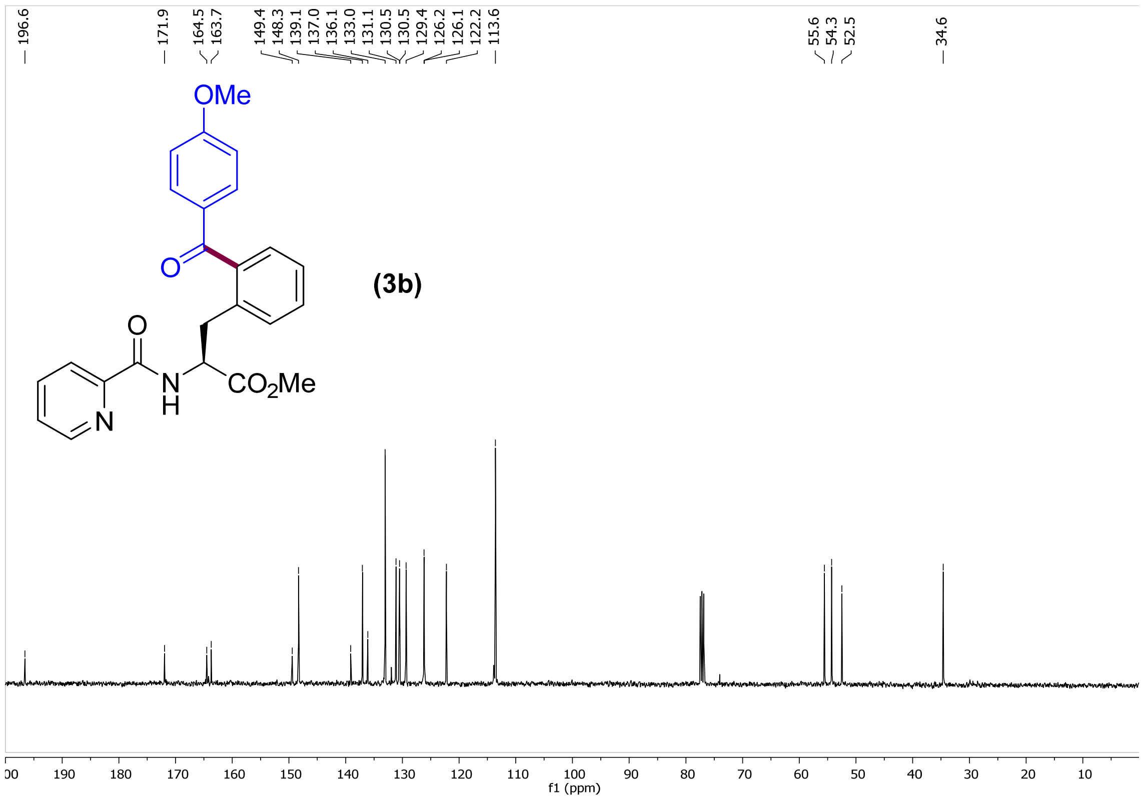

(3b)

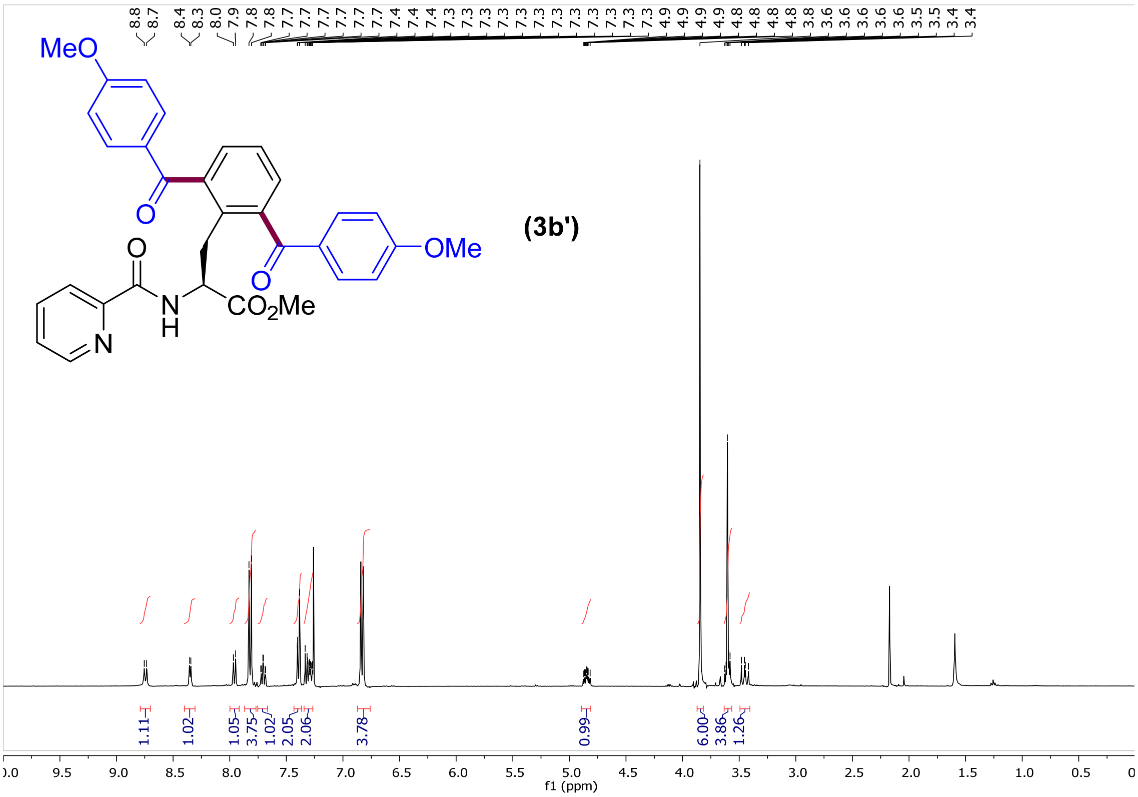
MeO
OMe
O
N
H
CO2Me
f1 (ppm)

(3b')

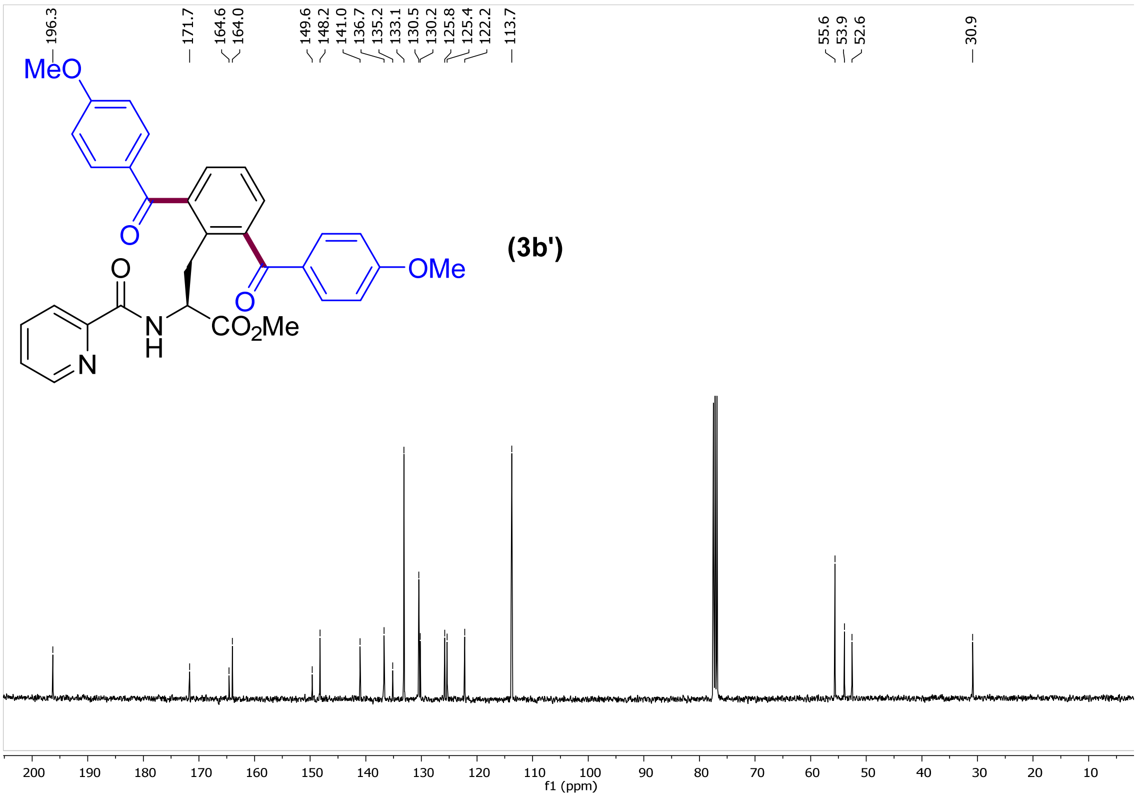
196.3
171.7
164.6
164.0
149.6
148.2
141.0
136.7
135.2
133.1
130.5
130.2
125.8
125.4
122.2
113.7
55.6
53.9
52.6
30.9
MeO
OMe
CO2Me
f1 (ppm)

(3b')

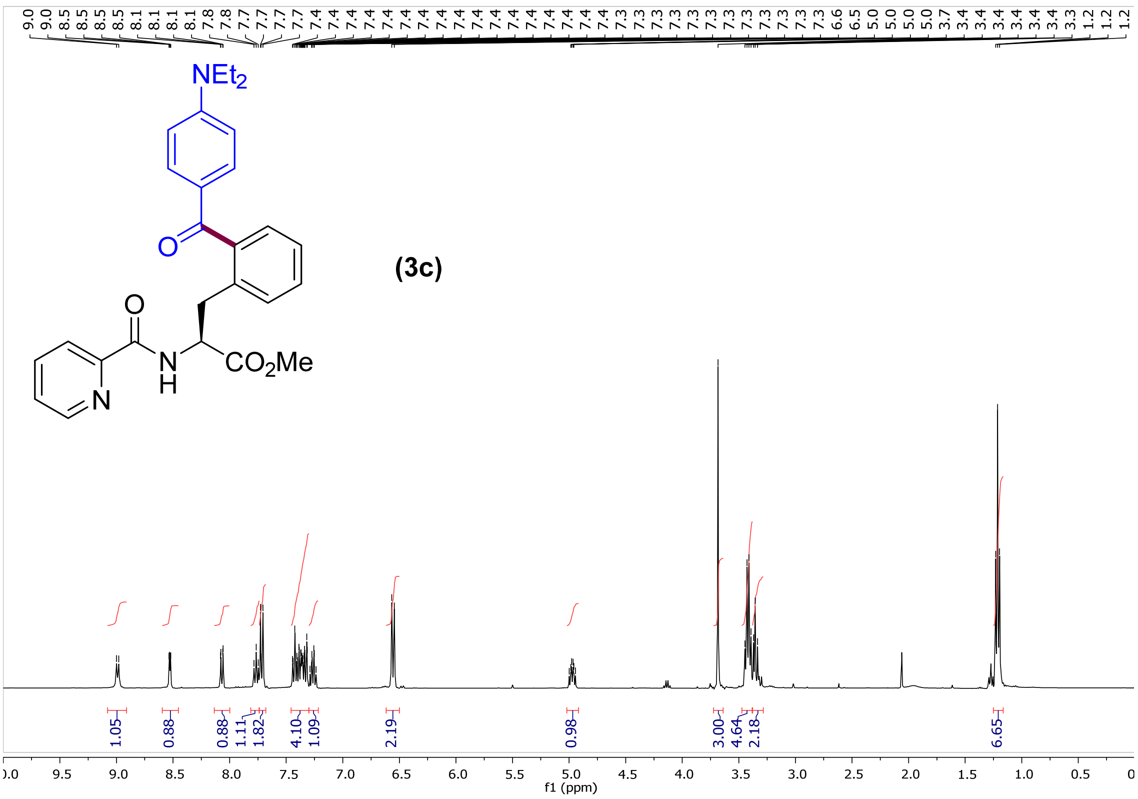

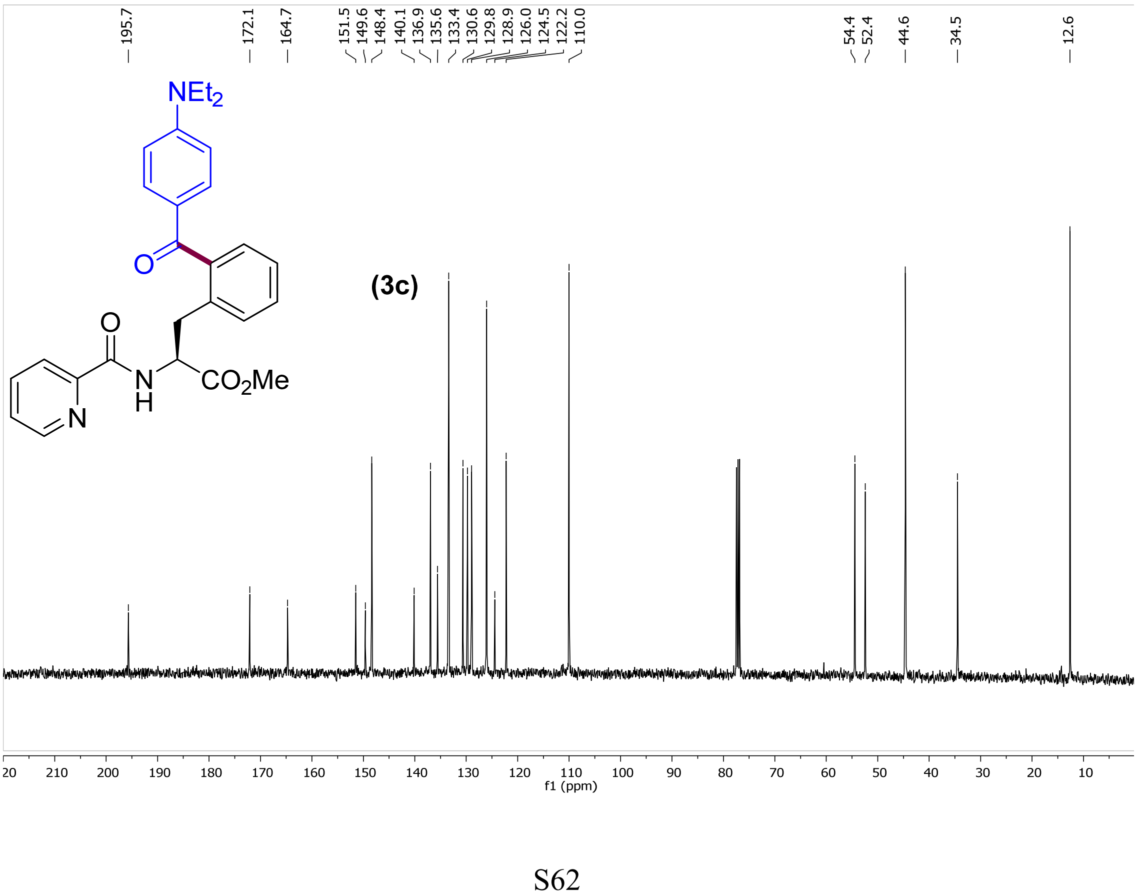

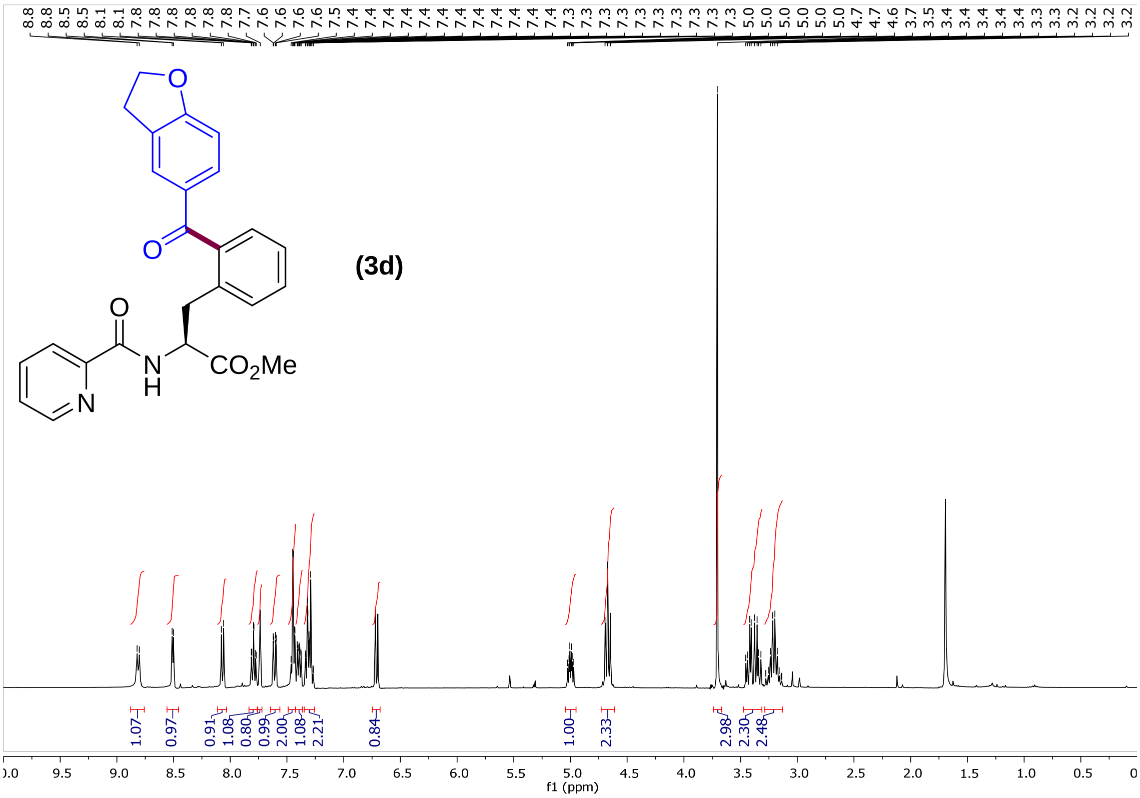

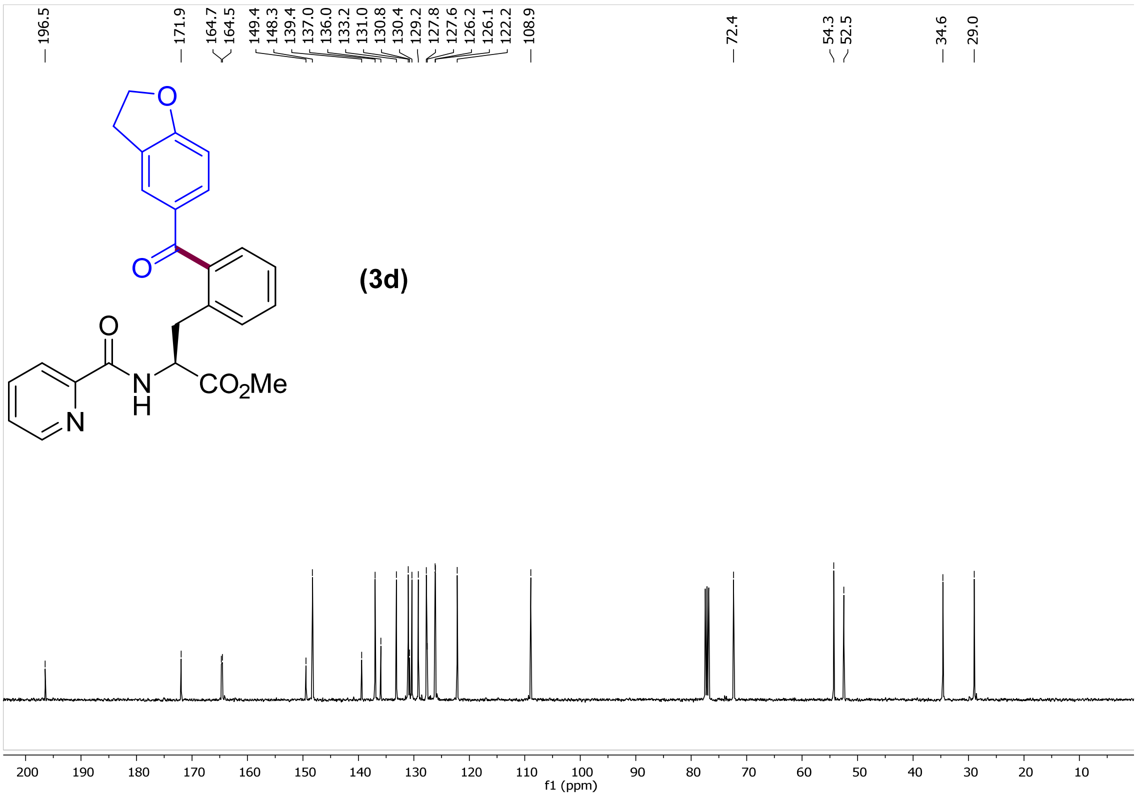

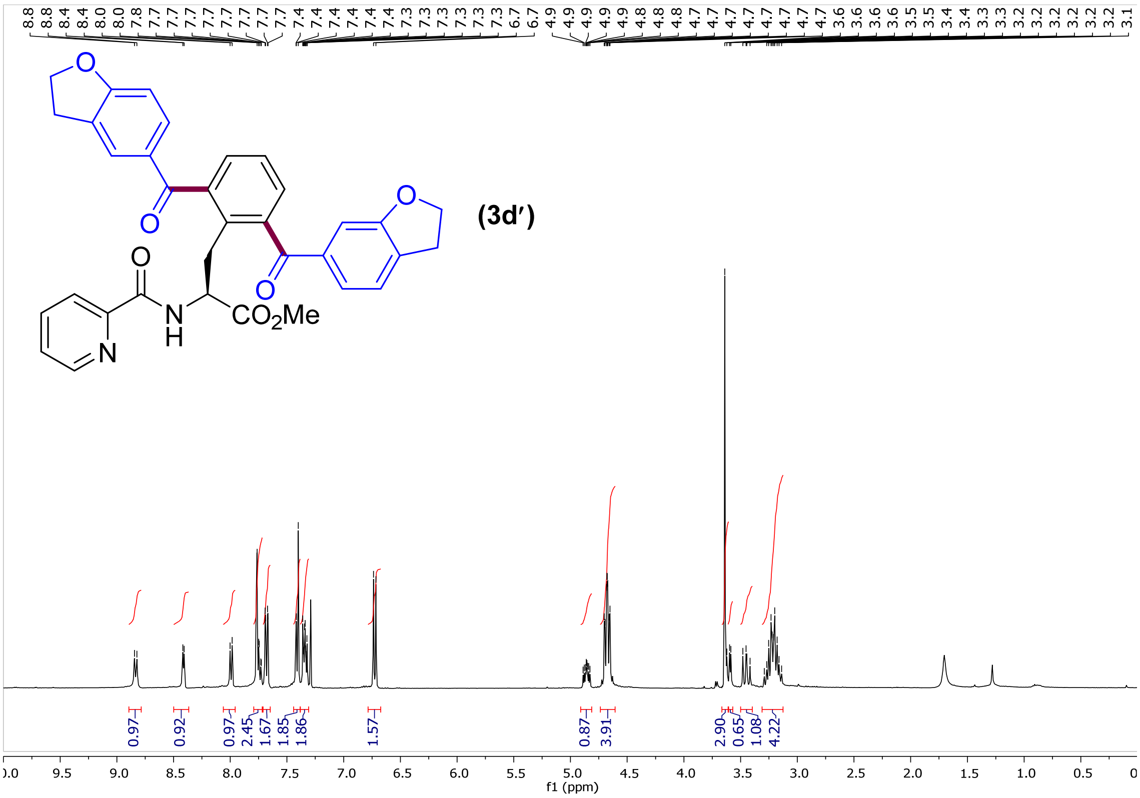

(3d′)

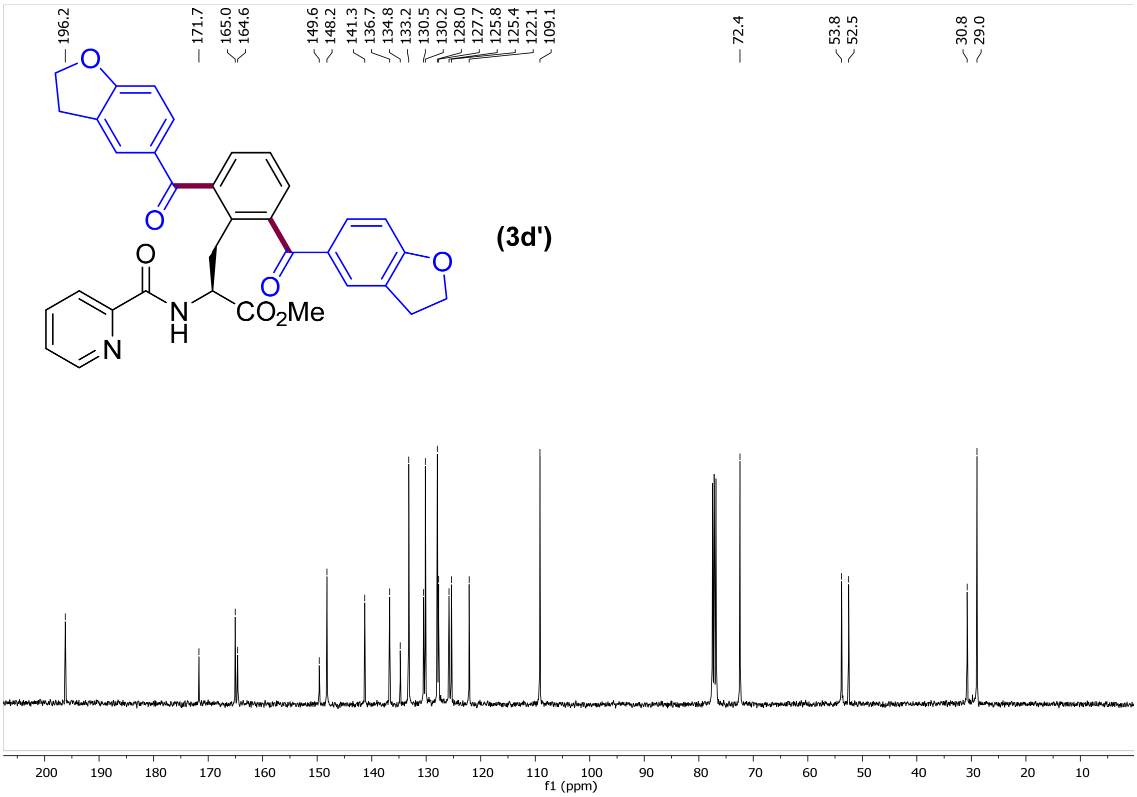

(3d')

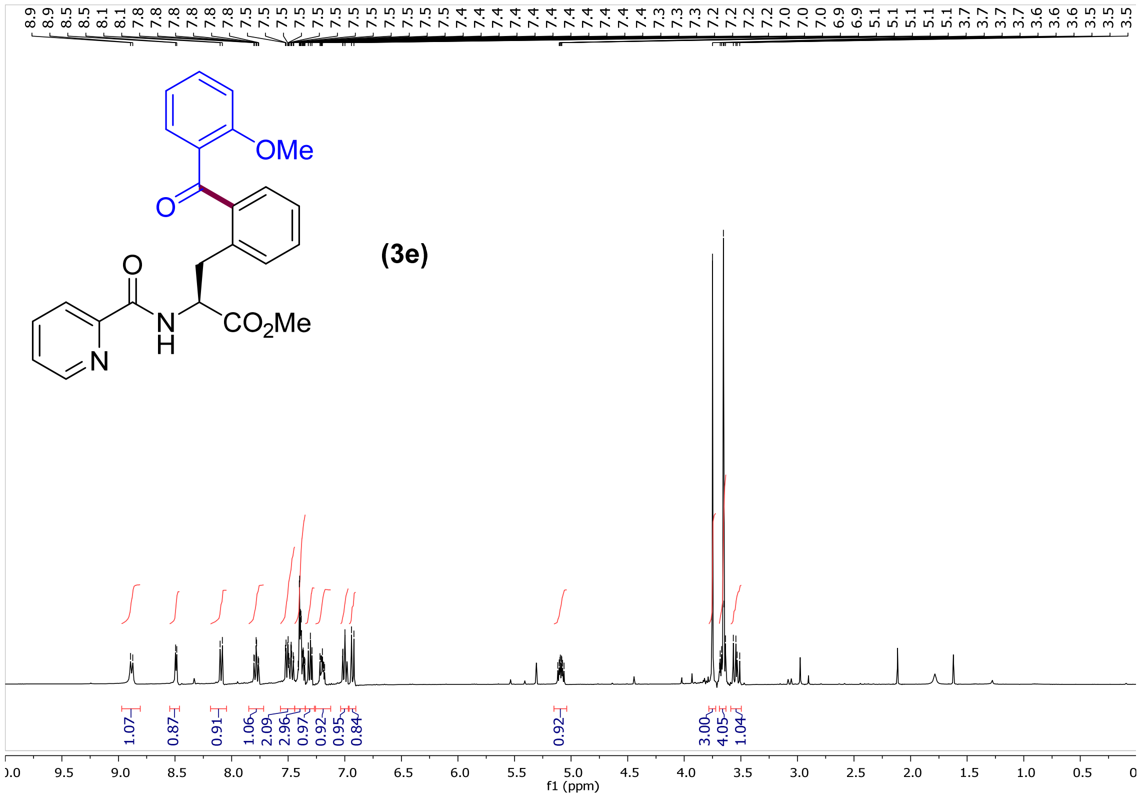

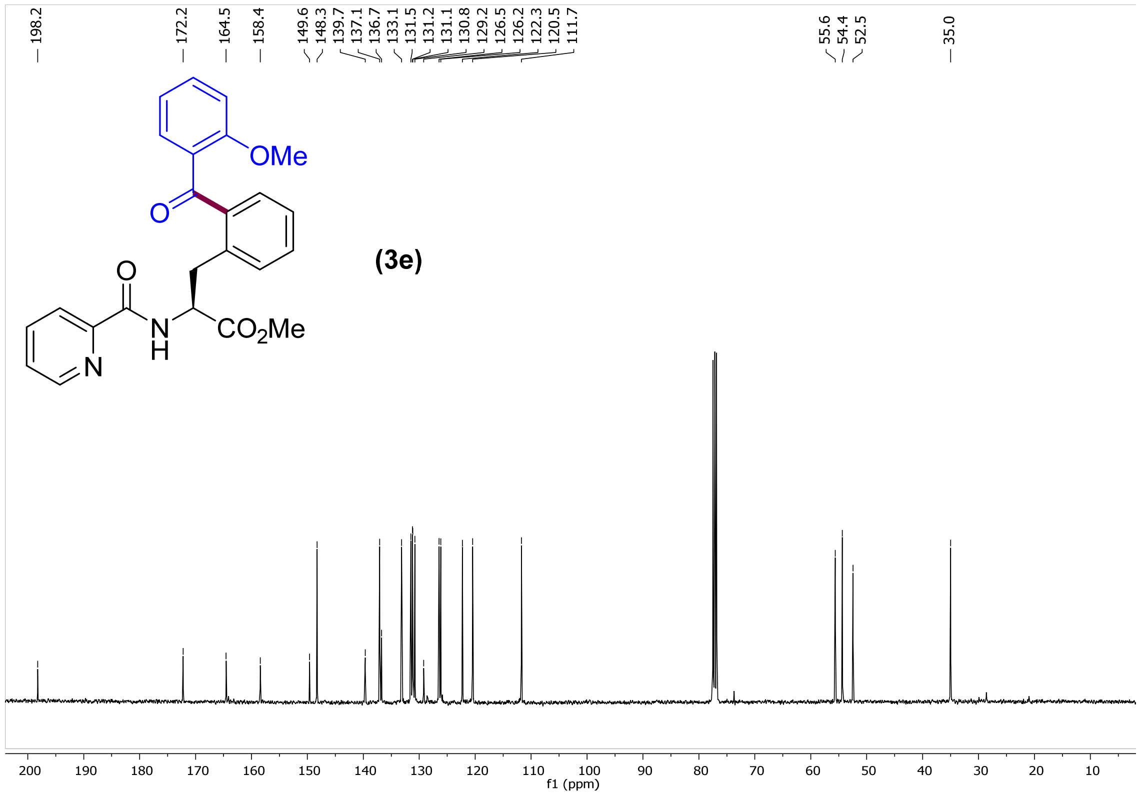

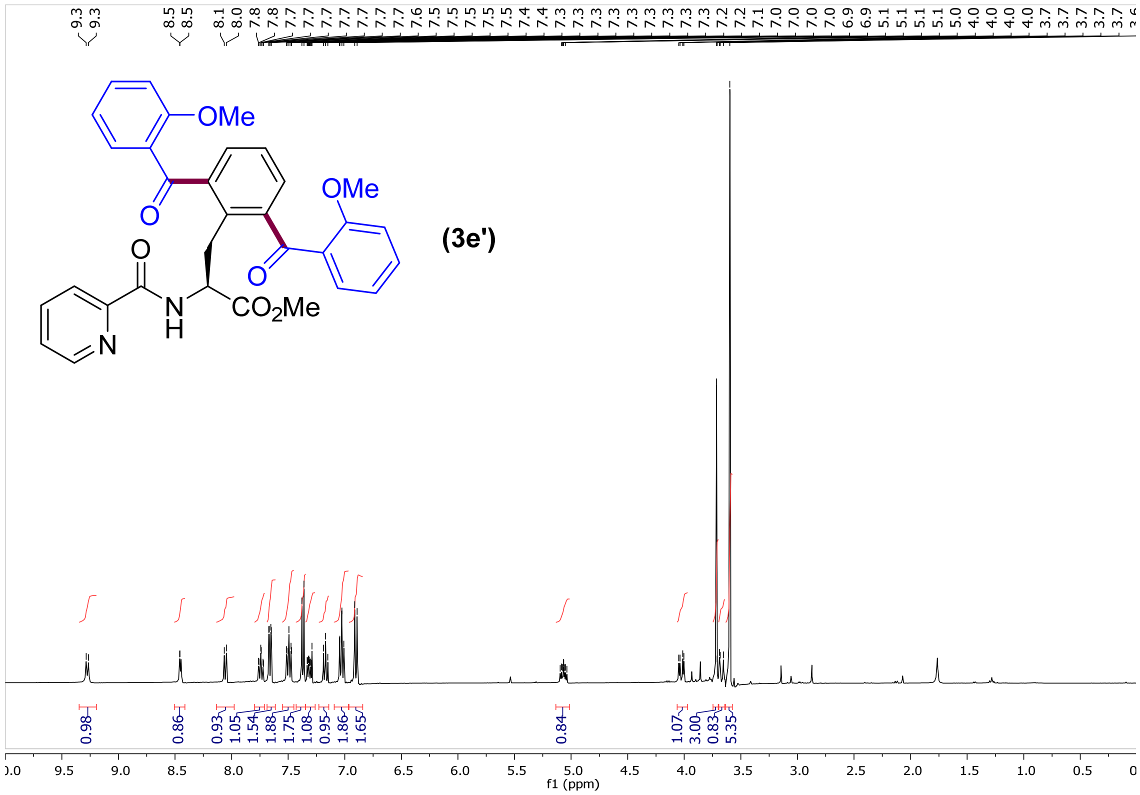

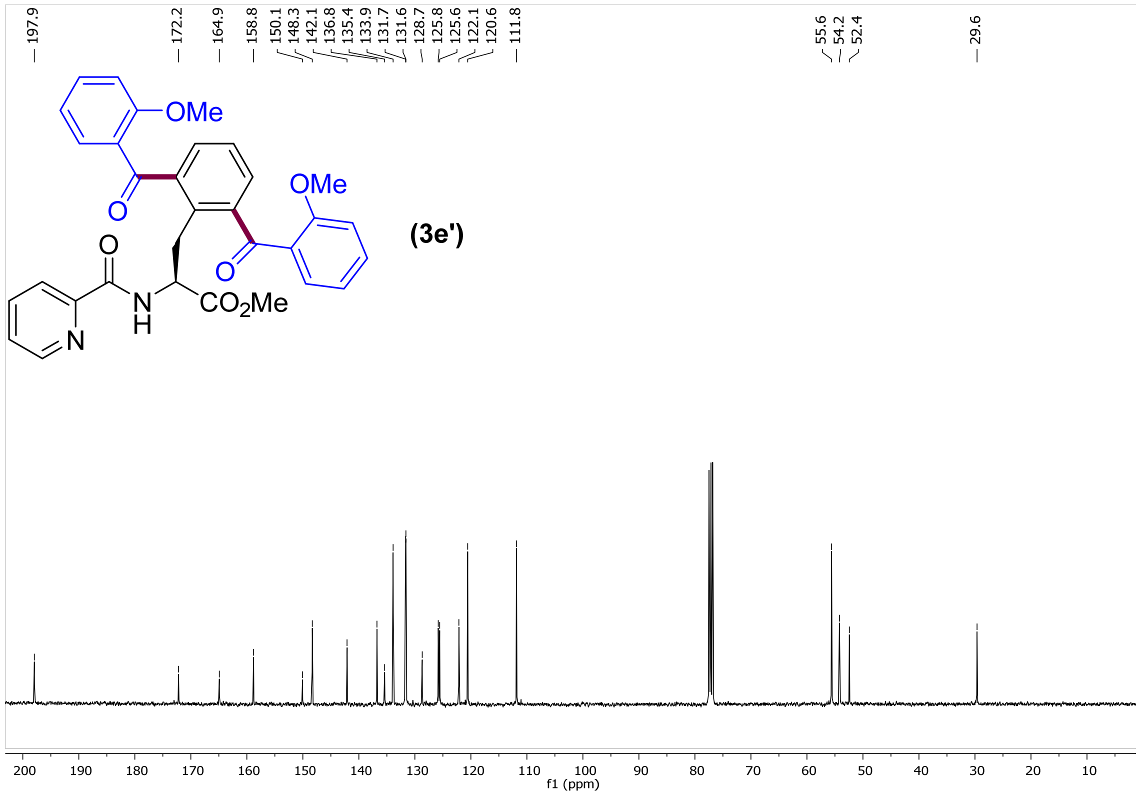

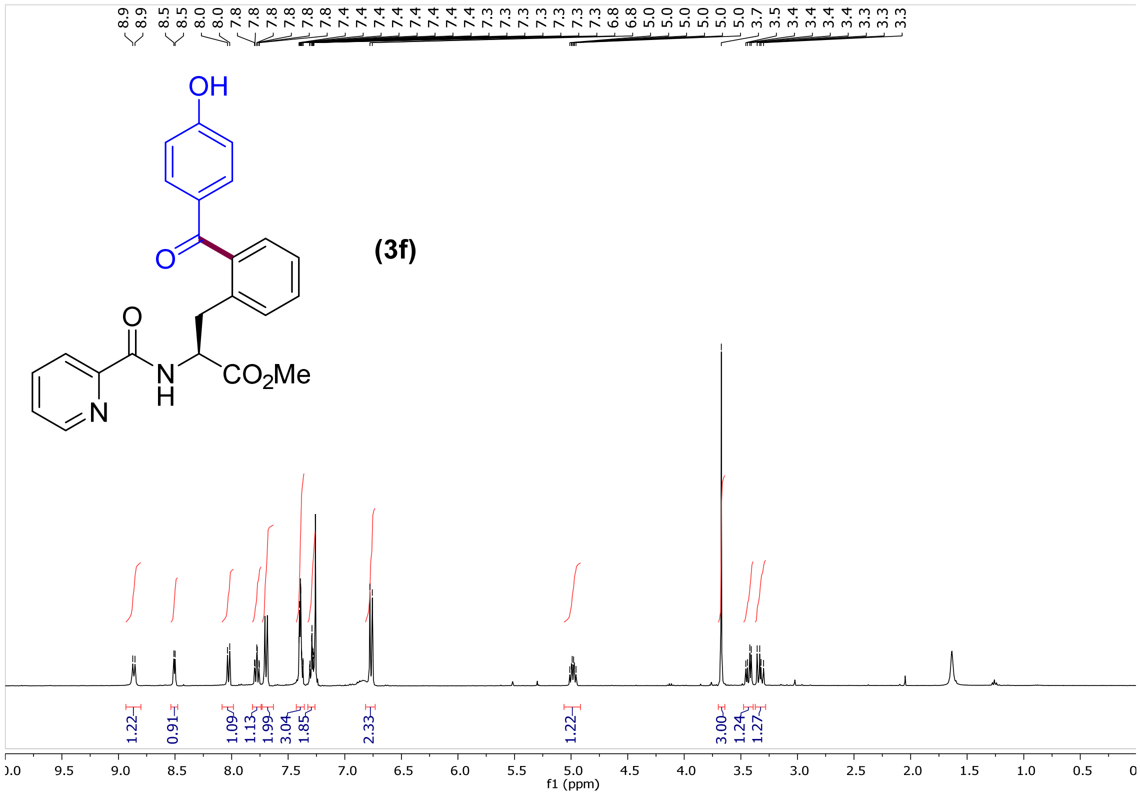

(3f)
f1 (ppm)

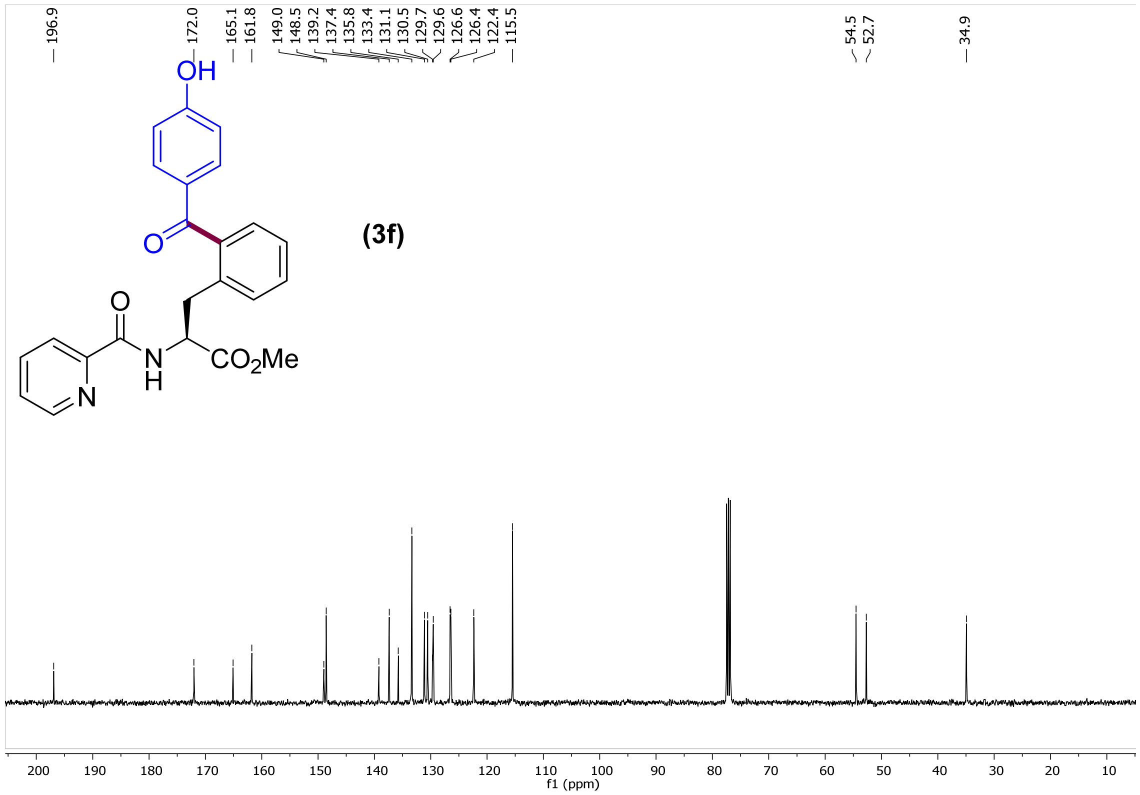

(3f)
f1 (ppm)

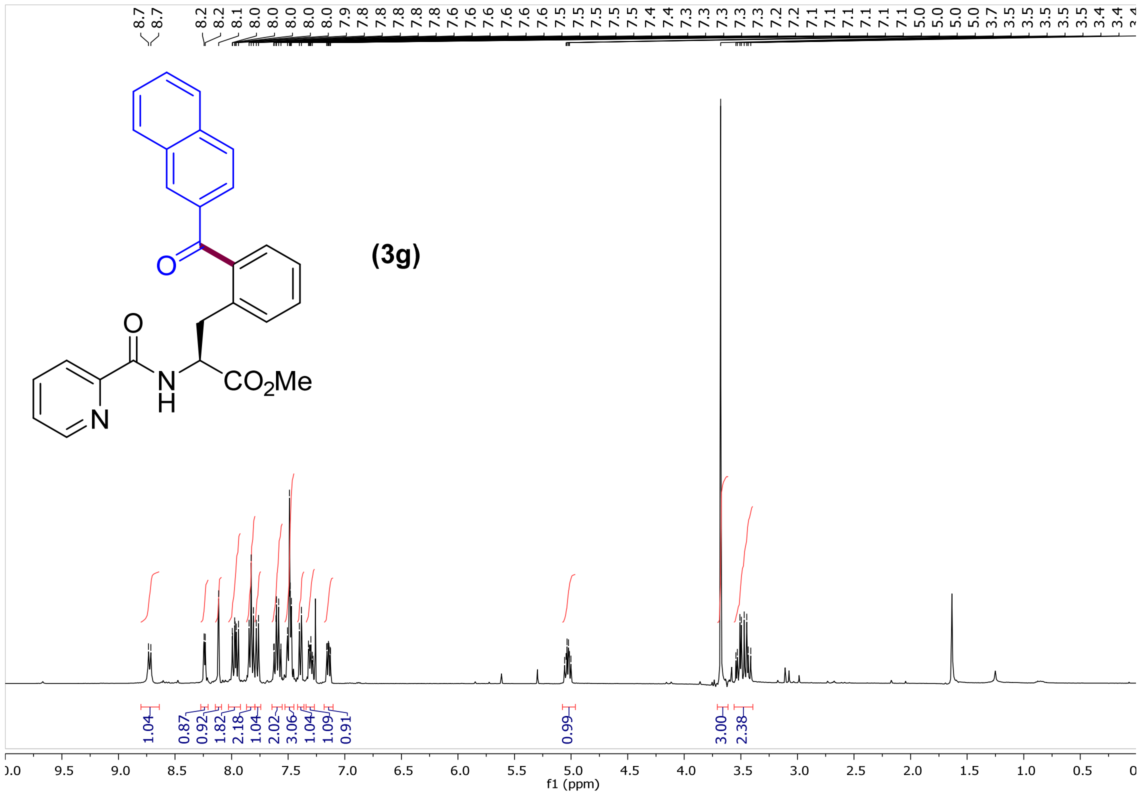

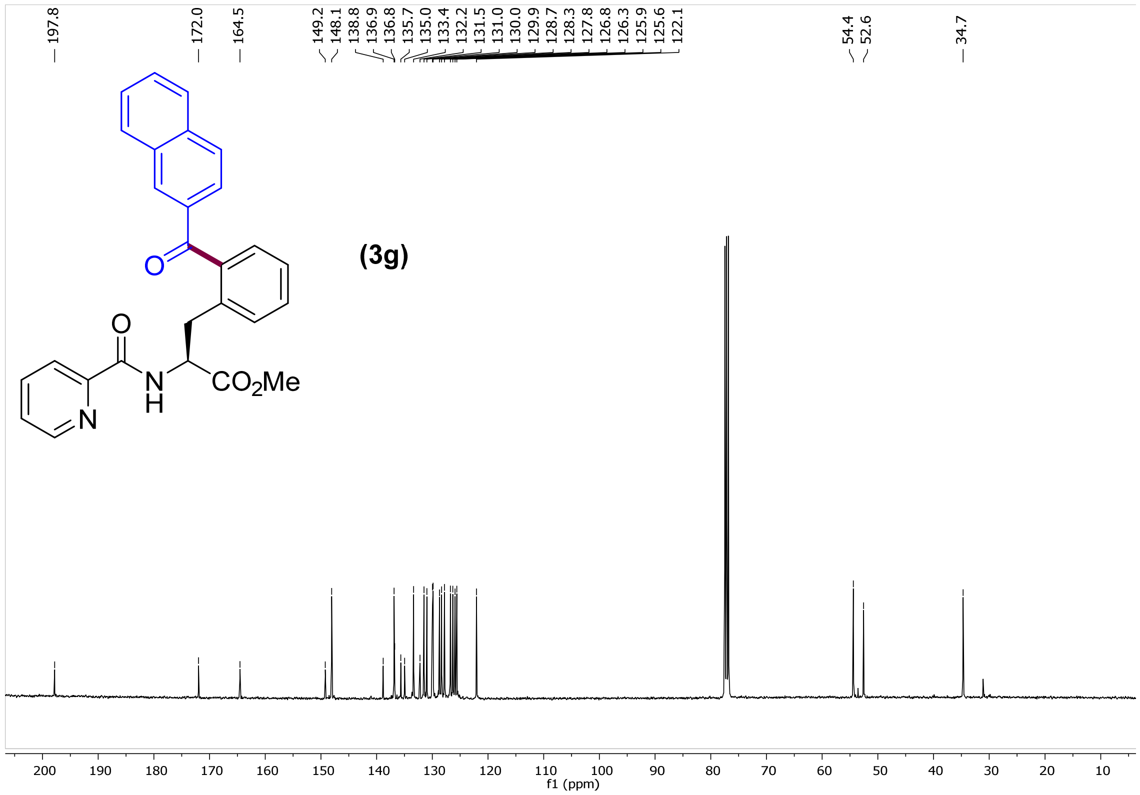

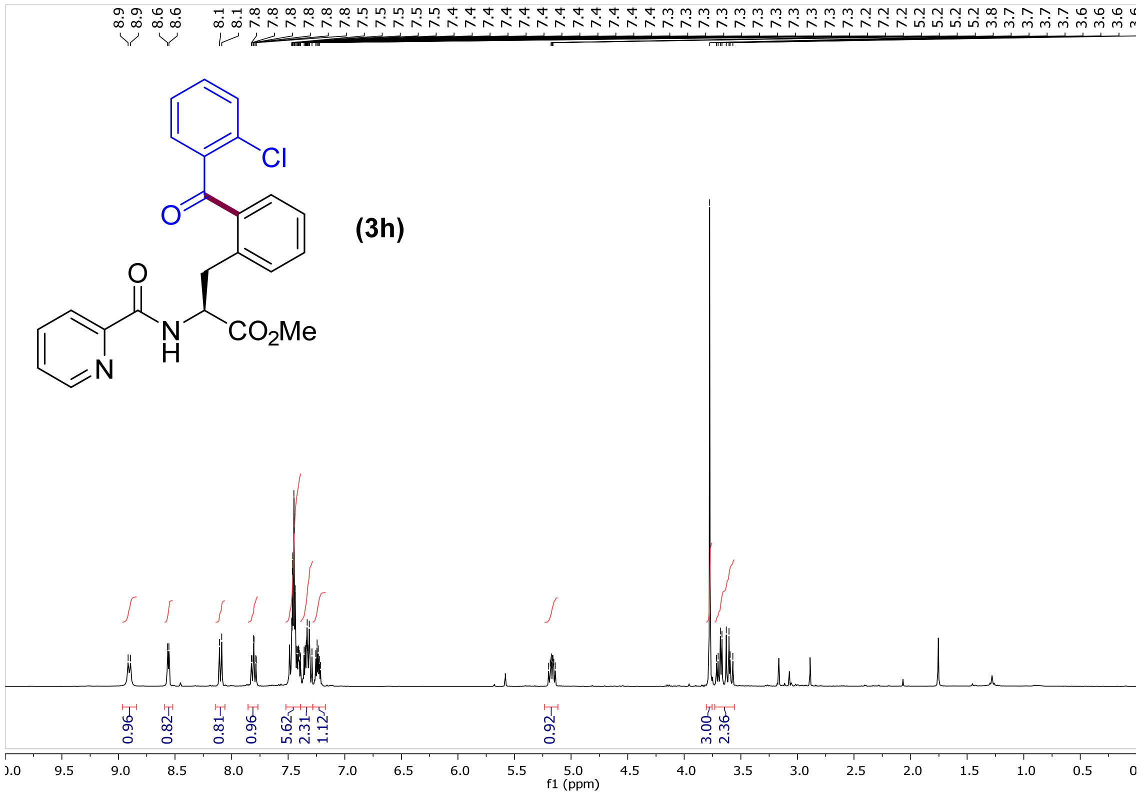

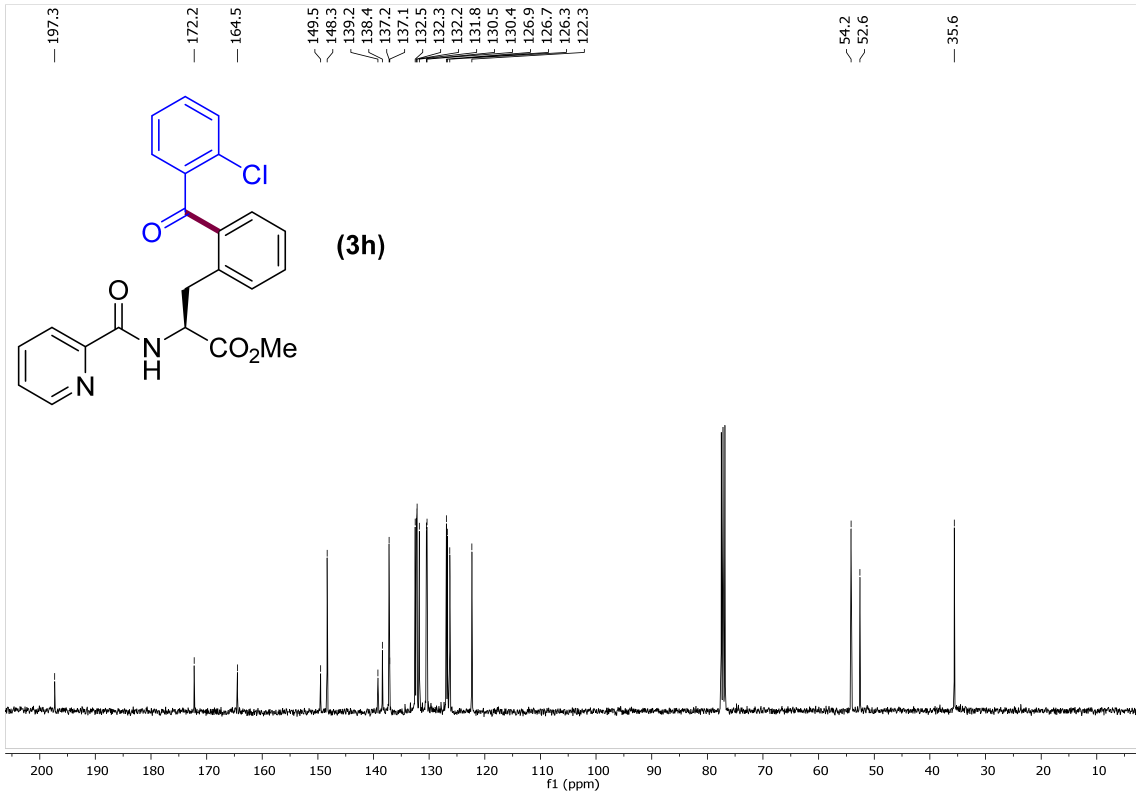

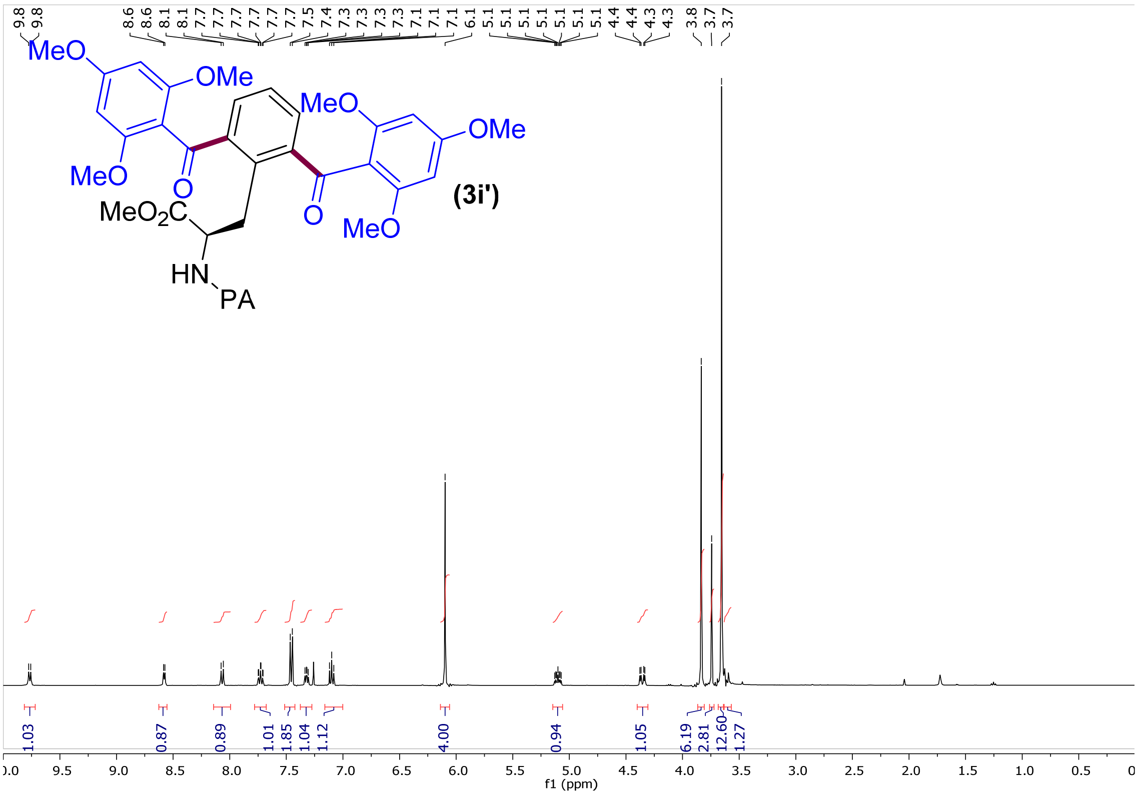
MeO
OMe
MeO
OMe
MeO
MeO
O
O
MeO_2C
HN
PA
(3i')
1.03
0.87
0.89
1.01
1.85
1.04
1.12
4.00
0.94
1.05
6.19
2.81
12.60
1.27
f1 (ppm)

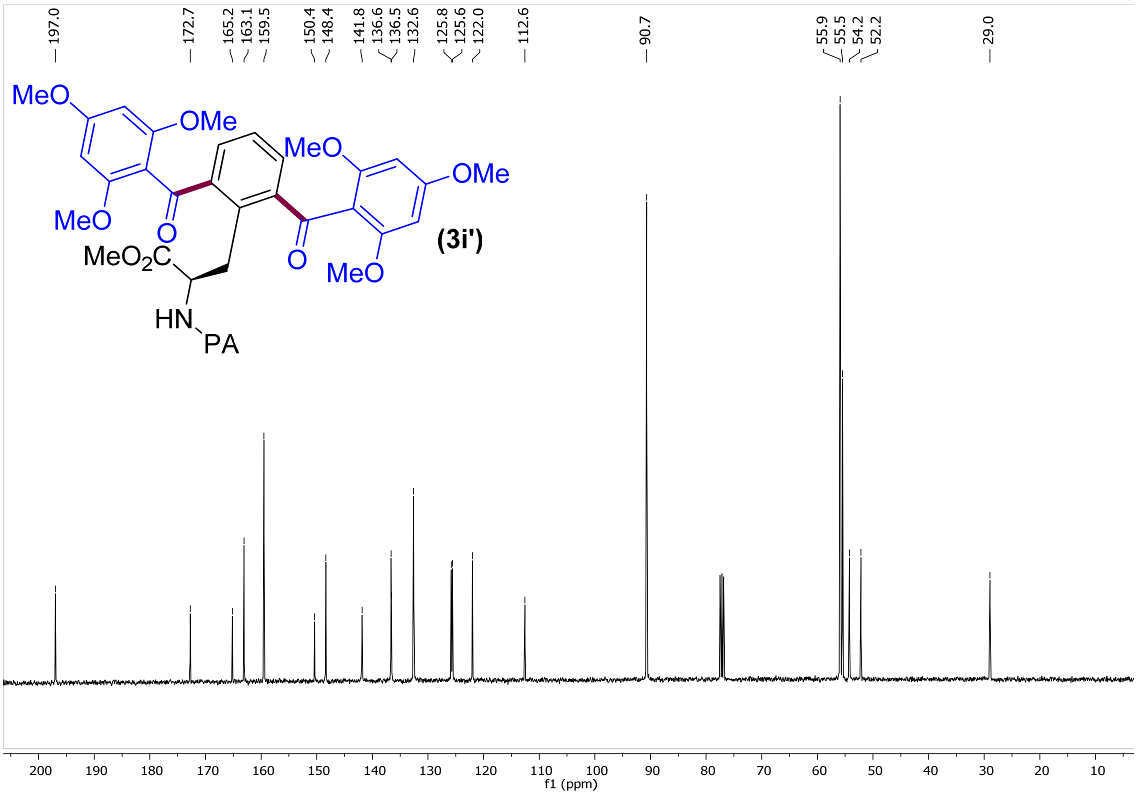
197.0
172.7
165.2
163.1
159.5
150.4
148.4
141.8
136.6
136.5
132.6
125.8
125.6
122.0
112.6
90.7
55.9
55.5
54.2
52.2
29.0
MeO
OMe
MeO
OMe
MeO
MeO
O
O
MeO_2C
HN
PA
(3i')
f1 (ppm)

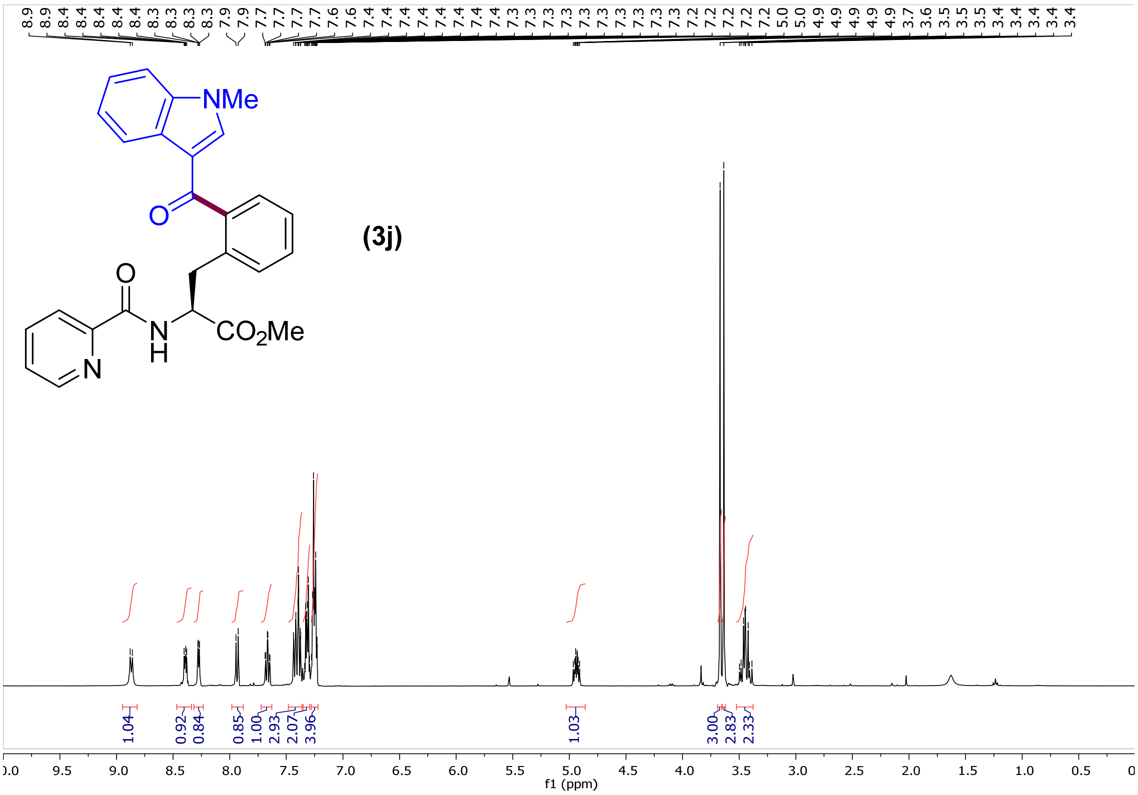

NMe
O
O
N
H
CO2Me
N
(3j)
f1 (ppm)

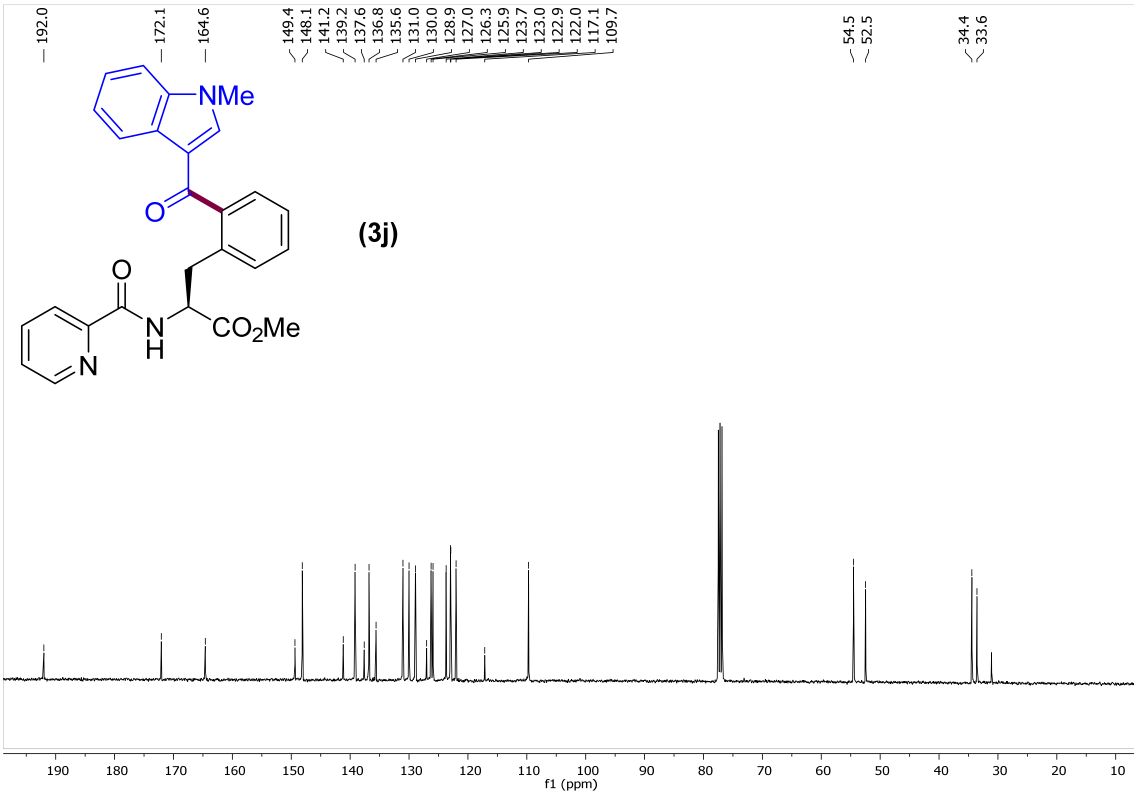

192.0
172.1
164.6
149.4
148.1
141.2
139.2
137.6
136.8
135.6
131.0
130.0
128.9
127.0
126.3
125.9
123.7
123.0
122.9
122.0
117.1
109.7
54.5
52.5
34.4
33.6
NMe
O
O
N
H
CO2Me
N
(3j)
f1 (ppm)

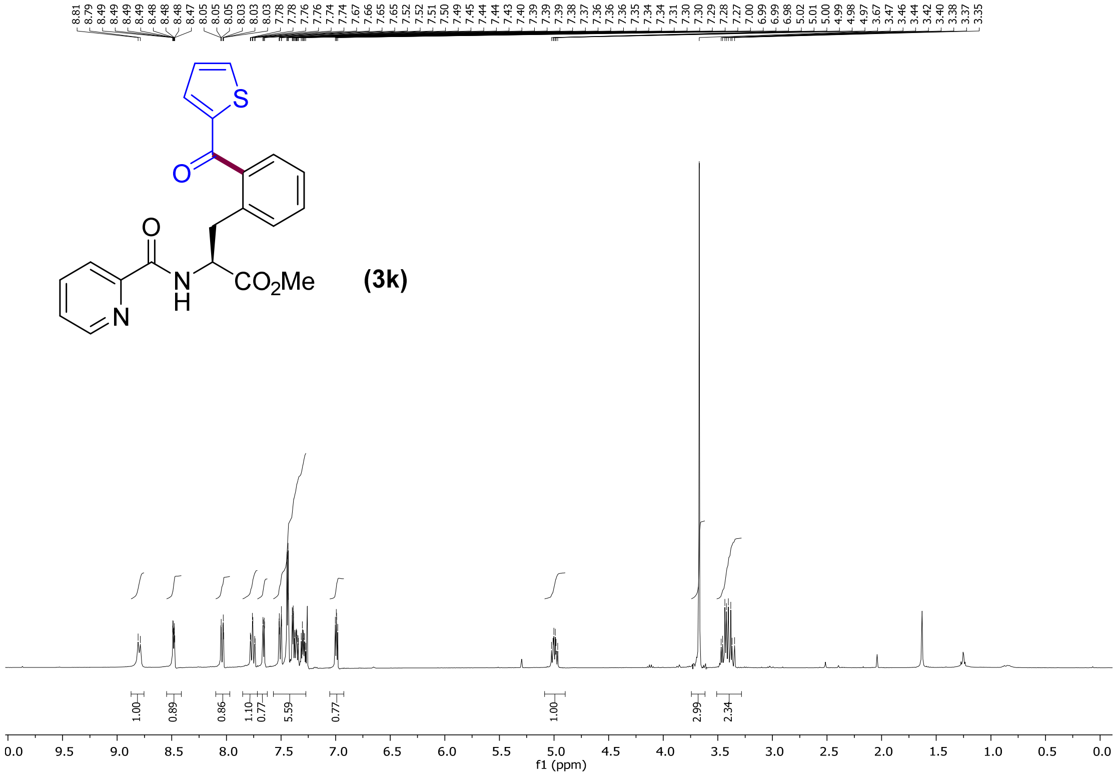
(3k)
S
O
O
N
H
CO2Me
N
1.00
0.89
0.86
1.10
0.77
5.59
0.77
1.00
2.99
2.34
f1 (ppm)

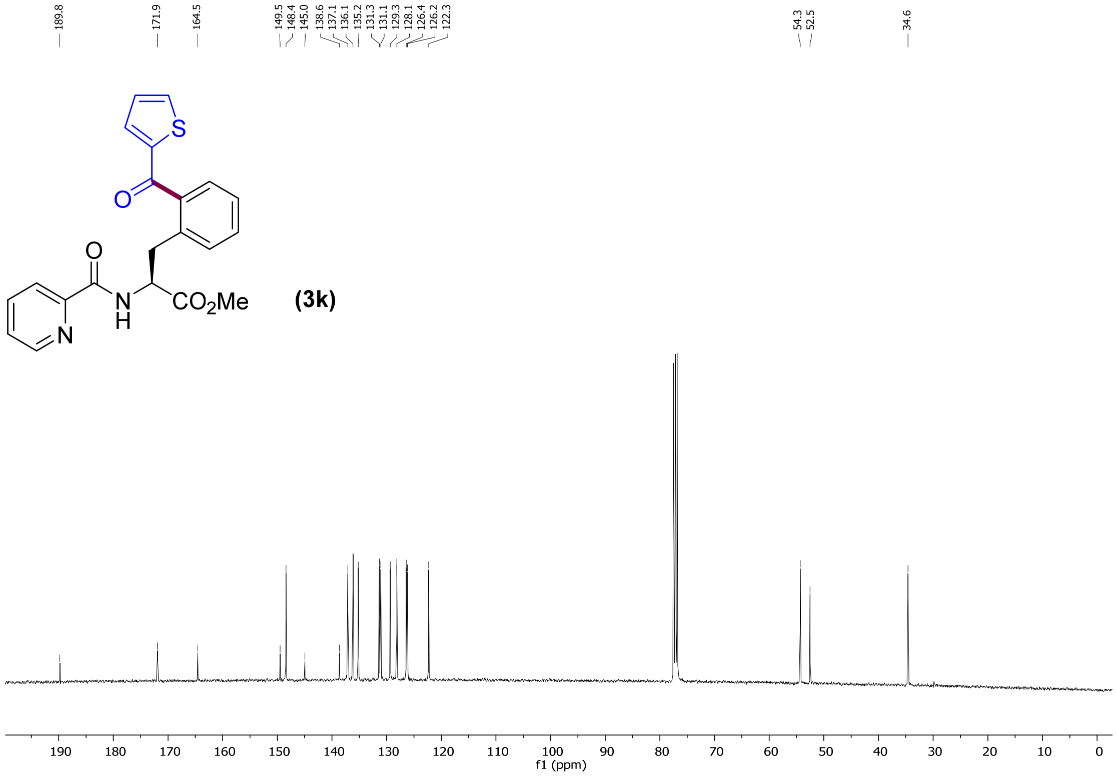
189.8
171.9
164.5
149.5
148.4
145.0
138.6
137.1
136.1
135.2
131.3
131.1
129.3
128.1
126.4
126.2
122.3
54.3
52.5
34.6
(3k)
f1 (ppm)

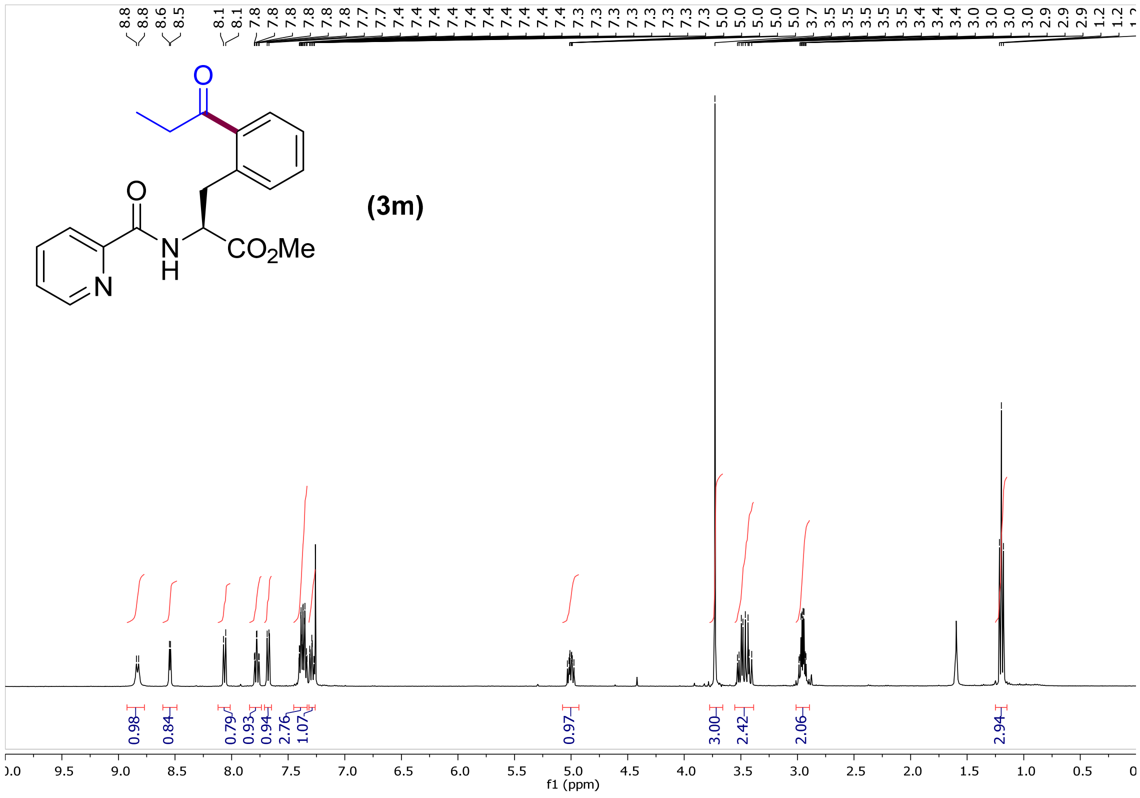

(3m)

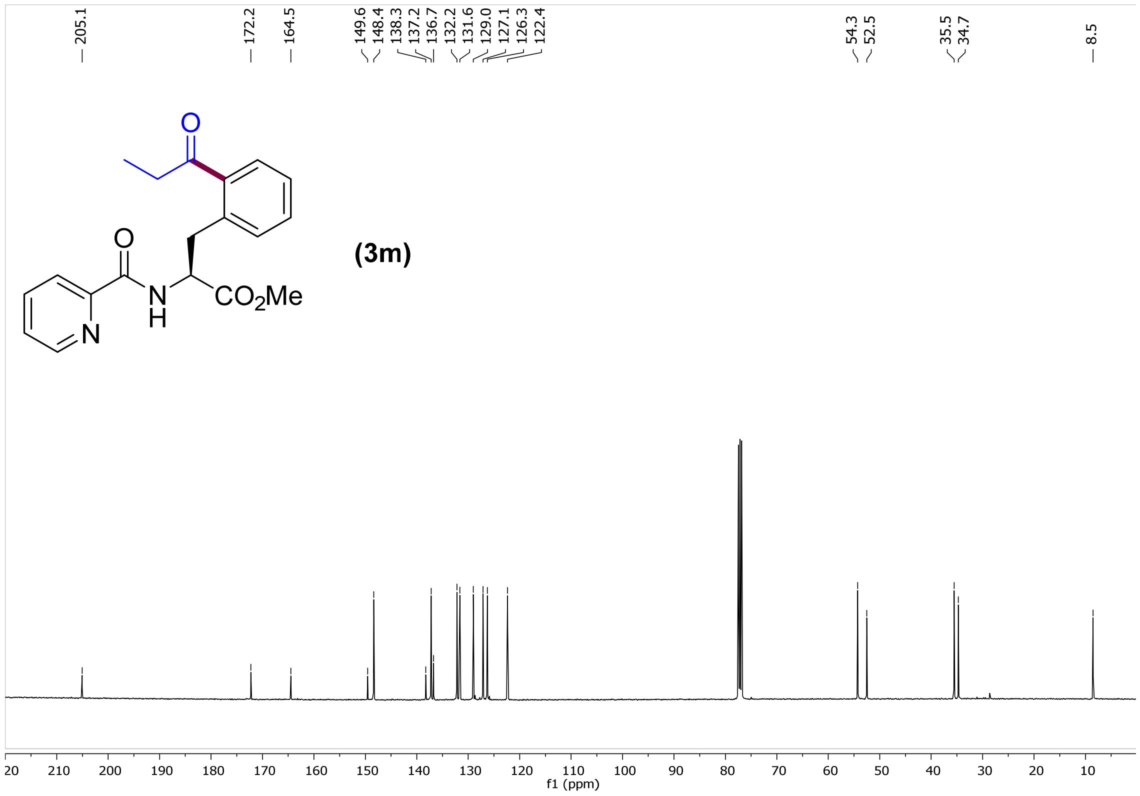

(3m)

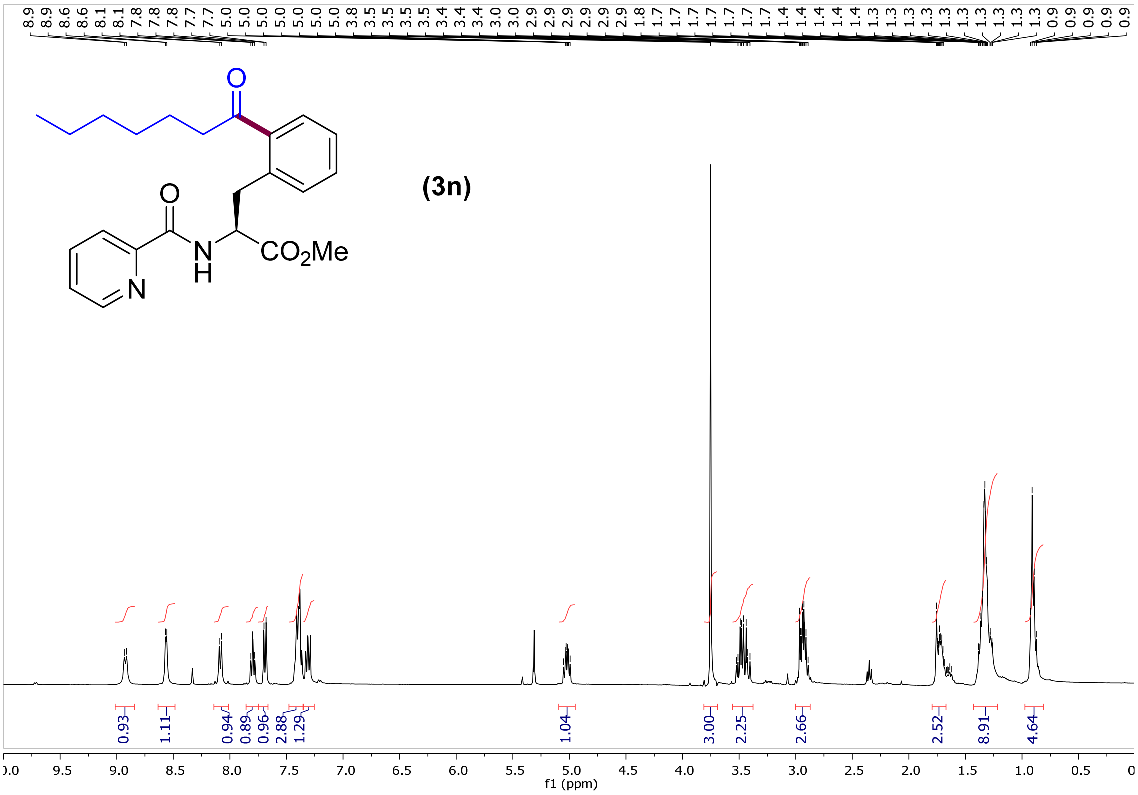

(3n)

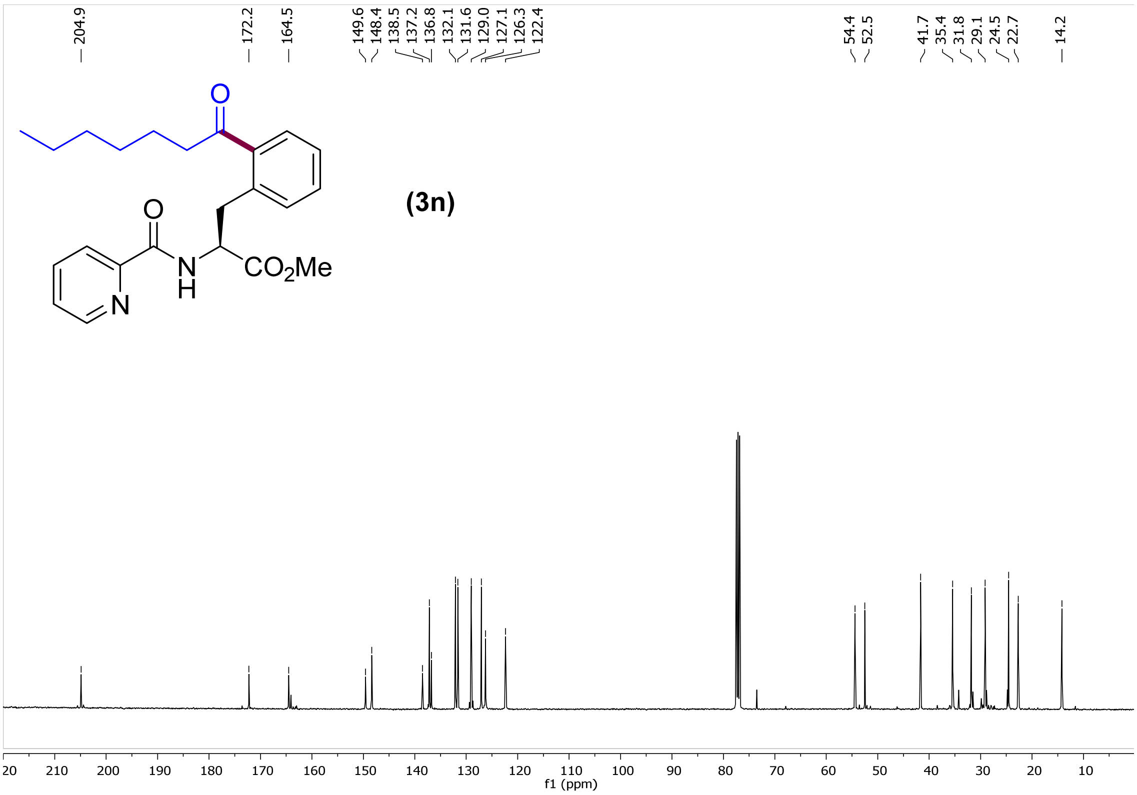

(3n)

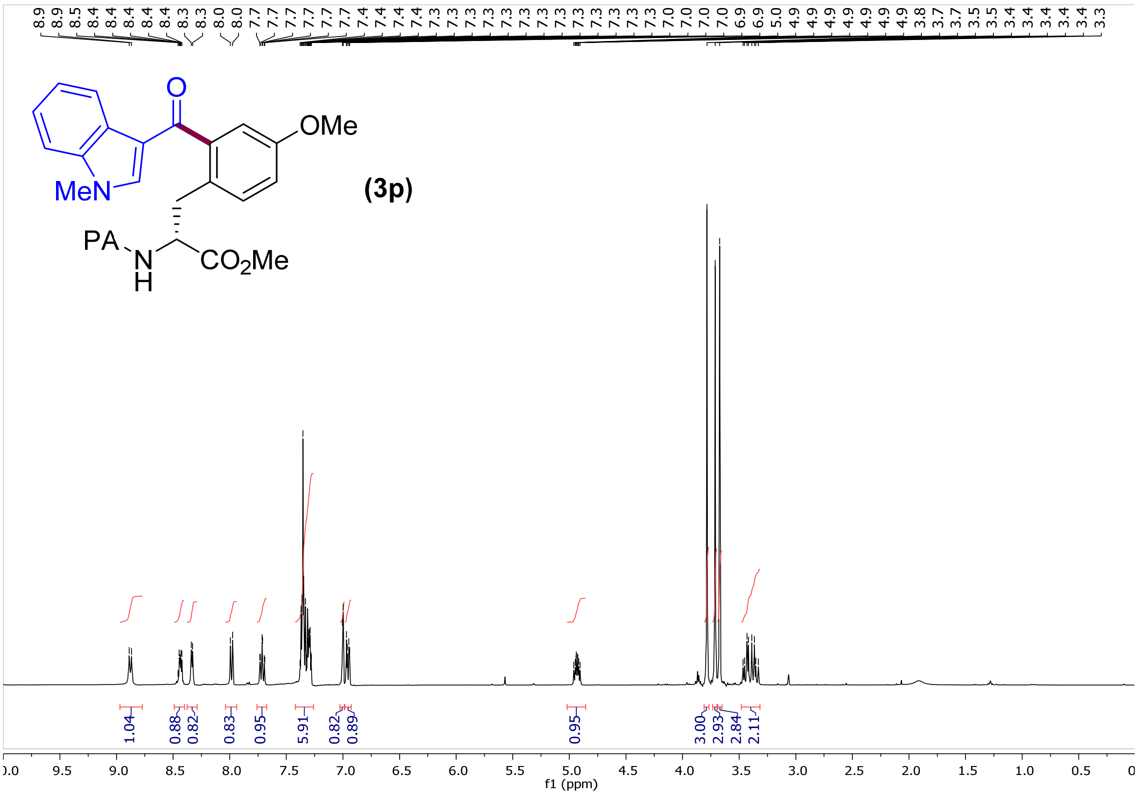

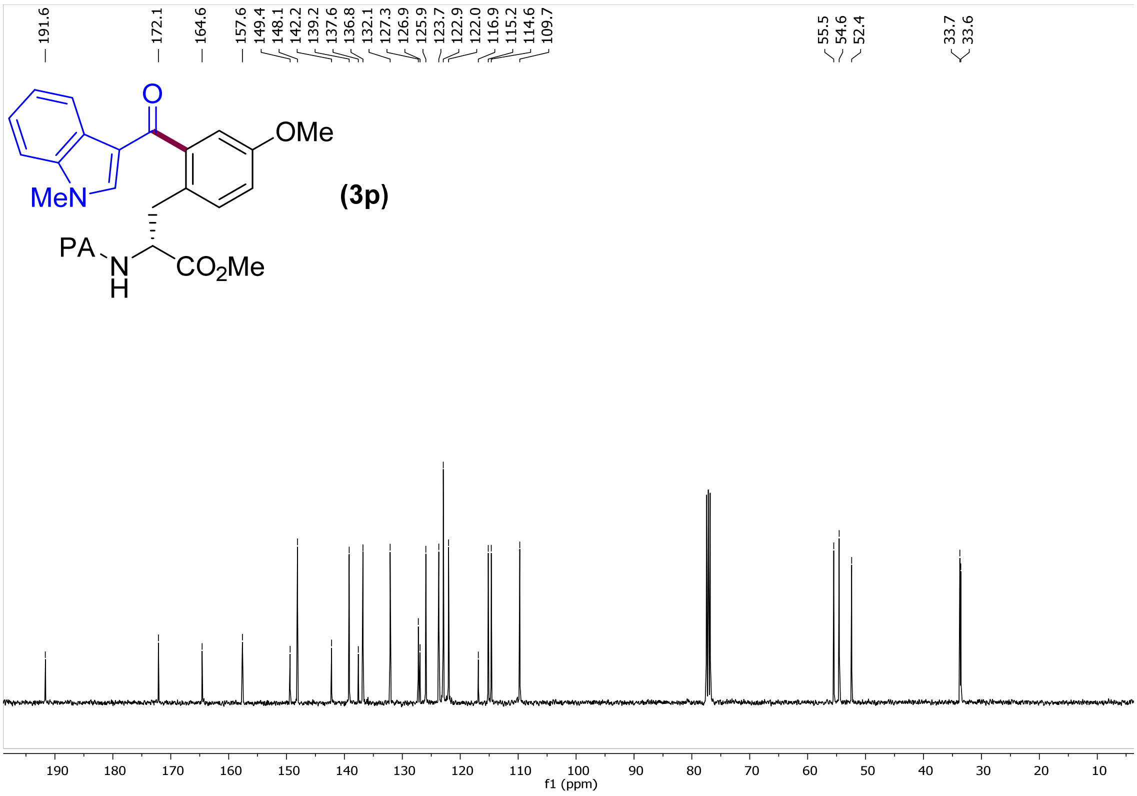

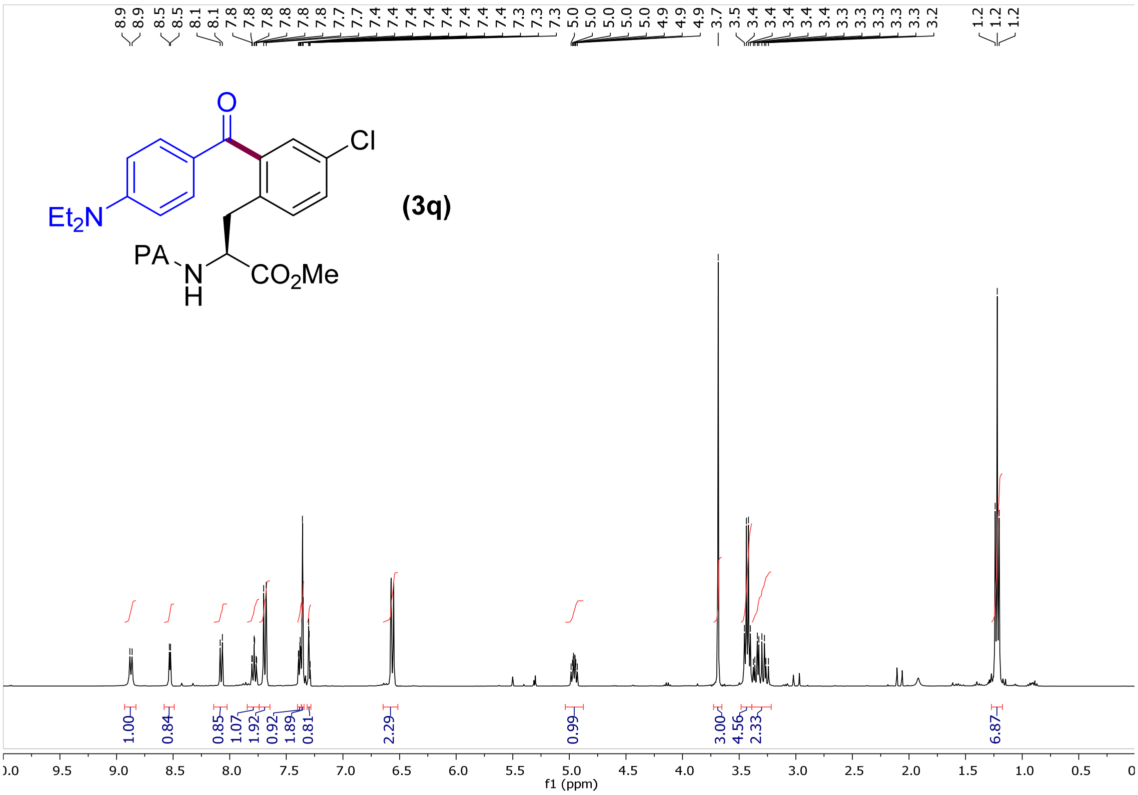

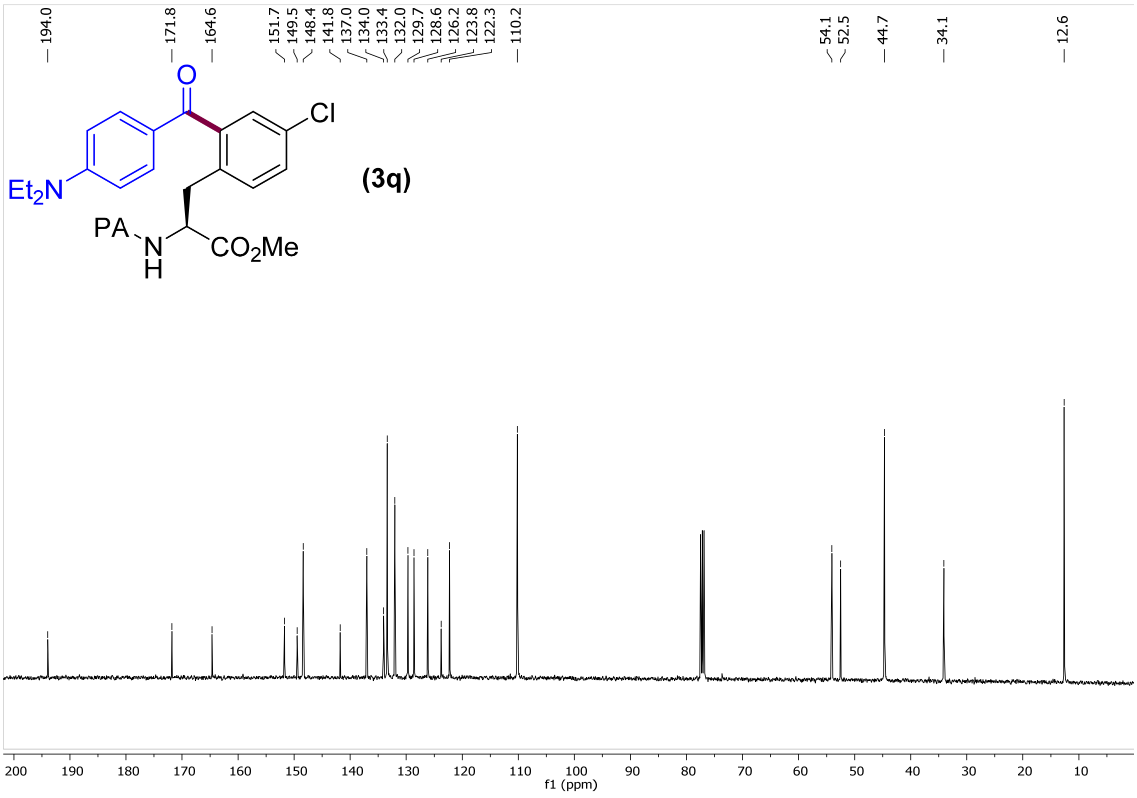

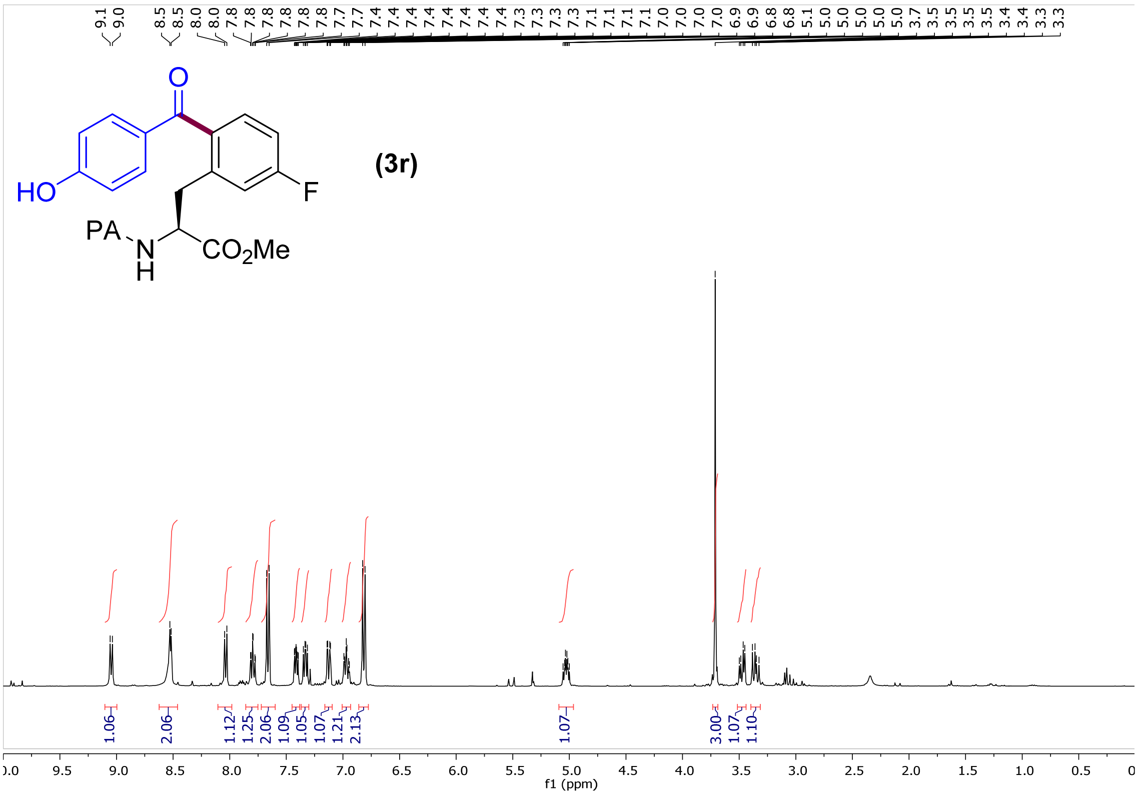
(3r)
HO
PA
N
H
CO2Me
F
O
f1 (ppm)

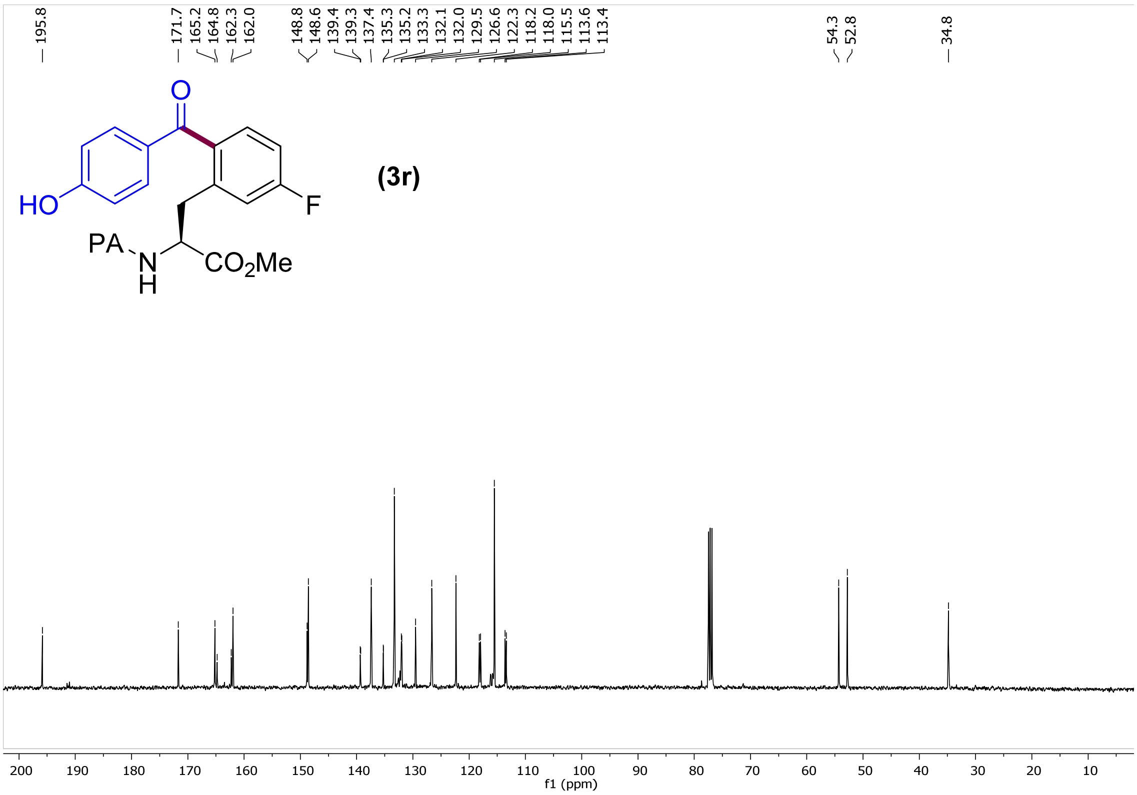
(3r)
HO
PA
N
H
CO2Me
F
O
f1 (ppm)

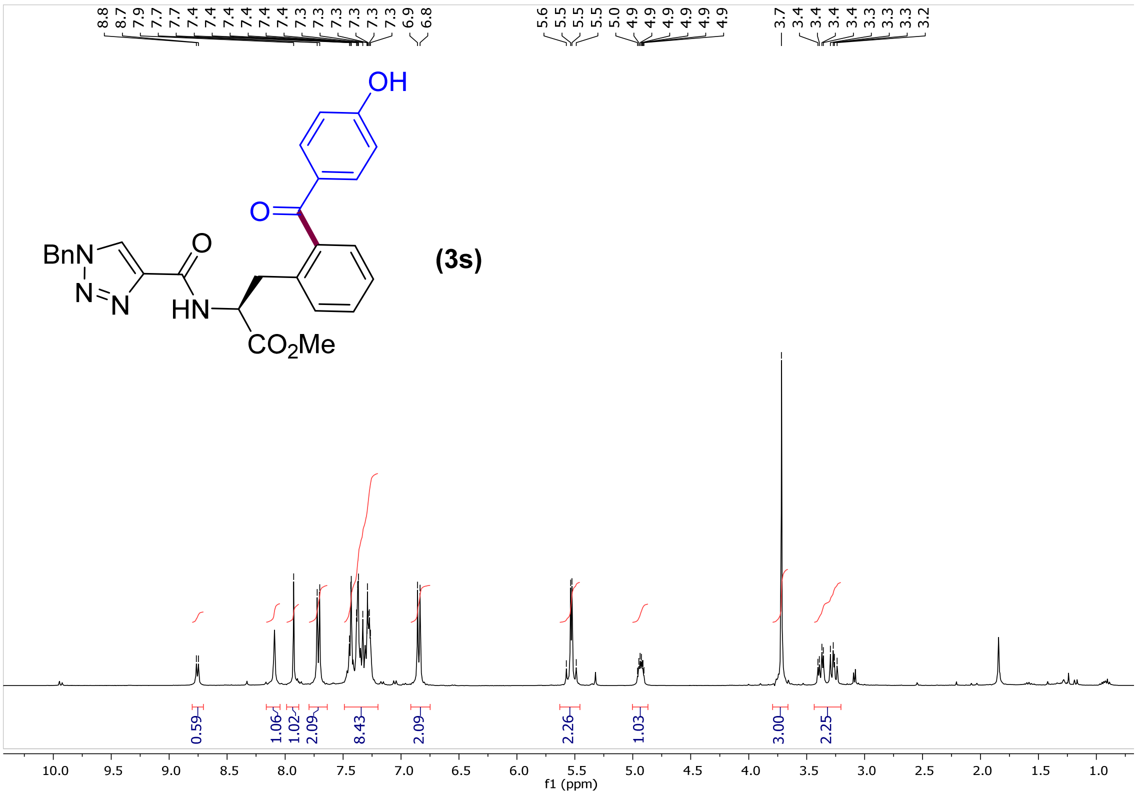
(3s)
f1 (ppm)

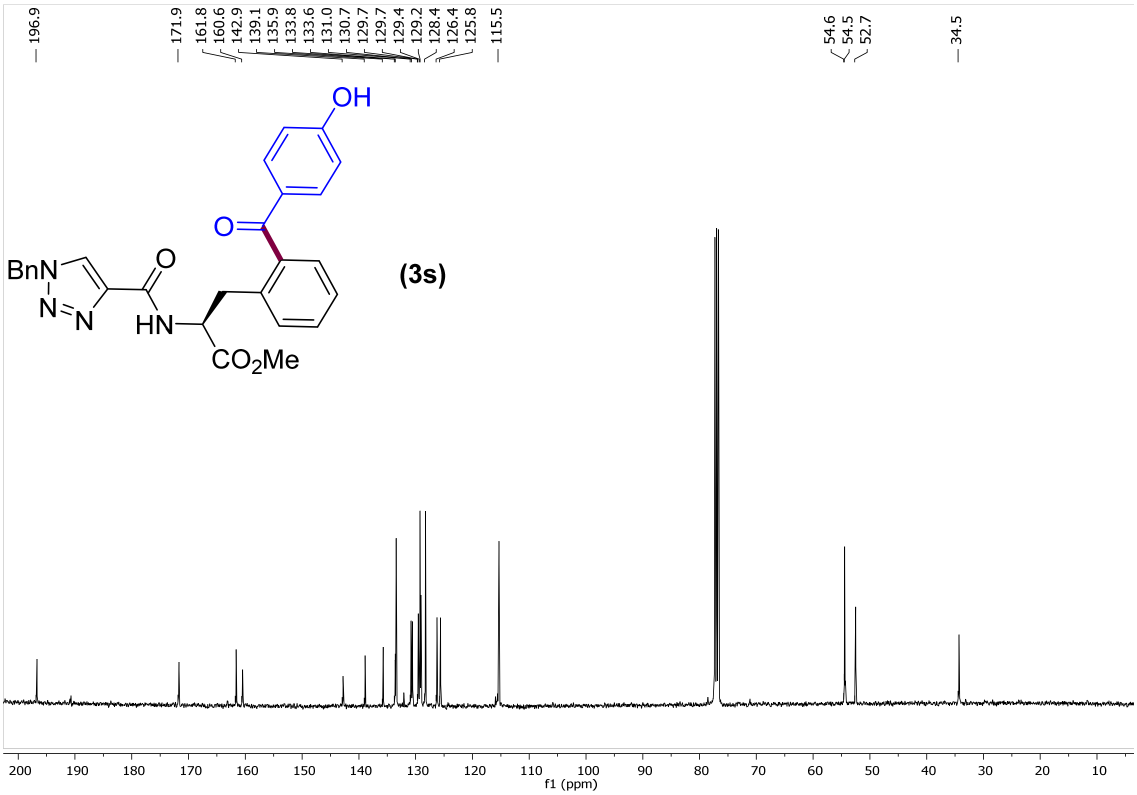
(3s)
f1 (ppm)

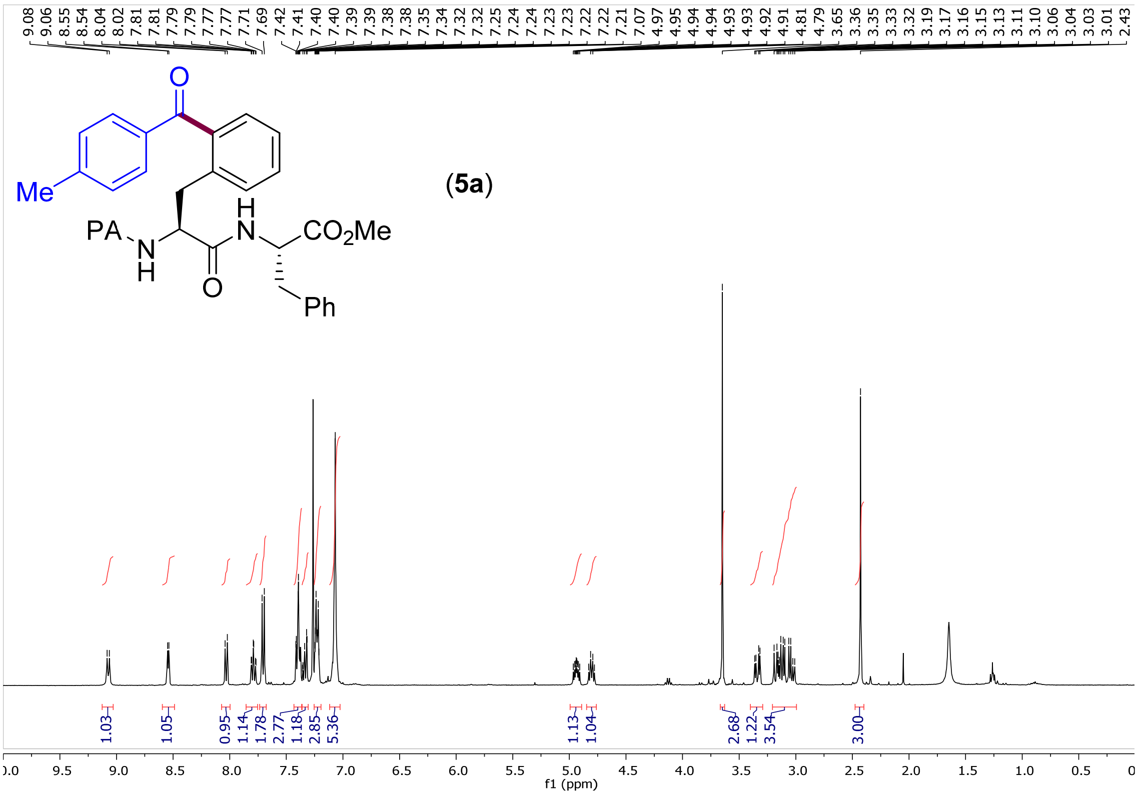

(5a)
f1 (ppm)

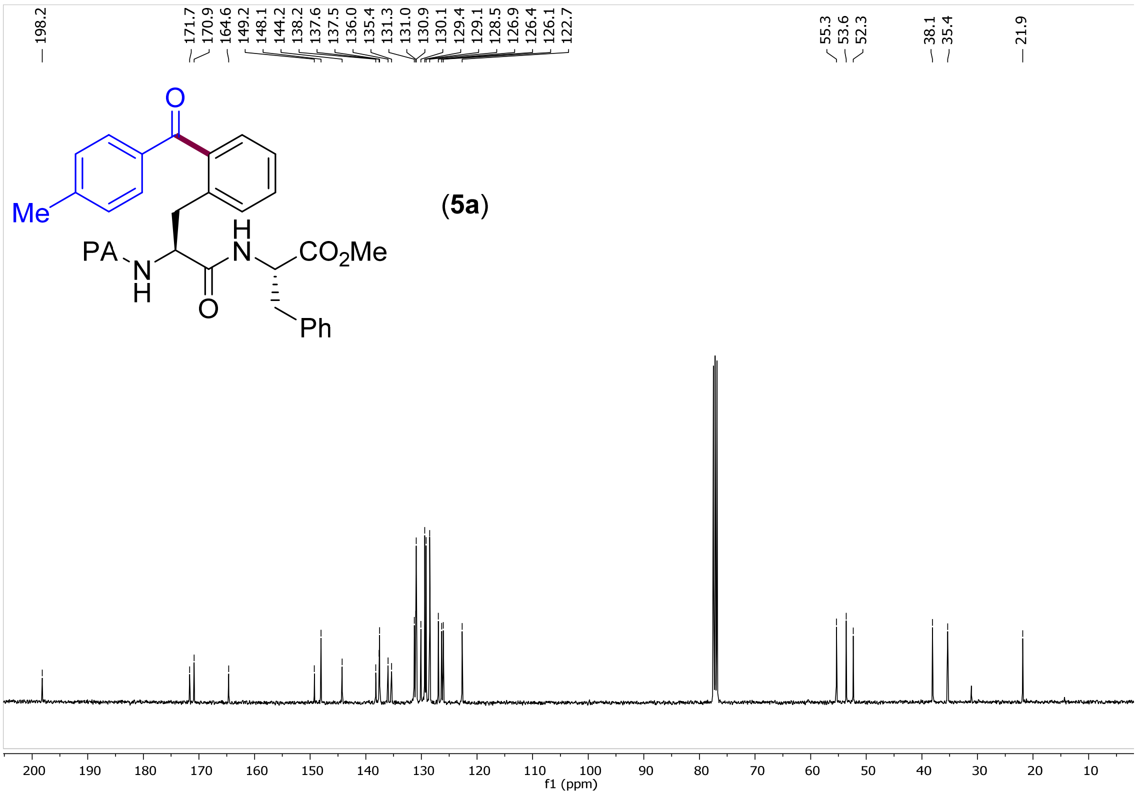

(5a)
f1 (ppm)

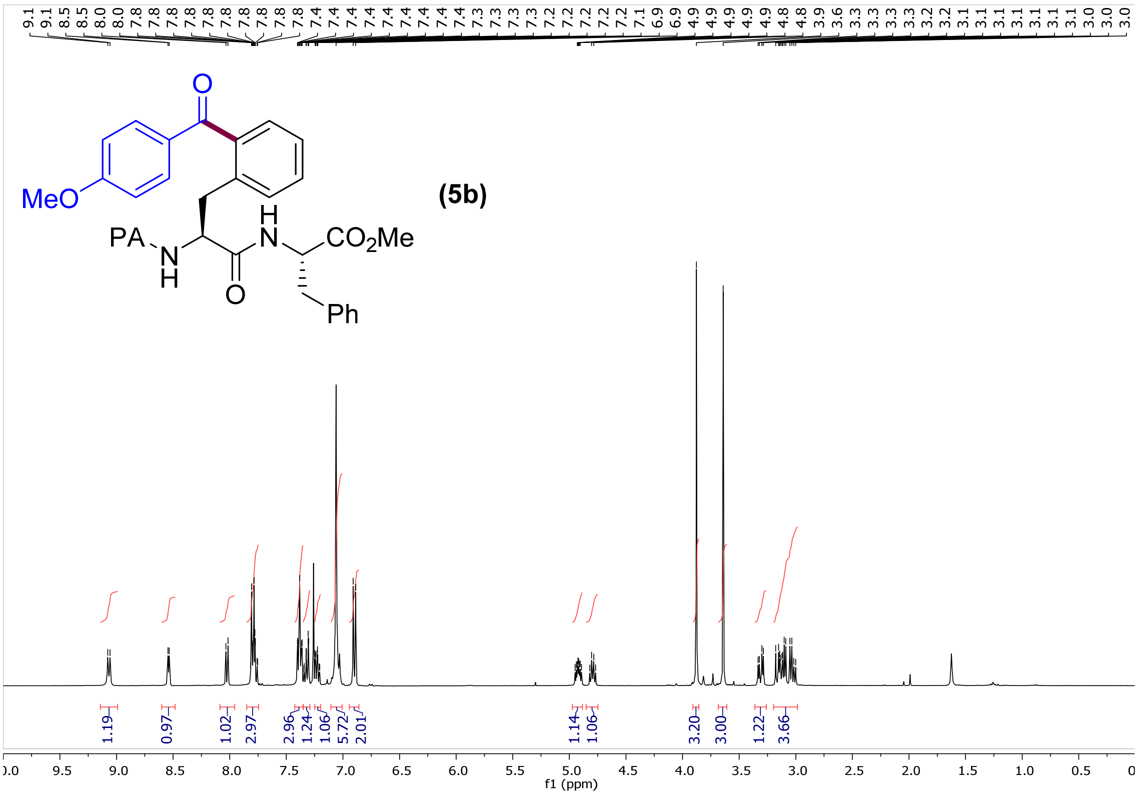

MeO
PA
N
H
O
CO2Me
Ph
(5b)
f1 (ppm)

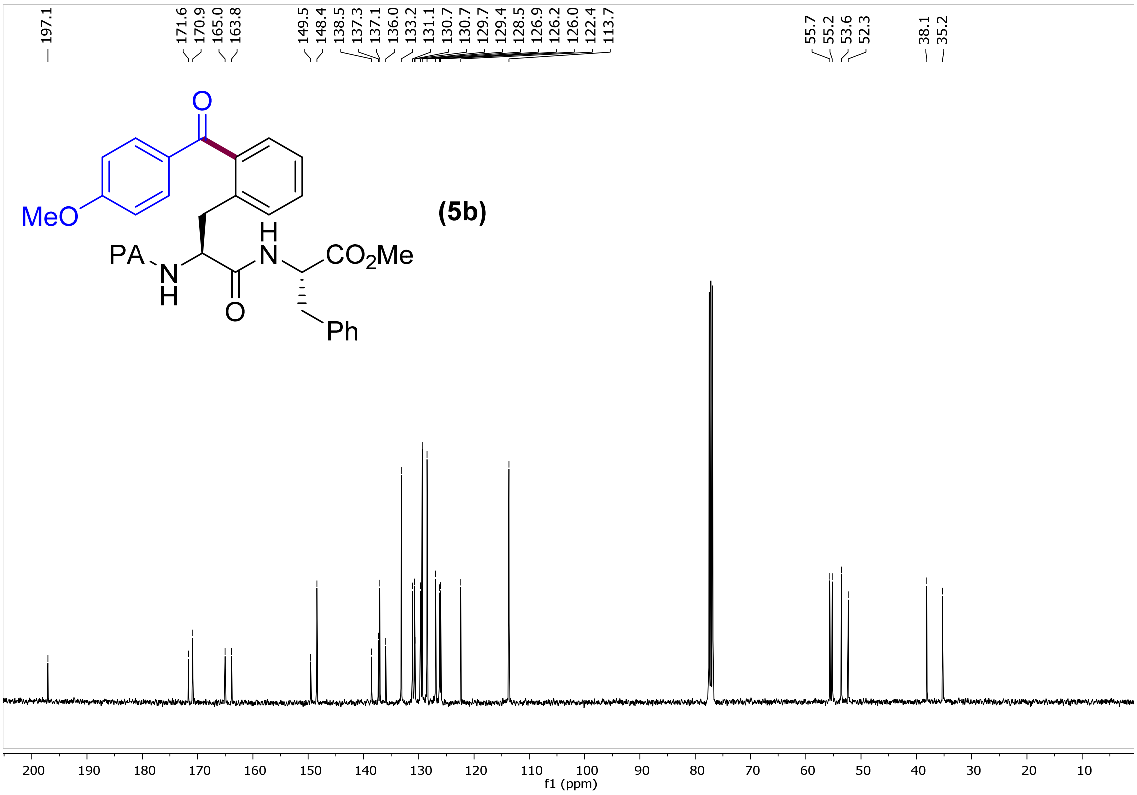

197.1
171.6
170.9
165.0
163.8
149.5
148.4
138.5
137.3
137.1
136.0
133.2
131.1
130.7
130.7
129.7
129.4
128.5
126.9
126.2
126.0
122.4
113.7
55.7
55.2
53.6
52.3
38.1
35.2
MeO
PA
N
H
O
CO2Me
Ph
(5b)
f1 (ppm)

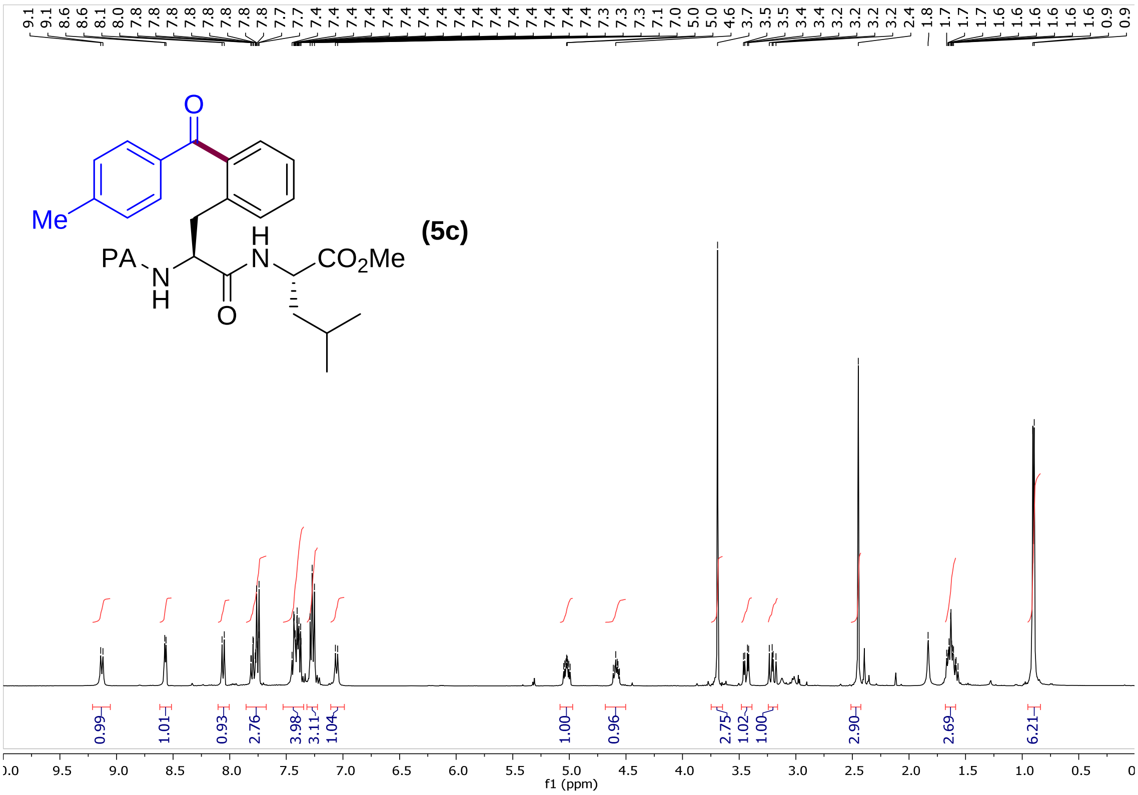
(5c)
Me
PA
CO2Me
f1 (ppm)

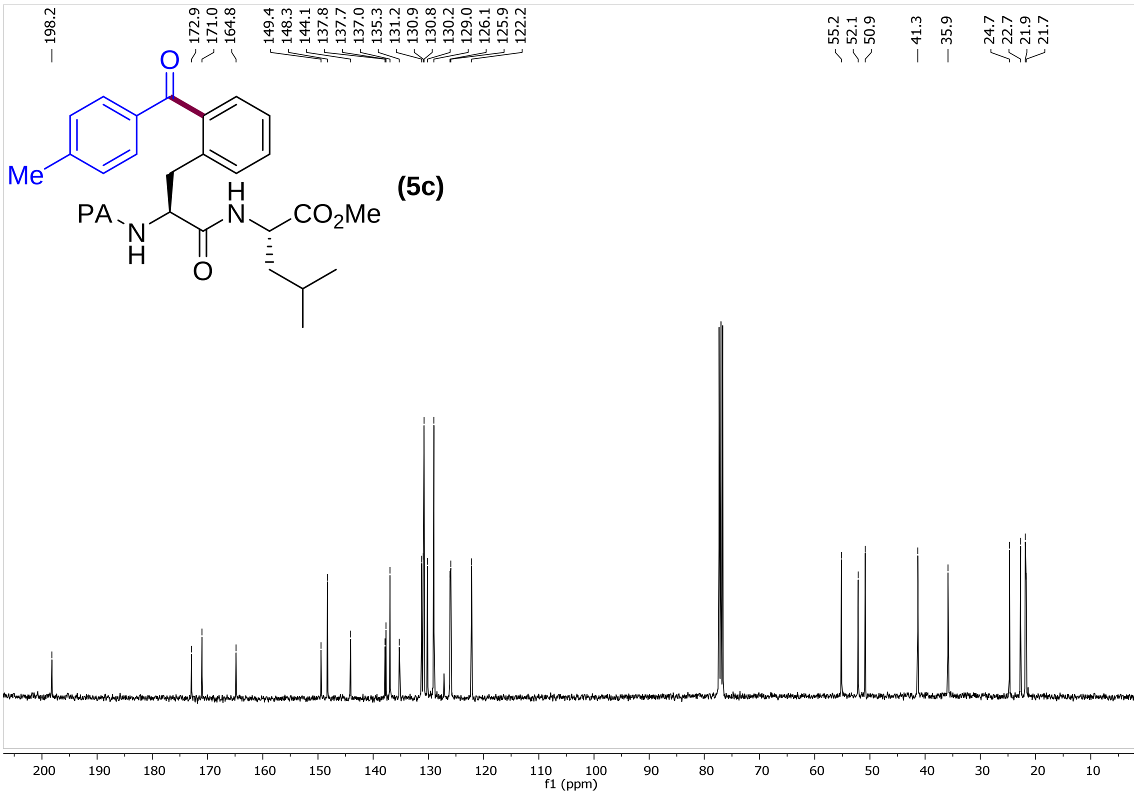
(5c)
Me
PA
CO2Me
f1 (ppm)

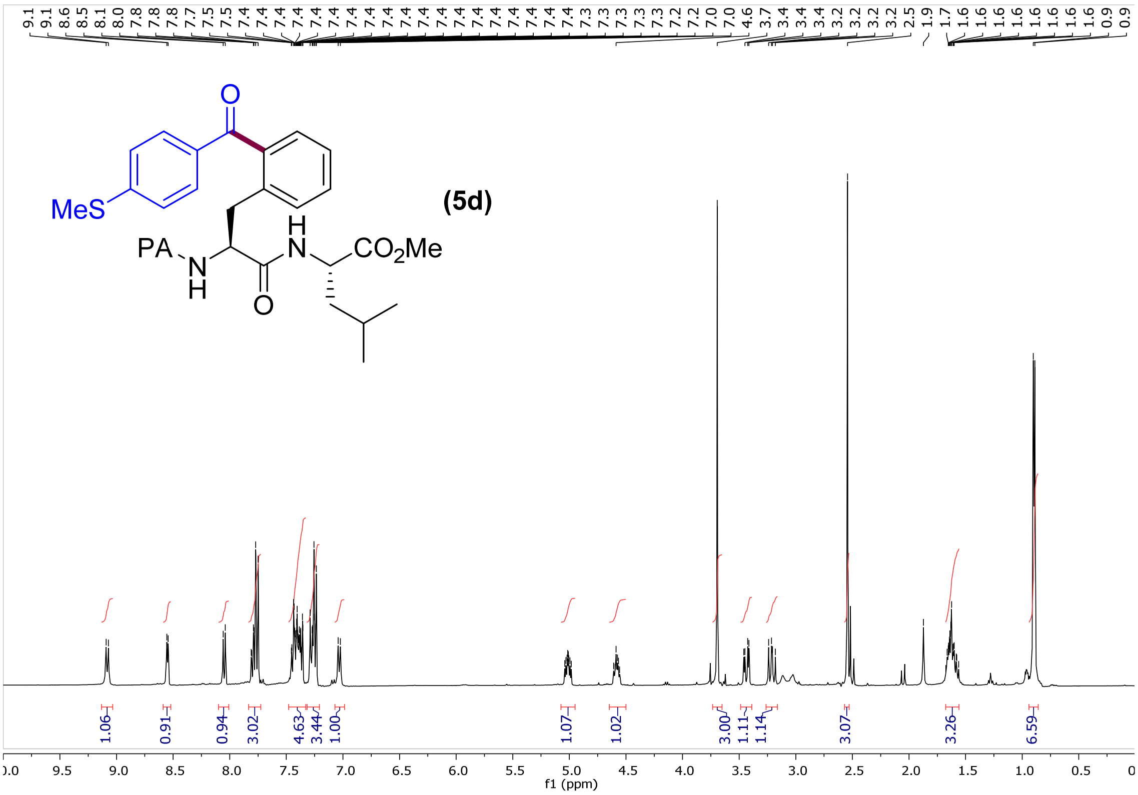

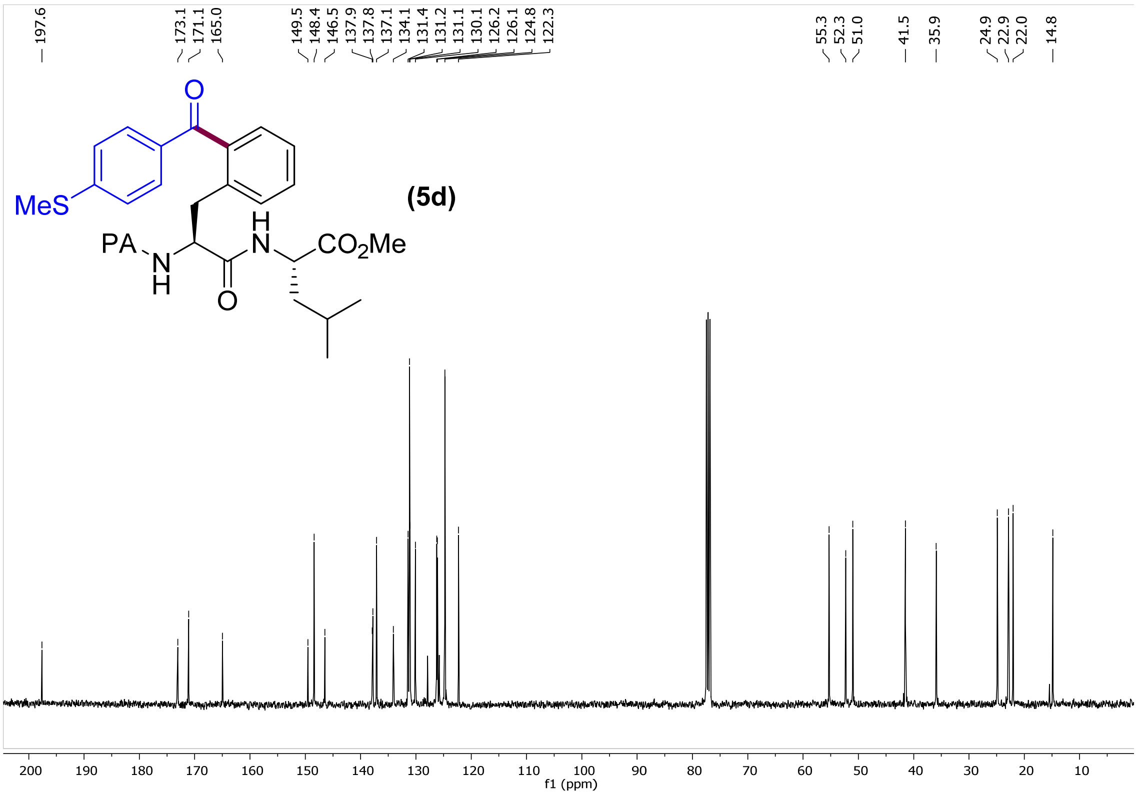

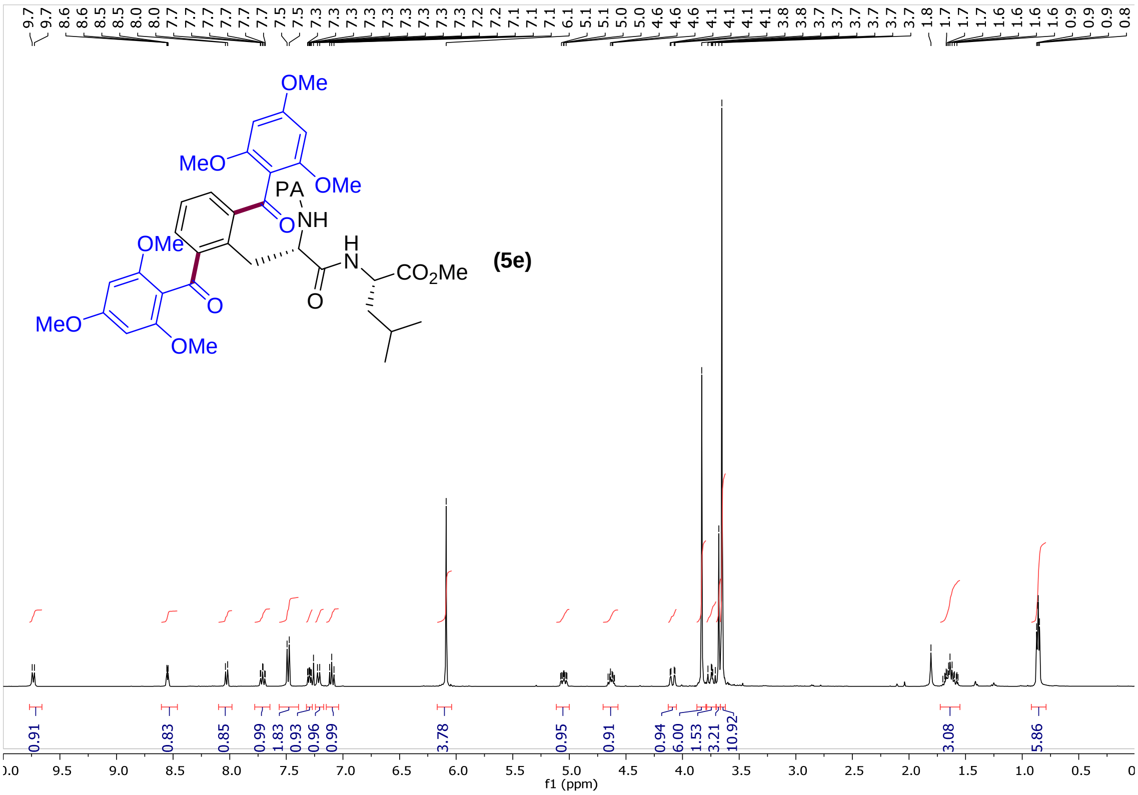

OMe
MeO
OMe
PA
NH
OMe
O
H
N
CO2Me
MeO
O
O
OMe
(5e)
0.91
0.83
0.85
0.99
1.83
0.93
0.96
0.99
3.78
0.95
0.91
0.94
6.00
1.53
3.21
10.92
3.08
5.86
f1 (ppm)

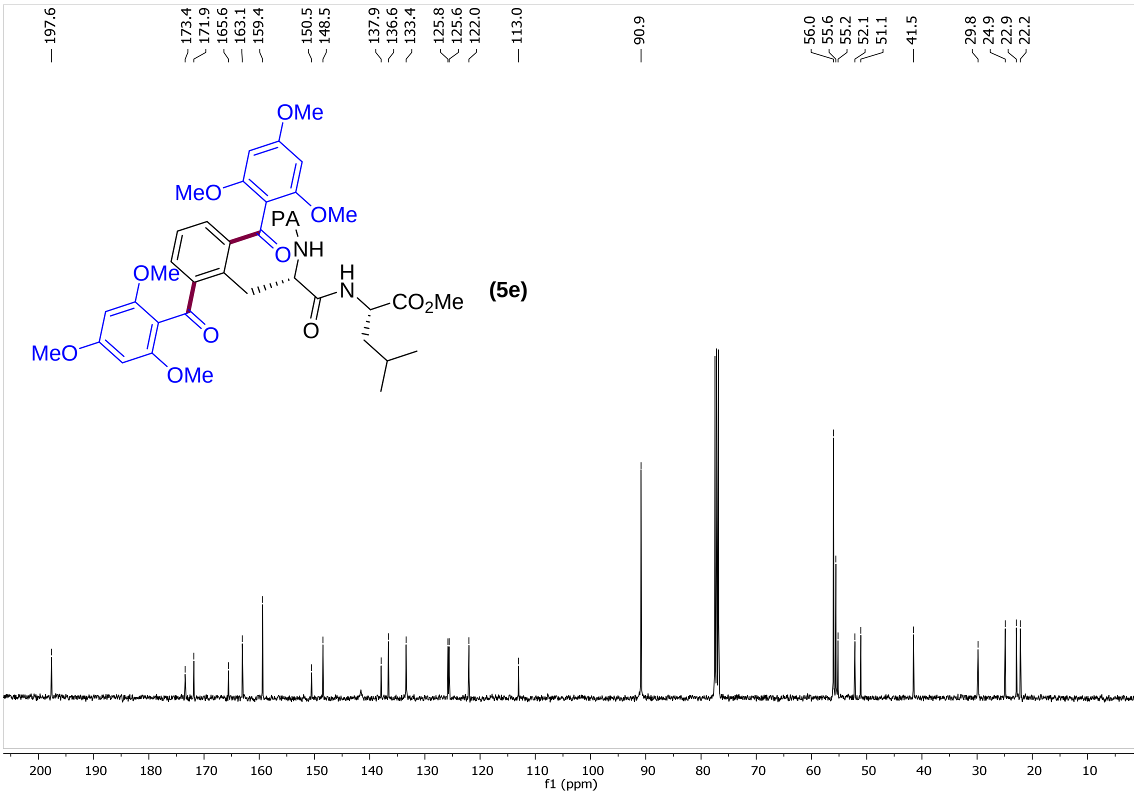

197.6
173.4
171.9
165.6
163.1
159.4
150.5
148.5
137.9
136.6
133.4
125.8
125.6
122.0
113.0
90.9
56.0
55.6
55.2
52.1
51.1
41.5
29.8
24.9
22.9
22.2
OMe
MeO
OMe
PA
NH
OMe
O
H
N
CO2Me
MeO
O
O
OMe
(5e)
f1 (ppm)

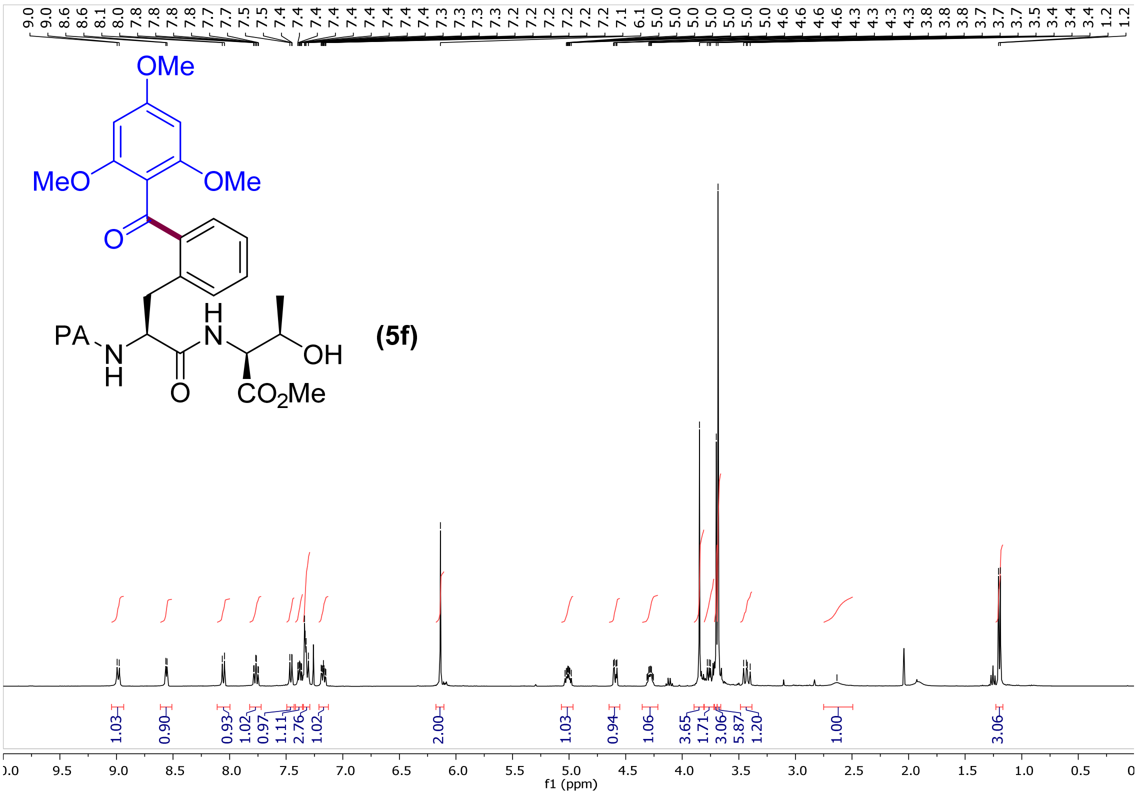

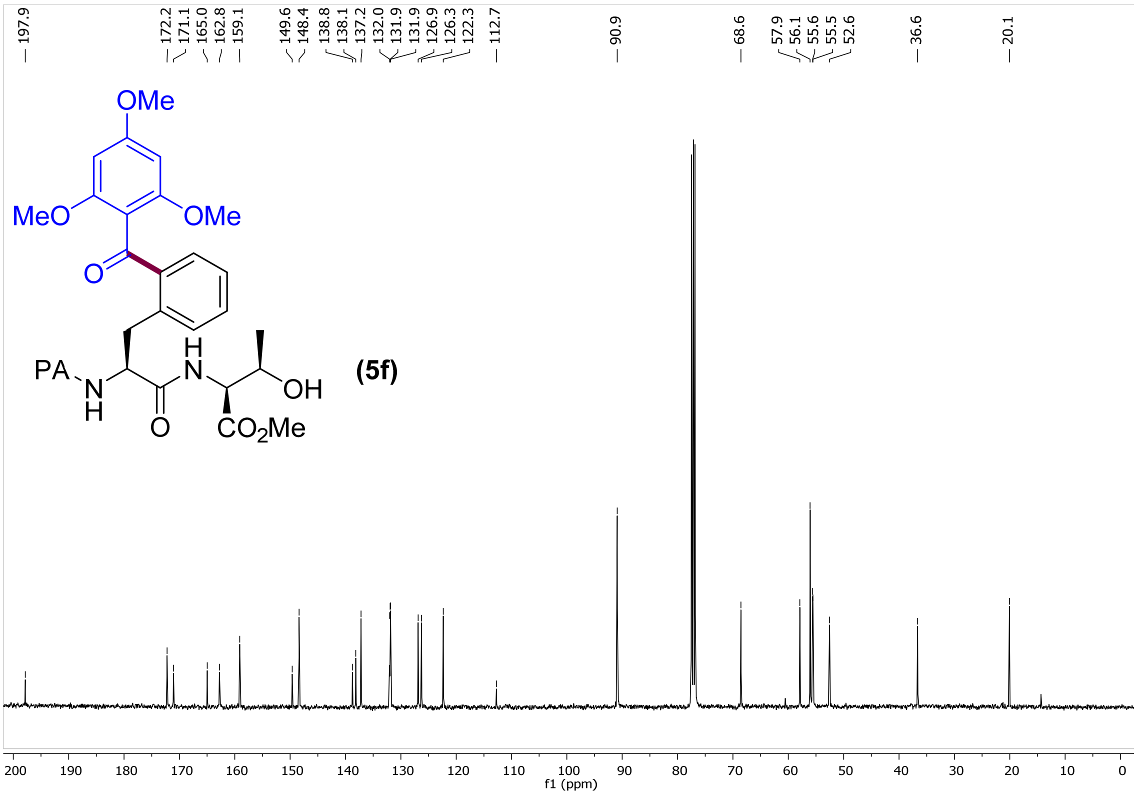

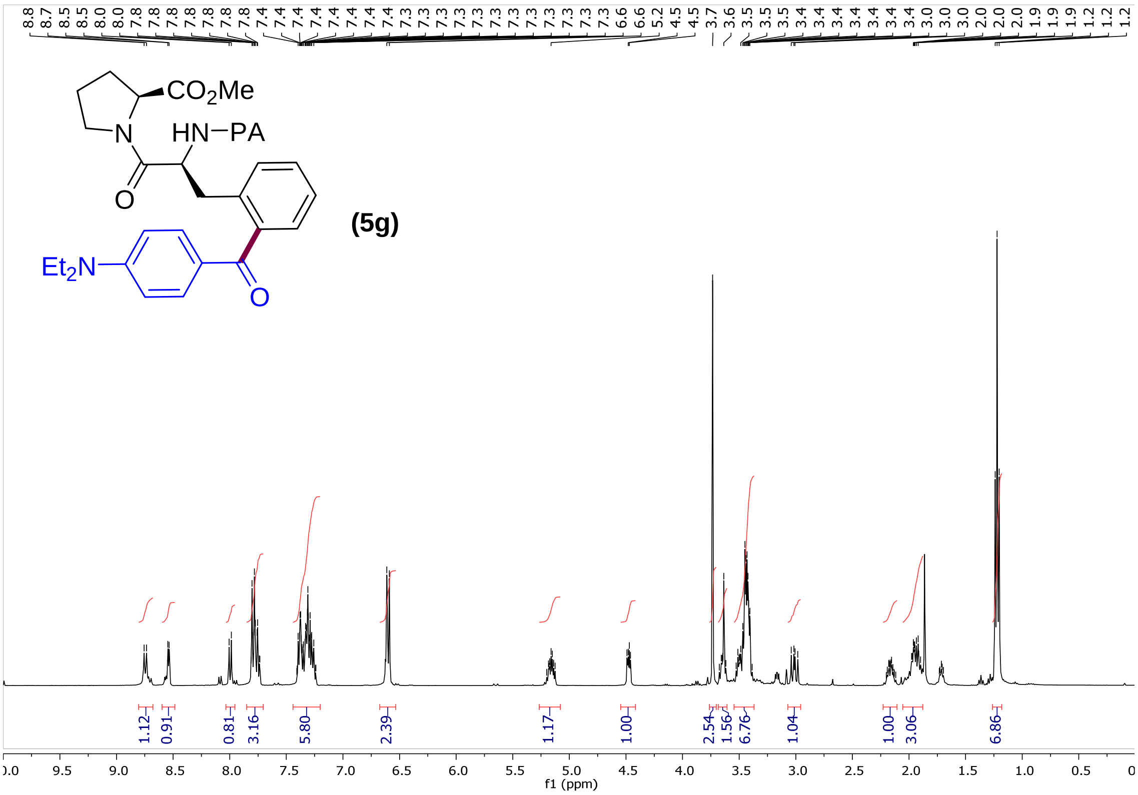
CO2Me
HN−PA
Et2N
(5g)
f1 (ppm)

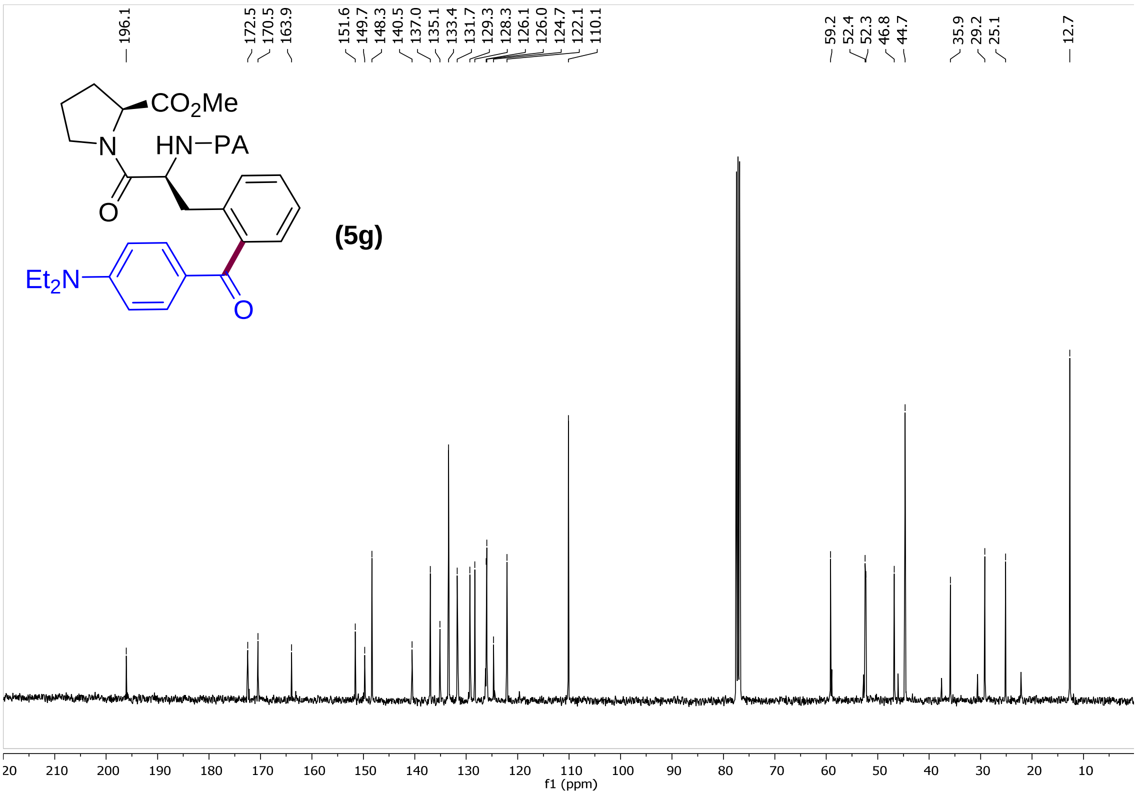
196.1
172.5
170.5
163.9
151.6
149.7
148.3
140.5
137.0
135.1
133.4
131.7
129.3
128.3
126.1
126.0
124.7
122.1
110.1
59.2
52.4
52.3
46.8
44.7
35.9
29.2
25.1
12.7
CO2Me
HN−PA
Et2N
(5g)
f1 (ppm)

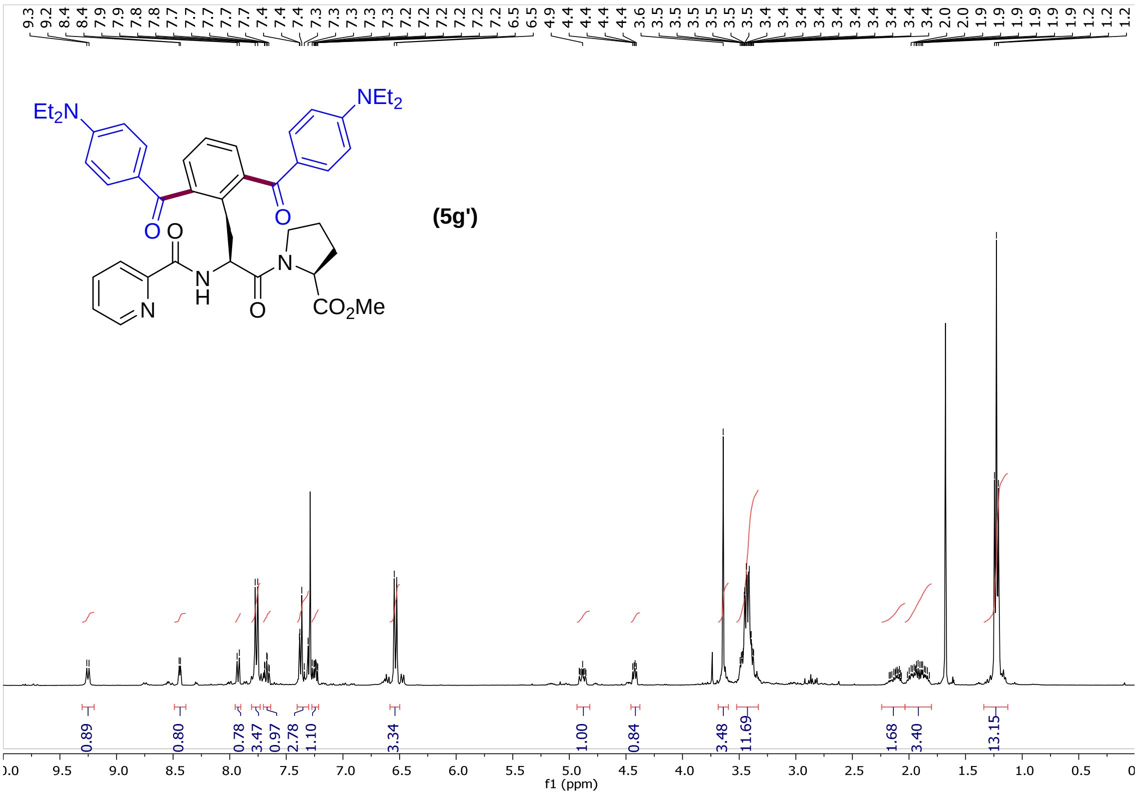

(5g')

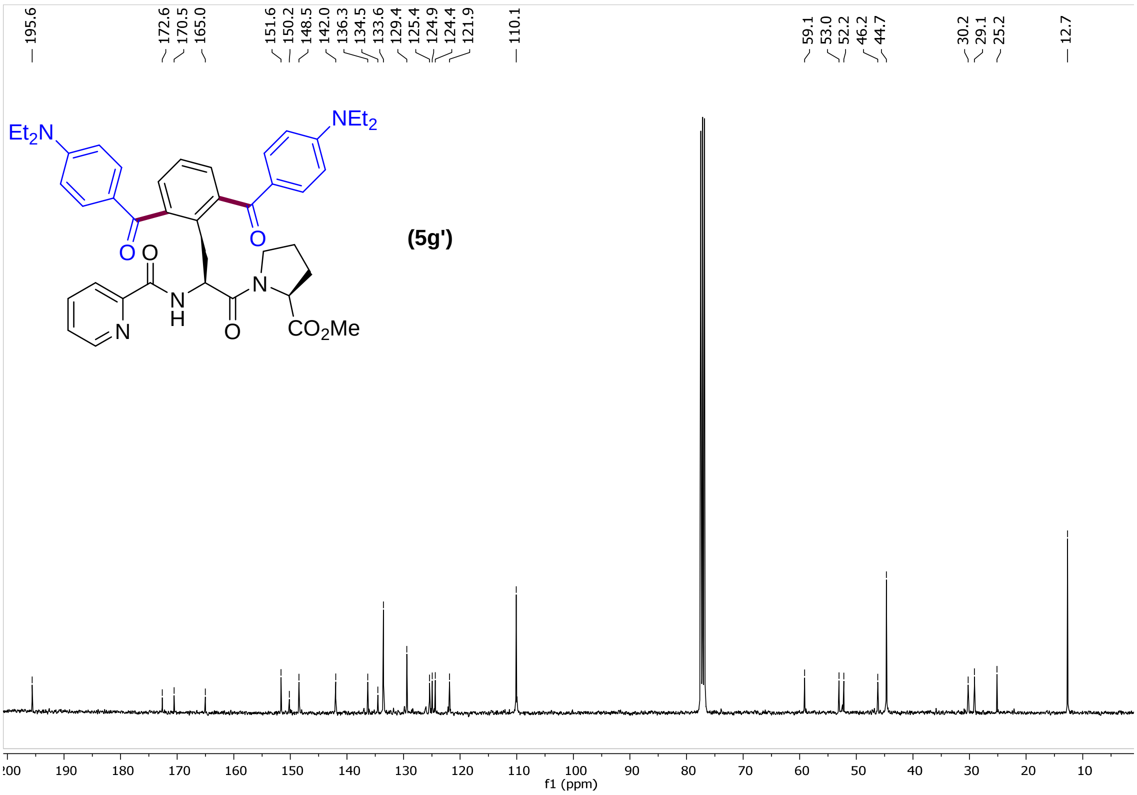

(5g')

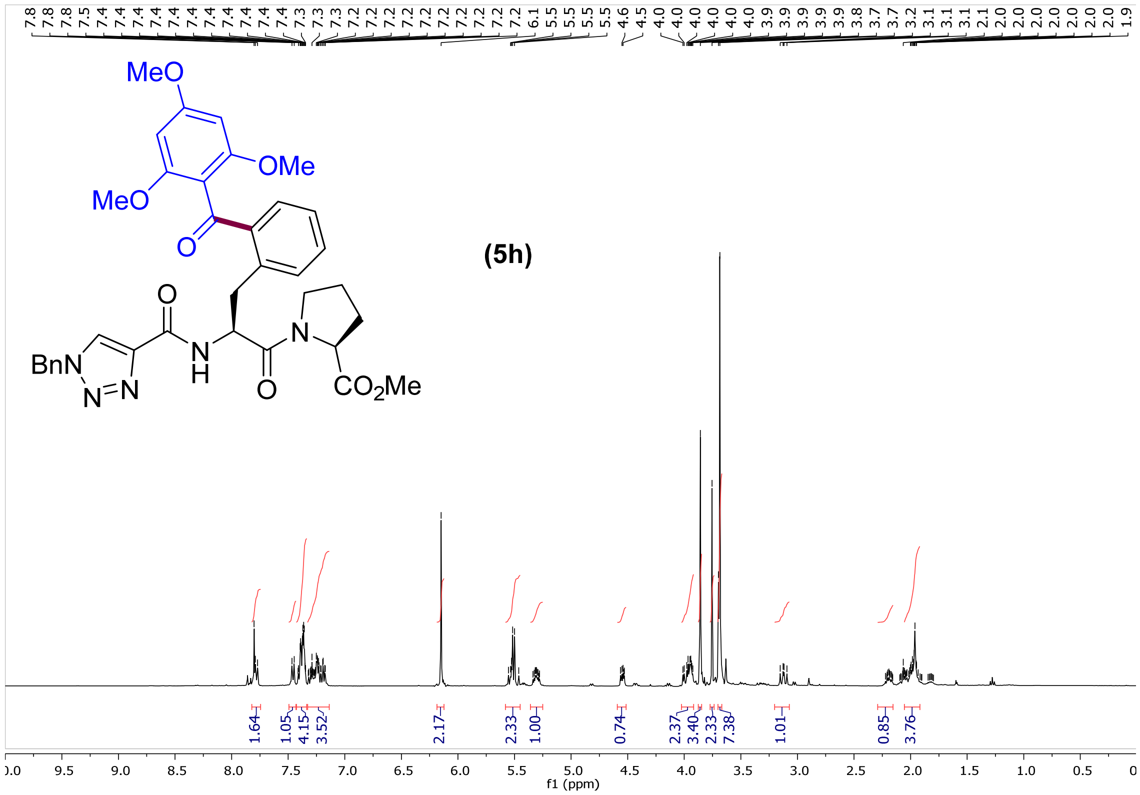

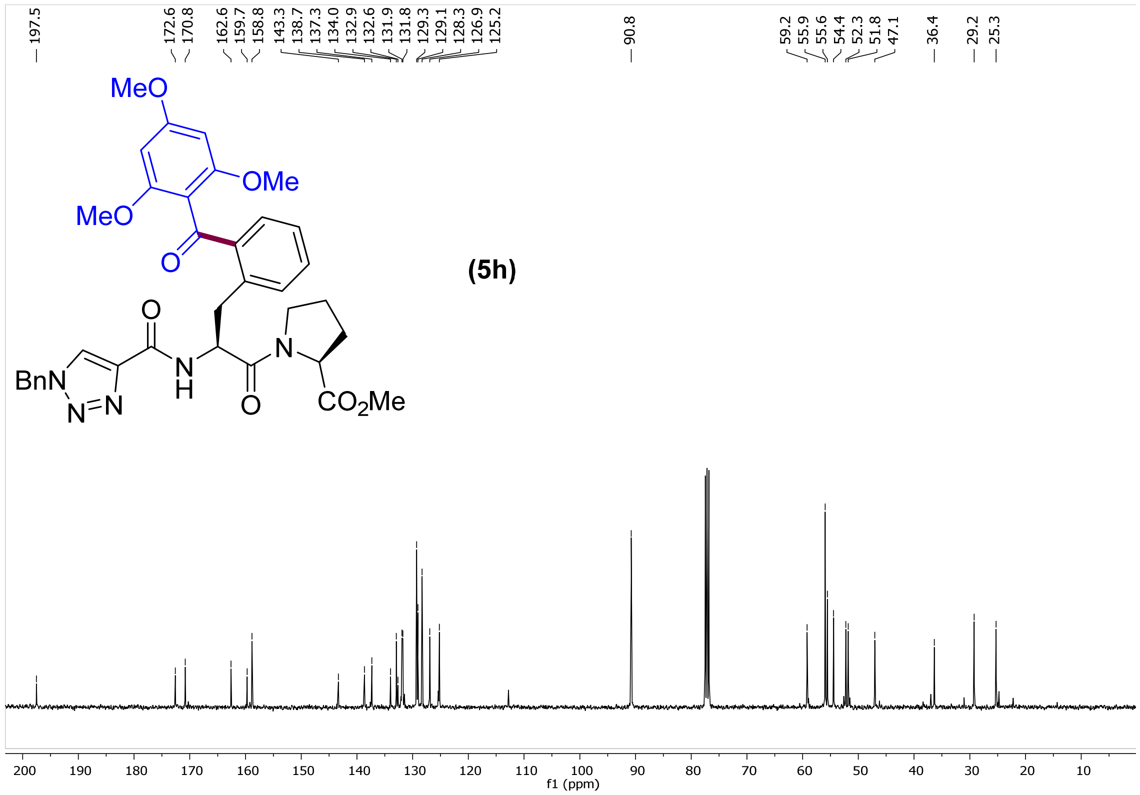

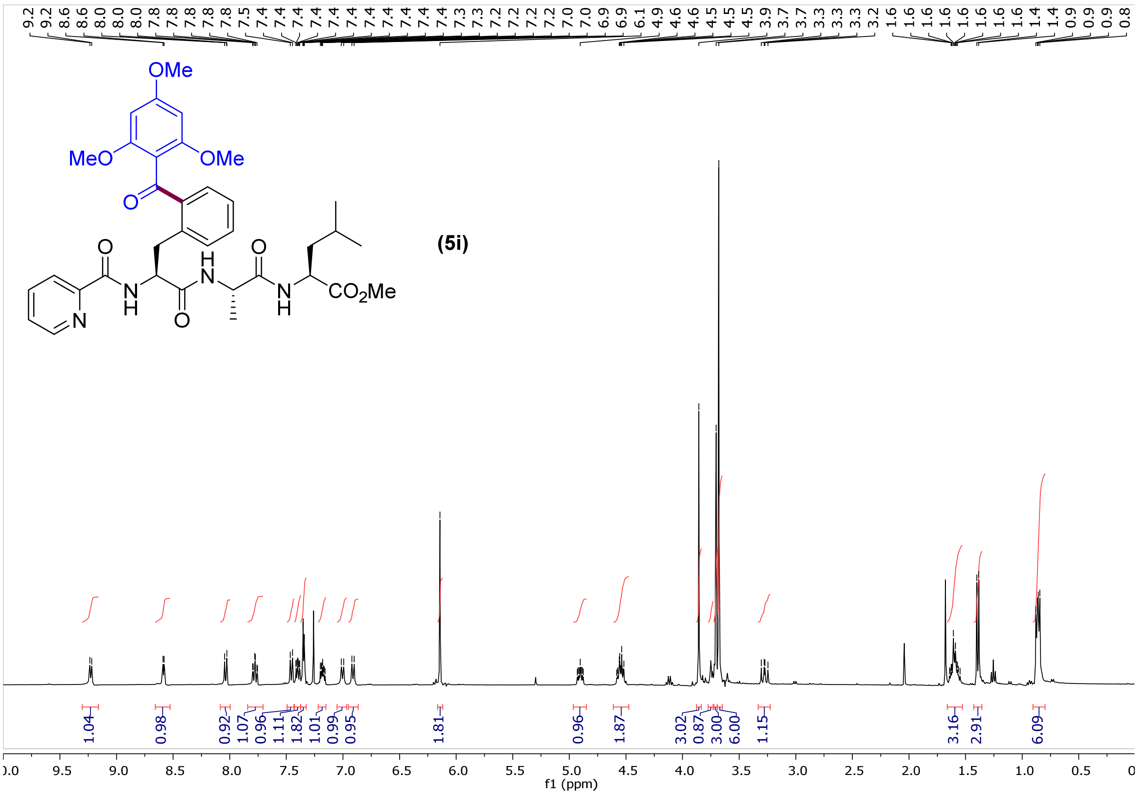
OMe
MeO
OMe
O
N
H
O
H
N
O
N
H
CO2Me
N
(5i)
1.04
0.98
0.92
1.07
0.96
1.11
1.82
1.01
0.99
0.95
1.81
0.96
1.87
3.02
0.87
3.00
6.00
1.15
3.16
2.91
6.09
f1 (ppm)

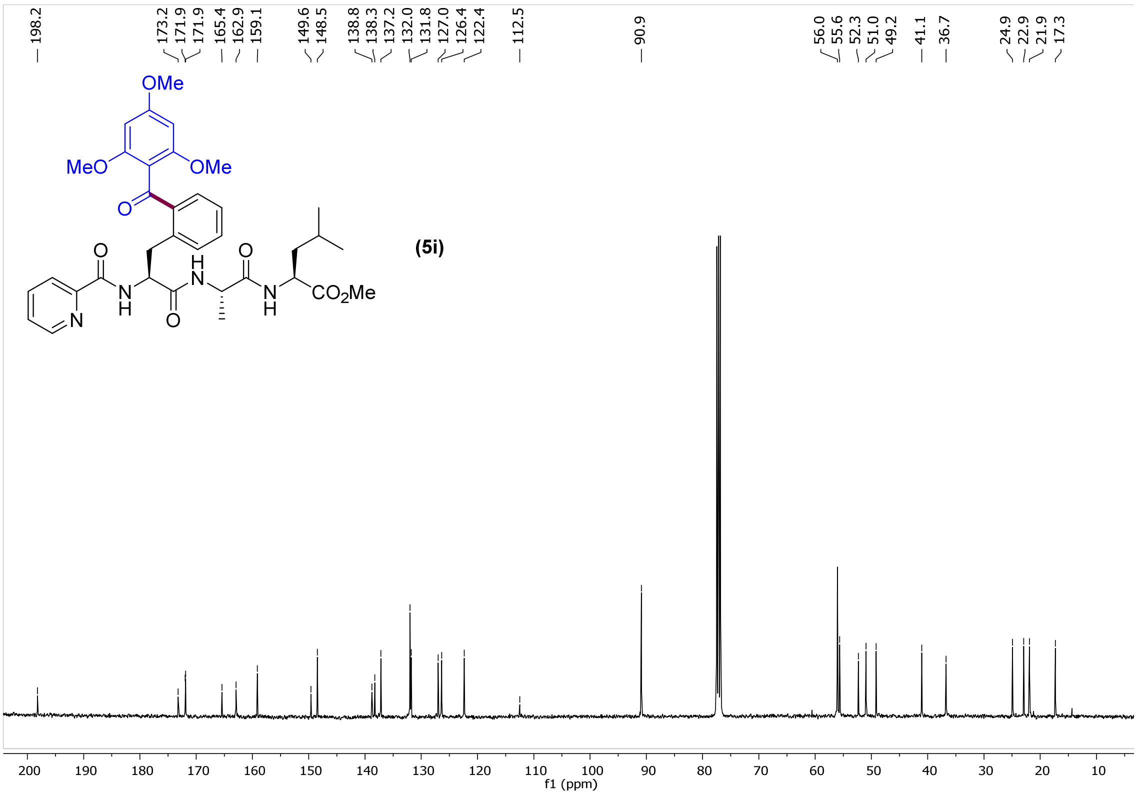
198.2
173.2
171.9
171.9
165.4
162.9
159.1
149.6
148.5
138.8
138.3
137.2
132.0
131.8
127.0
126.4
122.4
112.5
90.9
56.0
55.6
52.3
51.0
49.2
41.1
36.7
24.9
22.9
21.9
17.3
OMe
MeO
OMe
O
N
H
O
H
N
O
N
H
CO2Me
N
(5i)
f1 (ppm)

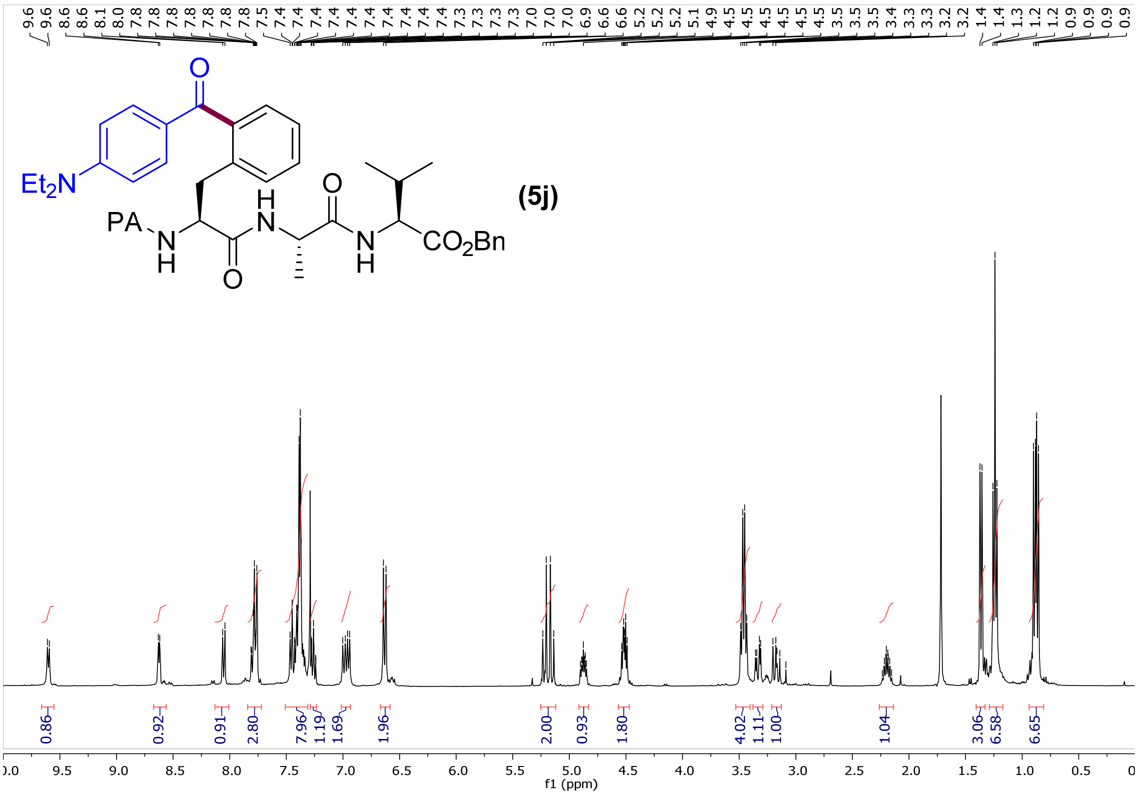
O
Et2N
PA
N
H
O
H
N
O
N
H
CO2Bn
(5j)
0.86
0.92
0.91
2.80
7.96
1.19
1.69
1.96
2.00
0.93
1.80
4.02
1.11
1.00
1.04
3.06
6.58
6.65
f1 (ppm)

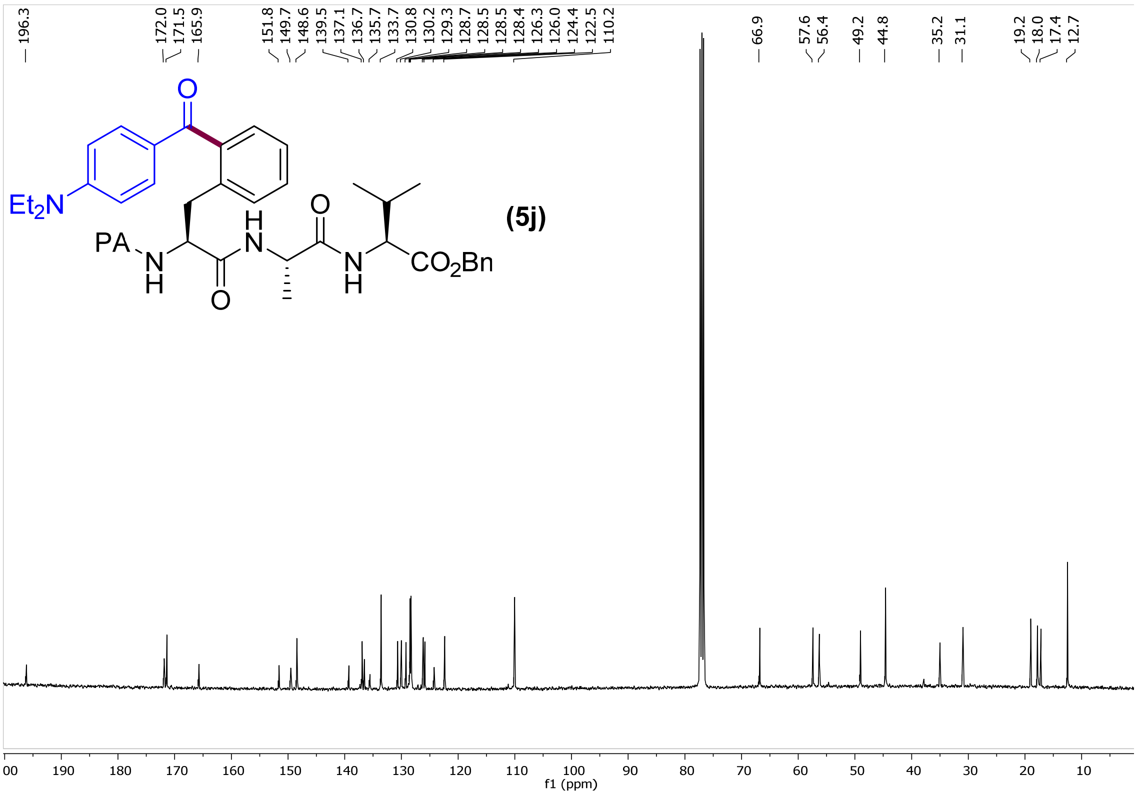
196.3
172.0
171.5
165.9
151.8
149.7
148.6
139.5
137.1
136.7
135.7
133.7
130.8
130.2
129.3
128.7
128.5
128.5
128.4
126.3
126.0
124.4
122.5
110.2
66.9
57.6
56.4
49.2
44.8
35.2
31.1
19.2
18.0
17.4
12.7
O
Et2N
PA
N
H
O
H
N
O
N
H
CO2Bn
(5j)
f1 (ppm)

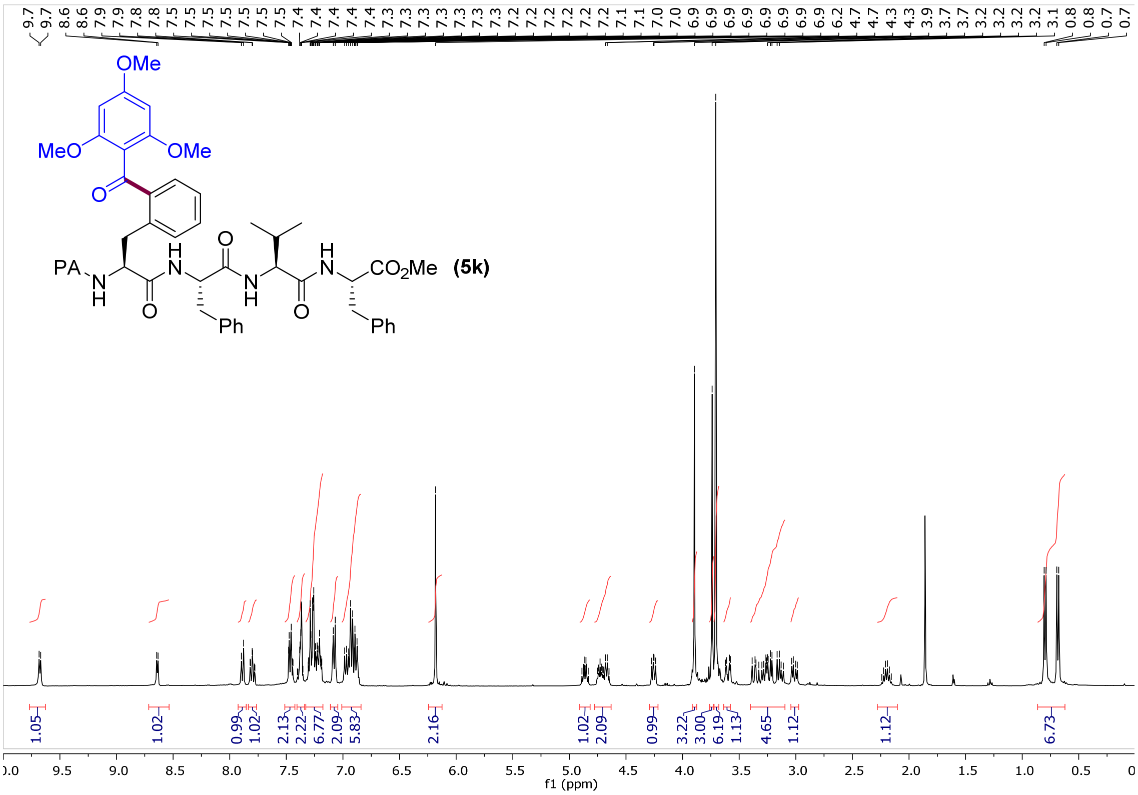

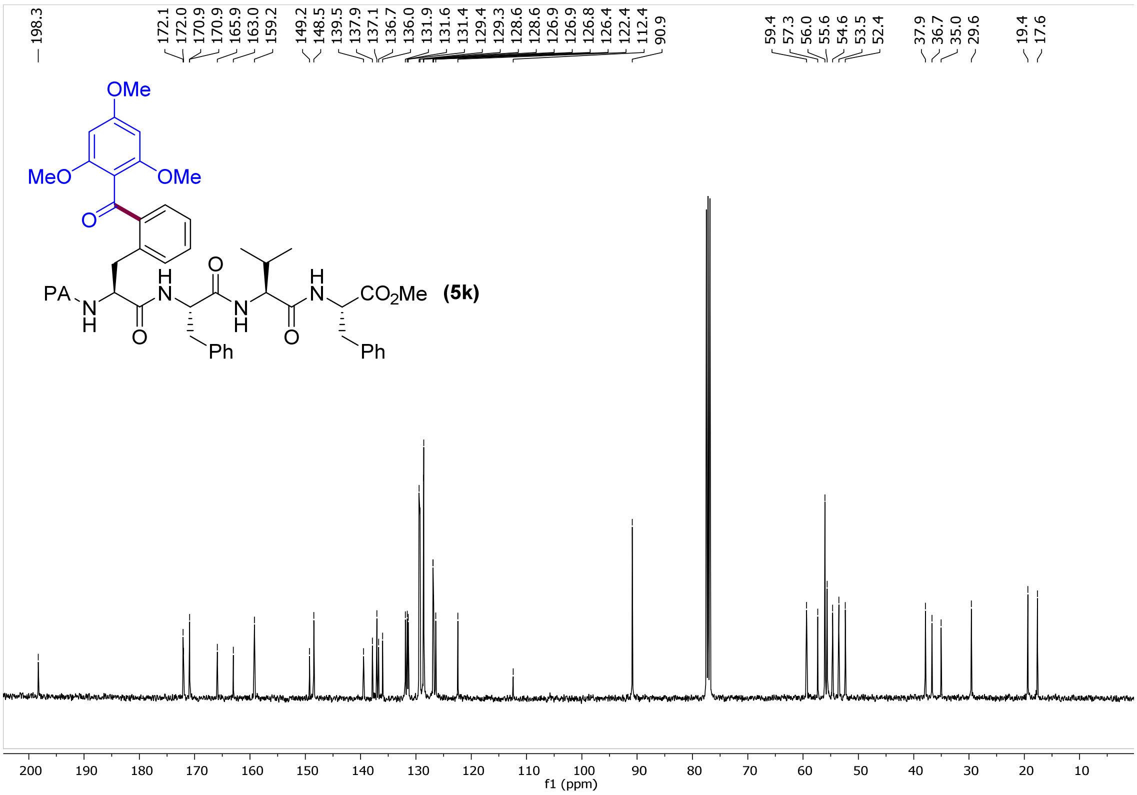

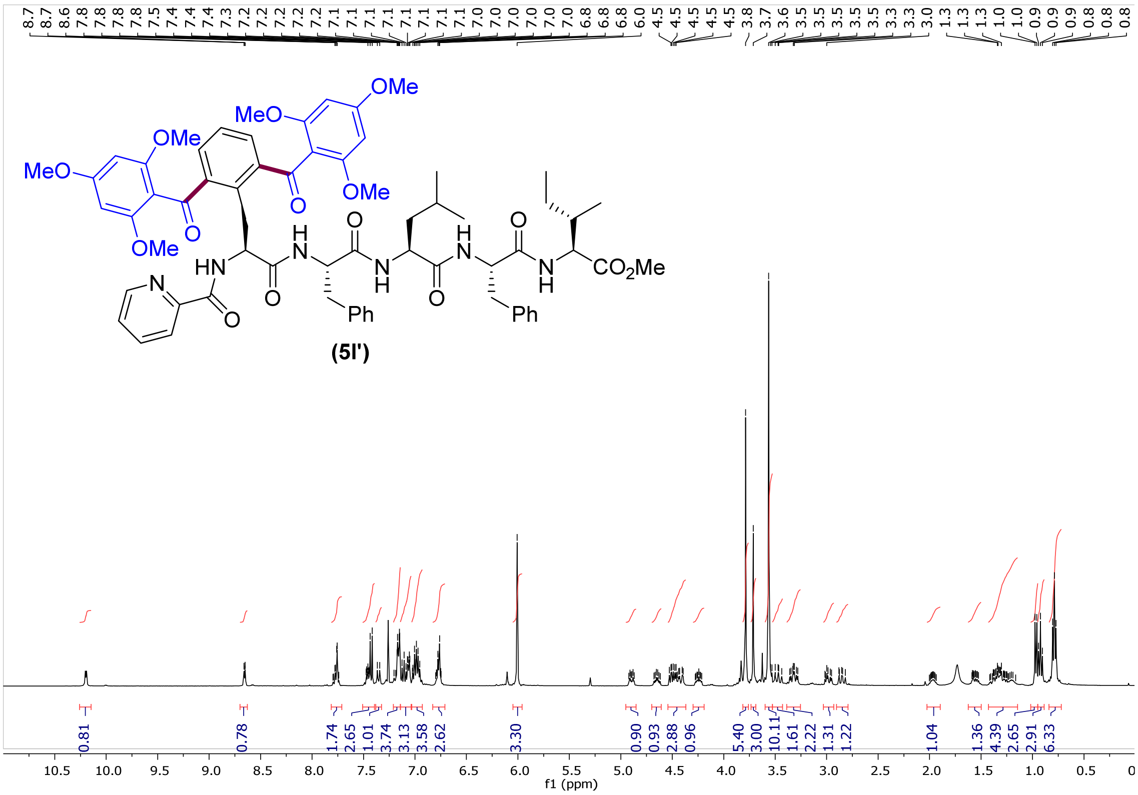
(5l')
f1 (ppm)

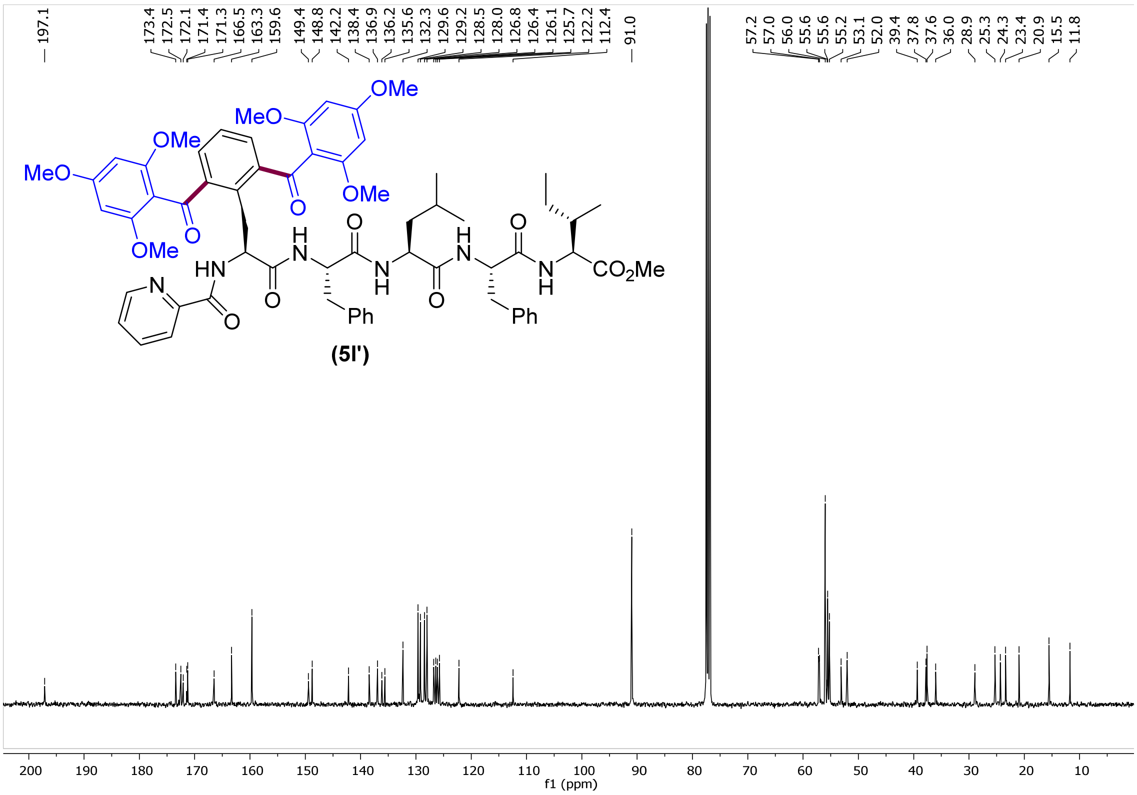
(5l')
f1 (ppm)

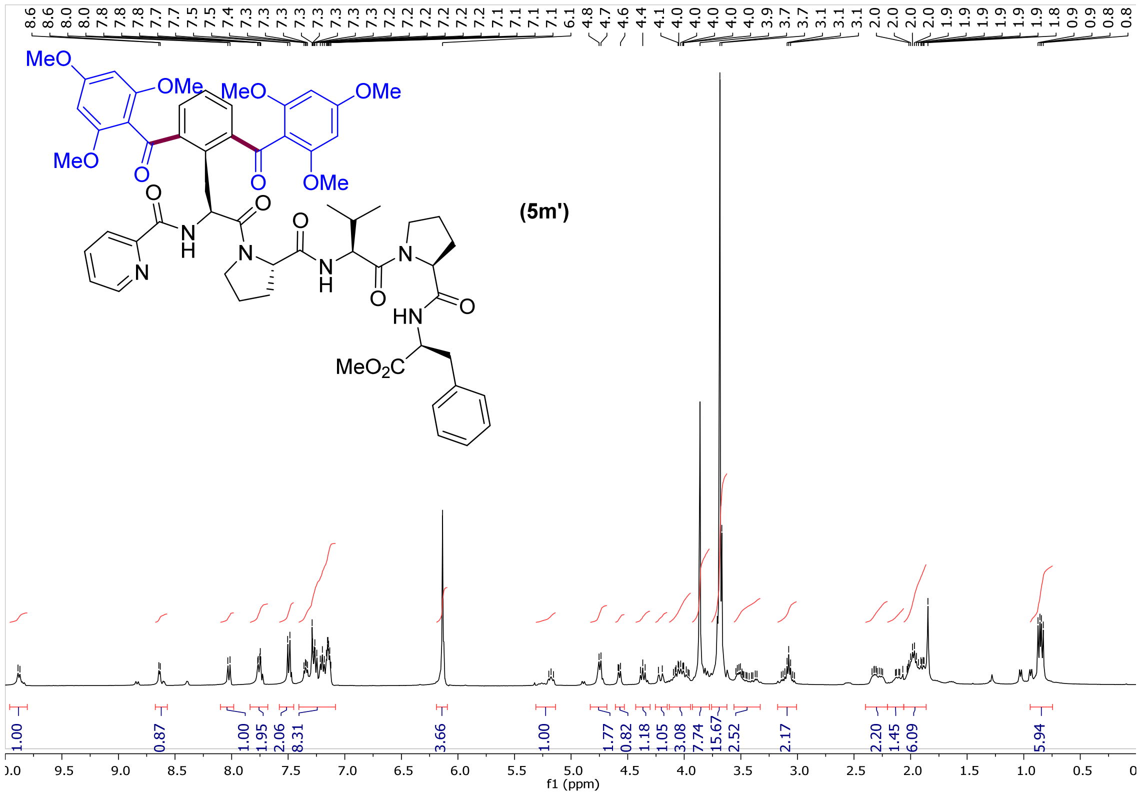

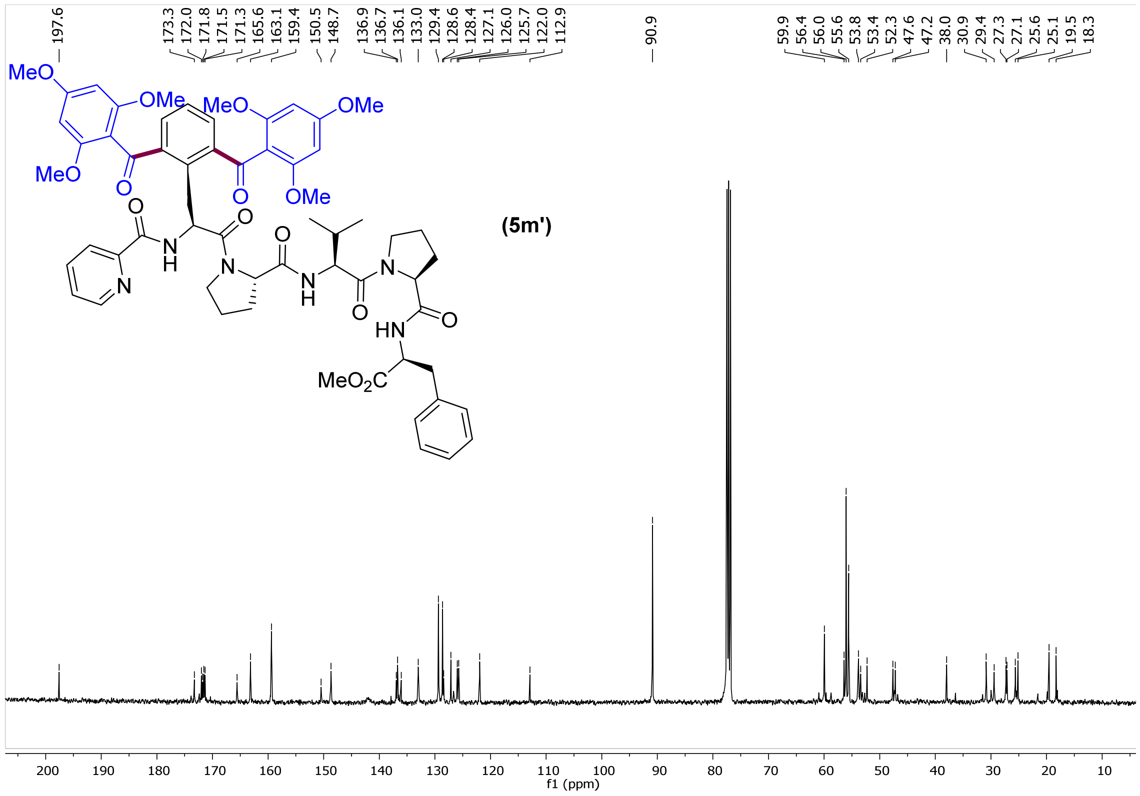

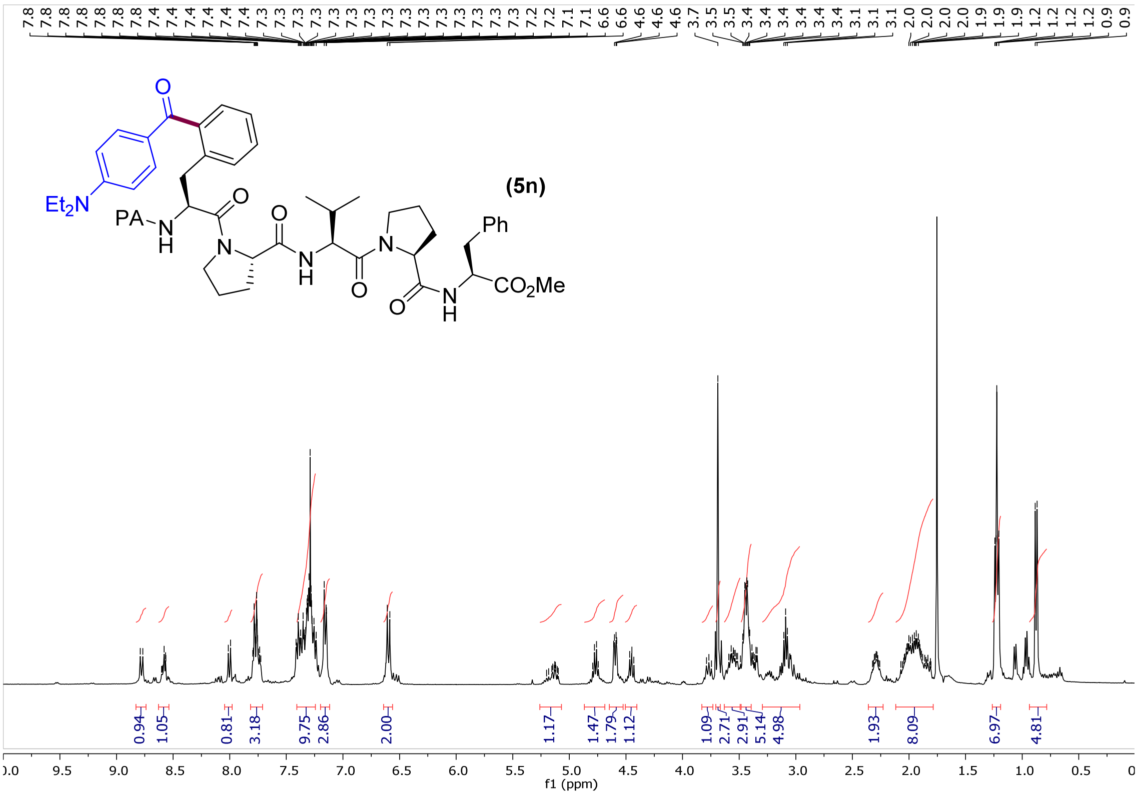
(5n)
Et2N
PA
Ph
CO2Me
f1 (ppm)

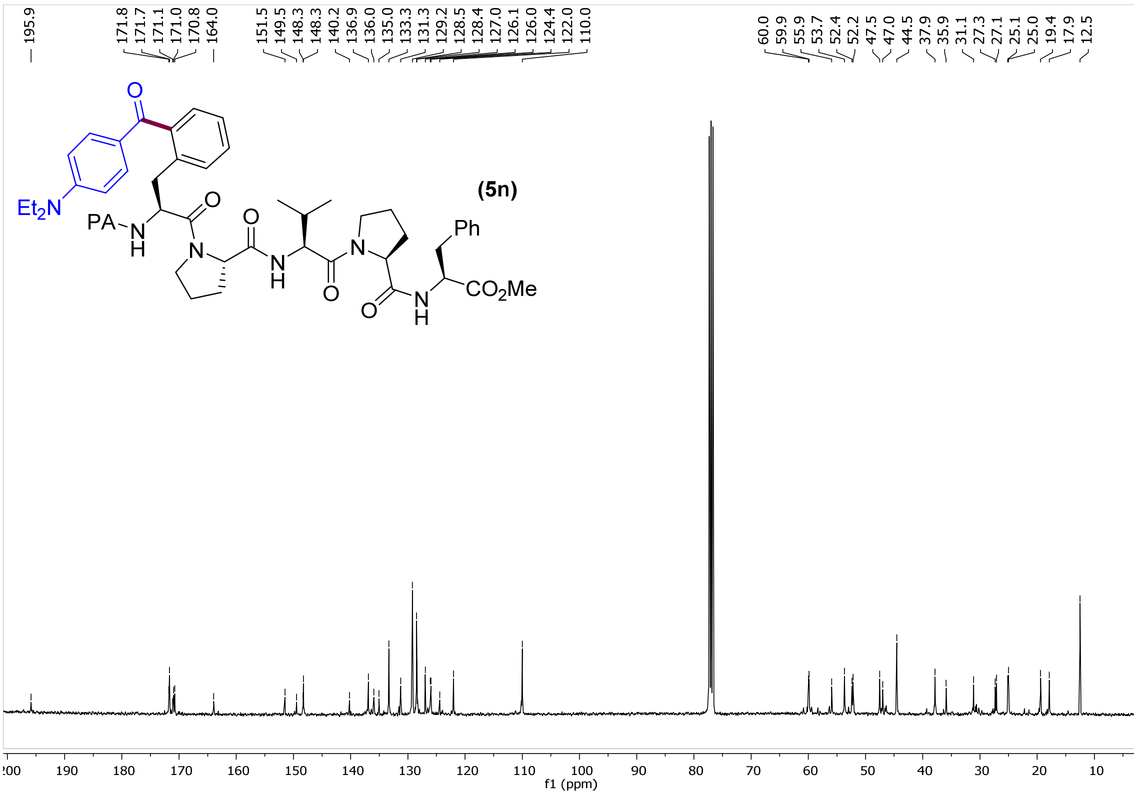
(5n)
Et2N
PA
Ph
CO2Me
f1 (ppm)

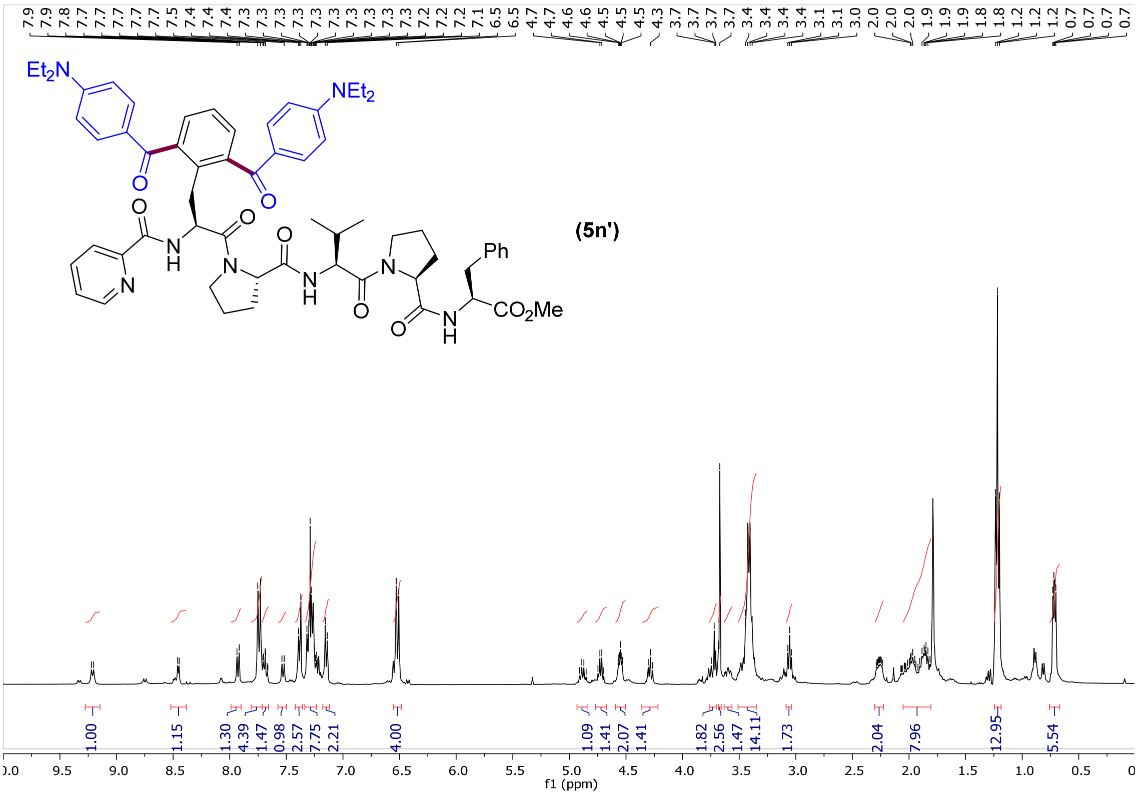

(5n')

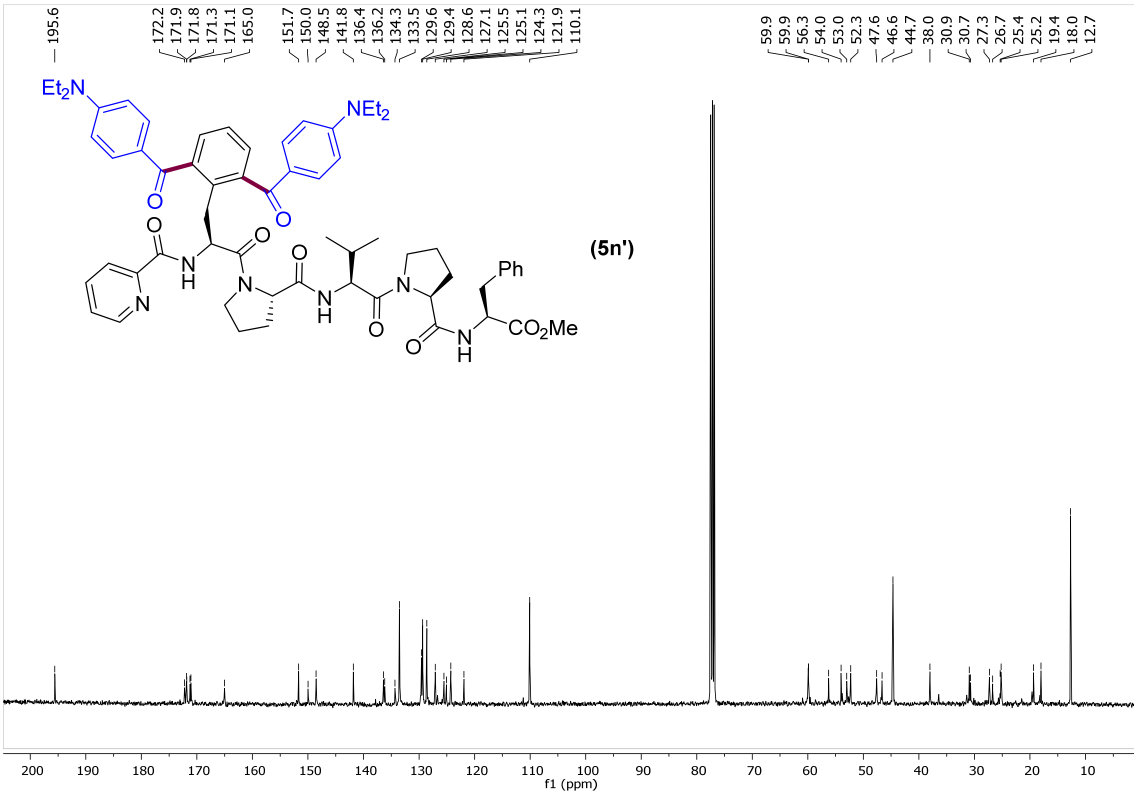

(5n')

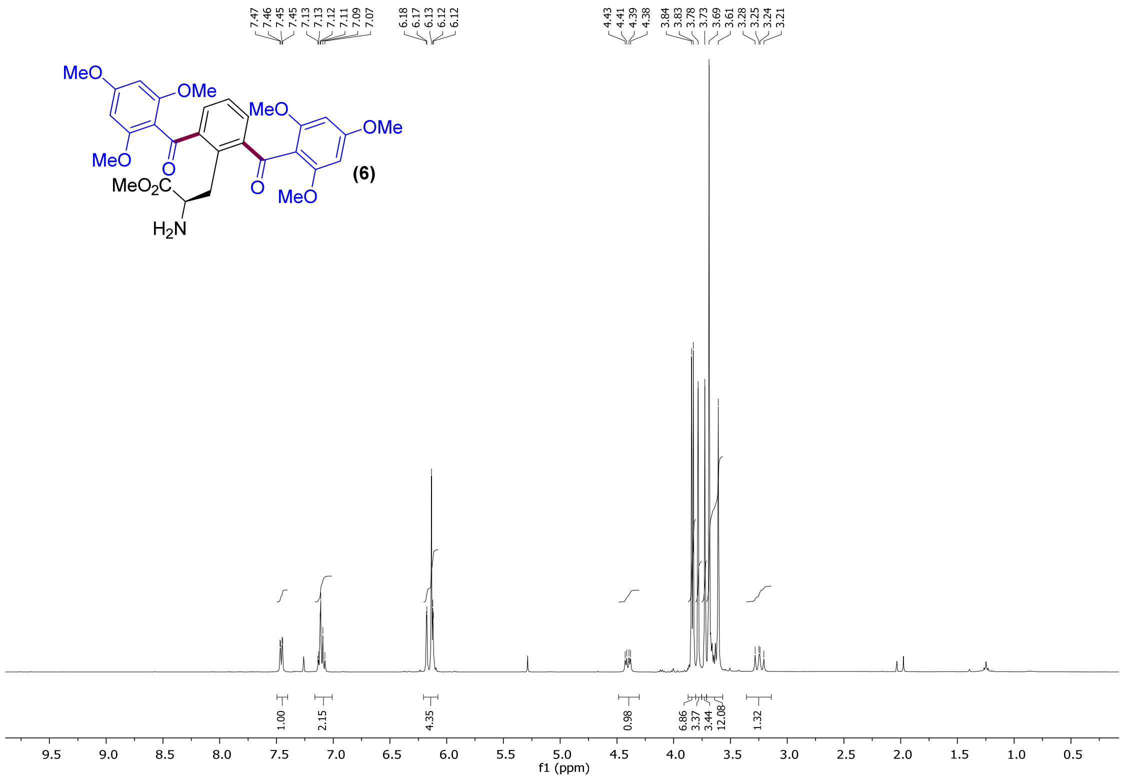
7.47
7.46
7.45
7.45
7.13
7.13
7.12
7.11
7.09
7.07
6.18
6.17
6.13
6.12
6.12
6.12
4.43
4.41
4.39
4.38
3.84
3.83
3.78
3.73
3.69
3.61
3.28
3.25
3.24
3.21
MeO
OMe
MeO
OMe
MeO
MeO
MeO₂C
H₂N
O
O
(6)
1.00
2.15
4.35
0.98
6.86
3.37
3.44
12.08
1.32
f1 (ppm)

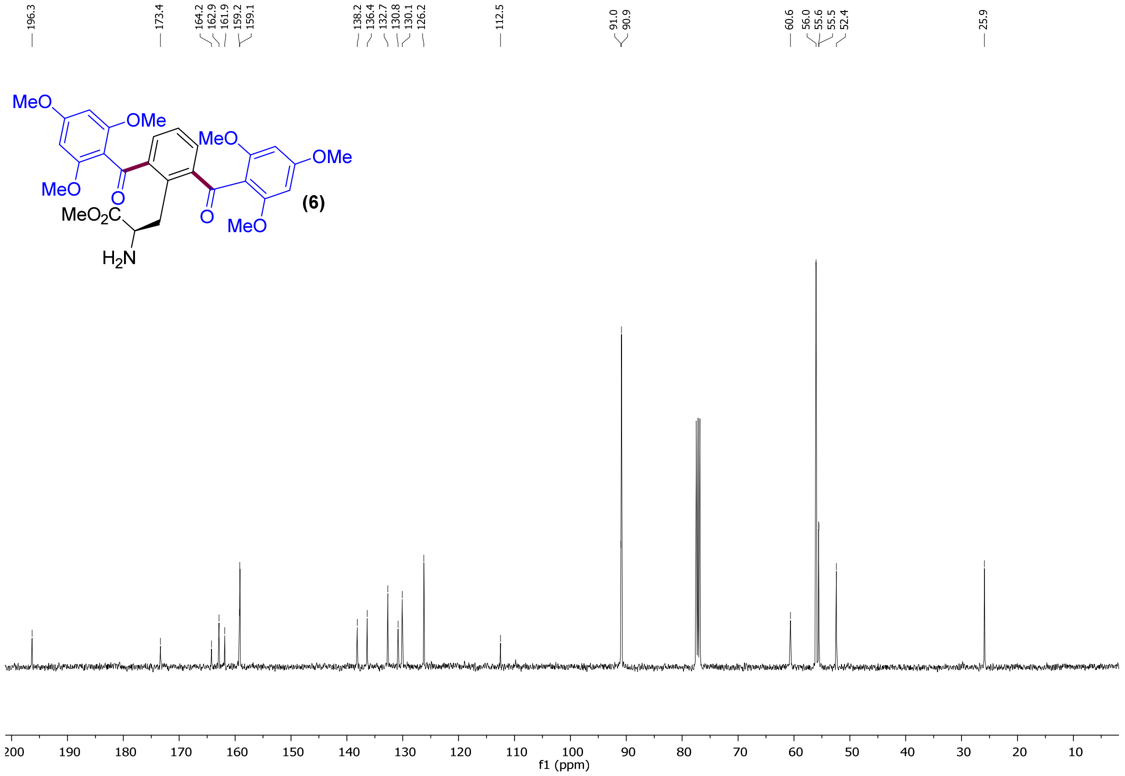
196.3
173.4
164.2
162.9
161.9
159.2
159.1
138.2
136.4
132.7
130.8
130.1
126.2
112.5
91.0
90.9
60.6
56.0
55.6
55.5
52.4
25.9
MeO
OMe
MeO
OMe
MeO
MeO
MeO₂C
H₂N
O
O
(6)
f1 (ppm)

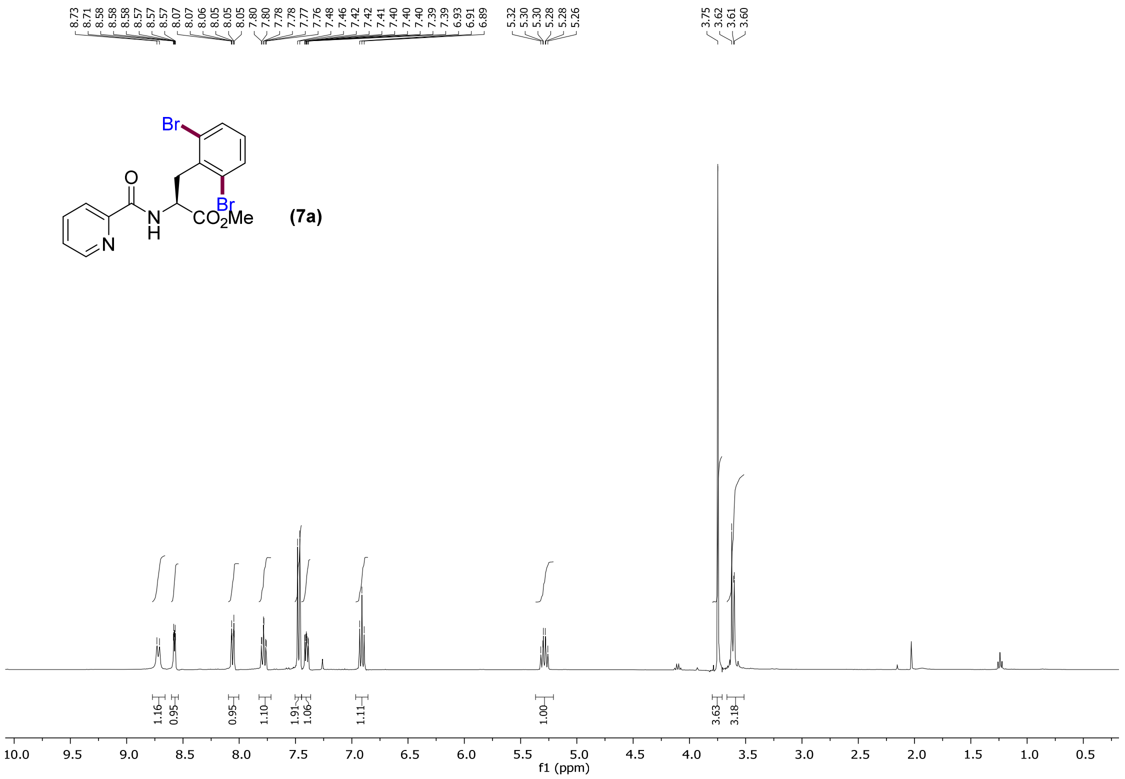
8.73
8.71
8.58
8.58
8.58
8.57
8.57
8.57
8.07
8.07
8.06
8.05
8.05
8.05
7.80
7.80
7.78
7.78
7.77
7.76
7.48
7.46
7.42
7.42
7.41
7.40
7.40
7.40
7.39
7.39
6.93
6.91
6.89
5.32
5.30
5.30
5.28
5.28
5.26
3.75
3.62
3.61
3.60
Br
Br
O
N
H
CO2Me
N
(7a)
1.16
0.95
0.95
1.10
1.91
1.06
1.11
1.00
3.63
3.18
f1 (ppm)

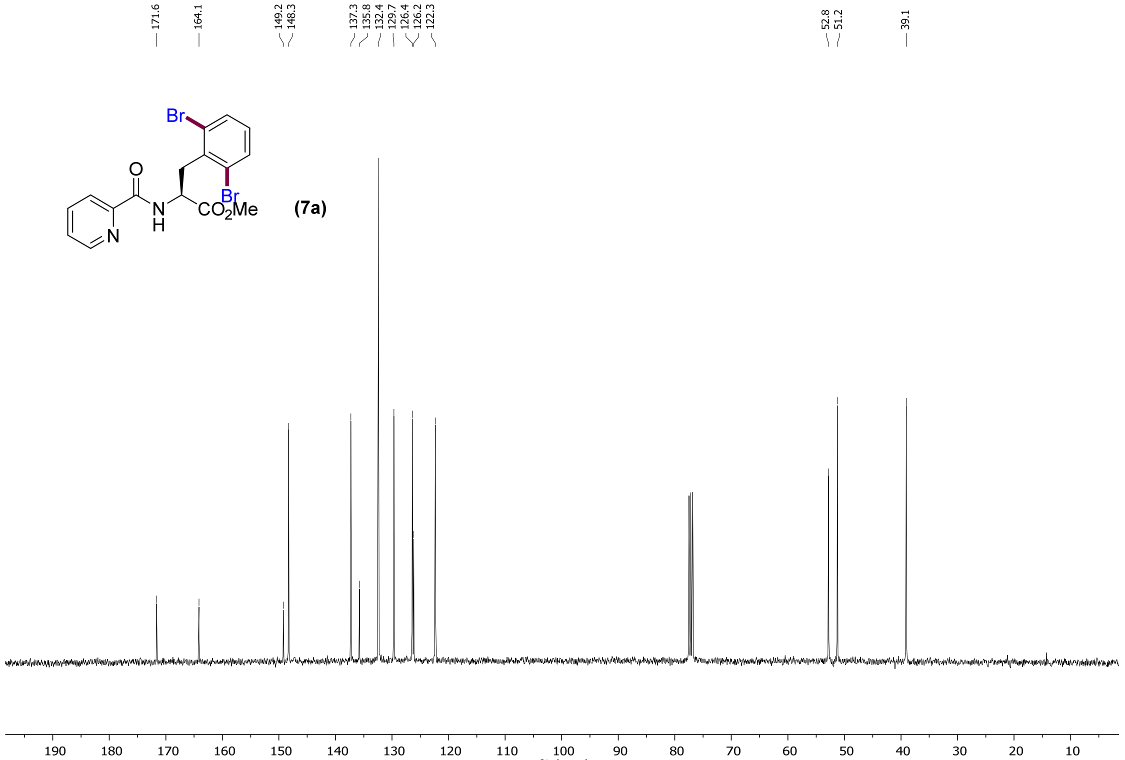
171.6
164.1
149.2
148.3
137.3
135.8
132.4
129.7
126.4
126.2
122.3
52.8
51.2
39.1
Br
Br
O
N
H
CO2Me
N
(7a)
f1 (ppm)

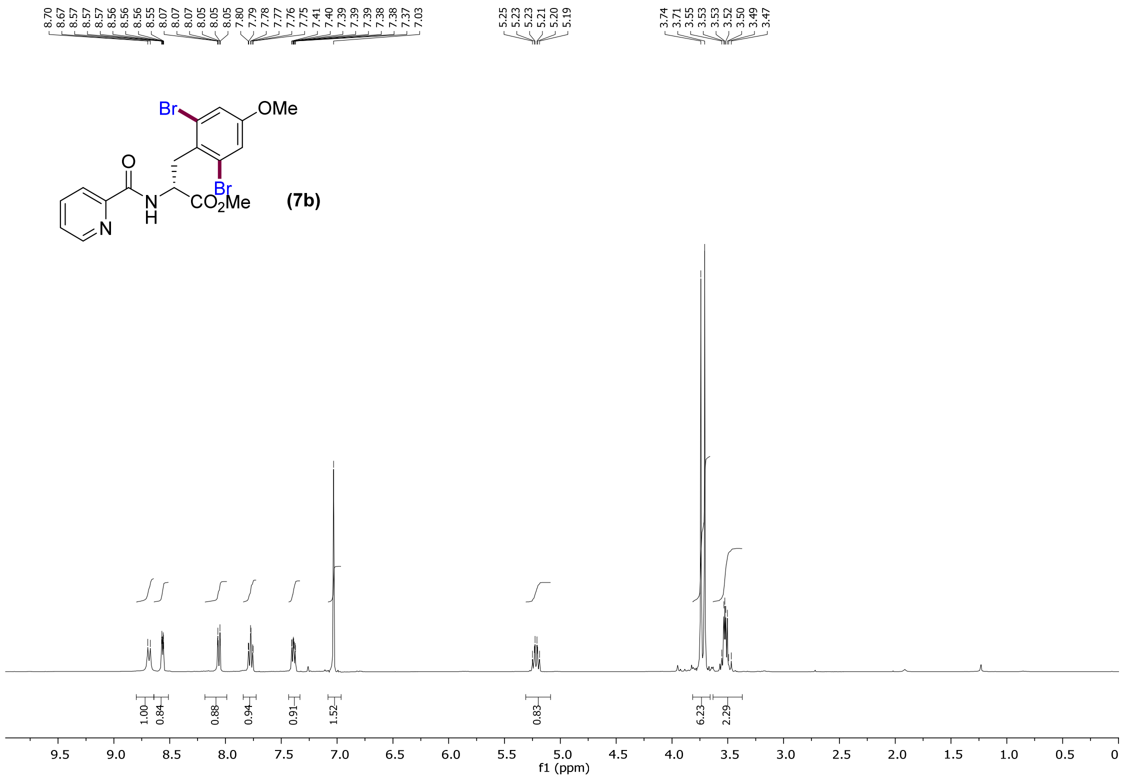

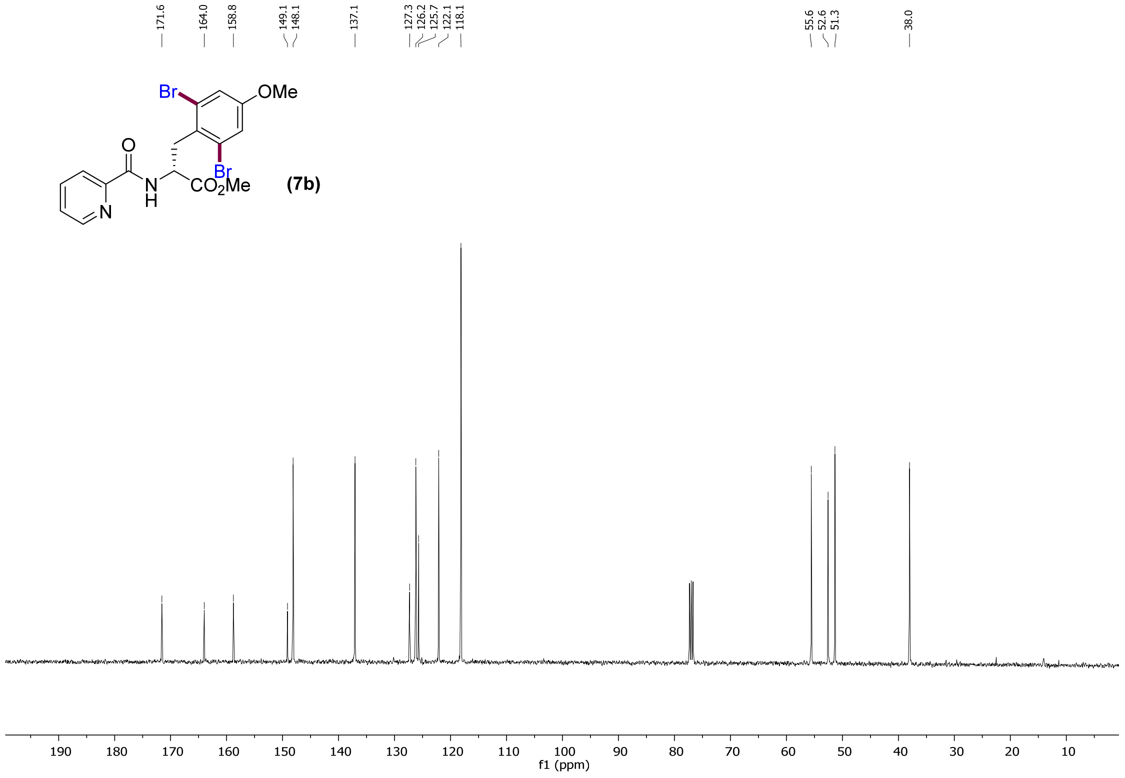

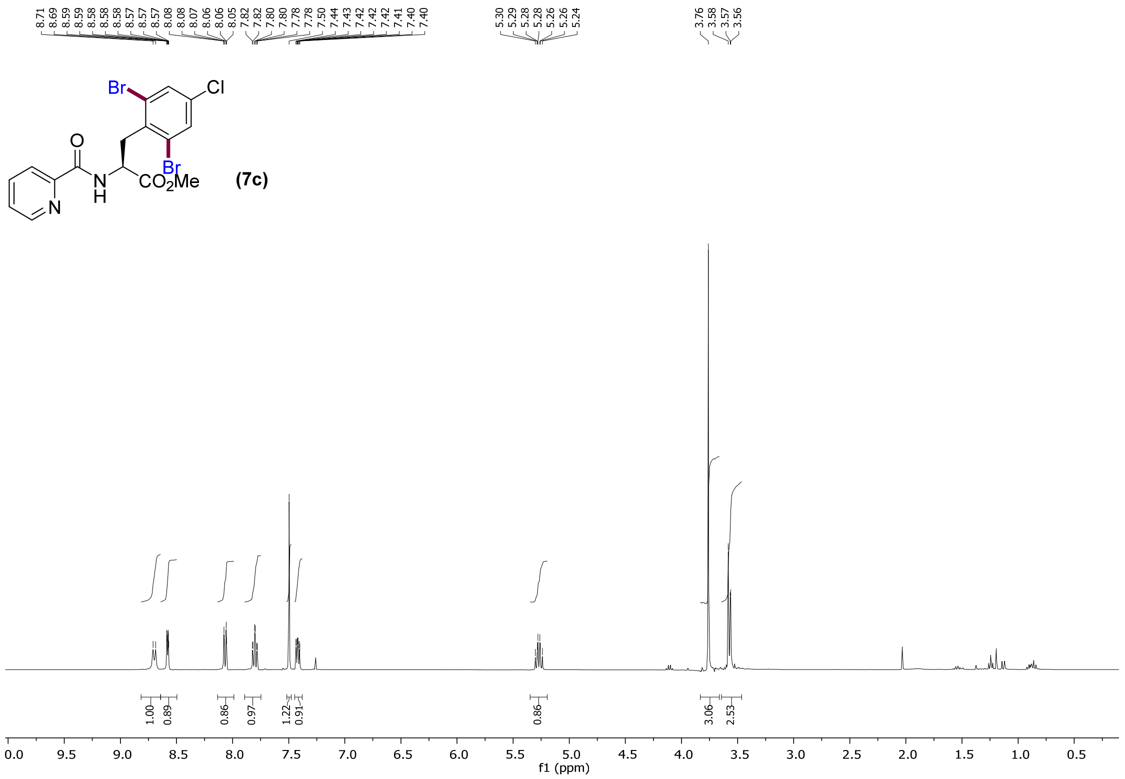
8.71
8.69
8.59
8.59
8.58
8.58
8.57
8.57
8.08
8.08
8.07
8.06
8.06
8.05
7.82
7.82
7.80
7.78
7.78
7.50
7.44
7.43
7.42
7.42
7.41
7.40
7.40
5.30
5.29
5.28
5.28
5.26
5.26
5.24
3.76
3.58
3.57
3.56
Br
Cl
O
N
H
Br
CO2Me
N
(7c)
1.00
0.89
0.86
0.97
1.22
0.91
0.86
3.06
2.53
f1 (ppm)

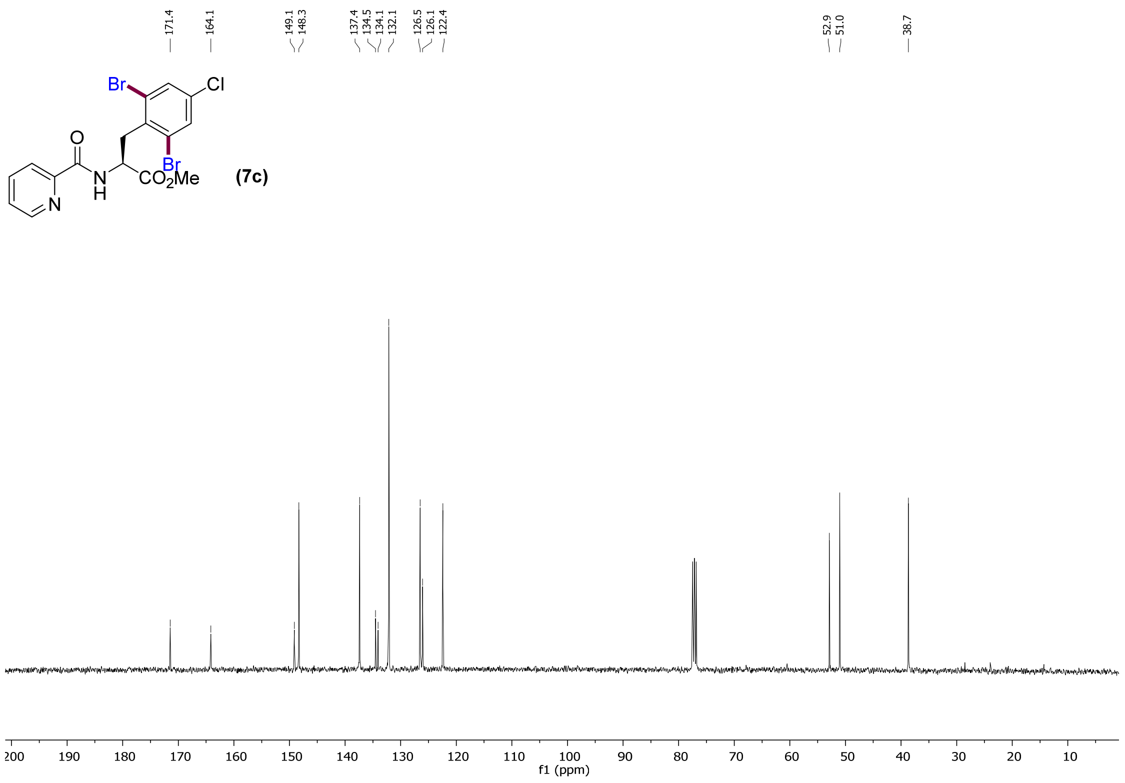
171.4
164.1
149.1
148.3
137.4
134.5
134.1
132.1
126.5
126.1
122.4
52.9
51.0
38.7
Br
Cl
O
N
H
Br
CO2Me
N
(7c)
f1 (ppm)

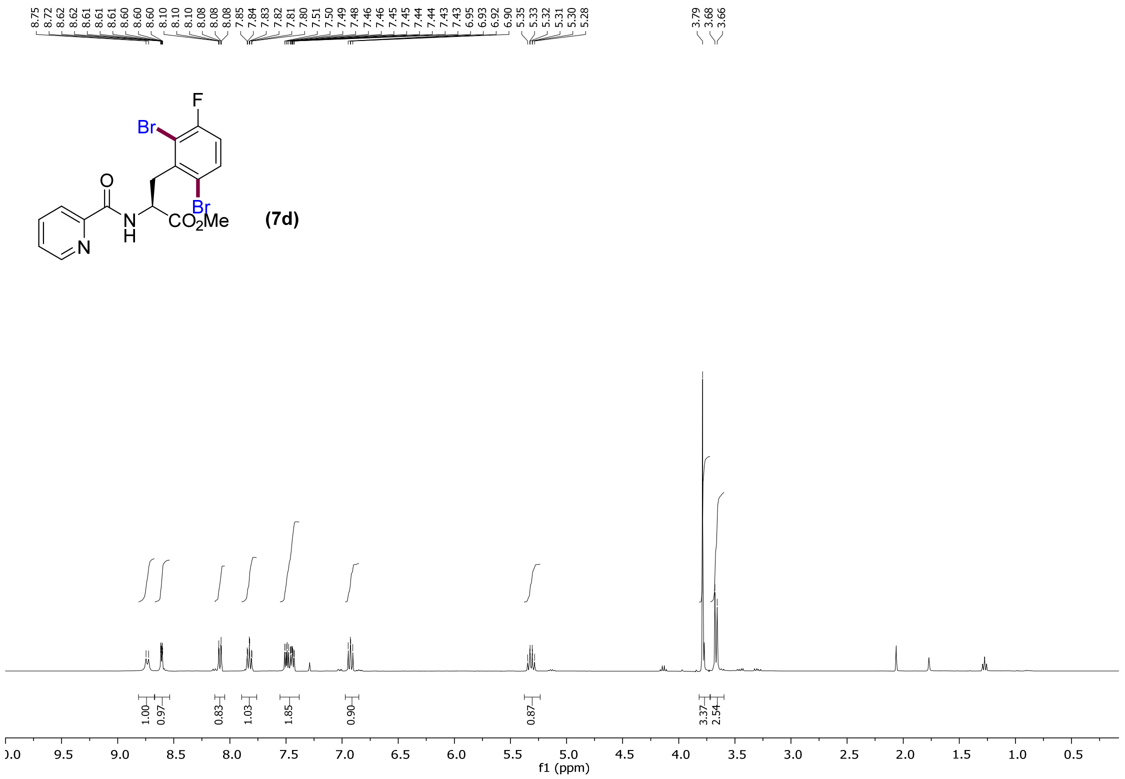
8.75 8.72 8.62 8.62 8.61 8.61 8.61 8.60 8.60 8.60 8.10 8.10 8.10 8.08 8.08 8.08 7.85 7.84 7.83 7.82 7.81 7.80 7.51 7.50 7.49 7.48 7.46 7.46 7.45 7.45 7.44 7.44 7.43 7.43 6.95 6.93 6.92 6.92 6.90 5.35 5.33 5.32 5.31 5.30 5.28
3.79 3.68 3.66
F
Br
Br
O
N
H
N
CO2Me
(7d)
1.00 0.97 0.83 1.03 1.85 0.90 0.87 3.37 2.54
f1 (ppm)

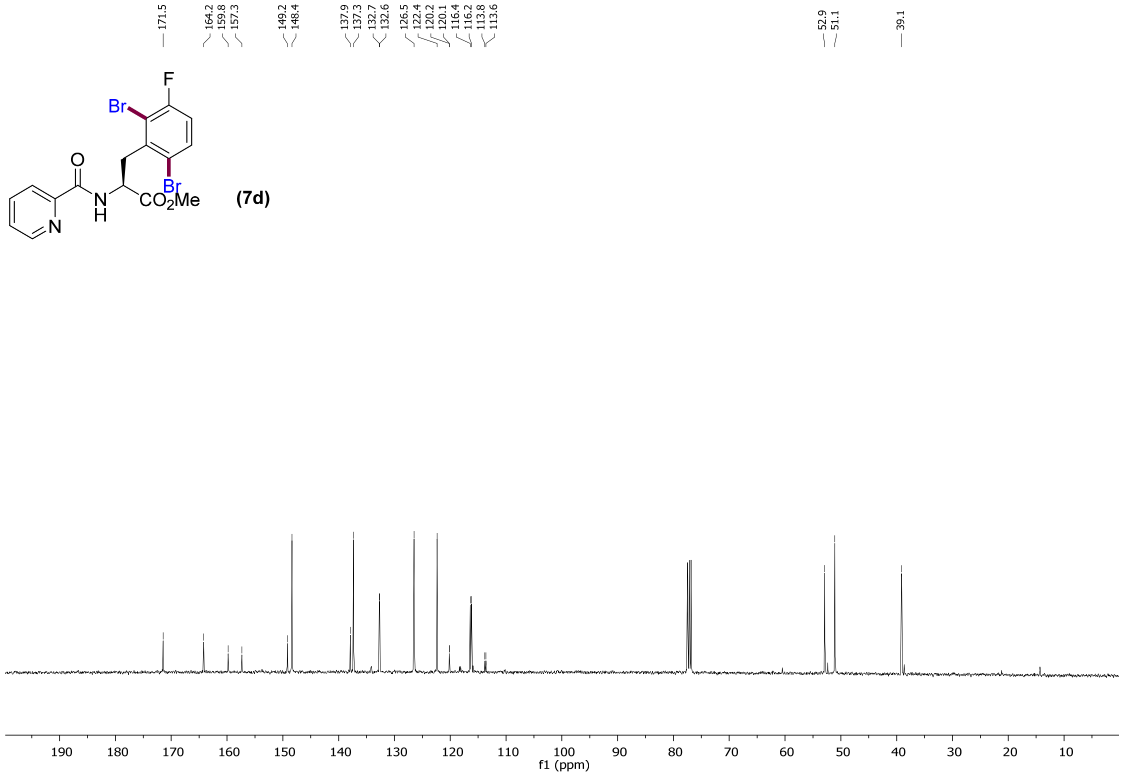
171.5
164.2 159.8 157.3
149.2 148.4
137.9 137.3 132.7 132.6 126.5 122.4 120.2 120.1 116.4 116.2 113.8 113.6
52.9 51.1
39.1
F
Br
Br
O
N
H
N
CO2Me
(7d)
f1 (ppm)

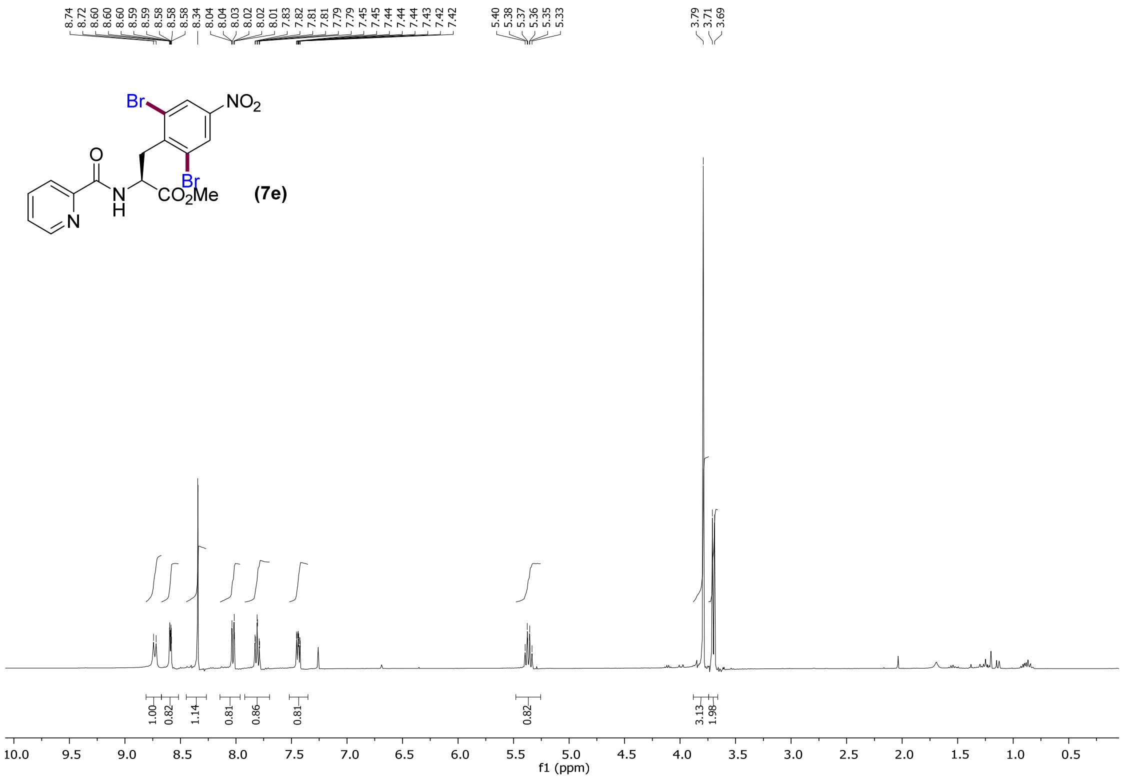
8.74 8.72 8.60 8.60 8.59 8.59 8.58 8.58 8.58 8.34 8.04 8.04 8.03 8.02 8.02 8.01 7.83 7.82 7.81 7.81 7.79 7.79 7.45 7.45 7.44 7.44 7.43 7.42 7.42
5.40 5.38 5.37 5.36 5.35 5.33
3.79 3.71 3.69
Br
NO2
O
Br
N
H
CO2Me
N
(7e)
1.00 0.82 1.14 0.81 0.86 0.81 0.82 3.13 1.98
f1 (ppm)

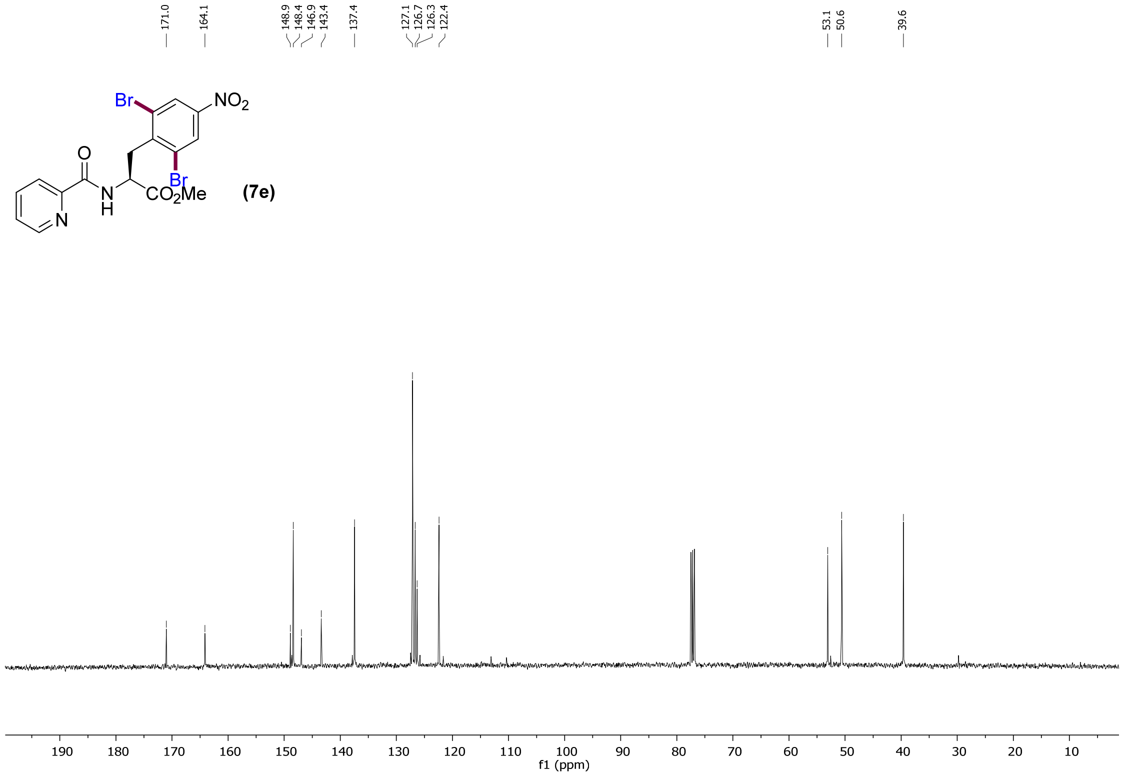
171.0 164.1 148.9 148.4 146.9 143.4 137.4 127.1 126.7 126.3 122.4 53.1 50.6 39.6
Br
NO2
O
Br
N
H
CO2Me
N
(7e)
f1 (ppm)

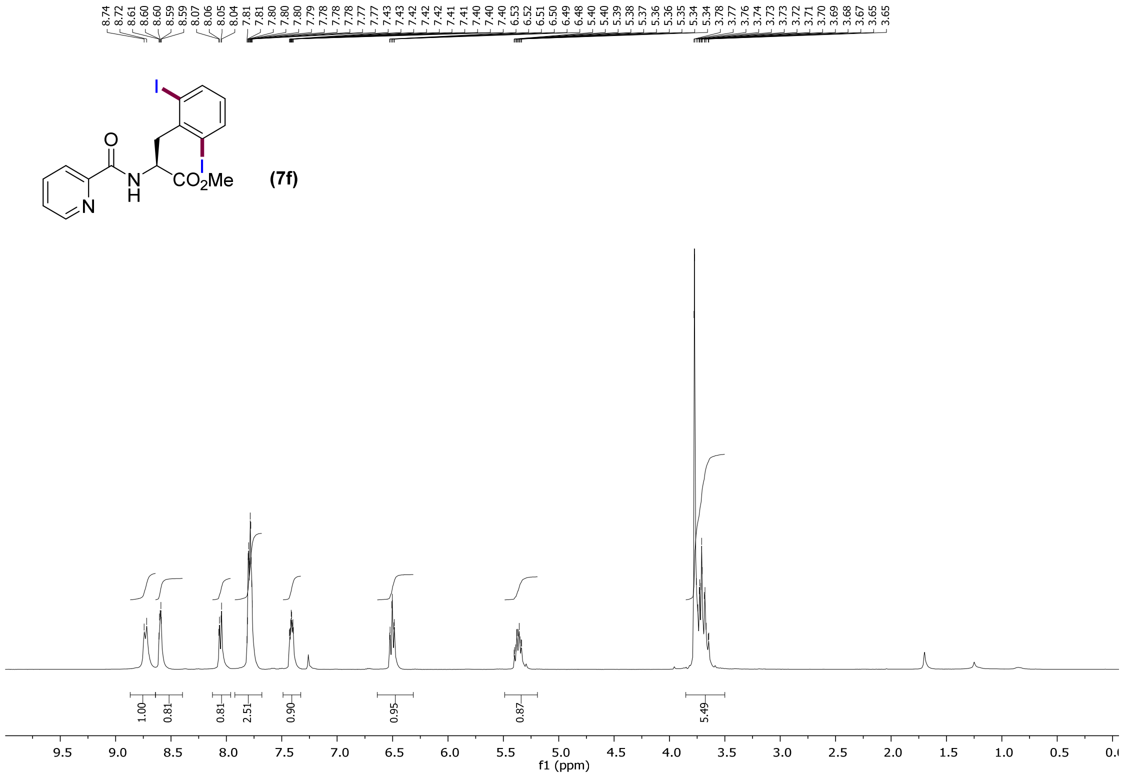
8.74 8.72 8.61 8.60 8.59 8.59 8.07 8.06 8.05 8.04 7.81 7.81 7.80 7.80 7.79 7.78 7.78 7.78 7.77 7.43 7.42 7.42 7.42 7.41 7.41 7.40 7.40 6.53 6.52 6.51 6.50 6.49 6.48 5.40 5.40 5.39 5.38 5.37 5.36 5.35 5.34 3.78 3.77 3.76 3.74 3.73 3.73 3.72 3.71 3.70 3.69 3.68 3.67 3.65 3.65
(7f)
1.00 0.81 0.81 2.51 0.90 0.95 0.87 5.49
f1 (ppm)

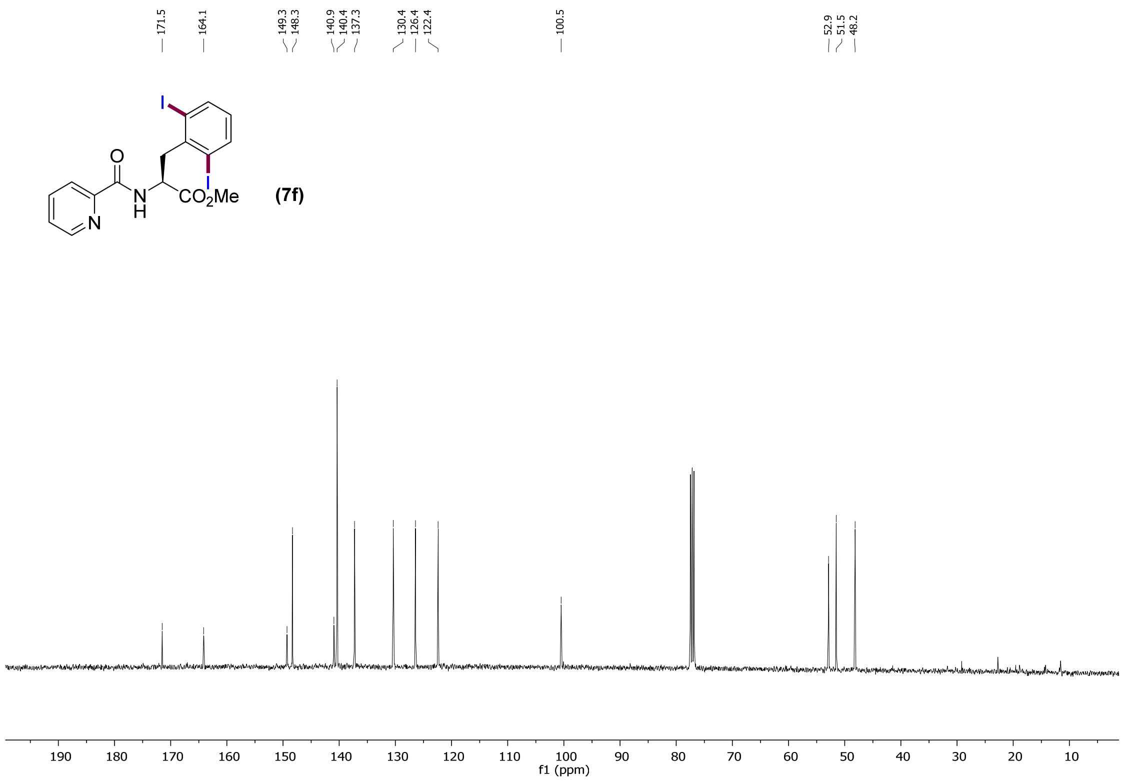
171.5 164.1 149.3 148.3 140.9 140.4 137.3 130.4 126.4 122.4 100.5 52.9 51.5 48.2
(7f)
f1 (ppm)

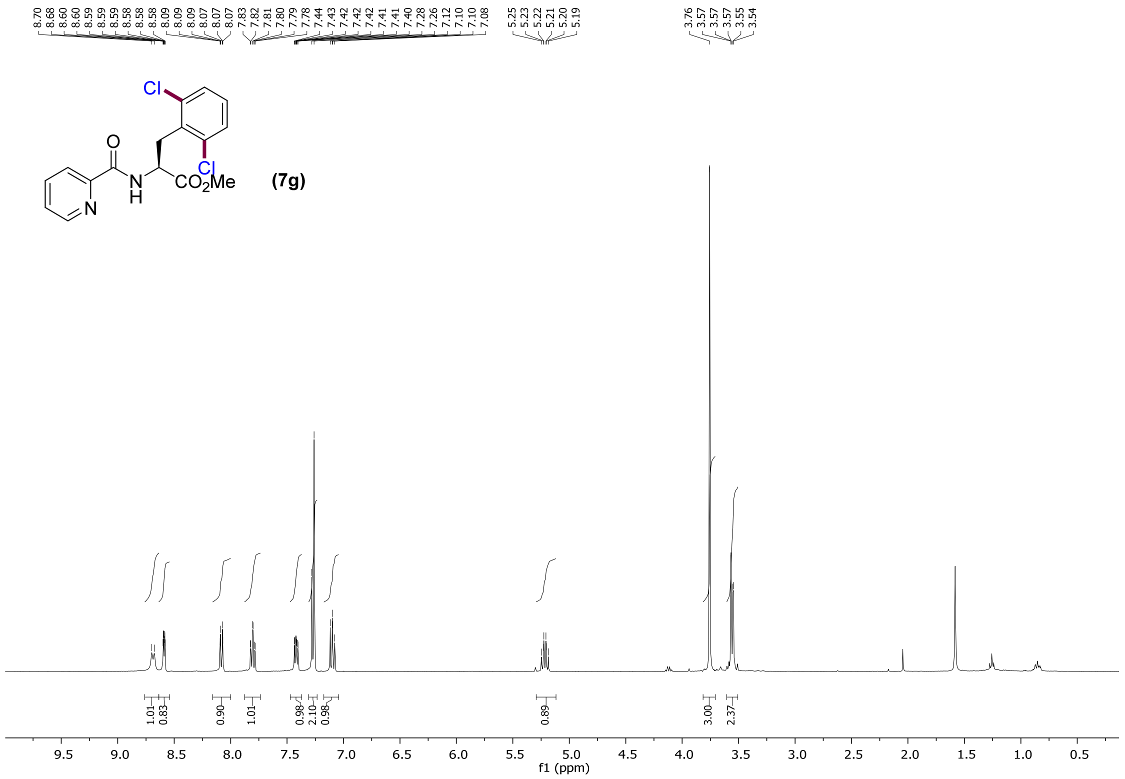
8.70 8.68 8.60 8.59 8.59 8.59 8.58 8.58 8.58 8.09 8.09 8.09 8.07 8.07 8.07 7.83 7.82 7.81 7.80 7.79 7.78 7.44 7.43 7.42 7.42 7.42 7.41 7.41 7.40 7.28 7.26 7.12 7.10 7.10 7.08
5.25 5.23 5.22 5.21 5.20 5.19
3.76 3.57 3.57 3.57 3.55 3.54
Cl
O
Cl
N
H
CO2Me
N
(7g)
1.01 0.83 0.90 1.01 0.98 2.10 0.98 0.89 3.00 2.37
9.5 9.0 8.5 8.0 7.5 7.0 6.5 6.0 5.5 5.0 4.5 4.0 3.5 3.0 2.5 2.0 1.5 1.0 0.5
f1 (ppm)

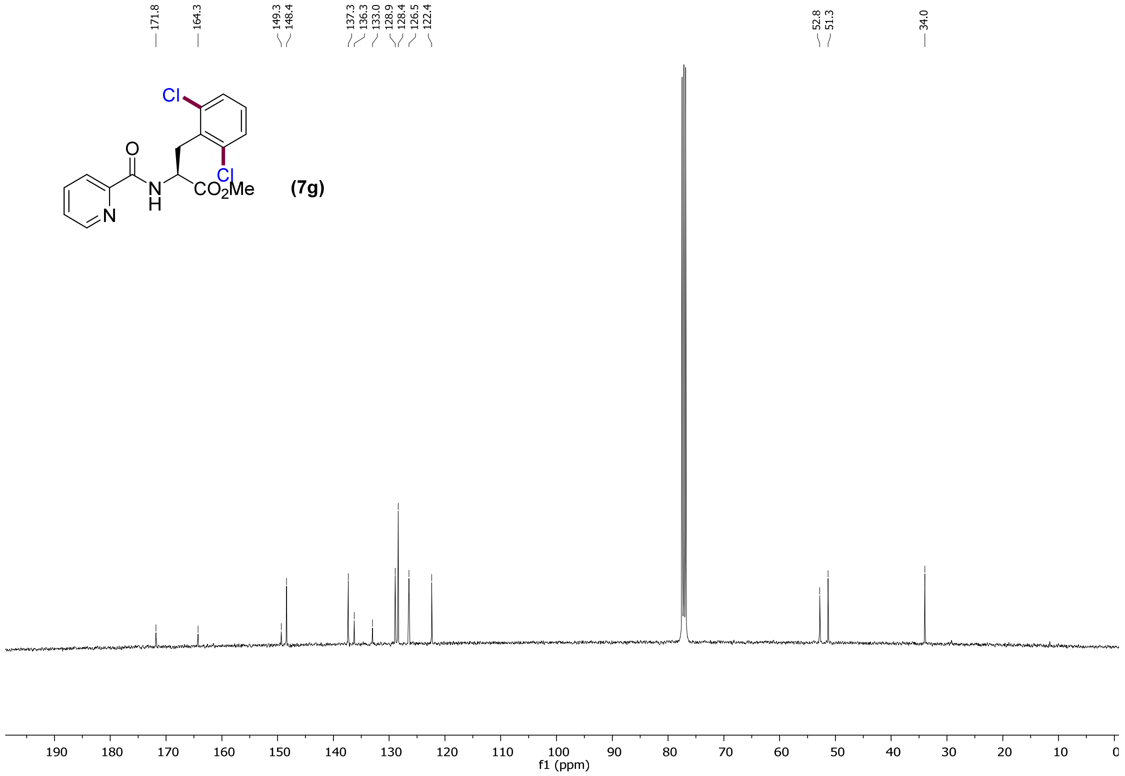
171.8
164.3
149.3 148.4
137.3 136.3 133.0 128.9 128.4 126.5 122.4
52.8 51.3
34.0
Cl
O
Cl
N
H
CO2Me
N
(7g)
190 180 170 160 150 140 130 120 110 100 90 80 70 60 50 40 30 20 10 0
f1 (ppm)

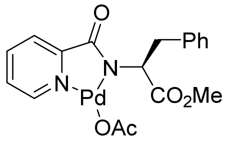

Int-A

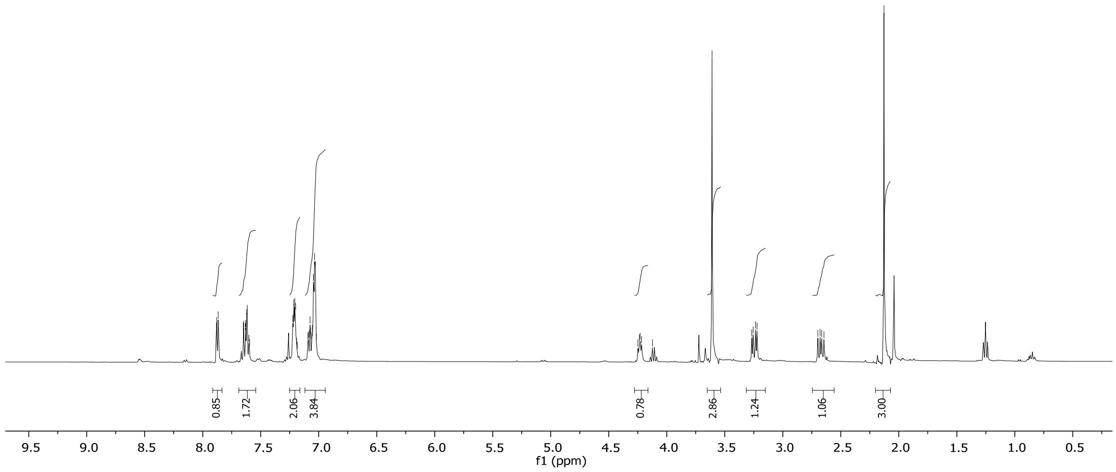
7.88 7.88 7.87 7.65 7.65 7.63 7.63 7.62 7.62 7.60 7.60 7.22 7.22 7.21 7.21 7.20 7.19 7.19 7.09 7.09 7.08 7.07 7.07 7.06 7.06 7.05 7.04 7.03 7.02
4.25 4.24 4.23 4.22 4.12
3.61
3.27 3.26 3.23 3.22
2.70 2.68 2.66 2.64
2.13
0.85 1.72 2.06 3.84 0.78 2.86 1.24 1.06 3.00
9.5 9.0 8.5 8.0 7.5 7.0 6.5 6.0 5.5 5.0 4.5 4.0 3.5 3.0 2.5 2.0 1.5 1.0 0.5
f1 (ppm)

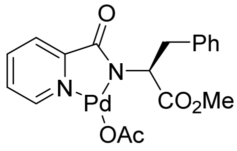

Int-A

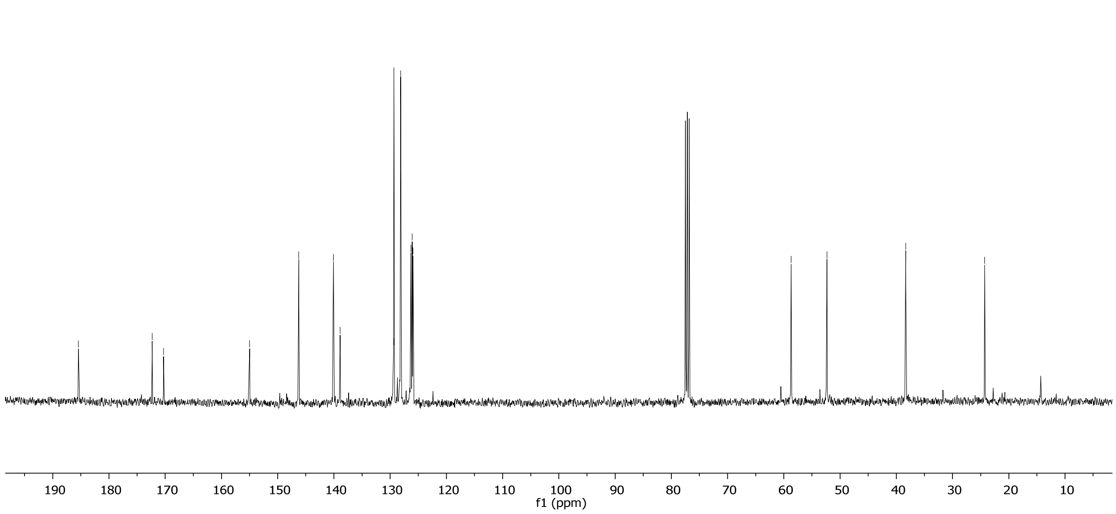
185.4
172.3 170.3
155.0
146.3
140.1 138.9
129.4 129.3 128.1 126.3 126.1 125.9
58.7
52.3
38.3
24.3
190 180 170 160 150 140 130 120 110 100 90 80 70 60 50 40 30 20 10
f1 (ppm)
